# Total Synthesis of the Nominal Structure of (+)-Talaromyolide D

**DOI:** 10.1021/jacs.5c10325

**Published:** 2025-08-14

**Authors:** Bo Qin, Alex Szyperek, Martin Tomanik

**Affiliations:** † Department of Chemistry, 5894 New York University, New York, New York 10003, United States

## Abstract

We describe a convergent and stereoselective total synthesis of the nominal structure of (+)-talaromyolide D (**4**), a recently isolated secondary metabolite. This meroterpenoid features a unique pentacyclic skeleton distinguished by a fused 6/6/6 dihydroisocoumarin core and an unusual pendant dimethylcyclobutanol embedded within a sequence of four contiguous stereocenters. Our synthetic strategy was enabled by a stereoretentive, nickel-catalyzed electrochemical sp^2^–sp^3^ decarboxylative cross-coupling, a two-fold bidirectional stitching sequence comprised of an oxime-directed β-C­(sp^3^)–H arylation and S_N_Ar to establish the central ring system of the target as well as a series of two late-stage carboxylic-acid-directed C­(sp^2^)–H oxidation reactions to ultimately access the isocoumarin lactone substructure. This asymmetric synthesis provides the first access to the reported structure of talaromyolide D (**4**). Comparison of the spectral data showed that the structure of this natural product had been misassigned, and our strategy may present an opportunity for further structural elucidation of the talaromyolide family isolates.

## Graphical abstract

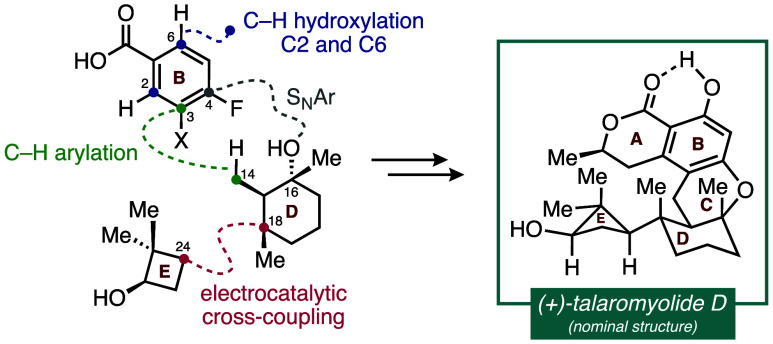

## Introduction

The talaromyolide meroterpenoids A–D (**1**–**4**, Figure 1A) are a small family of natural products isolated from the marine fungus *Talaromyces* sp. CX11 by Wu and co-workers in 2019.^1^ Structurally, the talaromyolides share a common 6/6/6 fused dihydroisocoumarin core and are structurally distinguished by the remaining D–E ring system. Characterization of the bioactivity of **1**–**4** determined that talaromyolide D (**4**) was uniquely active among the family members and displayed antiviral activity against pseudorabies virus (PRV) with a half-maximal cytotoxic concentration (CC_50_) of 3.35 μM.^1^ Notably, talaromyolide D features a rare dimethylcyclobutanol subunit as an equatorial substituent at the C18 position of the D ring, which may contribute to its distinct bioactivity compared to other family members. This substructure is part of a series of four contiguous stereocenters, two of which are quaternary and feature 1,3-diaxial methyl groups locked in a contrathermodynamic orientation. The proposed biosynthesis of **4** is depicted in Figure 1B.^1,2^ Specifically, a phenyltransferase-mediated electrophilic aromatic substitution between farnesyl diphosphate (FPP, **5**) and 6-hydroxymellein (**6**) followed by an acid-catalyzed terpene cyclization was proposed to establish the C–D ring system and provide the tetracyclic intermediate **7**. Oxidation of the pendant C25–C27 alkene residue would provide the epoxide **8,** which upon a regioselective epoxide ring-opening may generate the carbocation intermediate **9**.^3^ Trapping of carbocation **9** with concomitant loss of a proton was proposed to form the cyclobutane of the target.

**Figure 1:**
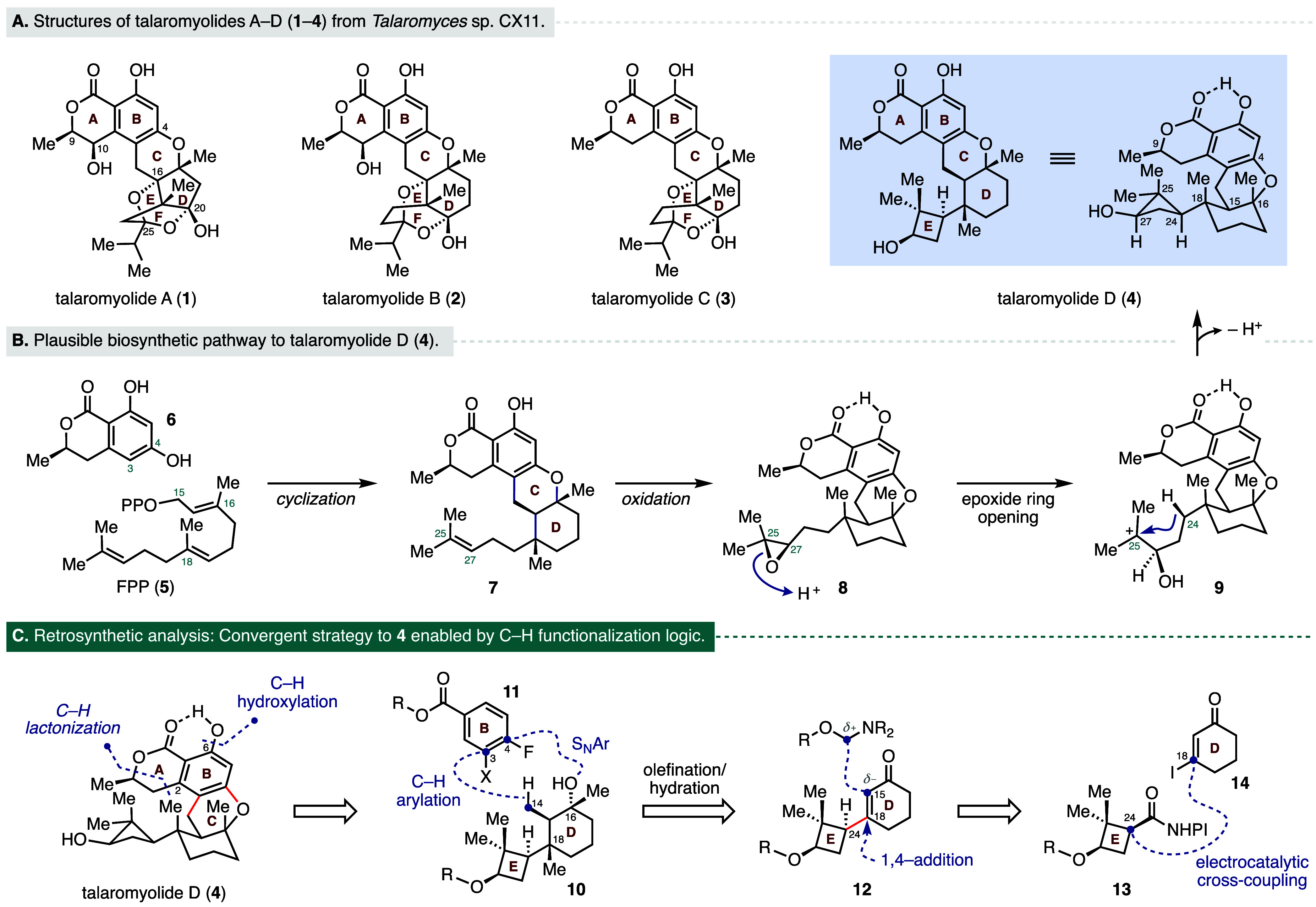
(A) Structures of talaromyolides A–D (**1**–**4**). (B) Plausible biosynthesis of **4**. (C) Retrosynthetic analysis of **4**.

The distinctive skeletal features and stereochemical complexity found in talaromyolide D (**4**) make it an attractive target for chemical synthesis, offering significant value for structural confirmation and further biological evaluation. To date, no synthesis of any talaromyolide family member has been reported. A synthesis of the structurally related chrodrimanin C was recently disclosed by Renata, featuring a 6π electrocyclization/aromatization sequence to construct the dihydroisocoumarin subunit.^4^ From a retrosynthetic perspective, we recognized that talaromyolide D (**4**) provides an opportunity to apply powerful and simplifying C–H activation logic to streamline access to this molecule.^5^ Such disconnections can greatly simplify the preparation of key synthetic intermediates by leveraging innate functional group handles to modify otherwise unreactive or inaccessible C–H positions.^6^ In our retrosynthetic analysis (Figure 1C), we identified a carboxylic acid residue embedded within the A-ring of the lactone that could serve as a directing handle for two separate C–H activation steps. In doing so, we could take advantage of the distinct steric environments to differentiate between the two positions and first perform an *ortho*-C–H hydroxylation at the more accessible C6 position, followed by a second *ortho*-C–H lactonization reaction at the C2 position of compound **4**. Disconnecting the C-ring at the C3–C14 bond and the C4–O bond maximizes the convergence of the route and reveals bifunctional fragments **10** and **11**, which could be sequentially stitched together via a β-C­(sp^3^)–H arylation and an intramolecular S_N_Ar cyclization strategy. The fragment **10** possessing four contiguous stereocenters could be accessed from the E–D bicycle **12** via a series of carefully orchestrated transformations that include an olefination–hydration sequence and a tandem 1,4-addition and enolate trapping. Importantly, we recognized that the critical C18–C24 bond joining the E and D ring system could be formed via a nickel-catalyzed electrochemical decarboxylative sp^2^–sp^3^ cross-coupling between the redox-active ester located on the cyclobutane ring of **13** and β-iodoenone **14**. The stereochemical outcome of this radical coupling could potentially be derived from the conformational bias of the cyclobutane ring.

## Results and Discussion

The synthesis of talaromyolide D (**4**) began with the preparation of the *cis*-substituted cyclobutane fragment **16**. This fragment was prepared in three steps on a decagram scale from (−)-verbenone (**15**) (Scheme 1). Oxidative cleavage^7^ of the alkene residue (ruthenium trichloride, sodium periodate) and subsequent treatment of the unpurified reaction mixture with *meta*-chloroperoxybenzoic acid (*m*-CPBA) provided the expected C27 Baeyer–Villiger oxidation product (not shown). The resulting C24 carboxylic residue was then converted to the redox-active *N*-hydroxyphthalimide (NHPI) ester **16** by coupling with *N*-hydroxyphthalimide mediated by 1-ethyl-3-(3-(dimethylamino)­propyl)­carbodiimide (EDC; 71% from **15**). With ample quantities of fragment **16**, we next investigated the key cross-coupling between **16** and vinyl iodide **17**. To this end, we found that Baran’s nickel catalyzed decarboxylative electrochemical cross-coupling enabled by silver nanoparticle-modified electrodes provided the desired E–D bicycle **20** in 85% yield and in 11:1 dr at the C24 position.^8^ The stereochemistry of the cross-coupled product was established by conversion of **20** to the crystalline alcohol **21** (potassium hydroxide, methanol, 97%). This sp^2^–sp^3^ radical cross-coupling reaction takes place in the absence of any chiral ligands at the nickel center and delivers the desired coupled product with excellent stereocontrol. We hypothesize that the high level of stereocontrol observed during the coupling reflects the favorable conformation of the cyclobutane radical, as depicted in structure **18**. This conformation minimizes nonbonding interactions between the acetate and the C25 methyl group while maximizing overlap between the radical SOMO and the adjacent C–C bond.^9^ Recent work by Diao and co-workers demonstrated that the binding of a secondary radical to a nickel­(II)–aryl species is a relatively slow process (*k* = 10^6^ M^–1^ s^–1^) and is also sensitive to the steric environment surrounding the nickel catalyst.^10^ This implies that free radical racemization (picosecond time scale) should outcompete nickel-mediated radical capture in the absence of any substrate-conferred stability.^11^ To verify our hypothesis, we prepared redox-active ester **16** and its C24-epimer as a 1:1.4 mixture via an alternative ring contraction strategy (see Supporting Information). Exposure of this mixture of diastereomers to the standard reaction conditions provided the E–D bicycle **20** in 83% yield with an identical 11:1 dr at C24 (Scheme 2A). This result indicates that the secondary radical **18** preferentially reacts via the more accessible convex face of the Ni­(II) species **19**, through either a stereoretentive stepwise or concerted inner-sphere mechanism to deliver the desired enone **20**.^12,10^ This four-step sequence provides expedient access to the E–D rings systems of **4** in three steps and 60% overall yield from commercial reagents.

**Scheme 1:**
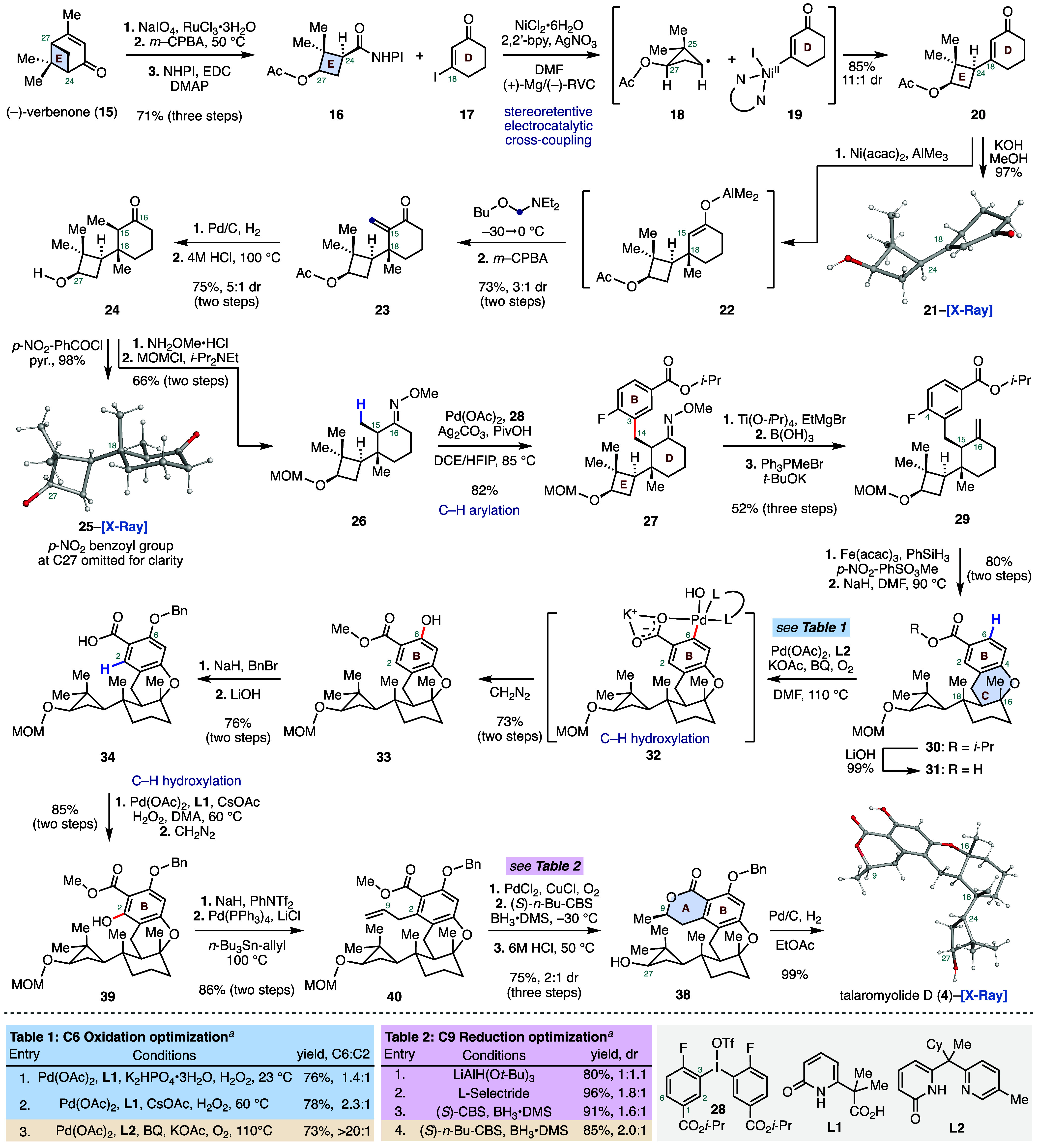
Total Synthesis of the Nominal Structure of (+)-Talaromyolide D (**4**)

a Yields were determined by ^1^H NMR analysis using CH_2_Br_2_ as an internal standard.

**Scheme 2:**
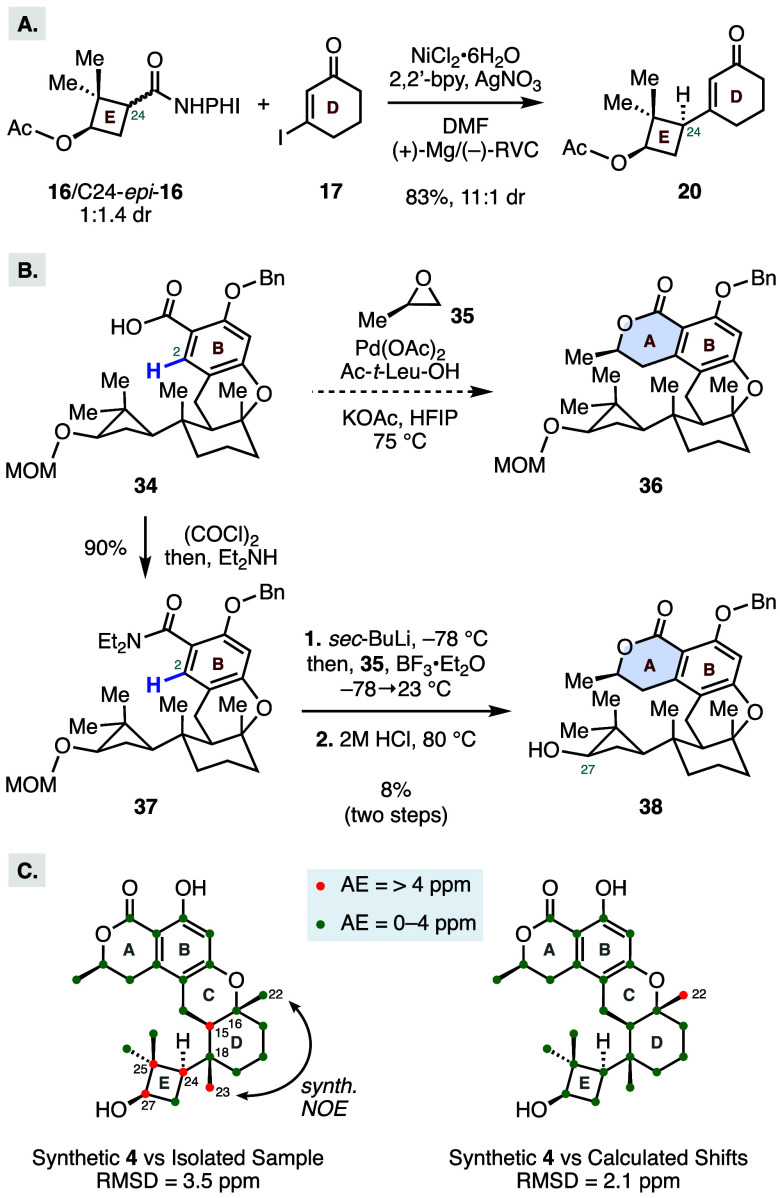
(A) Electrochemical Cross-Coupling with a Mixture of C24 Diastereomers; (B) Selected Efforts toward Construction of the Lactone A-Ring of **4**; (C) ^13^C NMR Comparison Data between Isolated and Synthetic Talaromyolide D Samples^a^

a Root-mean-square deviation (RMSD).

The bicycle **20** was elaborated by a three-component protocol starting first with a diastereoselective nickel-catalyzed conjugate addition of trimethylaluminum to the sterically hindered C18 position of the α,β-unsaturated ketone (**20** → **22**).^13^ In situ trapping of the resulting aluminum enolate **22** with *N*-(methoxymethyl)-diethylamine,^14^ followed by oxidative elimination of tertiary diethyl amine group (*m*-CPBA) provided the exocyclic α,β-unsaturated ketone **23** in 73% yield as a 3:1 ratio of diastereomers at the C18 position (^1^H NMR analysis). The configuration of the newly formed stereocenter was established by X-ray analysis of benzoate **25** (vide infra). Efforts to improve the stereoselectivity by using copper salts and chiral phosphoramidite ligands were ineffective (see the Supporting Information). Fortunately, the diastereomeric mixture was separated by flash-column chromatography on preparative scales. Reduction of the alkene residue (palladium on carbon, dihydrogen) and exposure of the reaction mixture to hydrogen chloride at elevated temperatures (100 °C) provided the *cis*-dimethyl ketone **24** with 5:1 diastereoselectivity at the C15 position in 75% yield (^1^H NMR analysis). Notably, the acidic treatment was critical to achieve high levels of diastereoselectivity, as upon enolization of the α-proton the reprotonation occurs so that the C15 methyl group is oriented *anti* to the steric bulk of the cyclobutane moiety. The stereochemistry of **24** was confirmed by acylation of the C27 alcohol residue with *para*-nitrobenzoyl chloride, which provided the crystalline **25** with the desired *cis*-dimethyl groups.

The next stages of our synthesis focused on the formation of the central C-ring of talaromyolide D (**4**). We envisioned achieving this via a bidirectional stitching of an aromatic subunit to bicycle **24** at C14 and C16. To this end, condensation of *O*-methylhydroxylamine with the C15 carbonyl and protection of the C27 alcohol (methoxymethyl chloride, Hünig’s base) delivered the oxime ether **26** in 66% yield over two steps. The aromatic group was then introduced to our substrate via efficient C­(sp^3^)–H arylation of the C15 methyl group directed by the proximal oxime ether group.^15^ Specifically, exposure of **26** to diaryliodonium salt **28** in the presence of palladium acetate, silver carbonate, and substoichiometric amounts of pivalic acid smoothly delivered the β-arylated product **27** in 82% yield.

We next attempted to remove the oxime ether directing group under acidic conditions, but this resulted in the unwanted epimerization of the stereochemistry at the C15 position. Similarly, efforts to perform selective oxidative cleavage of the oxime ether with photoexcited nitroarenes or with ozone were inefficient (see the Supporting Information).^16^ To overcome this, we employed low-valent titanium (titanium isopropoxide, ethylmagnesium bromide) to reductively cleave the N–O bond of the oxime ether and furnish the corresponding imine, which could be hydrolyzed to the desired ketone by the addition of boric acid.^17^ Subsequent Wittig olefination (potassium *tert*-butoxide, methyl triphenylphosphonium bromide) delivered the expected alkene product **29** (52%, three steps). Next, a two-step sequence comprising a diastereoselective iron-catalyzed Mukaiyama-type alkene hydration^18^ and intramolecular S_N_Ar (sodium hydride) of the generated tertiary alcohol with the pendant aryl-fluoride constructed the C-ring of the target and provided the tetracycle **30** in 80% yield over two steps. The Mukaiyama hydration provided the desired C16 tertiary alcohol as a single detectable diastereomer (^1^H NMR analysis) and the diastereoselectivity was determined by strong NOE correlations between the C16 and C18 methyl groups in the annulated product **31**.

Having completed four of the five rings present in talaromyolide D, our efforts focused on 2-fold C–H functionalization of the aromatic B ring of **30**. To this end, we first hydrolyzed the *iso-*propyl ester to reveal the corresponding carboxylic acid residue, which we envisioned could direct C–H activation preferentially at the less hindered C6 position, followed by a second C–H activation reaction at the more sterically hindered *ortho*-C2 site to systematically functionalize the B-ring. Our initial attempts to affect a palladium catalyzed C–H hydroxylation at the C6 position focused on employing hydrogen peroxide as the “oxygen” source in combination with Yu’s CarboxPyridone ligand **L1** (Table 1, Entry 1 and 2).^19^ While these conditions were able to successfully oxidize our aromatic ring, the selectivity between the two *ortho*-positions was only modest, favoring the desired C6 oxidation. However, employing molecular oxygen along with the tautomeric pyridine–pyridone ligand **L2** and 1,4-benzoquinone at elevated reaction temperate furnished the desired C6 hydroxylated methyl ester **33** after treatment with diazomethane as the C6 exclusive regioisomer in 73% yield.^20^ Elaboration of **33** via protection of the phenol residue (sodium hydride, benzyl bromide) and hydrolysis of the ester gave acid **34** (76% over two steps). We then envisioned constructing the final lactone ring of **4** via a C–H alkylation–lactonization sequence (Scheme 2B).^21^ However, our attempts to perform a palladium-catalyzed alkylation at the C2 position with epoxide **35** were unsuccessful. In all cases, the reaction only returned unreacted starting material or we observed decomposition of **34** at elevated reaction temperatures. A strategy based on directed lithiation was also explored but this approach delivered only limited quantities of the desired lactone **38** (Scheme 2B).^22^

To address this difficult challenge of introducing the desired carbon–carbon bond at the C2 position, we pursued a cross-coupling-based approach (Scheme 1). First, we performed a second C–H hydroxylation to arrive at the C2 hydroxylated species **39** (85%).^19^ This was followed by a triflation (sodium hydride and phenyl triflimide) and a Stille cross-coupling with allyltributylstannane to provide the C2 allyl product **40** in 86% yield over two steps. Wacker–Tsuji oxidation (palladium dichloride, copper­(I) chloride, oxygen) delivered a C9 methyl ketone (not shown), which was reduced to the requisite secondary alcohol with 2:1 diastereoselectivity (^1^H NMR analysis) utilizing a Corey–Bakshi–Shibata reduction ((*S*)-2-butyl-CBS-oxazaborolidine, borane dimethyl sulfide, 2:1 dr at C9).^23^ Notably, while performing this reduction, we first identified that bulky hydride-reducing agents (LiAlH­(O*t*-Bu)_3_ or l–selectride) favored the desired secondary alcohol diastereomer (Scheme 1, Table 2). In our attempts to improve the diastereoselectivity, we sought to explore a reagent-controlled reductions with CBS catalysts.

As seen in Table 2, we found that the larger 2-butyl containing oxazaborolidine provided improved selectivity. As the secondary alcohol product was found to undergo a partial translactonization, the unpurified reaction mixture was directly treated with hydrogen chloride to complete the formation of the lactone ring and simultaneously remove the C27 methoxymethyl ether, providing the lactone **38** (75% over three steps). Finally, cleavage of the benzyl protecting group (palladium on carbon and dihydrogen) delivered synthetic talaromyolide D (**4**) in 99% yield. X-ray crystallographic analysis of **4** served to confirm its structure as well as all of the established stereoselectivity.

Surprisingly, NMR spectroscopic data of synthetic **4** were not in agreement with the reported spectrum for the isolated sample (see the Supporting Information). Close inspection of the ^13^C NMR spectra identified a total of five carbons with large discrepancies (absolute error (AE) = >4 ppm, Scheme 2C). The chemical shifts in question are proximal to one another and are located around or as part of the cyclobutane E-ring. Additionally, our synthetic sample, as expected, displayed strong NOE correlations between the two diaxial methyl substituents at C16 and C18 that are noticeably absent in the reported ROESY experiment performed on the isolated sample. This data strongly suggest that one or more stereocenters in the isolated sample are misassigned. We attempted to investigate the stereochemical assignment using the NMR calculation protocol reported by Hehre.^24^ First, we calculated the ^13^C chemical shifts for the reported structure and compared it with our synthetic sample (Scheme 2C). We found that all shifts are in good agreement with a root-mean-square deviation (RMSD) of 2.1 ppm, except for a sole–CH_3_ carbon with AE > 4 ppm. Next, we evaluated a total of 33 structures by systematically altering one stereocenter at a time around the D–E ring system in hopes of identifying a stereochemical arrangement that would be in good agreement with the reported spectrum (see the Supporting Information). However, in all cases, there were five or more discrepancies (absolute error >4 ppm) between the calculated and reported ^13^C NMR spectra. While this method is powerful for supporting bond connectivity assignments, the subtle differences in NMR shifts typically exhibited by diastereomers limits the ability to make definitive stereochemical assignments.^25^ Nevertheless, the numerous large deviations observed in our calculations suggest that multiple stereocenters may be misassigned and that further revisions to the proposed bond connectivity may be necessary to correctly identify the structure of the isolated compound.

## Conclusion

In summary, we have described the first synthetic route to the nominal structure of pentacyclic meroterpenoid talaromyolide D (**4**). Key features of our convergent synthetic strategy include three palladium-catalyzed C–H activation reactions, a stereoretentive nickel-catalyzed electrochemical decarboxylative cross-coupling, a highly diastereoselective iron-catalyzed Mukaiyama-type alkene hydration, and an intramolecular *O*-arylation via an S_N_Ar reaction to construct the central ring system. Spectroscopic data revealed that the structure of this natural product had been misassigned during isolation, and our ^13^C NMR calculations suggest that a substantial revision of the proposed structures may be necessary to determine the correct structure of the talaromyolide D isolate.

## Supplementary Material



## Notes

General information, experimental procedures, substrate structures; characterization of substrates and products; crystallographic analysis of **21**, **25** and **4**; catalogue of ^1^H NMR and ^13^C NMR spectra (PDF)

## References

[1] Cao X., Shi Y., Wu X., Wang K., Huang S., Sun H., Dickschat J. S., Wu B. (2019). Talaromyolides A–D and Talaromytin:
Polycyclic Meroterpenoids from the Fungus Talaromyces sp. CX11. Org. Lett..

[2] Awakawa T. (2025). Biosynthesis
of unique natural product scaffolds by Fe­(II)/αKG-dependent
oxygenases. J. Nat. Med..

[3] Matsuda Y., Abe I. (2016). Biosynthesis of fungal
meroterpenoids. Natural Product Reports.

[4] Li F., Renata H. (2021). A Chiral-Pool-Based
Strategy to Access trans-syn-Fused
Drimane Meroterpenoids: Chemoenzymatic Total Syntheses of Polysin,
N-Acetyl-polyveoline and the Chrodrimanins. J. Am. Chem. Soc..

[5] Gutekunst W. R., Baran P. S. (2011). C–H functionalization logic
in total synthesis. Chem. Soc. Rev..

[6] Gutekunst W. R., Baran P. S. (2011). Total Synthesis
and Structural Revision
of the Piperarborenines via Sequential Cyclobutane C–H Arylation. J. Am. Chem. Soc..

[7] Tercio J., Ferreira B., Cruz W. O., Vieira P. C., Yonashiro M. (1987). Carbon-carbon double bond cleavage
using solid-supported
potassium permanaganate on silica gel. J. Org.
Chem..

[8] Harwood S. J., Palkowitz M. D., Gannett C. N., Perez P., Yao Z., Sun L., Abruña H. D., Anderson S. L., Baran P. S. (2022). Modular
terpene synthesis enabled by mild electrochemical couplings. Science.

[9] Cremer D. (1977). Ab initio calculations of the equilibrium structure
of cyclobutane. J. Am. Chem. Soc..

[10] Spielvogel E. H., Yuan J., Hoffmann N. M., Diao T. (2025). Nickel-Mediated Radical
Capture: Evidence for a Concerted Inner-Sphere Mechanism. J. Am. Chem. Soc..

[11] Johnston L. J., Ingold K. U. (1986). Kinetics of cyclopropyl
radical reactions. 2. Studies on the inversion of cyclopropyl and
1-methylcyclopropyl radicals and on the kinetics of some addition
and abstraction reactions of 1-methylcyclopropyl and 1-methoxycyclopropyl
radicals. J. Am. Chem. Soc..

[12] Lin Q., Spielvogel E. H., Diao T. (2023). Carbon-centered radical
capture at
nickel­(II) complexes: Spectroscopic evidence, rates, and selectivity. Chem..

[13] Flemming S., Kabbara J., Nickisch K., Neh H., Westermann J. (1995). Nickel-Catalysed
Conjugate Addition of Trimethylaluminum to Sterically Hindered α,β-Unsaturated
Ketones. Synthesis.

[14] Bleschke C., Tissot M., Müller D., Alexakis A. (2013). Direct Trapping of
Sterically Encumbered Aluminum Enolates. Org.
Lett..

[15] Peng J., Chen C., Xi C. (2016). *β*-Arylation
of oxime ethers using diaryliodonium salts through activation of inert
C­(sp^3^)–H bonds using a palladium catalyst. Chem. Sci..

[16] Göttemann L. T., Wiesler S., Sarpong R. (2023). Oxidative cleavage of ketoximes to
ketones using photoexcited nitroarenes. Chem.
Sci..

[17] Corey E. J., Niimura K., Konishi Y., Hashimoto S., Hamada Y. (1986). A new synthetic route to prostaglandins. Tetrahedron Lett..

[18] Bhunia A., Bergander K., Daniliuc C. G., Studer A. (2021). Fe-Catalyzed Anaerobic
Mukaiyama-Type Hydration of Alkenes using Nitroarenes. Angew. Chem., Int. Ed..

[19] Li Z., Park H. S., Qiao J. X., Yeung K.-S., Yu J.-Q. (2022). Ligand-Enabled
C–H Hydroxylation with Aqueous H_2_O_2_ at
Room Temperature. J. Am. Chem. Soc..

[20] Li Z., Wang Z., Chekshin N., Qian S., Qiao J. X., Cheng P. T., Yeung K.-S., Ewing W. R., Yu J.-Q. (2021). A tautomeric
ligand enables directed C–H hydroxylation with molecular oxygen. Science.

[21] Cheng G., Li T.-J., Yu J.-Q. (2015). Practical Pd­(II)-Catalyzed C–H
Alkylation with Epoxides: One-Step Syntheses of 3,4-Dihydroisocoumarins. J. Am. Chem. Soc..

[22] Shankaran K., Snieckus V. (1984). Directed ortho metalation-induced
epoxy cyclialkylation. Regiospecific 5-*exo*-tet and
6-*exo*-tet routes to benzofurans and -pyrans. J. Org. Chem..

[23] Corey E. J., Helal C. J. (1998). Reduction of Carbonyl Compounds with Chiral Oxazaborolidine
Catalysts: A New Paradigm for Enantioselective Catalysis and a Powerful
New Synthetic Method. Angew. Chem., Int. Ed..

[24] Hehre W., Klunzinger P., Deppmeier B., Driessen A., Uchida N., Hashimoto M., Fukushi E., Takata Y. (2019). Efficient Protocol
for Accurately Calculating ^13^C Chemical Shifts of Conformationally
Flexible Natural Products: Scope, Assessment, and Limitations. J. Nat. Prod..

[25] Lodewyk M. W., Siebert M. R., Tantillo D. J. (2012). Computational Prediction
of ^1^H and ^13^C Chemical Shifts: A Useful Tool
for Natural
Product, Mechanistic, and Synthetic Organic Chemistry. Chem. Rev..

