# Supplementary material for: Total Synthesis of the Nominal Structure of (+)-Talaromyolide D
Source: J Am Chem Soc. 2025 Aug 14;147(34):31221–7. doi: 10.1021/jacs.5c10325 (PMC12395475; doi:10.1021/jacs.5c10325)

Total Synthesis of the Nominal Structure of (+)-Talaromyolide D

Bo Qin[1], Alex Szyperek[1], and Martin Tomanik*,[1]

[1]Department of Chemistry, New York University, New York, New York 10003, United States.

Supporting information

Table of Contents

Supplementary Tables and Figures. S2
General Information. S15
Experimental Procedures. S16
Comparison of Spectroscopic Data of Reported and Synthetic (+)-Talaromyolide d (**4**)......S52
Crystallographic Analysis of Alcohol **21**. S55
Crystallographic Analysis of Nitroarene **25**. S57
Crystallographic Analysis of Talaromyolide D (**4**). S59
References S61
Catalogue of ^{1}H NMR and ^{13}C NMR spectra. S62

Supplementary Tables and Figures.

Table S1. Copper-catalyzed asymmetric conjugate addition.[1]

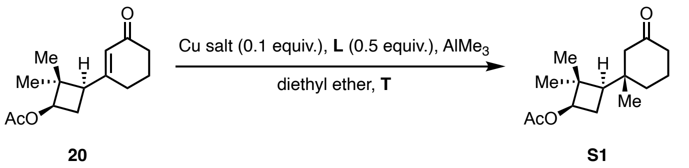

Entry	Cu salt	Ligand	T/°C	$AlMe_3$	Yield/%	d.r.
1	CuTc	**L1**	−20	4.0 equiv.	30	1:1
2	$(CuOTf)_2·C_6H_6$	**L1**	−20	4.0 equiv.	58	2.6:1
3	$(CuOTf)_2·C_6H_6$	**L1**	−25	2.5 equiv.	50	2.1:1
4	$(CuOTf)_2·C_6H_6$	**L6**	−30	2.0 equiv.	46	2.6:1

Note: The d.r. value was established via analysis of the ^{1}H NMR spectrum.

Table S2. Copper-catalyzed asymmetric conjugate addition.[1]

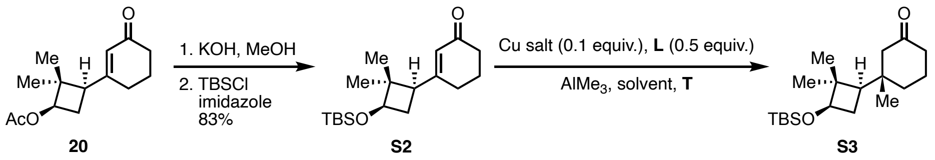

Entry	Cu salt	Ligand	T/°C	$AlMe_3$	solvent	Yield/%	d.r.
1	CuTc	**L1**	−10	4.0 equiv.	Et_2O	35	1.2:1
2	$Cu(MeCN)_4·BF_4$	**L1**	−20	4.0 equiv.	THF	0	N.D.
3	$(CuOTf)_2·C_6H_6$	**L2**	−30	4.0 equiv.	Et_2O	69	1.2:1
4	$(CuOTf)_2·C_6H_6$	**L3**	−35	4.0 equiv.	Et_2O	65	2.2:1
5	$(CuOTf)_2·C_6H_6$	**L4**	−35	4.0 equiv.	Et_2O	67	2.1:1
6	$(CuOTf)_2·C_6H_6$	**L5**	−30	2.2 equiv.	Et_2O	63	2.4:1
7	$(CuOTf)_2·C_6H_6$	**L6**	−30	2.2 equiv.	Et_2O	60	3.5:1

Note: The d.r. value was established via analysis of the ^{1}H NMR spectrum.

Figure S1. Ligands **L1-L6** for the asymmetric conjugate addition.[1]

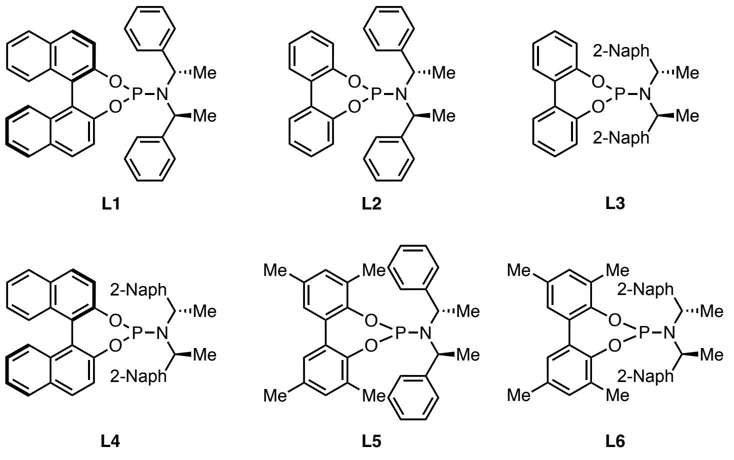

Table S3. Unsuccessful conditions for oxime cleavage.[2-6]

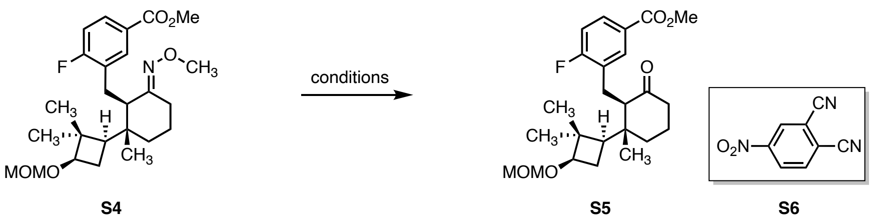

Entry	Conditions	Yield/%
1	O_3, CH_2Cl_2, −78 °C; then DMS	decomposed
2	**S6**, *hv* (390 nm), MeCN/H_2O	30
3	**S6**, *hv* (427 nm), MeCN/H_2O	30
4	Raney Ni, H_2, $B(OH)_3$, MeOH/H_2O, 45 °C	10*
5	Fe, NH_4Cl, EtOH/H_2O, 80 °C	0
6	$Mo(CO)_6$, H_2O, MeCN, 80 °C	0

* The imine intermediate was reduced to the corresponding amine before hydrolysis.

Figure S2. Failed synthesis of intermediate **24** employing enone **S7** bearing a preinstalled C15 methyl group.

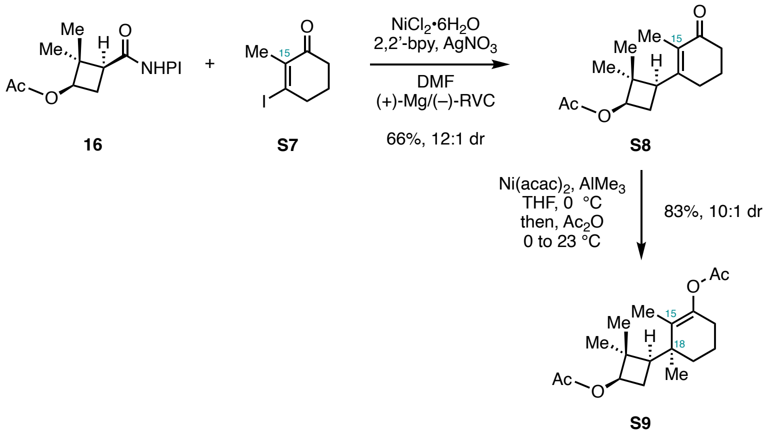

Figure S3. ^{13}C NMR comparison data between the isolated data for talaromyolide D (**4**) and calculated data for 33 possible isomers.

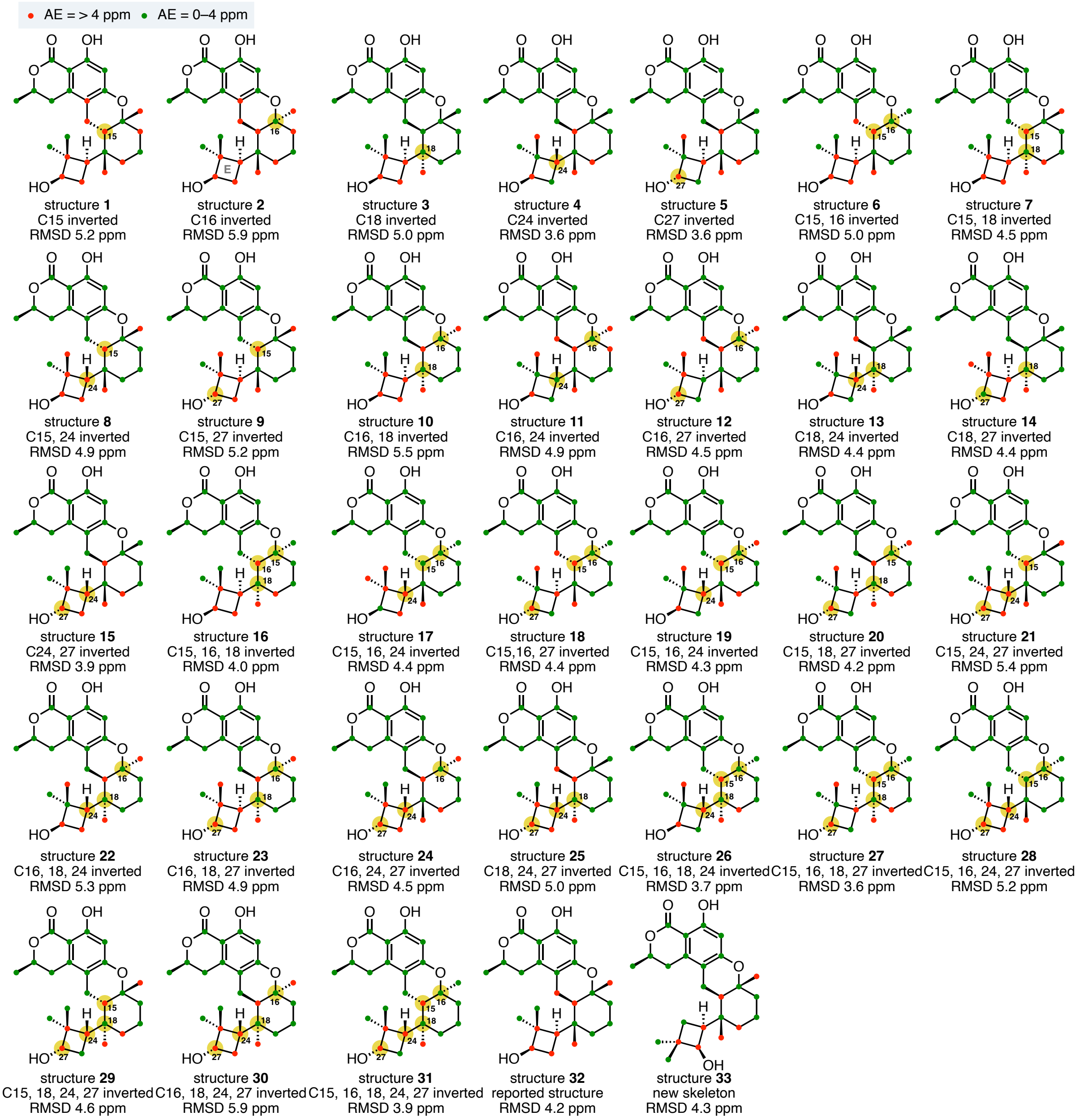

Note: AE = Absolute Error. RMSD = Root Mean Square Deviation. Carbons with inverted stereochemistry are highlighted in yellow. ^{13}C NMR calculations were obtained via protocol reported by Hehre.[7] The calculations were performed using the Spartan'24 software. The default calculation parameters are presented in **Table S4**. The calculated data are presented in **Tables S5-S13**. Structure **33** appeared in a review article as talaromyolide D published by the isolation group.[8]

Table S4. Default parameters for ^{13}C NMR calculations.

Step	Theory		Level	Keep ≤	Conformers at most
Geometry	Molecular Mechanics		MMFF	40 kJ/mol	200
Energy	Molecular Mechanics		Corrected MMFF	20 kJ/mol	100
Geometry	Hartree-Fock		3-21G	30 kJ/mol	100
Energy	Density Functional	ωB97X-D	6-31G*	15 kJ/mol	50
Geometry	Density Functional	ωB97X-D	6-31G*	10 kJ/mol	50
Energy	Density Functional	ωB97X-V	6-311+G(2df,2p)[6-311G*]	10 kJ/mol	30
Energy	Density Functional	ωB97X-D	6-31G*	-	-

Table S5. Calculated ^{13}C NMR chemical shifts of structures **1-4** and corresponding deviations (AEs/RMSs) from reported values.

	Calculated chemical shifts, AEs, and RMSs							
Position	**1**	AE	**2**	AE	**3**	AE	**4**	AE
C1	103.1	1.5	103.1	1.5	103.3	1.7	103.0	1.4
C2	139.3	0.3	139.1	0.1	139.5	0.5	137.7	1.3
C3	106.2	4.7	106.3	4.6	108.9	2.0	109.2	1.7
C4	159.0	1.5	158.9	1.6	160.3	0.2	160.4	0.1
C5	102.6	0.7	102.5	0.8	103.8	0.5	103.4	0.1
C6	164.5	2.3	164.6	2.4	164.6	2.4	164.4	2.2
C7	171.8	1.7	171.8	1.7	171.7	1.6	170.1	0.0
C9	73.0	1.6	73.1	1.5	73.2	1.4	72.8	1.8
C10	32.0	0.6	31.2	0.2	31.4	0.0	29.7	1.7
C12	21.1	0.2	21.0	0.1	21.0	0.1	18.9	2.0
C14	23.6	4.3	23.8	4.5	22.7	3.4	22.2	2.9
C15	44.4	7.2	41.3	10.3	51.4	0.2	42.7	8.9
C16	78.9	1.0	79.4	1.5	77.1	0.8	77.8	0.1
C18	39.2	2.5	39.7	3.0	37.7	1.0	37.4	0.7
C19	31.2	6.3	33.3	4.2	37.9	0.4	32.8	4.7
C20	20.0	0.58	20.3	0.88	19.8	0.38	19.8	0.38
C21	30.8	9.9	33.0	7.7	38.7	2.0	39.3	1.4
C22	29.9	9.1	32.3	11.5	21.4	0.6	20.8	0.0
C23	24.0	9.1	25.7	10.8	26.8	11.9	20.9	6.0
C24	43.8	11.2	40.0	15.0	37.9	17.1	47.5	7.5
C25	44.7	5.9	44.7	5.9	45.8	7.0	42.0	3.2
C26	31.7	4.59	32.5	5.39	34.3	7.19	26.4	0.71
C27	70.9	7.7	70.7	7.9	71.6	7.0	74.1	4.5
C28	17.8	2.3	18.9	3.4	17.8	2.3	22.8	7.3
C29	30.9	2.8	31.7	3.6	30.3	2.2	24.3	3.8
RMSD		5.2		5.9		5.0		3.6

Table S6. Calculated ^{13}C NMR chemical shifts of structures **5-8** and corresponding deviations (AEs/RMSs) from reported values.

	Calculated chemical shifts, AEs, and RMSs							
Position	**5**	AE	**6**	AE	**7**	AE	**8**	AE
C1	103.4	1.8	103.3	1.7	103.7	2.1	103.8	2.2
C2	139.9	0.9	139.5	0.5	139.1	0.1	141	2.0
C3	108.7	2.2	108.5	2.4	108.6	2.3	109	1.9
C4	160.3	0.2	160.1	0.4	161.7	1.2	161.7	1.2
C5	103.8	0.5	103.6	0.3	104.1	0.8	102.7	0.6
C6	164.6	2.4	164.6	2.4	164.6	2.4	164.7	2.5
C7	171.8	1.7	171.7	1.6	172.0	1.9	171.7	1.6
C9	73.1	1.5	73.1	1.5	73.2	1.4	73.5	1.1
C10	31.4	0.0	32.1	0.7	31.7	0.3	31.6	0.2
C12	21.0	0.1	21.0	0.1	21.0	0.1	20.8	0.1
C14	21.2	1.9	23.6	4.3	22.5	3.2	22	2.7
C15	42.8	8.8	47.5	4.1	39.9	11.7	45.4	6.2
C16	76.9	1.0	76.9	1.0	76.3	1.6	76.7	1.2
C18	38.4	1.7	39.0	2.3	37.2	0.5	38.3	1.6
C19	33.4	4.1	39.6	2.1	33.5	4.0	36.8	0.7
C20	19.6	0.18	18.8	0.62	18.4	1.02	18.4	1.02
C21	38.2	2.5	38.8	1.9	38.6	2.1	39	1.7
C22	20.7	0.1	23.0	2.2	27.0	6.2	26.7	5.9
C23	21.0	6.1	26.1	11.2	22.2	7.3	26.9	12.0
C24	48.0	7.0	38.8	16.2	44.9	10.1	40.7	14.3
C25	42.0	3.2	45.0	6.2	44.8	6.0	44.5	5.7
C26	25.2	1.91	32.5	5.39	31.5	4.39	32.8	5.69
C27	73.9	4.7	70.1	8.5	71.0	7.6	74.4	4.2
C28	23.7	8.2	18.4	2.9	17.1	1.6	23.2	7.7
C29	24.6	3.5	31.0	2.9	30.0	1.9	25.9	2.2
RMSD		3.6		5.0		4.5		4.9

Table S7. Calculated ^{13}C NMR chemical shifts of structures **9-12** and corresponding deviations (AEs/RMSs) from reported values.

	Calculated chemical shifts, AEs, and RMSs							
Position	**9**	AE	**10**	AE	**11**	AE	**12**	AE
C1	103.6	2.0	103.5	1.9	103.2	1.6	103.5	1.9
C2	141.0	2.0	139.4	0.4	139.3	0.3	139.2	0.2
C3	109.2	1.7	107.3	3.6	107.3	3.6	108.1	2.8
C4	162.0	1.5	159.7	0.8	160.7	0.2	161.1	0.6
C5	103.5	0.2	103.3	0.0	103.5	0.2	103.6	0.3
C6	163.5	1.3	164.5	2.3	164.6	2.4	164.4	2.2
C7	172.0	1.9	171.5	1.4	171.8	1.7	171.5	1.4
C9	73.7	0.9	72.9	1.7	73.0	1.6	73.1	1.5
C10	32.2	0.8	31.8	0.4	32.1	0.7	31.7	0.3
C12	20.8	0.1	21.0	0.1	21.0	0.1	21.1	0.2
C14	22.4	3.1	20.4	1.1	23.8	4.5	24.2	4.9
C15	46.0	5.6	42.8	8.8	35.4	16.2	37.0	14.6
C16	75.6	2.3	77.3	0.6	78.0	0.1	76.4	1.5
C18	37.4	0.7	36.7	0.0	36.6	0.1	38.5	1.8
C19	35.0	2.5	35.0	2.5	37.2	0.3	37.5	0.0
C20	19.0	0.42	18.4	1.02	17.9	1.52	17.7	1.72
C21	38.3	2.4	37.3	3.4	33.3	7.4	39.6	1.1
C22	29.2	8.4	27.2	6.4	27.7	6.9	26.9	6.1
C23	31.1	16.2	28.7	13.8	25.0	10.1	23.7	8.8
C24	44.6	10.4	40.5	14.5	52.9	2.1	51.5	3.5
C25	43.6	4.8	45.6	6.8	42.7	3.9	43.4	4.6
C26	34.2	7.09	34.3	7.19	30.3	3.19	30.1	2.99
C27	73.2	5.4	70.1	8.5	74.4	4.2	74.1	4.5
C28	21.6	6.1	17.0	1.5	21.3	5.8	21.9	6.4
C29	25.5	2.6	30.5	2.4	24.4	3.7	24.4	3.7
RMSD		5.2		5.5		4.9		4.5

Table S8. Calculated ^{13}C NMR chemical shifts of structures **13-16** and corresponding deviations (AEs/RMSs) from reported values.

	Calculated chemical shifts, AEs, and RMSs							
Position	**13**	AE	**14**	AE	**15**	AE	**16**	AE
C1	103.3	1.7	103.3	1.7	103.2	1.6	103.2	1.6
C2	139.4	0.4	139.5	0.5	139.5	0.5	139.8	0.8
C3	108.8	2.1	109.1	1.8	108.3	2.6	108.3	2.6
C4	160.6	0.1	160.5	0.0	160.4	0.1	160.3	0.2
C5	104	0.7	103.9	0.6	103.8	0.5	103.5	0.2
C6	164.6	2.4	164.6	2.4	164.8	2.6	164.8	2.6
C7	171.7	1.6	171.7	1.6	171.7	1.6	171.9	1.8
C9	73.1	1.5	73.1	1.5	73.2	1.4	73.1	1.5
C10	31.5	0.1	31.4	0.0	31.6	0.2	32.0	0.6
C12	21	0.1	21.0	0.1	21.0	0.1	21.0	0.1
C14	23.8	4.5	22.6	3.3	21.9	2.6	22.2	2.9
C15	47.8	3.8	51.6	0.0	43.8	7.8	43.3	8.3
C16	77.4	0.5	77.3	0.6	77.9	0.0	77.9	0.0
C18	39.2	2.5	37.7	1.0	37.2	0.5	37.4	0.7
C19	39	1.5	37.4	0.1	33.4	4.1	33.1	4.4
C20	18.9	0.52	19.9	0.48	19.8	0.38	19.8	0.38
C21	38.8	1.9	38.7	2.0	39.2	1.5	39.1	1.6
C22	22.7	1.9	21.5	0.7	21.0	0.2	20.7	0.1
C23	26.1	11.2	26.6	11.7	21.5	6.6	21.5	6.6
C24	42.1	12.9	41.1	13.9	44.5	10.5	44.1	10.9
C25	42.5	3.7	43.1	4.3	44.8	6.0	44.9	6.1
C26	28.1	0.99	31.3	4.19	31.3	4.19	31.3	4.19
C27	73.3	5.3	75.2	3.4	70.8	7.8	70.7	7.9
C28	24.7	9.2	23.1	7.6	16.7	1.2	16.8	1.3
C29	25.9	2.2	24.1	4.0	29.8	1.7	29.6	1.5
RMSD		4.4		4.4		3.9		4.0

Table S9. Calculated ^{13}C NMR chemical shifts of structures **17-20** and corresponding deviations (AEs/RMSs) from reported values.

	Calculated chemical shifts, AEs, and RMSs							
Position	**17**	AE	**18**	AE	**19**	AE	**20**	AE
C1	103.1	1.5	103.1	1.5	103.6	2.0	103.7	2.1
C2	139.5	0.5	139.4	0.4	139.1	0.1	139.2	0.2
C3	108.9	2.0	108.7	2.2	108.7	2.2	108.8	2.1
C4	160.2	0.3	160.3	0.2	161.8	1.3	161.8	1.3
C5	103.5	0.2	103.7	0.4	103.9	0.6	104.1	0.8
C6	164.5	2.3	164.5	2.3	164.6	2.4	164.6	2.4
C7	171.7	1.6	171.6	1.5	171.9	1.8	171.9	1.8
C9	73.2	1.4	73.1	1.5	73.4	1.2	73.4	1.2
C10	32.0	0.6	32.2	0.8	31.5	0.1	31.7	0.3
C12	21.0	0.1	21.0	0.1	21.0	0.1	21.0	0.1
C14	22.6	3.3	23.9	4.6	21.3	2.0	22.5	3.2
C15	51.5	0.1	47.5	4.1	38.8	12.8	39.2	12.4
C16	77.3	0.6	77.2	0.7	76.7	1.2	76.4	1.5
C18	37.7	1.0	39.2	2.5	38.5	1.8	37.4	0.7
C19	37.5	0.0	39.1	1.6	33.0	4.5	33.2	4.3
C20	19.9	0.48	18.8	0.62	17.8	1.62	18.4	1.02
C21	39.0	1.7	39.0	1.7	38.4	2.3	38.7	2.0
C22	21.3	0.5	22.8	2.0	27.2	6.4	27.0	6.2
C23	26.6	11.7	25.7	10.8	21.4	6.5	21.8	6.9
C24	41.2	13.8	41.8	13.2	47.6	7.4	48.1	6.9
C25	43.1	4.3	42.2	3.4	41.9	3.1	41.8	3.0
C26	31.3	4.19	27.2	0.09	25.5	1.61	26.4	0.71
C27	75.3	3.3	73.4	5.2	73.8	4.8	74.4	4.2
C28	23.1	7.6	24.5	9.0	23.4	7.9	22.9	7.4
C29	24.1	4.0	25.8	2.3	24.2	3.9	24.3	3.8
RMSD		4.4		4.4		4.3		4.2

Table S10. Calculated ^{13}C NMR chemical shifts of structures **21-24** and corresponding deviations (AEs/RMSs) from reported values.

	Calculated chemical shifts, AEs, and RMSs							
Position	**21**	AE	**22**	AE	**23**	AE	**24**	AE
C1	103.8	2.2	103.4	1.8	103.0	1.4	103.5	1.9
C2	140.1	1.1	139.7	0.7	139.6	0.6	139.2	0.2
C3	109.3	1.6	107.2	3.7	109.6	1.3	108.3	2.6
C4	161.5	1.0	160.0	0.5	161.9	1.4	161.6	1.1
C5	104.0	0.7	103.0	0.3	104.4	1.1	103.7	0.4
C6	164.4	2.2	164.1	1.9	164.2	2.0	164.6	2.4
C7	171.9	1.8	171.8	1.7	171.6	1.5	171.9	1.8
C9	73.5	1.1	73.3	1.3	73.1	1.5	73.2	1.4
C10	31.6	0.2	32.0	0.6	32.0	0.6	31.9	0.5
C12	21.0	0.1	21.0	0.1	21.0	0.1	21.0	0.1
C14	22.1	2.8	21.5	2.2	22.3	3.0	22.8	3.5
C15	45.8	5.8	43.0	8.6	46.5	5.1	40.2	11.4
C16	76.5	1.4	77.0	0.9	76.5	1.4	76.3	1.6
C18	38.4	1.7	37.2	0.5	38.2	1.5	37.2	0.5
C19	37.5	0.0	33.9	3.6	37.1	0.4	33.5	4.0
C20	18.4	1.02	18.6	0.82	18.4	1.02	18.4	1.02
C21	39.0	1.7	39.5	1.2	39.0	1.7	38.6	2.1
C22	26.7	5.9	28.8	8.0	26.6	5.8	26.8	6.0
C23	27.3	12.4	31.7	16.8	26.9	12.0	22.2	7.3
C24	36.4	18.6	45.0	10.0	40.5	14.5	44.8	10.2
C25	44.8	6.0	42.1	3.3	43.2	4.4	44.7	5.9
C26	31.3	4.19	32.5	5.39	32.5	5.39	31.5	4.39
C27	71.3	7.3	72.3	6.3	73.4	5.2	71.0	7.6
C28	18.8	3.3	22.4	6.9	23.5	8.0	17.1	1.6
C29	29.9	1.8	24.9	3.2	24.9	3.2	29.9	1.8
RMSD		5.4		5.3		4.9		4.5

Table S11. Calculated ^{13}C NMR chemical shifts of structures **25-28** and corresponding deviations (AEs/RMSs) from reported values.

	Calculated chemical shifts, AEs, and RMSs							
Position	**25**	AE	**26**	AE	**27**	AE	**28**	AE
C1	103.4	1.8	103.3	1.7	103.1	1.5	103.3	1.7
C2	139.4	0.4	140.0	1.0	139.8	0.8	139.5	0.5
C3	108.6	2.3	108.5	2.4	108.4	2.5	108.6	2.3
C4	160.4	0.1	160.1	0.4	160.3	0.2	160.0	0.5
C5	104.0	0.7	103.5	0.2	103.5	0.2	103.5	0.2
C6	164.6	2.4	164.5	2.3	164.8	2.6	164.6	2.4
C7	171.7	1.6	171.7	1.6	171.7	1.6	171.8	1.7
C9	73.2	1.4	73.2	1.4	73.2	1.4	73.2	1.4
C10	31.5	0.1	31.8	0.4	32.1	0.7	32.0	0.6
C12	21.0	0.1	21.1	0.2	21.1	0.2	21.0	0.1
C14	23.5	4.2	21.3	2.0	22.2	2.9	22.7	3.4
C15	47.6	4.0	42.6	9.0	42.8	8.8	51.4	0.2
C16	77.0	0.9	76.8	1.1	77.8	0.1	77.0	0.9
C18	39.0	2.3	38.3	1.6	37.4	0.7	37.6	0.9
C19	39.5	2.0	33.4	4.1	32.9	4.6	38.1	0.6
C20	18.8	0.62	19.5	0.08	19.8	0.38	19.8	0.38
C21	38.6	2.1	38.4	2.3	39.3	1.4	38.8	1.9
C22	22.9	2.1	20.6	0.2	20.8	0.0	21.4	0.6
C23	26.1	11.2	20.9	6.0	20.9	6.0	26.8	11.9
C24	38.8	16.2	47.9	7.1	47.6	7.4	37.8	17.2
C25	45.0	6.2	41.9	3.1	42.0	3.2	46.5	7.7
C26	32.5	5.39	25.2	1.91	26.5	0.61	35.9	8.79
C27	70.1	8.5	73.7	4.9	74.2	4.4	71.6	7.0
C28	18.4	2.9	23.8	8.3	22.8	7.3	16.9	1.4
C29	30.9	2.8	24.6	3.5	24.2	3.9	29.9	1.8
RMSD		5.0		3.7		3.6		5.2

Table S12. Calculated ^{13}C NMR chemical shifts of structures **29-32** and corresponding deviations (AEs/RMSs) from reported values.

	Calculated chemical shifts, AEs, and RMSs							
Position	**29**	AE	**30**	AE	**31**	AE	**32**	AE
C1	103.7	2.1	103.6	2.0	103.3	1.7	103.2	1.6
C2	139.0	0.0	138.9	0.1	139.8	0.8	139.1	0.1
C3	108.3	2.6	108.3	2.6	108.3	2.6	107.9	3.0
C4	161.7	1.2	161.6	1.1	160.0	0.5	160.1	0.4
C5	104.0	0.7	104.2	0.9	103.5	0.2	103.6	0.3
C6	164.7	2.5	164.6	2.4	164.6	2.4	164.7	2.5
C7	171.8	1.7	171.6	1.5	171.7	1.6	171.7	1.6
C9	73.2	1.4	73.1	1.5	73.1	1.5	73.1	1.5
C10	31.6	0.2	32.1	0.7	31.9	0.5	31.7	0.3
C12	21.0	0.1	21.0	0.1	21.1	0.2	21.0	0.1
C14	21.4	2.1	22.9	3.6	21.4	2.1	23.9	4.6
C15	39.5	12.1	47.1	4.5	43.0	8.6	41.4	10.2
C16	76.5	1.4	75.2	2.7	76.9	1.0	78.3	0.4
C18	38.3	1.6	37.4	0.7	38.2	1.5	36.4	0.3
C19	33.3	4.2	35.3	2.2	33.8	3.7	34.5	3.0
C20	17.7	1.72	18.9	0.52	19.6	0.18	17.4	2.02
C21	38.3	2.4	38.0	2.7	38.3	2.4	38.0	2.7
C22	27.1	6.3	27.1	6.3	20.5	0.3	26.1	5.3
C23	21.7	6.8	31.2	16.3	21.5	6.6	21.3	6.4
C24	44.4	10.6	38.6	16.4	45.1	9.9	46.8	8.2
C25	44.7	5.9	45.5	6.7	44.8	6.0	44.7	5.9
C26	30.4	3.29	36.8	9.69	30.3	3.19	32.1	4.99
C27	70.7	7.9	70.2	8.4	70.6	8.0	70.6	8.0
C28	17.2	1.7	16.2	0.7	17.7	2.2	18.3	2.8
C29	30.3	2.2	29.5	1.4	30.5	2.4	28.3	0.2
RMSD		4.6		5.9		3.9		4.2

Table S13. Calculated ^{13}C NMR chemical shifts of structure **33** and corresponding deviations (AEs/RMSs) from reported values.

	Calculated chemical shifts, AEs, and RMSs	
Position	**33**	AE
C1	103.7	2.1
C2	139.0	0.0
C3	108.3	2.6
C4	161.7	1.2
C5	104.0	0.7
C6	164.7	2.5
C7	171.8	1.7
C9	73.2	1.4
C10	31.6	0.2
C12	21.0	0.1
C14	21.4	2.1
C15	39.5	12.1
C16	76.5	1.4
C18	38.3	1.6
C19	33.3	4.2
C20	17.7	1.72
C21	38.3	2.4
C22	27.1	6.3
C23	21.7	6.8
C24	44.4	10.6
C25	44.7	5.9
C26	30.4	3.29
C27	70.7	7.9
C28	17.2	1.7
C29	30.3	2.2
RMSD		4.3

General Information.

Unless otherwise stated, all reagents were purchased from commercial suppliers and used without further purification. Unless otherwise noted, all reactions were performed with anhydrous solvents and under a positive pressure of nitrogen. Anhydrous solvents were obtained from the solvent purification system produced by Innovative Technology company. Analytical thin-layer chromatography (TLC) was performed on Merck Millipore precoated (0.25 mm thickness) silica gel plates with F254 fluorescent indicator. TLC plates were visualized by exposure to ultraviolet light (UV) and/or submersion in aqueous potassium permanganate solution ($KMnO_4$), ceric ammonium molybdate solution (CAM), *para*-anisaldehyde (PAA) and followed by brief heating on a hot plate (120 °C, 10–15 s). Flash-column chromatography was performed employing silica gel (32-63 μm particle size) supplied by Dynamic Adsorbents. Proton nuclear magnetic resonance spectra (^{1}H NMR) were recorded on a Bruker instrument (400, 500 or 600 MHz). Chemical shifts are expressed in parts per million (ppm, d scale) downfield from tetramethylsilane and are referenced to residual protium in the NMR solvent ($CHCl_3$, δ 7.26; CD_2HSCD_3, δ 2.50). Data are represented as follows: chemical shift, multiplicity (s = singlet, d = doublet, t = triplet, q = quartet, m = multiplet and/or multiple resonances, br. = broad), coupling constant, *J*, in Hertz (Hz) and integration. Proton-decoupled carbon nuclear magnetic resonance spectra (^{13}C NMR) were recorded on a Bruker instrument (101, 126, or 151 MHz). Chemical shifts are expressed in parts per million (ppm, δ scale) downfield from tetramethylsilane and are referenced to the carbon resonances of the solvent ($CDCl_3$, δ 77.16; DMSO-*d6*, δ 39.52). High-resolution mass spectra (HRMS) were recorded on a Waters mass spectrometer using ESI-QTOF (electrospray ionization-quadrupole time of flight). Optical rotations were measured on a Jasco P-2000 polarimeter equipped with a sodium (589 nm, D) lamp. Optical rotation data are represented as follows: specific rotation ($[\alpha]_{\lambda}^{T}$), concentration (g/100 mL), and solvent.

Experimental procedures.

*Synthesis of the acid **S10**:*

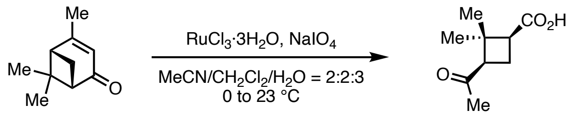

Ruthenium(III) chloride trihydrate (174.1 mg, 0.67 mmol, 0.02 equiv.) was added to a solution of (*S*)-verbenone (5.0 g, 33.3 mmol, 1.0 equiv.) in acetonitrile-dichloromethane-water (221 mL, *v*/*v*/*v* = 2:2:3). The flask was then placed in an ice bath and cooled to 0 °C. $NaIO_4$ (28.5 g, 133.1 mmol, 4.0 equiv.) was added in three portions within 30 min. The reaction was then allowed to warm gradually to 23 °C and stirred overnight. Upon complete conversion of (*S*)-verbenone, the mixture was diluted with water (300 mL), transferred to a separatory funnel, and extracted with diethyl ether (3 × 100 mL). The combined organic layers were washed with brine (100 mL) and dried over anhydrous sodium sulfate. The dried solution was filtered, and the filtrate was concentrated. The residue obtained was eluted over a short plug of silica gel with diethyl ether to provide the corresponding carboxylic acid **S10** as a yellow crystalline solid. The crude product was used directly in the next reaction without further purification.

R_f = 0.3 (5% methanol-dichloromethane, CAM stain).
^{1}H NMR (400 MHz, $CDCl_3$): δ 2.90 (dd, *J* = 10.6, 7.7 Hz, 1H), 2.82 (dd, *J* = 10.8, 8.0 Hz, 1H), 2.61 (dt, *J* = 11.7, 10.7 Hz, 1H), 2.07 (s, 3H), 1.90 (dt, *J* = 11.8, 7.8 Hz, 1H), 1.45 (s, 3H), 0.97 (s, 3H).
^{13}C NMR (101 MHz, $CDCl_3$): δ 207.1, 178.1 , 53.1 , 45.13 , 45.08 , 30.4 , 30.1 , 18.9 , 18.2.
$[\alpha]_D^{23}$ = − 43.9 (*c* 1.0, $CHCl_3$).
HRMS-QTOF (m/z): $[M + Na]^+$ calculated for $C_9H_{14}NaO_3^+$, 193.0835; found 193.0827.

*Synthesis of the acid **S11**:*

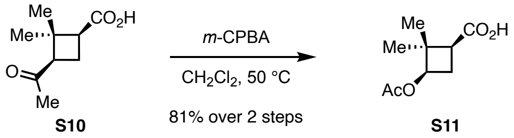

meta-Chloroperoxybenzoic acid (23.0 g, 75 wt.%, 99.9 mmol, 1.5 equiv.) was added to a solution of crude acid **S10** (previous step carried out twice in total) in dichloromethane (220 mL). The flask was placed in an oil bath preheated to 50 °C and stirred for 24 h. The flask was removed from the oil bath and cooled to 23 °C. An additional portion of *meta*-chloroperoxybenzoic acid (7.7 g, 75 wt.%, 33.3 mmol, 0.5 equiv.) was added. The reaction mixture was stirred for an additional 12 hours at 50 °C. Upon complete conversion of acid **S10** (as monitored by NMR), the flask was placed in an ice bath and cooled to 0 °C. The resulting white precipitate was filtered off, and the filtrate was concentrated. The residue obtained was purified by flash column chromatography (eluting with dichloromethane; then 10-20% ethyl acetate-hexanes with 1% acetic acid) to provide acid **S11** as a pale-yellow oil (10.0 g, 81% over 2 steps).

R_f = 0.4 (5% methanol-dichloromethane, CAM stain).
^{1}H NMR (400 MHz, $CDCl_3$): δ 4.71 (t, *J* = 8.2 Hz, 1H), 2.55 (dd, *J* = 10.5, 7.8 Hz, 1H), 2.40 - 2.30 (m, 2H), 2.05 (s, 3H), 1.31 (s, 3H), 1.03 (s, 3H).
^{13}C NMR (101 MHz, $CDCl_3$): 178.1, 170.9, 72.6, 46.8, 41.6, 28.4, 26.2, 20.8, 16.8.
$[\alpha]_D^{23}$ = + 47.0 (*c* 1.0, $CHCl_3$).
HRMS-QTOF (m/z): $[M + Na]^+$ calculated for $C_9H_{14}NaO_4^+$, 209.0784; found 209.0777.

*Synthesis of the NHPI ester **16**:*

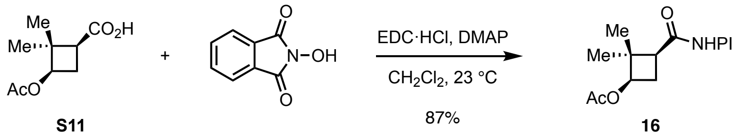

N-Hydroxyphthalimide (14.7 g, 90.2 mmol, 1.5 equiv.), 4-dimethylaminopyridine (1.5 g, 12.0 mmol, 0.2 equiv.), and 1-ethyl-3-(3-dimethylaminopropyl)carbodiimide hydrochloride (23.1 g, 120.3 mmol, 2.0 equiv.) were sequentially added to a solution of acid **S11** (11.2 g, 60.1 mmol, 1.0 equiv.) in dichloromethane (300 ml). The flask was then covered with foil and stirred at 23 °C for 48 h. Upon complete conversion of **S11**, the reaction mixture was transferred to a separatory funnel and washed sequentially with water (3 × 100 mL), 1 M sodium hydroxide (100 mL), and brine (100 mL). The organic layer was dried over anhydrous sodium sulfate. The dried solution was filtered, and the filtrate was concentrated. The residue obtained was purified by flash column chromatography (eluting with 10-20% ethyl acetate-hexanes) to provide ester **16** as a pale-yellow oil (18.2 g, 95% purity, 87%).

R_f = 0.5 (30% ethyl acetate-hexanes, UV & CAM stain).
^{1}H NMR (400 MHz, $CDCl_3$): δ 7.92 - 7.84 (m, 2H), 7.83 - 7.74 (m, 2H), 4.80 (t, *J* = 8.2 Hz, 1H), 2.89 (dd, *J* = 10.4, 7.8 Hz, 1H), 2.57 - 2.39 (m, 2H), 2.06 (s, 3H), 1.43 (s, 3H), 1.20 (s, 3H).
^{13}C NMR (101 MHz, $CDCl_3$): δ 170.7, 168.4, 162.0, 134.9, 129.1, 124.1, 72.5, 47.7, 39.3, 28.3, 26.3, 20.8, 16.8.
$[\alpha]_D^{23}$ = + 81.7 (*c* 1.0, $CHCl_3$).
HRMS-QTOF (m/z): $[M + Na]^+$ calculated for $C_{17}H_{17}NNaO_6^+$, 354.0948; found 354.0947.

*Synthesis of the vinyl iodide **17**:*

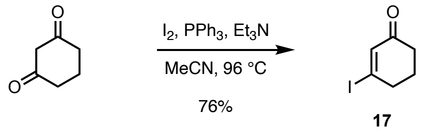

17

Fine powered iodine (24.9 g, 98.1 mmol, 1.1 equiv.) was added to a solution of triphenylphosphine (25.7 g, 98.1 mmol, 1.1 equiv.) in acetonitrile (400 mL) in three portions within 30 min. Triethylamine (13.7 mL, 98.1 mmol, 1.1 equiv.) and 1,3-cyclohexanedione (10.0 g, 89.2 mmol, 1.0 equiv.) were sequentially added. The flask was then equipped with a reflux condenser, placed in an oil bath preheated to 96 °C, and refluxed overnight. Upon complete conversion of 1,3-cyclohexanedione, the reaction mixture was concentrated under a stream of nitrogen. Diethyl ether (150 mL) was added to the residue. The white precipitation formed was filtered off and washed with diethyl ether (2 × 100.0 mL). The filtrate was washed sequentially with saturated aqueous sodium thiosulfate (100 mL) and brine (100 mL). The organic layer was dried over anhydrous sodium sulfate. The dried solution was filtered, and the filtrate was concentrated. The residue obtained was purified by flash column chromatography (eluting with 10% ethyl acetate-hexanes) to provide vinyl iodide **17** as a pale-yellow oil (15.0 g, 76%).

R_f = 0.7 (30% ethyl acetate-hexanes, UV & $KMnO_4$ stain).
^{1}H NMR (400 MHz, $CDCl_3$): δ 6.80 (s, 1H), 2.90 (td, *J* = 6.0, 1.7 Hz, 2H), 2.42 (t, *J* = 6.7 Hz, 2H), 2.02 (p, *J* = 6.3 Hz, 2H).
^{13}C NMR (101 MHz, $CDCl_3$): δ 195.3, 140.9, 127.1, 40.7, 36.7, 24.1.
HRMS-QTOF (m/z): $[M + H]^+$ calculated for $C_6H_8IO^+$, 222.9614; found 222.9613.

*Synthesis of the enone **20**:*

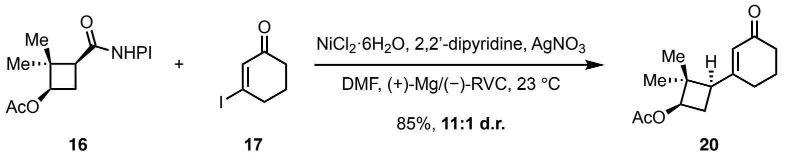

NHPI ester **16** (350.0 mg, 95% purity, 1.0 mmol, 1.0 equiv.), vinyl iodide **17** (334.2 mg, 1.5 mmol, 1.5 equiv.), nickel chloride hexahydrate (35.8 mg, 0.15 mmol, 0.15 equiv.), 2,2'-dipyridine (23.5 mg, 0.15 mmol, 0.15 equiv.), and silver nitrate (51.1 mg, 0.3 mmol, 0.3 equiv.) were added to a 10 mL ElectraSyn 2.0 vial. The vial was sealed with a 5 mL ElectraSyn 2.0 cap fitted with a magnesium sacrificial anode and a 100 ppi RVC cathode (6 mm × 6 mm × 40 mm). The vial was evacuated and backfilled with nitrogen three times. Degassed anhydrous DMF (9 mL) was added. The vial was then placed on an IKA ElectraSyn 2.0 stir plate. Electrolysis was set to 20 mA, 1.0 mmol substrate scale, and 3.1 F/mol. Upon completion of the programmed electrolysis, the reaction mixture was diluted with 1 M hydrochloric acid (30 mL), transferred to a separatory funnel, and extracted with diethyl ether (3 × 10 mL). The combined organic layers were washed successively with 1 M hydrochloric acid (20 mL), 1 M sodium hydroxide (20 mL), and brine (20 mL). The organic layer was dried over anhydrous sodium sulfate. The dried solution was filtered, and the filtrate was concentrated. The residue obtained was purified by flash column chromatography (eluting with 20% ethyl acetate-hexanes) to provide enone **20** as a colorless oil (202 mg, 85%).

Enone **20** and its C24-diastereomer were obtained as an inseparable mixture. ^{1}H NMR analysis of the purified product indicated a 11:1 ratio of diastereomers.

R_f = 0.5 (30% ethyl acetate-hexanes, UV & CAM stain).
^{1}H NMR (400 MHz, $CDCl_3$): δ 5.80 (s, 1H), 4.72 (t, *J* = 8.1 Hz, 1H), 2.46 - 2.35 (m, 3H), 2.34 - 2.26 (m, 1H), 2.22 - 2.18 (m, 2H), 2.13 - 2.06 (m, 1H), 2.04 (s, 3H), 2.01 - 1.95 (m, 2H), 1.32 (s, 3H), 0.82 (s, 3H).
^{13}C NMR (101 MHz, $CDCl_3$): δ 199.6, 170.9, 164.1, 125.4, 72.4, 47.2, 44.5, 37.8, 30.2, 29.2, 27.0, 22.7, 20.9, 16.3.
$[\alpha]_D^{23}$ = + 29.9 (*c* 1.0, $CHCl_3$).
HRMS-QTOF (m/z): $[M + Na]^+$ calculated for $C_{14}H_{20}NaO_3^+$, 259.1305; found 259.1301.

Note: The reaction carried out using the original reported condition (substrate scale: 1 mmol, current: 30 mA, change:2.3 F/mol, 10 mol% loading of catalyst)[9] gave product yield of 47%.

*Synthesis of the alcohol **21**:*

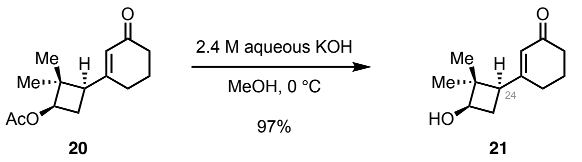

Enone **20** (24.0 mg, 0.10 mmol, 1.0 equiv.) was dissolved in methanol (0.4 mL). The flask was placed in an ice bath and cooled to 0 °C. Potassium hydroxide (51 μL, 2.4 M aqueous solution, 0.12 mmol, 1.2 equiv.) was added dropwise. The reaction was stirred at 0 °C for 1 h. Upon complete conversion of enone **20**, the reaction mixture was diluted with saturated aqueous ammonium chloride (10 mL), transferred to a separatory funnel, and extracted with ethyl acetate (3 × 5 mL). The combined organic layers were washed with brine (10 mL) and dried over anhydrous sodium sulfate. The dried solution was filtered, and the filtrate was concentrated. The residue obtained was purified by flash column chromatography (eluting with 40% ethyl acetate-hexanes) to provide alcohol **21** as a white crystalline solid (19.1 mg, 97%).

Alcohol **21** and its C24-diastereomer were obtained as an inseparable mixture. ^{1}H NMR analysis of the purified product indicated a 11:1 ratio of diastereomers.

R_f = 0.25 (50% ethyl acetate-hexanes, UV & CAM stain).
^{1}H NMR (400 MHz, $CDCl_3$): δ 5.78 (s, 1H), 3.87 (dd, *J* = 8.4, 6.9 Hz, 1H), 2.37 (td, *J* = 6.4, 2.5 Hz, 2H), 2.32 – 2.23 (m, 2H), 2.22 – 2.15 (m, 2H), 2.01 – 1.93 (m, 3H), 1.91 – 1.80 (m, 1H), 1.24 (s, 3H), 0.84 (s, 3H).
^{13}C NMR (101 MHz, $CDCl_3$): δ 199.8, 165.1, 125.1, 71.3, 47.7, 44.1, 37.7, 30.5, 30.3, 28.9, 22.7, 15.3.
$[\alpha]_D^{23}$ = −10.6, (*c* 1.0, $CHCl_3$).
HRMS-QTOF (m/z): $[M + H]^+$ calculated for $C_{12}H_{19}O_2^+$, 195.1380; found 195.1369.

*Synthesis of the enone **23**:*

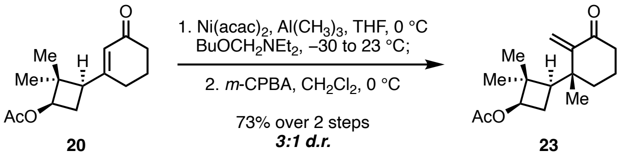

Nickel(II) bis(acetylacetonate) (108.7 mg, 0.4 mmol, 0.05 equiv.) was added to a solution of enone **20** (2.0 g, 8.5 mmol, 1.0 equiv.) in dry THF (53 mL). The flask was placed in an ice bath and cooled to 0 °C. Trimethylaluminum (8.5 mL, 2 M in heptane, 16.9 mmol, 2.0 equiv.) was added dropwise. The reaction mixture was stirred at 0 ℃ for 2 h. Upon complete conversion of enone **20**, the reaction was cooled to −30 °C. *n*-$BuOCH_2NEt_2$ (5.3 mL, 25.4 mmol, 3.0 equiv.) was added dropwise. The reaction was then stirred for 10 min at −30 °C, 10 min at 0 °C, and 6 h at 23 °C. Upon complete conversion of the 1,4-addition intermediate (as monitored by TLC), the reaction was cooled to 0 °C and diluted with saturated aqueous Rochelle salt (200 mL). The mixture was transferred to a separatory funnel and extracted with ethyl acetate (3 × 40 mL). The combined organic layers were washed with brine and dried over anhydrous sodium sulfate. The dried solution was filtered, and the filtrate was concentrated. The crude amine intermediates obtained were directly used in the next reaction without further purification.

The crude amine products were dissolved in dichloromethane (116 mL). The flask was placed in an ice bath and cooled to 0 °C. *meta*-Chloroperoxybenzoic acid (2.6 g, 80 wt.%, 11.9 mmol, 1.4 equiv.) was added. The reaction was stirred at 0 °C for 30 min. Upon complete conversion of the amine intermediates, the reaction mixture was transferred to a separatory funnel immediately, washed sequentially with 1 M sodium hydroxide (3 × 50 mL) and brine (50 mL). The organic layer was dried over anhydrous sodium sulfate. The dried solution was filtered, and the filtrate was concentrated. The residue obtained was purified by flash column chromatography (eluting with 8% ethyl acetate-hexanes) to provide enone **23** (1.2 g, 55% over 2 steps) as a colorless oil.

R_f = 0.5 (20% ethyl acetate-hexanes, UV & CAM stain).
^{1}H NMR (400 MHz, $CDCl_3$): δ 5.77 (d, *J* = 1.2 Hz, 1H), 5.19 (d, *J* = 1.3 Hz, 1H), 4.50 (dd, *J* = 8.3, 7.1 Hz, 1H), 2.52 – 2.27 (m, 2H), 2.18 (dt, *J* = 10.1, 7.1 Hz, 1H), 2.01 (s, 3H), 1.95 – 1.81 (m, 3H), 1.77 (dd, *J* = 12.3, 7.1 Hz, 1H), 1.72 – 1.63 (m, 1H), 1.59 – 1.51 (m, 1H), 1.21 (s, 3H), 1.12 (s, 3H), 0.96 (s, 3H).
^{13}C NMR (101 MHz, $CDCl_3$): δ 204.3, 171.0, 154.1, 118.8, 73.4, 46.6, 44.3, 42.4, 40.4, 36.3, 30.2, 27.5, 24.9, 21.0, 19.3, 17.4.
$[\alpha]_D^{24}$ = + 99.4, (*c* 0.3, $CHCl_3$).
HRMS-QTOF (m/z): $[M + H]^+$ calculated for $C_{16}H_{25}O_3^+$, 265.1798; found 265.1791.

*Synthesis of the ketone **S12**:*

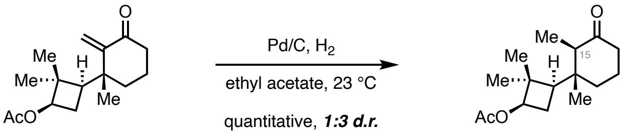

23 **S12**

Palladium on carbon (495.1 mg, 10 wt.% on carbon, 0.47 mmol, 0.1 equiv.) was added to a solution of α, β-unsaturated ketone **23** (1.2 g, 4.7 mmol, 1.0 equiv.) in ethyl acetate (23 mL). The reaction was then purged with hydrogen and stirred under a 1 atm hydrogen atmosphere for 4 h. Upon complete conversion of **23**, the reaction mixture was filtered through a short pad of celite. The filtrate was concentrated to provide enone **S12** and its C15-diastereomer as a colorless oil (quantitative). ^{1}H NMR analysis of the unpurified products indicated the presence of a 1:3 mixture of diastereomers at C15. The crude mixture was directly used in the epimerization reaction without further purification.

R_f = 0.4 (20% ethyl acetate-hexanes, CAM stain).
^{1}H NMR (400 MHz, $CDCl_3$): δ 4.53 (dd, J = 8.4, 7.1 Hz, 1H), 2.40 – 2.29 (m, 1H), 2.28 – 2.18 (m, 2H), 2.12 (dt, J = 10.3, 7.4 Hz, 1H), 2.04 (s, 3H), 1.99 – 1.90 (m, 2H), 1.88 – 1.81 (m, 1H), 1.80 – 1.68 (m, 3H), 1.21 (s, 3H), 1.07 (s, 3H), 0.94 (d, J = 6.8 Hz, 3H), 0.81 (s, 3H).
^{13}C NMR (101 MHz, $CDCl_3$): δ 213.6, 171.0, 73.3, 52.1, 46.6, 45.2, 43.9, 41.2, 32.8, 30.9, 26.5, 22.5, 21.0, 18.8, 18.2, 9.0.
$[\alpha]_D^{24}$ = – 16.9, (*c* 0.3, $CHCl_3$).
HRMS-QTOF (m/z): $[M + H]^+$ calculated for $C_{16}H_{26}NaO_3^+$, 289.1774; found 289.1772.

Synthesis of the alcohol ***24****:*

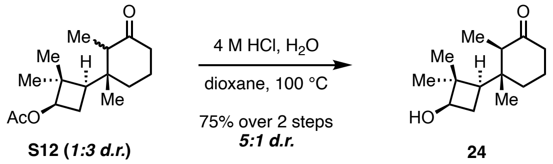

Water (0.42 mL, 23.3 mmol, 5.0 equiv.) and hydrochloric acid (17.4 mL, 4 M in 1,4-dioxane, 69.8 mmol, 15.0 equiv.) were sequentially added to a solution of ketone **S12** in 1,4-dioxane (17.4 mL). The flask was then equipped with a reflux condenser, placed in an oil bath preheated to 100 °C, and refluxed overnight. The reaction was cooled to 23 °C. The solvent was removed under a stream of nitrogen. The residue was diluted with saturated aqueous sodium bicarbonate (100 mL), transferred to a separatory funnel, and extracted with ethyl acetate (3 × 30 mL). The combined organic layers were washed with brine (50 mL) and dried over anhydrous sodium sulfate. The dried solution was filtered, and the filtrate was concentrated. The residue obtained was purified by flash column chromatography (eluting with 25% ethyl acetate-hexanes) to provide alcohol **24** as a colorless oil (782.0 mg, 63%).

R_f = 0.1 (20% ethyl acetate-hexanes, CAM stain).
^{1}H NMR (400 MHz, $CDCl_3$): δ 3.65 (t, *J* = 7.7 Hz, 1H), 2.39 – 2.29 (m, 1H), 2.29 – 2.16 (m, 2H), 2.09 (dt, *J* = 10.0, 7.1 Hz, 1H), 2.02 – 1.90 (m, 1H), 1.87 – 1.66 (m, 4H), 1.60 (dd, *J* = 12.5, 7.3 Hz, 1H), 1.13 (s, 3H), 1.08 (s, 3H), 0.94 (d, *J* = 6.8 Hz, 3H), 0.81 (s, 3H).
^{13}C NMR (101 MHz, $CDCl_3$): δ 214.0, 72.2, 52.2, 46.6, 44.9, 43.9, 41.1, 32.9, 30.6, 29.8, 22.5, 19.1, 17.4, 9.0.
$[\alpha]_D^{24}$ = – 34.4, (*c* 1.0, $CHCl_3$).
HRMS-QTOF (m/z): $[M + H]^+$ calculated for $C_{14}H_{25}NO_2^+$, 225.1849; found 225.1843.

*Synthesis of the nitroarene **25**:*

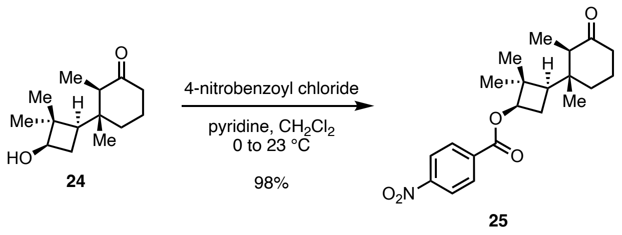

Pyridine (24.3 µL, 0.30 mmol, 10.0 equiv.) was added to a solution of alcohol **24** (6.8 mg, 0.03 mmol, 1.0 equiv.) in dichloromethane (1 mL). The reaction tube was placed in an ice bath and cooled to 0 °C. 4-Nitrobenzoyl chloride (27.9 mg, 0.15 mmol, 5.0 equiv.) was added. The reaction was allowed to warm gradually to 23 °C and stirred for 4 h. Upon complete conversion of alcohol **24**, the reaction mixture was diluted with saturated aqueous sodium bicarbonate (15 mL), transferred to a separatory funnel, and extracted with ethyl acetate (3 × 5 mL). The combined organic layers were washed with brine (5 mL) and dried over anhydrous sodium sulfate. The dried solution was filtered, and the filtrate was concentrated. The residue obtained was purified by flash column chromatography (eluting with 10% ethyl acetate-hexanes) to provide nitroarene **25** as a white crystalline solid (11.0 mg, 98%).

R_f = 0.4 (20% ethyl acetate-hexanes, UV & CAM stain).
^{1}H NMR (400 MHz, $CDCl_3$): δ 8.32 – 8.26 (m, 2H), 8.23 – 8.16 (m, 2H), 4.82 (t, *J* = 7.7 Hz, 1H), 2.44 – 2.33 (m, 1H), 2.33 – 2.21 (m, 3H), 2.19 – 2.07 (m, 1H), 2.04 – 1.95 (m, 1H), 1.92 – 1.74 (m, 4H), 1.31 (s, 3H), 1.20 (s, 3H), 0.99 (d, *J* = 6.7 Hz, 3H), 0.86 (s, 3H).
^{13}C NMR (101 MHz, $CDCl_3$): δ 213.4, 164.4, 150.7, 135.7, 130.8, 123.7, 74.8, 52.1, 47.0, 45.3, 43.9, 41.1, 32.9, 30.9, 26.7, 22.4, 18.9, 18.4, 9.1.
$[\alpha]_D^{24}$ = − 14.9, (*c* 1.0, $CHCl_3$).
HRMS-QTOF (m/z): $[M + H]^+$ calculated for $C_{21}H_{28}NO_5^+$, 374.1962; found 374.1965.

*Synthesis of the oxime **S13**:*

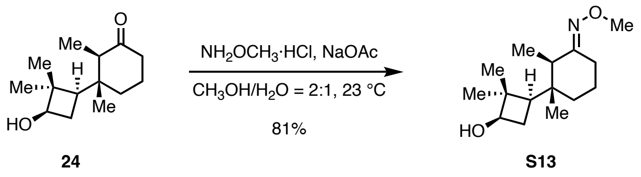

Sodium acetate (1.1 g, 13.4 mmol, 2.5 equiv.) and methoxyamine hydrochloride (893.5 mg, 10.7 mmol, 2.0 equiv.) were sequentially added to a solution of alcohol **24** (1.2 g, 5.3 mmol, 1.0 equiv.) in methanol-water (19.2 mL, *v*/*v* = 2:1). The reaction was then stirred at 23 °C overnight. Upon complete conversion of alcohol **24**, the mixture was diluted with water (50 mL), transferred to a separatory funnel, and extracted with ethyl acetate (3 × 15 mL). The combined organic layers were washed with brine (20 mL), dried over anhydrous sodium sulfate. The dried solution was filtered, and the filtrate was concentrated. The residue obtained was purified by flash column chromatography (eluting with 15% ethyl acetate-hexanes) to provide oxime **S13** (1.1 g, 81%) as a colorless oil.

R_f = 0.5 (30% ethyl acetate-hexanes, CAM stain).
^{1}H NMR (400 MHz, $CDCl_3$): δ 3.80 (s, 3H), 3.63 (dd, *J* = 8.3, 7.0 Hz, 1H), 2.51 (dt, *J* = 14.5, 5.5 Hz, 1H), 2.30 – 2.16 (m, 2H), 2.05 (dt, *J* = 9.9, 7.0 Hz, 1H), 1.72 (dd, *J* = 12.3, 7.1 Hz, 1H), 1.62 (dd, *J* = 9.9, 8.3 Hz, 1H), 1.55 – 1.43 (m, 3H), 1.33 – 1.23 (m, 1H), 1.18 (s, 3H), 1.06 – 0.98 (m, 6H), 0.88 (s, 3H).
^{13}C NMR (101 MHz, $CDCl_3$): δ 162.5, 72.4, 61.1, 46.6, 43.1, 42.3, 40.3, 32.2, 30.8, 30.0, 21.6, 20.9, 20.5, 17.4, 13.4.
$[\alpha]_D^{24}$ = − 14.9 (*c* 1.0, $CHCl_3$).
HRMS-QTOF (m/z): $[M + H]^+$ calculated for $C_{15}H_{28}NO_2^+$, 254.2115; found 254.2115.

*Synthesis of the oxime **26**:*

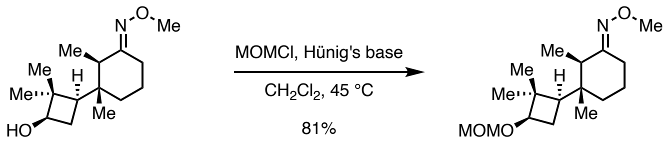

Oxime **S13** (1.1 g, 4.3 mmol, 1.0 equiv.) and Hünig's base (1.9 mL, 10.9 mmol, 2.5 equiv.) were dissolved in dichloromethane (22 mL). The flask was placed in an ice bath and cooled to 0 °C. Chloromethyl methyl ether (0.66 mL, 8.7 mmol, 2.0 equiv.) was added dropwise. The reaction was then placed in a heating block preheated to 45 °C and stirred overnight. Upon complete conversion of oxime **S13**, the reaction was cooled to 23 °C and diluted with dichloromethane (30 mL). The mixture was transferred to a separatory funnel and washed sequentially with saturated sodium bicarbonate (20 mL) and brine (20 mL). The organic layer was dried over anhydrous sodium sulfate. The dried solution was filtered, and the filtrate was concentrated. The residue obtained was purified by flash column chromatography (eluting with 5% ethyl acetate-hexanes) to afford oxime **26** as a colorless oil (1.1g, 81%).

R_f = 0.7 (20% ethyl acetate-hexanes, CAM stain).
^{1}H NMR (400 MHz, $CDCl_3$): δ 4.59 (d, *J* = 6.4 Hz, 1H), 4.57 (d, *J* = 6.5 Hz, 1H), 3.80 (s, 3H), 3.54 (t, *J* = 7.4 Hz, 1H), 3.35 (s, 3H), 2.48 (dt, *J* = 14.5, 5.5 Hz, 1H), 2.32 – 2.14 (m, 2H), 2.06 – 1.93 (m, 1H), 1.79 – 1.66 (m, 2H), 1.62 – 1.43 (m, 3H), 1.37 – 1.27 (m, 1H), 1.21 (s, 3H), 1.08 (s, 3H), 1.03 (d, *J* = 7.1 Hz, 3H), 0.89 (s, 3H).
^{13}C NMR (101 MHz, $CDCl_3$): δ 162.5, 95.8, 76.9, 61.1, 55.5, 46.6, 43.1, 42.3, 40.4, 32.3, 31.3, 27.6, 21.7, 20.7, 20.6, 18.3, 13.3.
$[\alpha]_D^{23}$ = − 7.2 (*c* 1.0, $CHCl_3$).
HRMS-QTOF (m/z): $[M + H]^+$ calculated for $C_{17}H_{32}NO_3^+$, 298.2377; found 298.2378.

Synthesis of the ester ***S14****:*

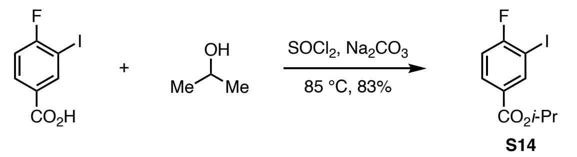

Thionyl chloride (2.0 mL, 27.1 mmol, 1.2 equiv.) was added dropwise to a suspension of 4-fluoro-3-iodobenzoic acid (6.0 g, 22.6 mmol, 1.0 equiv.) and sodium carbonate (2.9 g, 27.1 mmol, 1.2 equiv.) in isopropanol (75 mL). The flask was then equipped with a reflux condenser, placed in an oil bath preheated to 85 °C, and refluxed for 36 h. Upon complete conversion of 4-fluoro-3-iodobenzoic acid, the reaction was cooled to 23 °C and diluted with saturated aqueous $NaHCO_3$ (100 mL). The mixture was then diluted with water (200 mL), transferred to a separatory funnel, and extracted with dichloromethane (3 × 50 mL). The combined organic layers were washed with brine (100 mL) and dried over anhydrous sodium sulfate. The dried solution was filtered, and the filtrate was concentrated. The resulting residue was purified by flash column chromatography (eluting with 1% ethyl acetate-hexanes) to afford ester **S14** (5.8 g, 83%) as a white crystalline solid.

R_f = 0.5 (10% ethyl acetate-hexanes, UV stain).
^{1}H NMR (400 MHz, $CDCl_3$): δ 8.43 (dd, *J* = 6.1, 2.1 Hz, 1H), 8.01 (ddd, *J* = 8.6, 5.0, 2.1 Hz, 1H), 7.10 (dd, *J* = 8.6, 7.5 Hz, 1H), 5.24 (p, *J* = 6.3 Hz, 1H), 1.37 (d, *J* = 6.2 Hz, 6H).
^{13}C NMR (101 MHz, $CDCl_3$): δ 164.6 (d, *J* = 252.4 Hz), 164.0, 141.2 (d, *J* = 2.9 Hz), 132.0 (d, *J* = 8.7 Hz), 128.9 (d, *J* = 3.3 Hz), 115.6 (d, *J* = 24.6 Hz), 81.1 (d, *J* = 26.5 Hz), 69.2, 22.1.
HRMS-QTOF (m/z): $[M + H]^+$ calculated for $C_{10}H_{11}FIO_2^+$, 308.9782; found 308.9772.

*Synthesis of the boronic acid **S15**:*

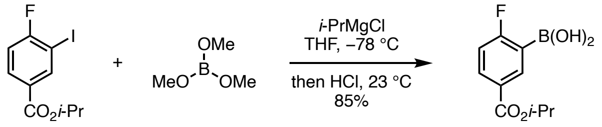

Ester **S14** (3.0 g, 9.7 mmol, 1.0 equiv.) was dissolved in tetrahydrofuran (48.7 mL). The flask was placed in a dry ice-acetone bath and cooled to −78 °C. Isopropylmagnesium chloride (5.8 mL, 2M in tetrahydrofuran, 11.7 mmol, 1.2 equiv.) was added dropwise. The reaction mixture was stirred at −78 °C for 30 min, −30 °C for 30 min, and cooled back to −78 °C. Trimethyl borate (2.2 mL, 19.5 mmol, 2.0 equiv.) was added dropwise. The reaction was stirred at −78 °C for 1 h. Upon complete conversion of the I-Mg exchange intermediate (as monitored by TLC), hydrochloric acid (29.2 mL, 1 M aqueous solution, 29.2 mmol, 3.0 equiv.) was added. The reaction was allowed to gradually warm to 23 °C and stirred for 1 h. The reaction mixture was then diluted with water (200 mL), transferred to separatory funnel, and extracted with ethyl acetate (3 × 40 mL). The combined organic layers were washed with brine (50 mL) and dried over anhydrous sodium sulfate. The dried solution was filtered, and the filtrate was concentrated. The residue obtained was purified by flash column chromatography (eluting firstly with 10% ethyl acetate-hexanes; then with 5% methanol-dichloromethane) to provide boronic acid **S15** as a white crystalline solid (1.9 g, 85%).

R_f: tailing observed on TLC plate, no well-defined R_f.
^{1}H NMR (400 MHz, DMSO-*d6*): δ 8.47 (dd, *J* = 6.1, 2.4 Hz, 0.34H), 8.40 (s, 1H), 8.17 (dd, *J* = 5.9, 2.3 Hz, 0.69H), 7.99 (ddd, *J* = 8.6, 5.4, 2.4 Hz, 1H), 7.23 (t, *J* = 8.7 Hz, 1H), 5.20 – 5.05 (m, 1H), 1.32 (d, *J* = 6.3 Hz, 6H).
^{13}C NMR (101 MHz, DMSO-*d6*): δ 169.5, 167.0, 164.6, 136.9 (d, *J* = 11.0 Hz), 133.0 (d, *J* = 10.1 Hz), 125.9 (d, *J* = 2.9 Hz), 115.5 (d, *J* = 25.7 Hz), 68.2, 21.7.
HRMS-QTOF (m/z): $[M + H]^+$ calculated for $C_{10}H_{13}BFO_4^+$, 227.0885; found 227.0876.

*Synthesis of the salt **28**:*

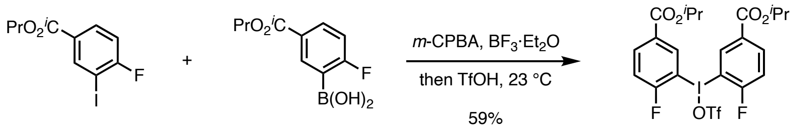

S14 **S15** **28**

Iodobenzene **S14** (3.6 g, 11.7 mmol, 1.0 equiv.) and boron trifluoride diethyl etherate (3.7 mL, 29.2 mmol, 2.5 equiv.) were sequentially added to a solution of *meta*-chloroperoxybenzoic acid (2.8 g, 80 wt.%, 12.9 mmol, 1.1 equiv.) in dichloromethane (58.4 mL). The reaction was stirred at 23 °C for 2 h. The flask was then placed in an ice bath and cooled to 0 °C. Boronic acid **S15** (2.9 g, 12.9 mmol, 1.1 equiv.) was added. The reaction was allowed to warm to 23 °C and stirred for 2 h. Triflic acid (1.1 mL, 12.9 mmol, 1.1 equiv.) was added dropwise. The reaction was stirred for 20 min. The crude reaction mixture was filtered through a silica gel plug (32 g) and eluted with dichloromethane (260 mL). The filtrate was discarded. The product remaining on the column was then eluted with dichloromethane-methanol (520 mL, *v*/*v* = 95:5). The filtrate was collected and concentrated. Diethyl ether (50 mL) was added to the residue to induce the precipitation of salt **28**, the supernatant was decanted. The solid was washed with diethyl ether (2 × 50 mL). The residue obtained was dried under vacuum to provide salt **28** as a white crystalline solid (4.4 g, 59%).

R_f: tailing observed on TLC plate, no well-defined R_f.
^{1}H NMR (400 MHz, DMSO-*d6*): δ 9.09 – 8.99 (m, 2H), 8.23 (dq, *J* = 8.6, 2.1 Hz, 2H), 7.75 – 7.66 (m, 2H), 5.15 (p, *J* = 6.3 Hz, 2H), 1.32 (d, *J* = 6.3 Hz, 12H).
^{13}C NMR (101 MHz, DMSO-*d6*): δ 162.8, 161.9 (d, *J* = 256.6 Hz), 138.1, 136.8 – 136.3 (m), 129.3, 120.7 (q, *J* = 322.3 Hz), 117.3 (dt, *J* = 23.0, 4.7 Hz), 105.1 – 104.6 (m), 69.5, 21.5.
HRMS-QTOF (m/z): $[M - OTf]^+$ calculated for $C_{20}H_{20}F_2IO_4^+$, 489.0369; found 489.0381.

*Synthesis of the oxime **27**:*

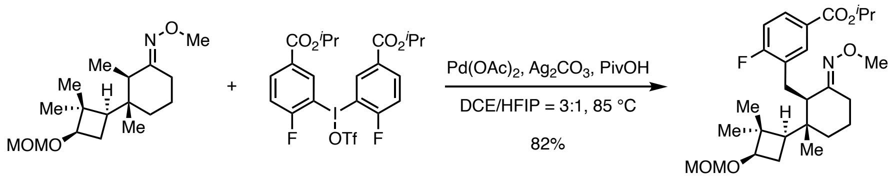

26 **28** **27**

The oxime **26** (90.0 mg, 0.3 mmol, 1.0 equiv.), iodonium salt **28** (202.8 mg, 0.32 mmol, 1.01 equiv.), palladium acetate (6.8 mg, 0.03 mmol, 0.1 equiv.), silver carbonate (166.9 mg, 0.6 mmol, 2.0 equiv.), and pivalic acid (18.5 mg, 0.18 mmol, 0.6 equiv.) were added to a 10 mL reaction tube. Dichloroethane-hexafluoro-2-propanol (3.0 mL, *v*/*v* = 3:1,) was added. The tube was sealed and stirred at room temperature for 5 min, then placed in a heating block preheated to 85 °C and stirred for 10 h. Upon complete conversion of the oxime **26**, the reaction was cooled to 23 °C, diluted with ethyl acetate (10 mL), and passed through a short pad of celite. The filtrate was washed sequentially with saturated aqueous sodium bicarbonate (10 mL), brine (10 mL) and dried over anhydrous sodium sulfate. The dried solution was filtered, and the filtrate was concentrated. The residue obtained was purified by flash column chromatography (eluting with 10% ethyl acetate-hexanes) to provide oxime **27** as a yellow oil (108 mg, 82%).

R_f = 0.3 (10% ethyl acetate-hexanes, UV & CAM stain).
^{1}H NMR (400 MHz, $CDCl_3$): δ 7.95 (dd, *J* = 7.4, 2.2 Hz, 1H), 7.83 (ddd, *J* = 8.6, 5.0, 2.3 Hz, 1H), 6.99 (dd, *J* = 9.8, 8.6 Hz, 1H), 5.22 (p, *J* = 6.3 Hz, 1H), 4.60 (d, *J* = 6.5 Hz, 1H), 4.57 (d, *J* = 6.5 Hz, 1H), 3.66 (s, 3H), 3.56 (t, *J* = 7.4 Hz, 1H), 3.36 (s, 3H), 3.02 – 2.77 (m, 2H), 2.47 – 2.34 (m, 2H), 2.26 (dt, *J* = 16.0, 5.4 Hz, 1H), 2.10 (dt, *J* = 9.1, 6.5 Hz, 1H), 1.92 – 1.72 (m, 2H), 1.61 – 1.52 (m, 3H), 1.50 – 1.41 (m, 1H), 1.34 (dd, *J* = 6.3, 1.8 Hz, 6H), 1.20 (s, 3H), 1.12 (s, 3H), 1.02 (s, 3H).
^{13}C NMR (101 MHz, $CDCl_3$): δ 165.6, 163.1, 158.3, 134.4 (d, *J* = 6.7 Hz), 129.5 (d, *J* = 9.7 Hz), 128.4 (d, *J* = 16.0 Hz), 126.5 (d, *J* = 3.0 Hz), 115.1 (d, *J* = 23.6 Hz), 95.8, 76.9, 68.4, 61.1, 55.5, 49.3, 46.8, 42.6, 41.3, 33.2, 31.4, 27.7, 26.0, 22.9, 22.1, 20.8, 20.7, 18.3.
$[\alpha]_D^{24}$ = − 14.7 (*c* 1.0, $CHCl_3$).
HRMS-QTOF (m/z): $[M + H]^+$ calculated for $C_{27}H_{41}FNO_5^+$, 478.2963; found 478.2980.

*Synthesis of the ketone **S16**:*

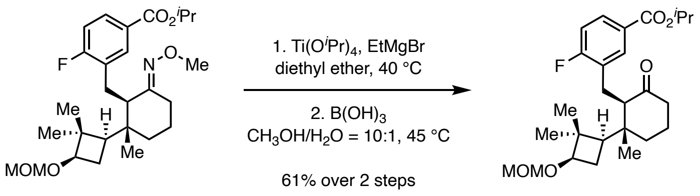

Ethylmagnesium bromide (3M in THF, 4.6 mL, 13.9 mmol, 10.0 equiv.) was added dropwise to a solution of titanium isopropoxide (4.1 mL, 13.9 mmol, 10.0 equiv.) in diethyl ether (70 mL). The reaction was stirred vigorously at 23 °C for 30 min, then warmed to 40 °C and stirred for another 30 min. After cooling to 23 °C, a solution of oxime **27** (665 mg, 1.4 mmol, 1.0 equiv.) in diethyl ether (10 mL) was added. The flask was placed in an oil bath preheated to 40 °C and stirred for 10 h. Upon complete conversion of **27**, the reaction was cooled to 23 °C and diluted with saturated aqueous Rochelle's salt (200 mL). The mixture was transferred to a separatory funnel and extracted with ethyl acetate (5 × 30 mL). The combined organic layers were washed with brine (100 mL) and dried over anhydrous sodium sulfate. The dried solution was filtered, and the filtrate was concentrated to provide the crude imine intermediate. The crude product was used directly in the next reaction without further purification.
Boric acid (430.4 mg, 7.0 mmol, 5.0 equiv.) was added to a solution of the above crude product in methanol-water (33 mL, *v*/*v* = 10:1). The flask was placed in an oil bath preheated to 45 °C and stirred overnight. Upon complete conversion of the imine intermediate, the reaction mixture was concentrated under reduced pressure. The residue obtained was diluted with water (30 mL), transferred to a separatory funnel, and extracted with ethyl acetate (3 × 15 mL). The combined organic layers were washed with brine (30 mL) and dried over anhydrous sodium sulfate. The dried solution was filtered, and the filtrate was concentrated. The residue obtained was purified by flash column chromatography (eluting with 15% ethyl acetate-hexanes) to afford ketone **S16** as a pale-yellow oil (393 mg, 61%).

R_f = 0.5 (20% ethyl acetate-hexanes, UV & CAM stain).
^{1}H NMR (400 MHz, $CDCl_3$): δ 7.95 (dd, *J* = 7.5, 2.2 Hz, 1H), 7.87 – 7.78 (m, 1H), 7.03 – 6.94 (m, 1H), 5.21 (p, *J* = 6.2 Hz, 1H), 4.60 (d, *J* = 6.6 Hz, 1H), 4.58 (d, *J* = 6.5 Hz, 1H), 3.54 (t, *J* = 7.4 Hz, 1H), 3.36 (s, 3H), 3.02 (dd, *J* = 13.9, 10.0 Hz, 1H), 2.79 – 2.71 (m, 1H), 2.56 (dd, *J* = 10.0, 2.8 Hz, 1H), 2.33 (d, *J* = 11.9 Hz, 1H), 2.26 – 2.14 (m, 1H), 2.13 – 2.03 (m, 1H), 2.01 – 1.71 (m, 6H), 1.36 (d, *J* = 3.7 Hz, 3H), 1.34 (d, *J* = 3.5 Hz, 3H), 1.20 (s, 3H), 1.13 (s, 3H), 0.92 (s, 3H).
^{13}C NMR (101 MHz, $CDCl_3$): δ 211.6, 165.5, 164.0 (d, *J* = 251.9 Hz), 133.7 (d, *J* = 6.4 Hz), 129.6 (d, *J* = 9.6 Hz), 128.6 (d, *J* = 15.8 Hz), 126.9 (d, *J* = 3.1 Hz), 115.2 (d, *J* = 23.4 Hz), 95.9, 76.8, 68.6, 58.6, 55.6, 46.8, 45.2, 44.8, 42.0, 33.3, 31.1, 27.7 (d, *J* = 1.9 Hz), 23.7, 23.0, 22.1, 19.2, 18.2.
$[\alpha]_D^{22}$ = −41.7 (*c* 1.0, $CHCl_3$).
HRMS-QTOF (m/z): $[M + Na]^+$ calculated for $C_{26}H_{37}FNaO_5^+$, 471.2517; found 471.2526.

*Synthesis of the alkene **29**:*

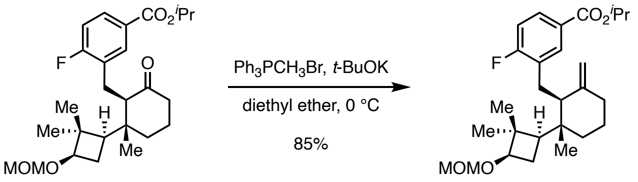

Methyltriphenylphosphonium bromide (3.1 g, 8.7 mmol, 5.0 equiv.) was suspended in diethyl ether (70 mL). The flask was placed in an ice bath and cooled to 0 °C. Potassium *tert*-butoxide (956.1 mg, 8.5 mmol, 4.9 equiv.) was added. The reaction was allowed to warm to 23°C and stirred for 2 h. The reaction was then placed in an ice bath and cooled to 0 °C. A solution of ketone **S16** (780.0 mg, 1.7 mmol, 1.0 equiv.) in diethyl ether (10 mL) was added dropwise. The reaction was stirred at 0 °C for 2 h. Upon complete conversion of ketone **S16**, the reaction was diluted with water (100 mL), transferred to a separatory funnel, and extracted with ethyl acetate (3 × 15 mL). The combined organic layers were washed with brine (50 mL) and dried over anhydrous sodium sulfate. The dried solution was filtered, and the filtrate was concentrated. The residue obtained was purified by flash column chromatography (eluting with 5% ethyl acetate-hexanes) to provide alkene **29** as a pale-yellow oil (660 mg, 85%).

R_f = 0.4 (10% ethyl acetate-hexanes, UV & CAM stain).
^{1}H NMR (400 MHz, $CDCl_3$): δ 7.82 (ddd, *J* = 8.1, 5.0, 2.2 Hz, 1H), 7.75 (dd, *J* = 7.3, 2.2 Hz, 1H), 6.99 (t, *J* = 9.1 Hz, 1H), 5.21 (p, *J* = 6.2 Hz, 1H), 4.63 – 4.53 (m, 3H), 4.11 (s, 1H), 3.52 (dd, *J* = 8.4, 6.3 Hz, 1H), 3.35 (s, 3H), 2.96 (dd, *J* = 13.6, 3.9 Hz, 1H), 2.69 (dd, *J* = 13.6, 11.9 Hz, 1H), 2.37 (dd, *J* = 11.9, 3.9 Hz, 1H), 2.25 – 2.12 (m, 1H), 2.08 – 1.94 (m, 3H), 1.70 (ddd, *J* = 15.0, 12.6, 8.5 Hz, 1H), 1.54 – 1.46 (m, 2H), 1.35 (d, *J* = 6.2 Hz, 7H), 1.25 (s, 1H), 1.14 (s, 3H), 1.12 (s, 3H), 1.08 (s, 3H).
^{13}C NMR (101 MHz, $CDCl_3$): δ 165.6, 164.3 (d, *J* = 251.6 Hz), 146.9, 133.2 (d, *J* = 6.9 Hz), 129.3 (d, *J* = 9.5 Hz), 129.0 (d, *J* = 16.4 Hz), 126.6 (d, *J* = 3.1 Hz), 115.1 (d, *J* = 23.6 Hz), 112.7, 95.8, 76.7, 68.5, 55.5, 51.7, 46.7, 40.9, 39.8, 33.0, 31.5, 30.9, 27.9, 27.7, 22.6, 22.4, 22.1, 18.5.
$[\alpha]_D^{24}$ = −26.0 (*c* 1.0, $CHCl_3$).
HRMS-QTOF (m/z): $[M + H]^+$ calculated for $C_{27}H_{40}FO_4^+$, 447.2905; found 447.2906.

*Synthesis of the alcohol **S17**:*

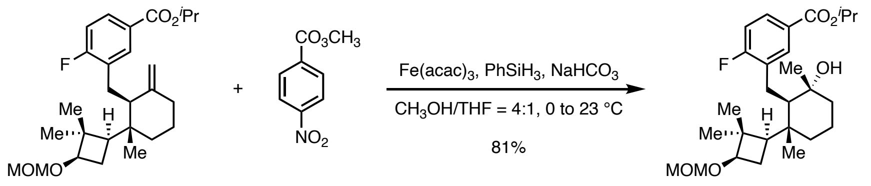

29 **S17**

Alkene **29** (495.0 mg, 1.1 mmol, 1.0 equiv.), methyl 4-nitrobenzenesulfonate (385.2 mg, 1.8 mmol, 1.6 equiv.), sodium bicarbonate (186.2 mg, 2.2 mmol, 2.0 equiv.), and iron(III) acetylacetonate (39.1 mg, 0.11 mmol, 0.1 equiv.) were added to a 50 mL Schlenk tube. The tube was evacuated and backfilled with nitrogen three times. Dry methanol-tetrahydrofuran (18.1 mL, *v*/*v* = 4:1) was added. The reaction tube was placed in an ice bath and cooled to 0 °C. Phenyl silane (0.69 mL, 5.5 mmol, 5.0 equiv.) was added dropwise. The reaction was then allowed to warm gradually to 23 °C and stirred overnight. Upon full conversion of the alkene **29**, the reaction solvent was removed under reduced pressure. The residue was diluted with water (50 mL), transferred to a separatory funnel, and extracted with ethyl acetate (3 × 20 mL). The combined organic layers were washed with brine (30 mL) and dried over anhydrous sodium sulfate. The dried solution was filtered, and the filtrate was concentrated. The oil obtained was purified twice by flash column chromatography (first eluting with 10% acetone-hexanes; second eluting with 15% ethyl acetate) to provide the tertiary alcohol **S17** as a colorless oil (417 mg, 81%).

R_f = 0.4 (20% ethyl acetate-hexanes, UV & CAM stain).
^{1}H NMR (400 MHz, CDCl$_3$): δ 8.04 (dd, *J* = 7.5, 2.3 Hz, 1H), 7.83 (ddd, *J* = 7.9, 5.1, 2.2 Hz, 1H), 7.02 (t, *J* = 9.4 Hz, 1H), 5.22 (p, *J* = 6.2 Hz, 1H), 4.46 (s, 2H), 3.28 (s, 3H), 3.09 (t, *J* = 5.7 Hz, 1H), 2.91 (dd, *J* = 15.0, 4.4 Hz, 1H), 2.77 (dd, *J* = 14.9, 6.4 Hz, 1H), 1.77 (d, *J* = 12.2 Hz, 1H), 1.69 – 1.64 (m, 1H), 1.62 – 1.51 (m, 4H), 1.49 – 1.42 (m, 1H), 1.36 (d, *J* = 6.2 Hz, 8H), 1.28 (s, 4H), 1.13 (s, 3H), 1.02 (s, 3H), 1.01 (s, 3H).
^{13}C NMR (101 MHz, CDCl$_3$): δ 165.5, 163.6 (d, *J* = 250.4 Hz), 133.2 (d, *J* = 6.3 Hz), 131.7 (d, *J* = 15.3 Hz), 129.1 (d, *J* = 9.5 Hz), 127.0 (d, *J* = 3.0 Hz), 115.3 (d, *J* = 24.2 Hz), 95.9, 77.0, 73.9, 68.6, 55.5, 54.7, 46.5, 45.7, 43.4, 41.2, 34.1, 31.3, 27.6, 24.6, 24.1, 22.1, 20.0, 19.1, 18.1.
$[\alpha]_D^{23}$ = −18.2 (*c* 1.0, CHCl$_3$).
HRMS-QTOF (m/z): $[M + Na]^+$ calculated for $C_{27}H_{41}FNaO_5^+$, 487.2830; found 487.2833.

*Synthesis of the ester **30**:*

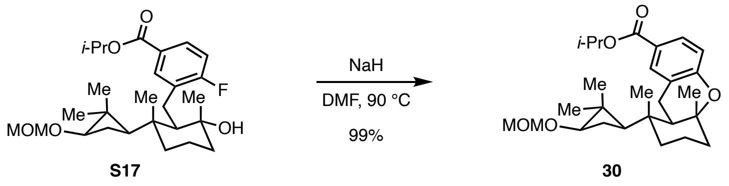

Sodium hydride (36.2 mg, 60% dispersion in mineral oil, 0.9 mmol, 3.0 equiv.) was added to a solution of tertiary alcohol **S17** (140.0 mg, 0.3 mmol, 1.0 equiv.) in dry *N*,*N*-dimethylmethanamide (6 mL). The reaction tube was then placed in an oil bath preheated to 90 °C and stirred for 2 h. Upon complete conversion of **S17**, the reaction was cooled to 23 °C and diluted with water (50 mL). The mixture was transferred to a separatory funnel and extracted with ethyl acetate (4 × 15 mL). The combined organic layers were washed with brine (30 mL) and dried over anhydrous sodium sulfate. The dried solution was filtered, and the filtrate was concentrated. The oil obtained was purified by flash column chromatography (eluting with 5% ethyl acetate-hexanes) to provide ester **30** as a white foam (133 mg, 99%).

R_f = 0.7 (20% ethyl acetate-hexanes, UV & CAM stain).
^{1}H NMR (400 MHz, $CDCl_3$): δ 7.79 (s, 1H), 7.76 (dd, *J* = 8.5, 2.1 Hz, 1H), 6.74 (d, *J* = 8.4 Hz, 1H), 5.21 (p, *J* = 6.2 Hz, 1H), 4.58 (d, *J* = 6.5 Hz, 1H), 4.56 (d, *J* = 6.4 Hz, 1H), 3.52 (t, *J* = 7.4 Hz, 1H), 3.34 (s, 3H), 2.73 (dd, *J* = 16.4, 4.9 Hz, 1H), 2.60 (dd, *J* = 16.4, 13.1 Hz, 1H), 2.01 – 1.91 (m, 2H), 1.82 – 1.64 (m, 4H), 1.57 – 1.45 (m, 4H), 1.34 (d, *J* = 6.2 Hz, 6H), 1.21 (s, 6H), 1.09 (s, 3H), 0.96 (s, 3H).
^{13}C NMR (101 MHz, $CDCl_3$): δ 166.3, 157.3, 131.8, 129.2, 122.5, 122.4, 117.0, 95.8, 78.4, 76.6, 67.8, 55.5, 46.5, 46.0, 44.2, 39.5, 39.0, 33.7, 31.5, 27.6, 23.2, 22.2, 20.6, 19.3, 19.1, 18.1.
$[\alpha]_D^{24}$ = +64.8 (*c* 1.0, $CHCl_3$).
HRMS-QTOF (m/z): $[M + H]^+$ calculated for $C_{27}H_{41}O_5^+$, 445.2949; found 445.2948.

*Synthesis of the acid **31**:*

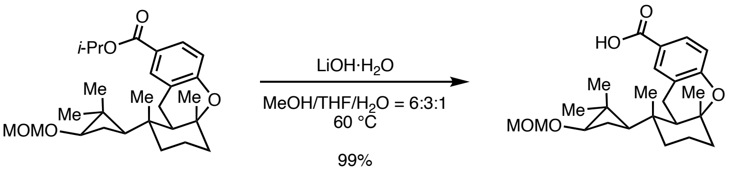

30 **31**

Lithium hydroxide monohydrate (37.7 mg, 0.9 mmol, 3.0 equiv.) was added to a solution of ester **30** (133.0 mg, 0.3 mmol, 1.0 equiv.) in methanol-tetrahydrofuran-water (10 mL, *v*/*v*/*v* = 6:3:1). The flask was then placed in an oil bath preheated to 70 °C and stirred for 24 h. Upon full conversion of the ester **30**, the reaction was cooled to 23 °C and diluted with water (30 mL). The mixture was transferred to a separatory funnel, acidified to pH ~ 2 with 1 M hydrochloric acid, and extracted with ethyl acetate (3 × 10 mL). The combined organic layers were washed with brine (20 mL) and dried over anhydrous sodium sulfate. The dried solution was filtered, and the filtrate was concentrated. The oil obtained was purified by flash column chromatography (eluting with 2% methanol-dichloromethane) to afford the acid **31** as a white foam (119 mg, 99%).

R_f = 0.4 (20% ethyl acetate-hexanes, UV & CAM stain).
^{1}H NMR (400 MHz, $CDCl_3$): δ 7.87 (d, *J* = 2.1 Hz, 1H), 7.83 (dd, *J* = 8.5, 2.1 Hz, 1H), 6.78 (d, *J* = 8.5 Hz, 1H), 4.58 (t, *J* = 1.3 Hz, 2H), 3.53 (t, *J* = 7.4 Hz, 1H), 3.34 (s, 3H), 2.74 (dd, *J* = 16.5, 4.9 Hz, 1H), 2.61 (dd, *J* = 16.4, 13.1 Hz, 1H), 2.03 – 1.93 (m, 2H), 1.83 – 1.64 (m, 4H), 1.61 – 1.45 (m, 4H), 1.23 (s, 3H), 1.22 (s, 3H), 1.10 (s, 3H), 0.97 (s, 3H).
^{13}C NMR (101 MHz, $CDCl_3$): δ 172.1, 158.3, 132.7, 130.0, 122.6, 120.8, 117.2, 95.8, 78.7, 76.6, 55.5, 46.6, 46.0, 44.2, 39.5, 39.0, 33.7, 31.6, 27.6, 23.2, 20.7, 19.3, 19.0, 18.1.
$[\alpha]_D^{22}$ = +89.7 (*c* 1.0, $CHCl_3$).
HRMS-QTOF (m/z): $[M + H]^+$ calculated for $C_{24}H_{35}O_5^+$, 403.2479; found 403.2482.

The relative stereochemistry at the C16 position was established by NOESY experiment analysis. Correlation between the C18 methyl substituent and the C16 methyl substituent supports the relative assignment shown (see page S83).

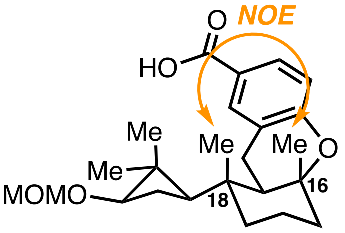

Synthesis of the phenol ***33***:

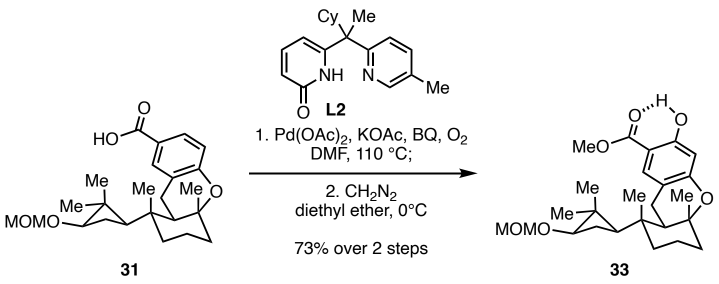

Acid **31** (90.0 mg, 0.22 mmol, 1.0 equiv.), palladium acetate (5.0 mg, 0.022 mmol, 0.1 equiv.), ligand **L2** (6.6 mg, 0.022 mmol, 0.1 equiv.), potassium acetate (43.9 mg, 0.45 mmol, 2.0 equiv.), and *p*-benzoquinone (36.3 mg, 0.34 mmol, 1.5 equiv.) were added to a 10 mL reaction tube. The tube was then evacuated and backfilled with oxygen three times. DMF (3.7 mL, bubbled with oxygen for 30 min before adding) was added and the reaction was stirred at 23 °C for 10 min. The tube was then placed in an oil bath preheated to 110 °C and stirred under 1 atm oxygen atmosphere for 24 h. Upon complete conversion of **31** (as monitored by LC-MS), the reaction was cooled to 23 °C and diluted with water (20 mL). The mixture was transferred to a separatory funnel, acidified to pH ~2 with 1 M hydrochloric acid, and extracted with ethyl acetate (3 × 10 mL). The combined organic layers were washed sequentially with saturated aqueous sodium sulfite (10 mL), brine (10 mL) and dried over anhydrous sodium sulfate. The dried solution was filtered, and the filtrate was concentrated. The residue obtained was directly used in the methylation reaction without further purification.

Diazomethane was prepared according to the method of Arndt.[10] Diazomethane (~ 0.6 M in diethyl ether, freshly prepared) was added to a solution of the above crude product in diethyl ether (4 mL) at 0 °C until the solution turned pale yellow. The solvent was then removed under a stream of nitrogen. The oil obtained was purified by flash column chromatography (eluting with 5% ethyl acetate-hexanes) to afford **33** (71 mg, 73% over 2 steps) as a white foam.

R_f = 0.5 (20% ethyl acetate-hexanes, UV & CAM stain).
^{1}H NMR (400 MHz, $CDCl_3$): δ 10.60 (s, 1H), 7.57 (s, 1H), 6.30 (s, 1H), 4.57 (d, J = 6.5 Hz, 1H), 4.55 (d, J = 6.5 Hz, 1H), 3.89 (s, 3H), 3.49 (dd, J = 8.2, 6.8 Hz, 1H), 3.33 (s, 3H), 2.62 (dd, J = 15.9, 4.8 Hz, 1H), 2.49 (ddd, J = 15.8, 13.2, 1.3 Hz, 1H), 2.00 – 1.89 (m, 2H), 1.82 – 1.69 (m, 3H), 1.68 – 1.60 (m, 1H), 1.57 – 1.44 (m, 4H), 1.21 (s, 3H), 1.19 (s, 3H), 1.08 (s, 3H), 0.94 (s, 3H).
^{13}C NMR (101 MHz, $CDCl_3$): δ 170.5, 161.5, 159.8, 131.4, 114.7, 105.3, 104.2, 95.8, 78.7, 76.6, 55.5, 52.0, 46.5, 46.0, 44.3, 39.4, 39.0, 33.7, 31.5, 27.6, 22.5, 20.7, 19.3, 19.0, 18.1.
$[\alpha]_D^{24}$ = +70.9 (*c* 1.0, $CHCl_3$).
HRMS-QTOF (m/z): $[M + H]^+$ calculated for $C_{25}H_{37}O_6^+$, 433.2585; found 433.2582.

*Synthesis of the phenol **33** and **S18**:*

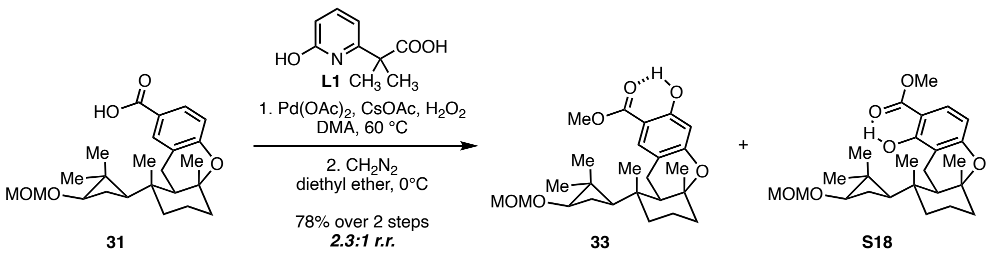

Acid **31** (250.0 mg, 0.62 mmol, 1.0 equiv.), lignad **L1** (11.3 mg, 0.062 mmol, 0.1 equiv.), palladium acetate (7.0 mg, 0.031 mmol, 0.05 equiv.) were added to a 10 mL reaction tube. Dimethylacetamide (6.2 mL) was added. The mixture was stirred at 23 °C for 5 min. Cesium acetate (178.8 mg, 0.93 mmol, 1.5 equiv.) was added. The mixture was stirred until complete dissolution. H_2O_2 (133 μL, 50 wt. % in H_2O, 2.17 mmol, 3.5 equiv.) was added. The tube was placed in a heating block preheated to 60 °C and stirred for 1 h. Upon complete conversion of **31** (as monitored by LC-MS), the reaction was cooled to 23 °C and diluted sequentially with saturated sodium sulfite (1 mL) and water (30 mL). The mixture was transferred to a separatory funnel, acidified to pH ~ 2 with 1 M hydrochloric acid, and extracted with ethyl acetate (3 × 10 mL). The combined organic layers were washed with brine (20 mL) and dried over anhydrous sodium sulfate. The dried solution was filtered, and the filtrate was concentrated. ^{1}H NMR analysis of the crude products revealed an inseparable mixture in a 2.3:1 ratio. The mixture was directly used in the next reaction without further purification.

Diazomethane[10] (~ 0.6 M in ether, freshly prepared) was added to a solution of the above crude product in ether (4 mL) at 0 °C until the solution turned pale yellow. The solvent was then removed under a stream of nitrogen. The oil obtained was purified by flash column chromatography (eluting with 5% ethyl acetate-hexanes) to afford **33** (146 mg, 54%) and **S18** (63 mg, 24%) as white foams.

Data of **33**: see page S37.

Data of **S18**:
R_f = 0.6 (20% ethyl acetate-hexanes, UV & CAM stain).
^{1}H NMR (400 MHz, $CDCl_3$): δ 11.34 (s, 1H), 7.58 (d, *J* = 8.8 Hz, 1H), 6.30 (d, *J* = 8.8 Hz, 1H), 4.57 (d, *J* = 6.5 Hz, 1H), 4.55 (d, *J* = 6.4 Hz, 1H), 3.90 (s, 3H), 3.57 – 3.49 (m, 1H), 3.33 (s, 3H), 2.74 (dd, *J* = 16.9, 4.9 Hz, 1H), 2.29 (dd, *J* = 16.9, 13.2 Hz, 1H), 2.01 – 1.90 (m, 2H), 1.83 – 1.68 (m, 4H), 1.58 – 1.43 (m, 4H), 1.21 (s, 3H), 1.21 (s, 3H), 1.09 (s, 3H), 0.98 (s, 3H).
^{13}C NMR (101 MHz, $CDCl_3$): δ 171.1, 161.2, 159.2, 128.4, 110.5, 109.2, 104.1, 95.8, 78.5, 76.6, 55.5, 52.0, 46.6, 45.7, 43.6, 39.4, 39.1, 33.7, 31.4, 27.6, 20.4, 19.3, 19.0, 18.2, 17.6.
$[\alpha]_D^{24}$ = +85.3 (*c* 1.0, $CHCl_3$).
HRMS-QTOF (m/z): $[M + H]^+$ calculated for $C_{25}H_{37}O_6^+$, 433.2585; found 433.2583.

*Synthesis of the ester **S19**:*

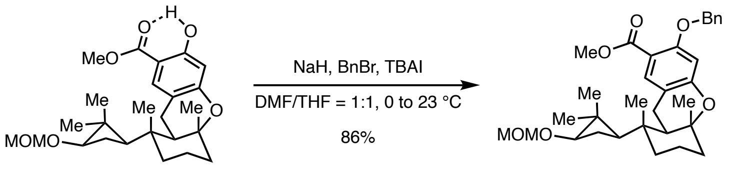

Phenol **33** (86.0 mg, 0.2 mmol, 1.0 equiv.) and tetrabutylammonium iodide (3.7 mg, 9.9 μmol, 0.05 equiv.) were added to a 10 mL reaction tube. *N*,*N*-Dimethylformamide-tetrahydrofuran (4 mL, *v*/*v* = 1:1) was added. The tube was placed in an ice bath and cooled to 0 °C. Sodium hydride (23.9 mg, 60% dispersion in mineral oil, 0.6 mmol, 3.0 equiv.) was added and the mixture was stirred for 5 min. BnBr (47.3 μL, 0.4 mmol, 2.0 equiv.) was added. The reaction was allowed to warm gradually to 23 °C and stirred for 3 h. Upon complete conversion of phenol **33**, the reaction was diluted with water (30 mL), transferred to a separatory funnel, and extracted with ethyl acetate (3 × 10 mL). The combined organic layers were washed with brine (20 mL) and dried over anhydrous sodium sulfate. The dried solution was filtered, and the filtrate was concentrated. The oil obtained was purified by flash column chromatography (eluting with 5% ethyl acetate-hexanes) to provide ester **S19** as a white foam (89.0 mg, 0.17 mmol, 86%).

R_f = 0.4 (20% ethyl acetate-hexanes, UV & CAM stain).
^{1}H NMR (400 MHz, $CDCl_3$): δ 7.68 (s, 1H), 7.50 (d, *J* = 7.1 Hz, 2H), 7.38 (t, *J* = 7.5 Hz, 2H), 7.29 (t, *J* = 7.4 Hz, 1H), 6.40 (s, 1H), 5.11 (d, *J* = 12.1 Hz, 1H), 5.07 (d, *J* = 12.2 Hz, 1H), 4.58 (d, *J* = 6.4 Hz, 1H), 4.56 (d, *J* = 6.6 Hz, 1H), 3.86 (s, 3H), 3.51 (dd, *J* = 8.1, 6.8 Hz, 1H), 3.34 (s, 3H), 2.65 (dd, *J* = 16.0, 4.8 Hz, 1H), 2.51 (dd, *J* = 16.0, 13.1 Hz, 1H), 1.95 (ddd, *J* = 16.1, 8.3, 4.6 Hz, 2H), 1.78 – 1.63 (m, 4H), 1.56 – 1.43 (m, 4H), 1.22 (s, 3H), 1.21 (s, 3H), 1.09 (s, 3H), 0.95 (s, 3H).
^{13}C NMR (101 MHz, $CDCl_3$): δ 166.5, 158.7, 157.9, 137.1, 134.1, 128.6, 127.7, 126.9, 114.9, 112.1, 102.2, 95.9, 78.7, 76.6, 70.6, 55.5, 51.8, 46.5, 46.0, 44.4, 39.5, 39.0, 33.7, 31.6, 27.6, 22.4, 20.6, 19.3, 19.1, 18.1.
$[\alpha]_D^{25}$ = +49.7 (*c* 1.0, $CHCl_3$).
HRMS-QTOF (m/z): $[M + H]^+$ calculated for $C_{32}H_{43}O_6^+$, 523.3054; found 523.3058.

*Synthesis of the acid **34**:*

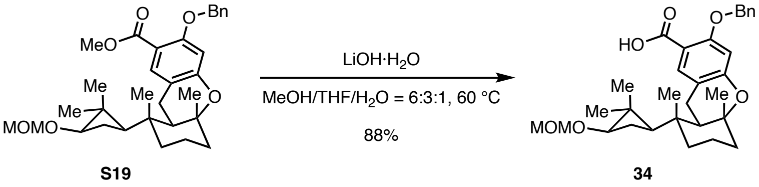

Lithium hydroxide monohydrate (21.4 mg, 0.51 mmol, 3.0 equiv.) was added to a solution of ester **S19** (89.0 mg, 0.17 mmol, 1.0 equiv.) in methanol-tetrahydrofuran-water (5.6 mL, *v*/*v*/*v* = 6:3:1). The flask was placed in an oil bath preheated to 60 °C and stirred for 24 h. Upon complete conversion of ester **S19**, the reaction was cooled to 23 °C and diluted with water (30 mL). The mixture was transferred to a separatory funnel, acidified to pH ~ 2 with 1 M hydrochloric acid, and extracted with ethyl acetate (3 × 10 mL). The combined organic layers were washed with brine (20 mL) and dried over anhydrous sodium sulfate. The dried solution was filtered, and the filtrate was concentrated. The oil obtained was purified with flash column chromatography (eluting with 20% ethyl acetate-hexanes) to afford acid **34** (76.0 mg, 88%) as a white foam.

R_f = 0.4 (30% ethyl acetate-hexanes, UV & CAM stain).
^{1}H NMR (400 MHz, $CDCl_3$): δ 10.59 (s, 1H), 7.93 (s, 1H), 7.47 – 7.34 (m, 5H), 6.48 (s, 1H), 5.19 (d, *J* = 11.0 Hz, 1H), 5.15 (d, *J* = 11.0 Hz, 1H), 4.58 (d, *J* = 6.4 Hz, 1H), 4.56 (d, *J* = 6.5 Hz, 1H), 3.52 (dd, *J* = 8.2, 6.8 Hz, 1H), 3.34 (s, 3H), 2.69 (dd, *J* = 16.2, 4.7 Hz, 1H), 2.53 (dd, *J* = 15.8, 13.1 Hz, 1H), 2.01 – 1.90 (m, 2H), 1.82 – 1.70 (m, 3H), 1.66 (dd, *J* = 12.5, 7.1 Hz, 1H), 1.50 (dd, *J* = 13.3, 4.9 Hz, 4H), 1.23 (s, 3H), 1.21 (s, 3H), 1.09 (s, 3H), 0.96 (s, 3H).
^{13}C NMR (101 MHz, $CDCl_3$): δ 165.5, 159.0, 157.2, 135.6, 134.6, 129.2, 128.1, 117.2, 109.9, 101.4, 95.9, 79.4, 76.6, 72.3, 55.5, 46.6, 46.0, 44.3, 39.5, 39.0, 33.7, 31.5, 27.6, 22.4, 20.7, 19.3, 19.0, 18.1.
$[\alpha]_D^{24}$ = + 53.6 (*c* 1.0, $CHCl_3$).
HRMS-QTOF (m/z): $[M + H]^+$ calculated for $C_{31}H_{41}O_6^+$, 509.2898; found 509.2896.

*Synthesis of the amide **37**:*

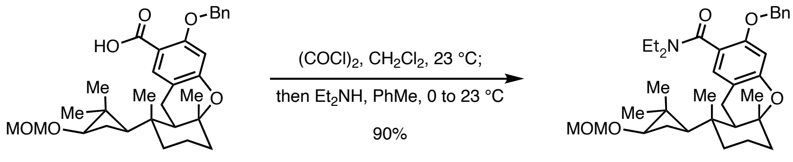

Oxalyl chloride (34.4 μL, 0.39 mmol, 10.0 equiv.) was added to a solution of acid **34** (20.0 mg, 0.039 mmol, 1.0 equiv.) in dichloromethane (2 mL). The reaction was stirred at 23 °C for 5 h. The solvent was then removed under a stream of nitrogen. Dry toluene (2 mL) was added. The flask was placed in an ice bath and cooled to 0 °C. Diethylamine (40.7 μL, 0.39 mmol, 10.0 equiv.) was added. The reaction was allowed to warm gradually to 23 °C and stirred for 30 min. Upon complete conversion of the acyl chloride intermediate, the reaction was diluted with saturated aqueous sodium bicarbonate (20 mL), transferred to a separatory funnel, and extracted with ethyl acetate (3 × 5 mL). The combined organic layers were washed with brine (10 mL) and dried over anhydrous sodium sulfate. The dried solution was filtered, and the filtrate was concentrated. The residue obtained was purified by flash column chromatography (25% ethyl acetate-hexanes) to provide amide **37** (20.0 mg, 90%) as a colorless oil.

R_f = 0.6 (50% ethyl acetate-hexanes, UV & CAM stain).
^{1}H NMR (400 MHz, $CDCl_3$): δ 7.39 – 7.27 (m, 5H), 6.93 (s, 1H), 6.36 (s, 1H), 5.00 (d, *J* = 12.5 Hz, 1H), 4.95 (d, *J* = 12.0 Hz, 1H), 4.58 (d, *J* = 6.4 Hz, 1H), 4.56 (d, *J* = 6.5 Hz, 1H), 3.77 (br. s, 1H), 3.52 (t, *J* = 7.5 Hz, 1H), 3.34 (s, 3H), 3.33 – 2.96 (m, 3H), 2.70 – 2.37 (m, 2H), 2.03 – 1.87 (m, 2H), 1.82 – 1.65 (m, 4H), 1.57 – 1.44 (m, 4H), 1.20 (s, 6H), 1.13 (t, *J* = 7.1 Hz, 3H), 1.08 (s, 3H), 1.00 (t, *J* = 7.1 Hz, 3H), 0.94 (s, 3H).
^{13}C NMR (101 MHz, $CDCl_3$): δ 169.1, 154.3, 153.9, 137.0, 128.8, 128.5, 127.8, 127.2, 119.6, 115.2, 101.3, 95.8, 77.9, 76.6, 70.3, 55.5, 46.5, 46.0, 44.5, 42.9, 39.6, 39.0, 38.8, 33.8, 31.5, 27.6, 22.5, 20.5, 19.3, 19.1, 18.1, 14.2, 12.9.
$[\alpha]_D^{23}$ = + 27.5 (*c* 1.0, $CHCl_3$).
HRMS-QTOF (m/z): $[M + H]^+$ calculated for $C_{35}H_{50}NO_5^+$, 564.3684; found 564.3683.

*Synthesis of the lactone **38**:*

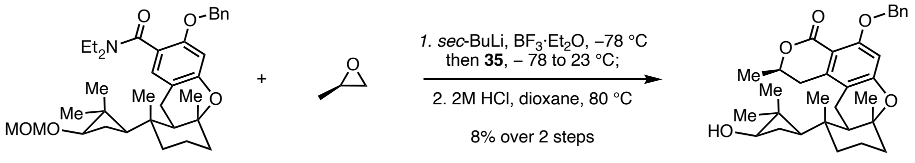

Amide **37** (20.0 mg, 0.036 mmol, 1.0 equiv.) was dissolved in tetrahydrofuran (2.0 mL). The reaction tube was placed in a dry ice-acetone bath and cooled to −78 °C. *sec*-Butyllithium (0.14 mL, 1.3 M in cyclohexane, 0.18 mmol, 5.0 equiv.) was added dropwise. The reaction was stirred at −78 °C for 2 h. Boron trifluoride diethyl etherate (22.2 µL, 0.18 mmol, 5.0 equiv.) and epoxide **35** (20.0 µL, 0.35 mmol, 10.0 equiv.) were sequentially added. The reaction was allowed to warm gradually to 23 °C and stirred overnight. The reaction mixture was diluted with water (20 mL), transferred to a separatory funnel, and extracted with ethyl acetate (3 × 5 mL). The combined organic layers were washed with brine (10 mL) and dried over anhydrous sodium sulfate. The dried solution was filtered, and the filtrate was concentrated. The residue obtained was used directly in the next reaction without further purification.

Hydrochloric acid (0.53 mL, 4 M in 1,4-dioxanes, 2.13 mmol, 60.0 equiv.) was added to a solution of the above crude product in 1,4-dioxanes (0.53 mL). The reaction was then placed in a heating block preheated to 80 °C and stirred for 5 h. The solvent was then removed under a stream of nitrogen. The residue obtained was purified by preparative thin layer chromatography (developing in 50% ethyl acetate-hexanes) to afford **38** as a colorless oil (1.5 mg, 8% over 2 steps).

R_f = 0.3 (50% ethyl acetate-hexanes, UV & CAM stain).
^{1}H NMR (400 MHz, $CDCl_3$): δ 7.53 (d, *J* = 7.1 Hz, 2H), 7.36 (t, *J* = 7.4 Hz, 2H), 7.32 – 7.26 (m, 1H), 6.36 (s, 1H), 5.21 (d, *J* = 12.5 Hz, 1H), 5.10 (d, *J* = 12.5 Hz, 1H), 4.54 (dtd, *J* = 12.5, 6.3, 2.9 Hz, 1H), 3.70 – 3.60 (m, 1H), 2.91 (dd, *J* = 16.5, 3.0 Hz, 1H), 2.61 (dd, *J* = 16.6, 11.3 Hz, 1H), 2.49 (dd, *J* = 16.0, 5.2 Hz, 1H), 2.20 (dd, *J* = 15.9, 13.0 Hz, 1H), 2.08 – 2.00 (m, 1H), 1.95 – 1.86 (m, 1H), 1.77 – 1.71 (m, 2H), 1.69 – 1.64 (m, 2H), 1.55 – 1.51 (m, 7H), 1.20 (s, 3H), 1.17 (s, 3H), 1.08 (s, 3H), 0.97 (s, 3H).
^{13}C NMR (151 MHz, $CDCl_3$): δ 163.0, 160.5, 158.6, 141.7, 136.9, 128.6, 127.7, 126.8, 111.5, 107.4, 101.6, 78.2, 72.8, 72.0, 70.5, 46.7, 46.1, 44.2, 39.3, 39.1, 33.8, 32.6, 31.1, 29.9, 21.0, 20.4, 20.4, 19.2, 19.0, 17.4.
$[\alpha]_D^{24}$ = + 25.8 (*c* 0.1, $CHCl_3$).
HRMS-QTOF (m/z): $[M + H]^+$ calculated for $C_{32}H_{41}O_5^+$, 505.2949; found 505.2962.

*Synthesis of the phenol **39**:*

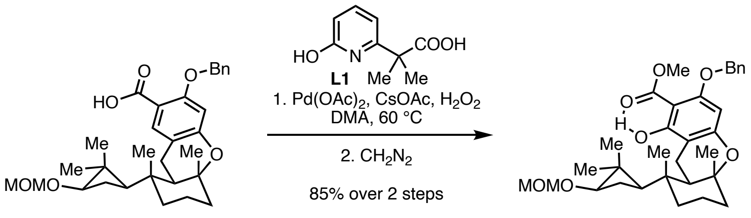

Acid **34** (56 mg, 0.11 mmol, 1.0 equiv.), ligand **L1** (4.0 mg, 0.022 mmol, 0.2 equiv.), palladium acetate (2.5 mg, 0.011 mmol, 0.1 equiv.) were added to a 10 mL reaction tube. Dimethylacetamide (2.2 mL) was added, and the mixture was stirred for 5 min. Cesium acetate (31.7 mg, 0.17 mmol, 1.5 equiv.) was added and the mixture was stirred until complete dissolution. H_2O_2 (24 μL, 50 wt. % in H_2O, 0.39 mmol, 3.5 equiv.) was added. The reaction was placed in a heating block preheated to 60 °C and stirred for 1 h. Upon complete conversion of acid **34** (as monitored by LC-MS), the reaction was cooled to 23 °C and diluted sequentially with saturated sodium sulfite (1 mL) and water (20 mL). The mixture was transferred to a separatory funnel, acidified to pH ~ 2 with 1 M hydrochloric acid, and extracted with ethyl acetate (3 × 5 mL). The combined organic layers were washed with brine (10 mL) and dried over anhydrous sodium sulfate. The dried solution was filtered, and the filtrate was concentrated. The oil obtained was used directly in the next reaction without further purification.

Diazomethane[10] (~ 0.6 M in ether, freshly prepared) was added to a solution of the above crude product in ether (4 mL) at 0 °C until the solution turned pale yellow. The solvent was then removed under a stream of nitrogen. The resulting oil was purified by flash column chromatography (eluting with 8% ethyl acetate-hexanes) to afford phenol **39** (49 mg, 85% over 2 steps) as a white foam.

R_f = 0.6 (20% ethyl acetate-hexanes, UV & CAM stain).
^{1}H NMR (400 MHz, $CDCl_3$): δ 12.41 (s, 1H), 7.47 (d, *J* = 6.8 Hz, 2H), 7.39 (t, *J* = 7.5 Hz, 2H), 7.31 (t, *J* = 7.2 Hz, 1H), 5.96 (s, 1H), 5.05 (d, *J* = 12.0 Hz, 1H), 5.01 (d, *J* = 11.9 Hz, 1H), 4.58 (d, *J* = 6.5 Hz, 1H), 4.56 (d, *J* = 6.4 Hz, 1H), 3.91 (s, 3H), 3.53 (t, *J* = 7.5 Hz, 1H), 3.34 (s, 3H), 2.68 (dd, *J* = 16.5, 4.9 Hz, 1H), 2.24 (dd, *J* = 16.5, 13.2 Hz, 1H), 2.03 – 1.90 (m, 2H), 1.84 – 1.68 (m, 4H), 1.56 – 1.43 (m, 4H), 1.23 (s, 3H), 1.22 (s, 3H), 1.09 (s, 3H), 0.98 (s, 3H).
^{13}C NMR (101 MHz, $CDCl_3$): δ 172.4, 163.0, 159.4, 159.0, 137.0, 128.5, 127.8, 126.9, 103.6, 96.0, 95.9, 93.5, 79.0, 76.7, 70.6, 55.5, 52.2, 46.6, 45.7, 43.9, 39.4, 39.1, 33.7, 31.4, 27.6, 20.4, 19.3, 19.0, 18.2, 17.4.
$[\alpha]_D^{24}$ = + 57.1 (*c* 1.0, $CHCl_3$).
HRMS-QTOF (m/z): $[M + H]^+$ calculated for $C_{32}H_{43}O_7^+$, 539.3003; found 539.3008.

*Synthesis of the triflate **S20**:*

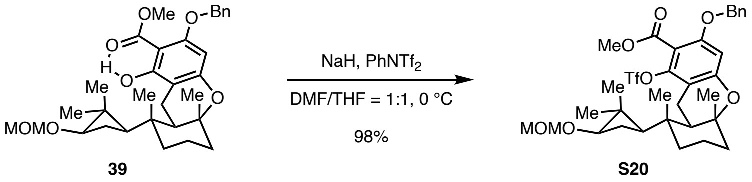

Phenol **39** (67.0 mg, 0.12 mmol, 1.0 equiv.) was dissolved in *N*,*N*-dimethylformamide-tetrahydrofuran (2.4 mL, *v/v* = 1:1). The flask was placed in an ice bath and cooled to 0 °C. Sodium hydride (10.0 mg, 60% dispersion in mineral oil, 0.25 mmol, 2.0 equiv.) was added. The reaction mixture was stirring for 5 minutes. $PhNTf_2$ (67.0 mg, 0.19 mmol, 1.5 equiv.) was added. The reaction was stirred at 0 °C for 30 min. Upon complete conversion of phenol **39**, the reaction was diluted with saturated aqueous ammonium chloride (30 mL), transferred to a separatory funnel, and extracted with dichloromethane (3 × 5 mL). The combined organic layers were washed successively with water (10 mL), 1 M NaOH (3 × 5 mL), and brine (10 mL). The organic layer was dried over anhydrous sodium sulfate. The dried solution was filtered, and the filtrate was concentrated. The residue obtained was purified by flash column chromatography (eluting with 10% acetone-hexanes) to afford triflate **S20** as a white foam (82 mg, 98%).

R_f = 0.5 (20% ethyl acetate-hexanes, UV & CAM stain).
^{1}H NMR (400 MHz, $CDCl_3$): δ 7.43 – 7.26 (m, 5H), 6.43 (s, 1H), 5.08 (d, *J* = 12.0 Hz, 1H), 5.02 (d, *J* = 12.0 Hz, 1H), 4.59 (d, *J* = 6.4 Hz, 1H), 4.56 (d, *J* = 6.5 Hz, 1H), 3.87 (s, 3H), 3.52 (dd, *J* = 8.3, 6.8 Hz, 1H), 3.35 (s, 3H), 2.75 (dd, *J* = 16.5, 4.8 Hz, 1H), 2.43 (dd, *J* = 16.4, 13.2 Hz, 1H), 2.02 – 1.89 (m, 2H), 1.81 – 1.68 (m, 3H), 1.62 (dd, *J* = 12.5, 7.2 Hz, 1H), 1.53 – 1.39 (m, 4H), 1.21 (s, 3H), 1.20 (s, 3H), 1.09 (s, 3H), 0.95 (s, 3H).
^{13}C NMR (101 MHz, $CDCl_3$): δ 164.1, 156.6, 145.2, 136.2, 128.7, 128.1, 127.0, 118.7 (q, *J* = 320.2 Hz), 110.4, 109.9, 101.9, 95.9, 79.1, 76.6, 71.0, 55.5, 52.6, 46.6, 46.1, 43.6, 39.2, 39.0, 33.7, 31.5, 27.5, 20.4, 19.2, 18.8, 18.6, 18.0.
$[\alpha]_D^{23}$ = + 34.5 (*c* 1.0, $CHCl_3$).
HRMS-QTOF (m/z): $[M + H]^+$ calculated for $C_{33}H_{42}F_3O_9S^+$, 671.2496; found 671.2518.

*Synthesis of the olefin **40**:*

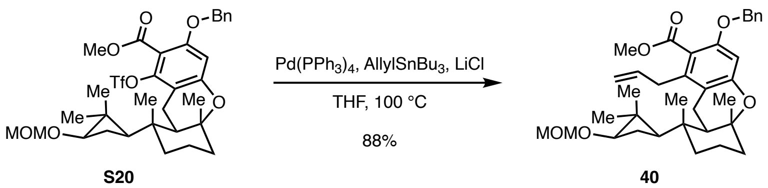

Triflate **S20** (50.0 mg, 0.075 mmol, 1.0 equiv.), tetrakis(triphenylphosphine)palladium(0) (8.6 mg, 0.0075 mmol, 0.1 equiv.), and lithium chloride (9.5 mg, 0.22 mmol, 3.0 equiv.) were added to a 10 mL Schlenk storage tube. The tube was then evacuated and backfilled with nitrogen three times. Degassed tetrahydrofuran (1.5 mL) and allyltributylstannane (35 μL, 0.11 mmol, 1.5 equiv.) were sequentially added. The tube was sealed and placed in a heating block preheated to 100 °C and stirred for 36 h. Upon complete conversion of triflate **S20** (as monitored by LC-MS), the reaction was cooled to 23 °C and diluted with saturated aqueous potassium fluoride (20 mL). The mixture was transferred to a separatory funnel and extracted with ethyl acetate (3 × 5 mL). The combined organic layers were washed with brine (10 mL) and dried over anhydrous sodium sulfate. The dried solution was filtered, and the filtrate was concentrated. The residue obtained was purified by flash column chromatography (eluting with 8% ethyl acetate-hexanes) to provide olefin **40** as a white foam (37 mg, 88%).

R_f = 0.6 (20% ethyl acetate-hexanes, UV & CAM stain).
^{1}H NMR (400 MHz, $CDCl_3$): δ 7.41 – 7.27 (m, 5H), 6.32 (s, 1H), 5.90 (ddt, *J* = 16.4, 10.1, 6.0 Hz, 1H), 5.10 – 4.94 (m, 4H), 4.58 (d, *J* = 6.5 Hz, 1H), 4.56 (d, *J* = 6.5 Hz, 1H), 3.84 (s, 3H), 3.50 (t, *J* = 7.6 Hz, 1H), 3.35 (s, 5H), 2.62 (dd, *J* = 16.1, 4.9 Hz, 1H), 2.29 (dd, *J* = 16.1, 13.1 Hz, 1H), 1.96 – 1.86 (m, 2H), 1.80 – 1.68 (m, 3H), 1.63 (dd, *J* = 12.5, 7.3 Hz, 1H), 1.53 – 1.42 (m, 4H), 1.21 (s, 3H), 1.16 (s, 3H), 1.09 (s, 3H), 0.95 (s, 3H).
^{13}C NMR (101 MHz, $CDCl_3$): δ 169.3, 155.2, 155.1, 137.1, 137.0, 135.8, 128.5, 127.8, 127.0, 117.2, 115.7, 114.3, 100.2, 95.8, 77.3, 76.6, 70.3, 55.5, 52.1, 46.5, 46.2, 44.3, 39.4, 39.0, 34.5, 33.8, 31.7, 27.5, 20.3, 20.0, 19.3, 19.0, 18.1.
$[\alpha]_D^{23}$ = + 47.9 (*c* 1.0, $CHCl_3$).
HRMS-QTOF (m/z): $[M + H]^+$ calculated for $C_{35}H_{47}O_6^+$, 563.3367; found 563.3376.

*Synthesis of the ketone **S21**:*

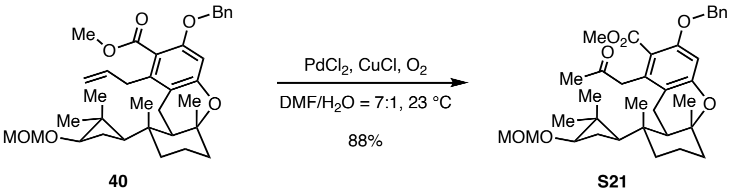

Olefin **40** (44.0 mg, 0.078 mmol, 1.0 equiv.), palladium(II) chloride (3.5 mg, 0.020 mmol, .025 equiv.), and copper(I) chloride (7.7 mg, 0.078 mmol, 1.0 equiv.) were added to a 10 mL reaction tube. *N,N*-dimethylformamide-water (4.6 mL, *v/v* = 7:1) was added. The reaction was purged with O_2 and stirred under a 1 atm O_2 atmosphere at 23 °C for 36 h. Upon complete conversion of olefin **40**, the reaction was diluted with water (20 mL), transferred to a separatory funnel, and extracted with ethyl acetate (3 × 5 mL). The combined organic layers were washed with brine (10 mL) and dried over anhydrous sodium sulfate. The dried solution was filtered, and the filtrate was concentrated. The residue obtained was purified by flash column chromatography (eluting with 20% ethyl acetate-hexanes) to afford ketone **S21** as a colorless oil (40.0 mg, 88%).

R_f = 0.2 (20% ethyl acetate-hexanes, UV & CAM stain).
^{1}H NMR (400 MHz, $CDCl_3$): δ 7.41 – 7.28 (m, 5H), 6.38 (s, 1H), 5.04 (d, *J* = 12.0 Hz, 1H), 5.00 (d, *J* = 12.0 Hz, 1H), 4.58 (d, *J* = 6.5 Hz, 1H), 4.56 (d, *J* = 6.5 Hz, 1H), 3.84 (s, 3H), 3.70 (s, 2H), 3.52 (dd, *J* = 8.3, 6.9 Hz, 1H), 3.34 (s, 3H), 2.43 (dd, *J* = 16.0, 5.0 Hz, 1H), 2.25 – 2.09 (m, 4H), 1.93 (dd, *J* = 10.2, 7.1 Hz, 2H), 1.80 – 1.67 (m, 3H), 1.65 – 1.56 (m, 1H), 1.54 – 1.43 (m, 4H), 1.20 (s, 3H), 1.17 (s, 3H), 1.08 (s, 3H), 0.93 (s, 3H).
^{13}C NMR (101 MHz, $CDCl_3$): δ 205.6, 168.9, 155.9, 155.6, 136.9, 133.9, 128.6, 127.9, 127.0, 116.9, 114.7, 101.3, 95.9, 77.5, 76.6, 70.5, 55.5, 52.2, 46.6, 46.1, 45.4, 44.3, 39.4, 39.0, 33.8, 31.6, 29.5, 27.5, 20.9, 20.3, 19.2, 18.9, 18.2.
$[\alpha]_D^{23}$ = + 43.9 (*c* 1.0, $CHCl_3$).
HRMS-QTOF (m/z): $[M + H]^+$ calculated for $C_{35}H_{47}O_7^+$, 579.3316; found 579.3324.

*Synthesis of the lactones **38** and **S22**:*

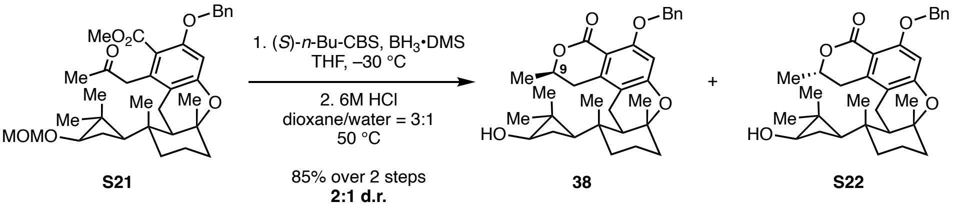

(*S*)-2-Butyl-CBS-oxazaborolidine (0.23 mL, 0.3 M in toluene, 0.069 mmol, 2.0 equiv.) was added to a solution of ketone **S21** (20.0 mg, 0.035 mmol, 1.0 equiv.) in tetrahydrofuran (4.0 mL). The flask was placed in an ethanol bath and cooled to −30 °C. Borane dimethylsulfide (34.6 µL, 2.0 M in tetrahydrofuran, 0.069 mmol, 2.0 equiv.) was added dropwise. The reaction was stirred at −30 °C for 6 h. Upon complete conversion of ketone **S21**, the reaction was diluted with water (20 mL), transferred to a separatory funnel, and extracted with ethyl acetate (3 × 5 mL). The combined organic layers were washed with brine (10 mL) and dried over anhydrous sodium sulfate. The dried solution was filtered, and the filtrate was concentrated. The crude alcohols mixture obtained was used directly in the next reaction without further purification.

Hydrochloric acid (0.29 mL, 6.0 M aqueous solution, 1.73 mmol, 50.0 equiv.) was added to a solution of crude alcohols in 1,4-dioxane-water (3.5 mL, *v*/*v* = 3:1). The flask was placed in an oil bath preheated to 50 °C and stirred overnight. Upon complete conversion of the alcohol intermediates, the reaction was cooled to 23 °C and diluted with water (20 mL). The mixture was transferred to a separatory funnel and extracted with ethyl acetate (3 × 5 mL). The combined organic layers were washed with brine (5 mL) and dried over anhydrous sodium sulfate. The dried solution was filtered, and the filtrate was concentrated. The residue obtained was purified by flash column chromatography (eluting with 40% ethyl acetate-hexanes) to afford **38** (9.9 mg, 57% over 2 steps) and **S22** (4.9 mg, 28%) as white foams.

Data of **38**: See page S42.
Data of **S22**: R_f = 0.3 (50% ethyl acetate-hexanes, UV & CAM stain).
^{1}H NMR (400 MHz, $CDCl_3$): δ 7.54 (d, *J* = 7.2 Hz, 2H), 7.36 (t, *J* = 7.5 Hz, 2H), 7.30 – 7.27 (m, 1H), 6.35 (s, 1H), 5.18 (d, *J* = 12.5 Hz, 1H), 5.13 (d, *J* = 12.6 Hz, 1H), 4.50 (dqd, *J* = 12.5, 6.2, 3.2 Hz, 1H), 3.69 – 3.61 (m, 1H), 2.80 (dd, *J* = 16.3, 3.1 Hz, 1H), 2.70 (dd, *J* = 16.3, 11.6 Hz, 1H), 2.43 – 2.23 (m, 2H), 2.08 – 1.98 (m, 1H), 1.97 – 1.89 (m, 1H), 1.73 (d, *J* = 7.4 Hz, 2H), 1.65 (d, *J* = 2.6 Hz, 2H), 1.55 – 1.45 (m, 7H), 1.21 (s, 3H), 1.20 (s, 3H), 1.07 (s, 3H), 0.98 (s, 3H).
^{13}C NMR (151 MHz, $CDCl_3$): δ 163.0, 160.4, 158.3, 142.1, 136.9, 128.6, 127.7, 126.8, 111.5, 107.3, 101.5, 78.2, 72.8, 72.0, 70.5, 46.7, 46.3, 44.2, 39.4, 39.1, 33.9, 33.2, 31.1, 29.9, 21.1, 20.6, 20.5, 19.2, 19.1, 17.4.
$[\alpha]_D^{24}$ = + 37.9 (*c* 0.2, $CHCl_3$).
HRMS-QTOF (m/z): $[M + H]^+$ calculated for $C_{32}H_{41}O_5^+$, 505.2949; found 505.2964.
Note: The relative stereochemistry at the C9 position was established by conversion of **38** to **4** and by obtaining its X-ray crystal structure (see page S59).

Synthesis of Talaromyolide D (4):

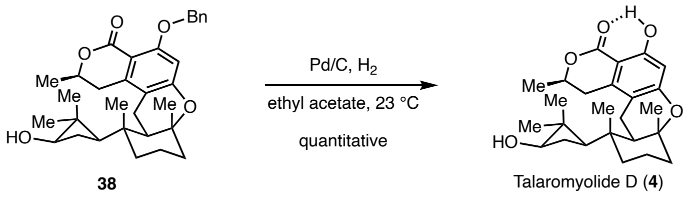

Lactone **38** (8.6 mg, 0.017 mmol, 1.0 equiv.) and palladium on carbon (1.8 mg, 10 wt.% on carbon, 0.0017 mmol, 0.1 equiv.) were added to a 10 mL reaction tube. Ethyl acetate (1.0 mL) was added. The reaction was then purged with hydrogen and stirred under a 1 atm hydrogen atmosphere at 23 °C for 8 h. Upon complete conversion of **38**, the reaction mixture was filtered through a short pad of celite. The filtrate was concentrated. The residue obtained was purified by preparative thin layer chromatography (developing in 50% ethyl acetate-hexanes) to afford talaromyolide D (**4**) as a white solid (7.0 mg, 100%).

R_f = 0.4 (50% ethyl acetate-hexanes, UV & CAM stain).
^{1}H NMR (400 MHz, $CDCl_3$): δ 11.11 (s, 1H), 6.27 (s, 1H), 4.68 (dqd, *J* = 12.5, 6.2, 3.3 Hz, 1H), 3.69 – 3.61 (m, 1H), 2.97 (dd, *J* = 16.7, 3.5 Hz, 1H), 2.64 (dd, *J* = 16.7, 11.3 Hz, 1H), 2.48 (dd, *J* = 15.9, 5.1 Hz, 1H), 2.19 (dd, *J* = 15.9, 13.1 Hz, 1H), 2.07 – 2.01 (m, 1H), 1.96 – 1.90 (m, 1H), 1.78 – 1.70 (m, 2H), 1.66 (d, *J* = 4.7 Hz, 2H), 1.57 (d, *J* = 6.3 Hz, 3H), 1.55 – 1.49 (m, 4H), 1.20 (s, 3H), 1.18 (s, 3H), 1.08 (s, 3H), 0.96 (s, 3H).
^{13}C NMR (151 MHz, $CDCl_3$): δ 170.2, 162.4, 160.6, 139.0, 111.6, 103.5, 101.8, 78.4, 74.7, 72.0, 46.7, 46.1, 44.1, 39.2, 39.0, 33.8, 31.5, 31.1, 30.0, 21.1, 20.5, 20.3, 19.2, 19.1, 17.4.
$[\alpha]_D^{24}$ = + 95.0 (*c* 0.2, $CHCl_3$).
HRMS-QTOF (m/z): $[M + H]^+$ calculated for $C_{25}H_{35}O_5^+$, 415.2479; found 415.2483.

*Synthesis of the diazo ketone **S24**:*

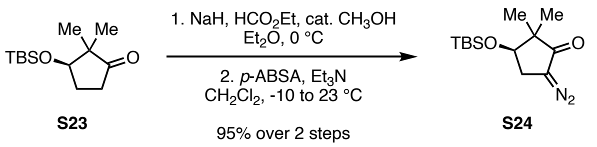

Ketone **S23** was prepared according to procedure reported by Han.[11] Sodium hydride (396.0 mg, 60% dispersion in mineral oil, 9.9 mmol, 1.2 equiv.) was suspended in dry diethyl ether (3.9 mL). The flask was then placed in an ice bath and cooled to 0 °C. Ethyl formate (1.5 mL, 19.0 mmol, 2.3 equiv.) and ketone **S23** (2.0g, 8.25 mmol, 1.0 equiv.) were dissolved in 2.0 mL of diethyl ether and methanol (10 µL, 0.2 mmol, 0.03 equiv.). This solution was subsequently added to the reaction mixture in a dropwise manner at 0 °C. The reaction was stirred at 0 °C for 2 h. Upon complete conversion of **S23**, the reaction mixture became a white solid. The solid was dissolved in water (100 mL), transferred to a separatory funnel, and washed with diethyl ether (3 × 50 mL). The water phase was acidified to pH~3 with 1 M HCl and extracted with diethyl ether (5 × 50 mL). The combined organic layers were washed with brine (50 mL) and dried over anhydrous sodium sulfate. The dried solution was filtered, and the filtrate was concentrated. The crude aldehyde obtained was used directly in the next reaction without further purification.

The residue obtained in the previous step was dissolved in dichloromethane (17 mL) and cooled to −10 °C. Triethylamine (5.8 mL, 41.2 mmol, 5.0 equiv.) and 4-acetamidobenzenesulfonyl azide (2.0 g, 8.25 mmol, 1.0 equiv.) were sequentially added. The reaction mixture was allowed to warm gradually to 23 °C and stirred overnight. The product mixture was then cooled to 0 °C. 4 M KOH (6.8 mL) was added. The reaction mixture was stirred for 10 min at 0 °C. The reaction was then diluted with water (100 mL), transferred to a separatory funnel, and extracted with dichloromethane (3 × 50 mL). The combined organic layers were washed with brine (50 mL) and dried over anhydrous sodium sulfate. The dried solution was filtered, and the filtrate was concentrated. The residue obtained was purified by flash column chromatography (eluting with 15% ethyl acetate-hexanes) to afford **S24** (2.1 g, 95% over 2 steps) as a yellow oil.

R_f = 0.6 (25% ethyl acetate-hexanes, UV & CAM stain).
^{1}H NMR (400 MHz, $CDCl_3$): δ 3.96 (t, *J* = 6.5 Hz, 1H), 3.14 (dd, *J* = 12.9, 6.7 Hz, 1H), 2.78 (dd, *J* = 12.9, 6.4 Hz, 1H), 1.07 (s, 3H), 1.01 (s, 3H), 0.90 (s, 9H), 0.09 (s, 3H), 0.08 (s, 3H).
^{13}C NMR (101 MHz, $CDCl_3$): δ 202.5, 76.0, 55.4, 50.8, 31.5, 25.8, 22.3, 18.2, 17.8, -4.4, -4.8.
HRMS-QTOF (m/z): $[M + H]^+$ calculated for $C_{13}H_{25}N_2O_2Si^+$, 269.1680; found 269.1689.

*Synthesis of NHPI esters **S25 and** C24-epi-**S25**:*

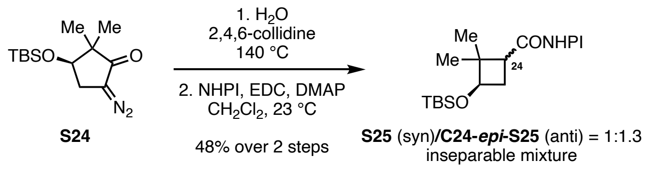

Note: We found that the Wolff rearrangement exhibited diminished efficiency on scale above 100 mg. Accordingly, 28 parallel reactions were conducted at a 50 mg scale to obtain sufficient quantities of the desired material.

Water (30.0 μL, 1.7 mmol, 9.0 equiv.) was added to a solution of diazo **S24** (50 mg, 0.19 mmol, 1.0 equiv.) in 2,4,6-collidine (0.2 mL). The reaction tube was then sealed and placed in a heating block preheated to 140 °C and stirred for 24 h. Upon complete conversion of diazo **S24**, the 28 parallel reaction mixtures were combined, diluted with ethyl acetate (100 mL), and transferred to a separatory funnel. The ethyl acetate solution was washed with 1 M HCl (3 × 50 mL) and brine (50 mL). The organic layer was dried over anhydrous sodium sulfate. The dried solution was filtered, and the filtrate was concentrated. The crude acid obtained was used directly in the next reaction without further purification. *N*-Hydroxyphthalimide (765.7 mg, 4.7 mmol, 0.9 equiv.), 4-dimethylaminopyridine (95.6 mg, 0.78 mmol, 0.15 equiv.), and 1-ethyl-3-(3-dimethylaminopropyl)carbodiimide hydrochloride (1.1 g, 5.7 mmol, 1.1 equiv.) were sequentially added to a solution of the above crude acid in dichloromethane (26 ml). The flask was then covered with foil and stirred at 23 °C for 48 h. Upon complete conversion of the acid intermediate, the reaction mixture was transferred to a separatory funnel and washed sequentially with water (3 × 30 mL), 1 M sodium hydroxide (50 mL), and brine (30 mL). The organic layer was dried over anhydrous sodium sulfate. The dried solution was filtered, and the filtrate was concentrated. The residue obtained was purified by flash column chromatography (eluting with 5% ethyl acetate-hexanes) to provide NHPI esters **S25** and C24-*epi*-**S25** (1.0 g, 1:1.3 inseparable mixture, 48% over 2 steps) as white solid.

Data of **S25**: R_f = 0.6 (20% ethyl acetate-hexanes, UV & CAM stain).
^{1}H NMR (400 MHz, $CDCl_3$): δ 7.92 – 7.87 (m, 2H), 7.83 – 7.78 (m, 2H), 3.91 (dd, *J* = 8.6, 7.2 Hz, 1H), 2.70 (dd, *J* = 10.7, 7.5 Hz, 1H), 2.40 – 2.20 (m, 2H), 1.30 (s, 3H), 1.16 (s, 3H), 0.88 (s, 9H), 0.04 (s, 3H), 0.04 (s, 3H).
^{13}C NMR (101 MHz, $CDCl_3$): δ 168.8, 162.2, 134.8, 129.2, 124.0, 71.6, 48.9, 38.7, 30.4, 28.2, 25.9, 18.2, 16.4, -4.6, -4.6.
HRMS-QTOF (m/z): $[M + H]^+$ calculated for $C_{21}H_{30}NO_6Si^+$, 404.1888; found 404.1901.

Data of C24-*epi*-**S25**: R_f = 0.6 (20% ethyl acetate-hexanes, UV & CAM stain).
^{1}H NMR (400 MHz, $CDCl_3$): δ 7.92 – 7.87 (m, 2H), 7.83 – 7.78 (m, 2H), 4.14 – 4.06 (m, 1H), 3.03 (ddd, *J* = 9.4, 4.3, 1.1 Hz, 1H), 2.58 (ddd, *J* = 11.9, 7.4, 4.3 Hz, 1H), 2.10 (ddd, *J* = 12.2, 9.4, 6.6 Hz, 1H), 1.25 (s, 3H), 1.24 (s, 3H), 0.88 (s, 9H), 0.03 (s, 3H), 0.02 (s, 3H).
^{13}C NMR (101 MHz, $CDCl_3$): δ 171.0, 162.2, 134.8, 129.1, 124.0, 72.3, 47.2, 40.9, 30.6, 25.9, 23.8, 22.9, 18.2, -4.7.
HRMS-QTOF (m/z): $[M + H]^+$ calculated for $C_{21}H_{30}NO_6Si^+$, 404.1888; found 404.1900.

*Synthesis of NHPI esters **16** and C24-epi-**16**:*

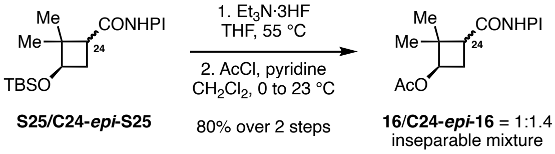

S25/C24-*epi*-S25

16/C24-*epi*-16 = 1:1.4
inseparable mixture

Triethylamine trihydrofluoride (1.4 mL, 8.7 mmol, 5.0 equiv.) was added to a solution of NHPI esters **S25**/C24-*epi*-**S25** (700 mg, 1.7 mmol, 1.0 equiv.) in tetrahydrofuran (17 mL). The plastic tube was placed in an oil bath preheated to 55 °C and stirred overnight. Upon complete conversion of **S25**/C24-*epi*-**S25**, the reaction mixture was diluted with saturated aqueous sodium bicarbonate (50 mL), transferred to a separatory funnel, and extracted with ethyl acetate (3 × 30 mL). The combined organic layers were washed with brine (30 mL) and dried over anhydrous sodium sulfate. The dried solution was filtered, and the filtrate was concentrated. The crude alcohol obtained was used directly in the next reaction without further purification.

Pyridine (2.1 mL, 26.0 mmol, 15.0 equiv.) was added to a solution of the residue obtained in the previous step dissolved in dichloromethane (35 mL). The flask was placed in an ice bath and cooled to 0 °C. Acyl chloride (1.2 mL, 17.4 mmol, 10.0 equiv.) was added dropwise. The reaction was then allowed to warm gradually to 23 °C and stirred for 5 h. Upon complete conversion of the alcohol, the reaction mixture was diluted with saturated aqueous sodium bicarbonate (50 mL), transferred to a separatory funnel, and extracted with dichloromethane (3 × 30 mL). The combined organic layers were washed with brine (50 mL) and dried over anhydrous sodium sulfate. The dried solution was filtered, and the filtrate was concentrated. The residue obtained was purified by flash column chromatography (eluting with 10% ethyl acetate-hexanes) to provide NHPI esters **16** and C24-*epi*-**16** (462 mg, 1:1.4 inseparable mixture, 80% over 2 steps) as white solid.

Data of **16**: See page S18.
Data of C24-*epi*-**16**: R_f = 0.5 (30% ethyl acetate-hexanes, UV & CAM stain).
^{1}H NMR (400 MHz, $CDCl_3$): δ 7.93 – 7.88 (m, 2H), 7.84 – 7.79 (m, 2H), 4.91 (ddd, *J* = 7.8, 5.8, 1.1 Hz, 1H), 3.17 (ddd, *J* = 9.6, 5.5, 1.1 Hz, 1H), 2.76 (ddd, *J* = 13.3, 7.6, 5.4 Hz, 1H), 2.27 (ddd, *J* = 13.1, 9.6, 5.8 Hz, 1H), 2.08 (s, 3H), 1.36 (s, 3H), 1.28 (s, 3H).
^{13}C NMR (101 MHz, $CDCl_3$): δ 170.7, 169.9, 162.0, 134.9, 129.1, 124.1, 74.1, 45.7, 41.7, 26.4, 23.4, 23.1, 20.9.
HRMS-QTOF (m/z): $[M + Na]^+$ calculated for $C_{17}H_{17}NNaO_6^+$, 354.0948; found 354.0946.

Comparison of Spectroscopic Data of Isolated and Synthetic talaromyolide D (4)

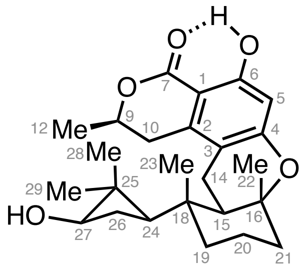

Talaromyolide D (**4**)

Table S14. Comparison of ^{1}H NMR data of isolated and synthetic talaromyolide D (**4**)

Position	isolated **4** ($CDCl_3$, 600 MHz) ^{1}H [δ H (ppm), mult., *J* (Hz)]	synthetic **4** ($CDCl_3$, 400 MHz) ^{1}H [δ H (ppm), mult., *J* (Hz)]	Δδ (ppm)
H5	6.28 (s)	6.27 (s)	0.01
H9	4.63 (m)	4.68 (dqd, 12.5, 6.2, 3.3)	0.05
H10	2.61 (dd, 16.8, 11.5)	2.64 (dd, 16.7, 11.3)	0.03
	2.92 (dd, 16.7, 3.5)	2.97 (dd, 16.7, 3.5)	0.05
H12	1.53 (d, 5.3)	1.57 (d, 6.3)	0.04
H14	2.24 (m)	2.19 (dd, 15.9, 13.1)	0.05
	2.50 (dd, 15.6, 5.5)	2.48 (dd, 15.9, 5.1)	0.02
H15	1.59 (m)	1.55 (m)	0.04
H19	1.44 (m)	1.50 (m)	0.06
	1.81 (m)	1.73 (m)	0.08
H20	1.15 (m)	1.50 (m)	0.35
	1.79 (m)	1.73 (m)	0.06
H21	1.67 (m)	1.52 (m)	0.15
	2.10 (m)	1.94 (m)	0.16
H22	1.18 (s)	1.18 (s)	0.00
H23	0.91 (s)	0.96 (s)	0.05
H24	1.01 (m)	1.66 (m)	0.65
H26	1.66 (m)	1.66 (m)	0.00
	1.73 (m)	2.03	0.30
H27	3.26 (dd, 11.6, 4.7)	3.65 (m)	0.39
H28	1.04 (s)	1.08 (s)	0.04
H29	0.83 (s)	1.20 (s)	0.37

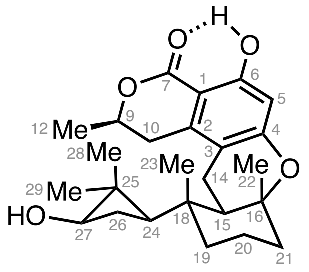

Talaromyolide D (**4**)

Table S15. Comparison of ^{13}C NMR data of isolated and synthetic talaromyolide D.

Position	isolated **4** ($CDCl_3$, 151 MHz) ^{13}C [δ C (ppm)]	synthetic **4** ($CDCl_3$, 151 MHz) ^{13}C [δ C (ppm)]	Δδ (ppm)
C1	101.6	101.8	0.2
C2	139.0	139.0	0.0
C3	110.9	111.6	0.7
C4	160.5	160.6	0.1
C5	103.3	103.5	0.2
C6	162.2	162.4	0.2
C7	170.1	170.2	0.1
C9	74.6	74.7	0.1
C10	31.4	31.5	0.1
C12	20.9	21.1	0.2
C14	19.3	20.3	1.0
C15	51.6	44.1	7.5
C16	77.9	78.4	0.5
C18	36.7	39.1	2.4
C19	19.42	33.8	3.7
C20	37.5	19.2	0.22
C21	40.7	39.2	1.5
C22	20.8	20.5	0.3
C23	14.9	19.2	4.3
C24	55.0	46.1	8.9
C25	38.8	46.7	7.9
C26	27.11	30.0	2.89
C27	78.6	72.3	6.3
C28	28.1	31.1	1.9
C29	15.5	17.4	3.0

Figure S4. Comparison of ^{1}H NMR spectra of isolated and synthetic talaromyolide D (**4**).

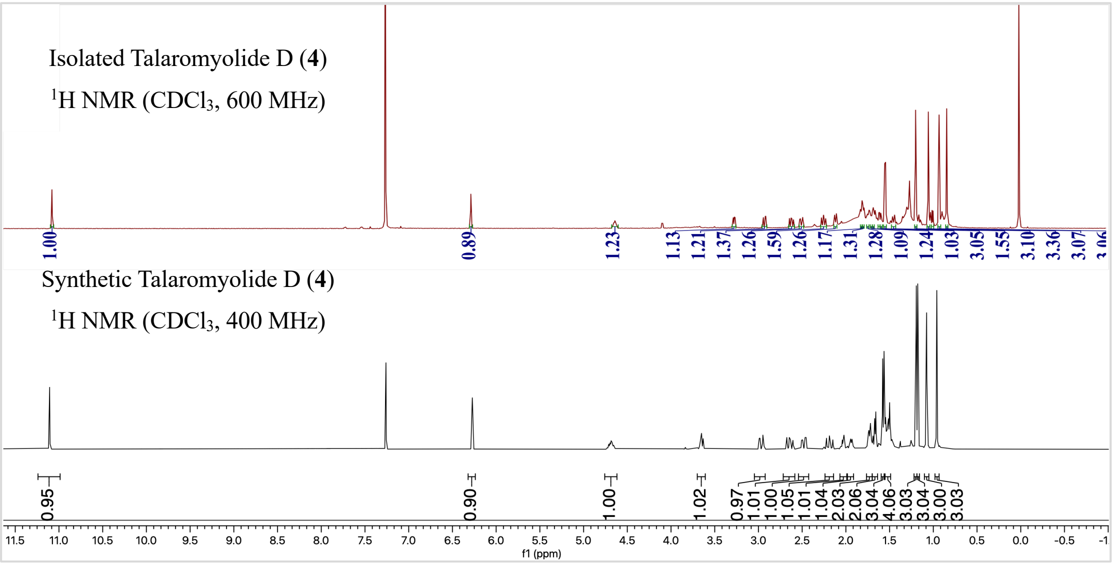

Figure S5. Comparison of ^{1}H NMR spectra of isolated and synthetic talaromyolide D (**4**).

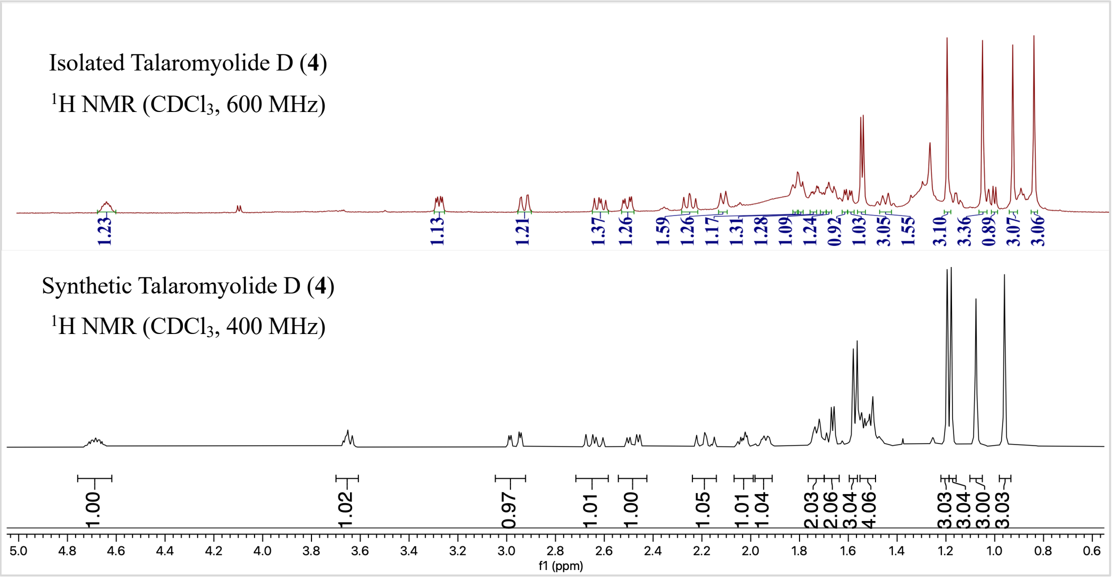

Figure S6. Comparison of ^{13}C NMR spectra of isaolated and synthetic talaromyolide D (**4**).

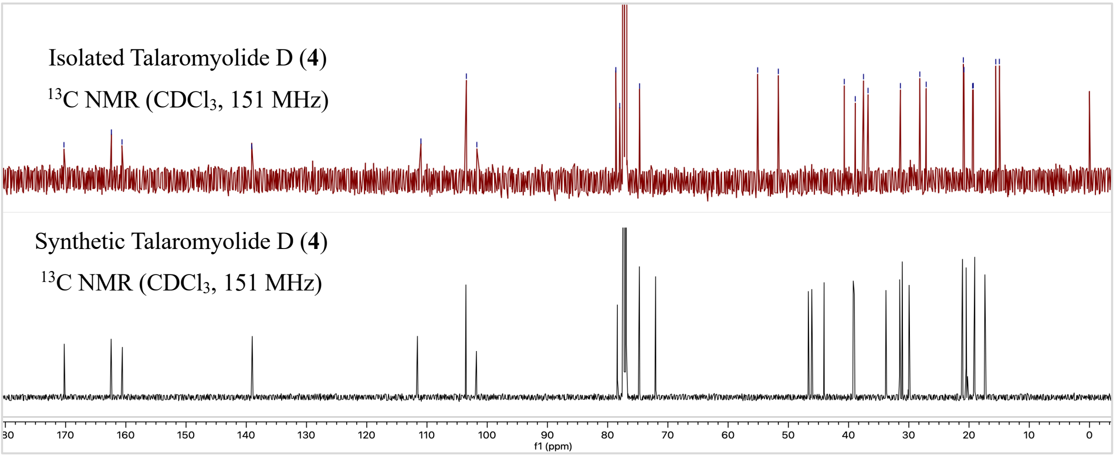

Crystallographic Analysis of Alcohol 21.

Single crystals of alcohol **21** suitable for X-ray analysis were obtained by the slow evaporation of a solution of compound **21** in hexanes-dichloromethane (10:1, *v*/*v*) at 23 °C. Crystals were mounted on a MiTeGen MicroMount with Type B immersion oil (Cargille Labs). Single crystal X-ray intensity data were measured on a Bruker D8 SMART APEXII "three-circle diffractometer" system equipped with a Incoatec "microfocus sealed X-ray tube" (MoK α radiation, λ = 0.71073 Å), a multilayer optics monochromator and a PHOTON-II-C14 detector. Crystal temperature was controlled by an Oxford Cryosystems 700 + Cooler. Full datasets were collected with ω scans at T = 100(2) K. The frames were integrated with the Bruker SAINT software package using a narrow-frame algorithm and the data were corrected for absorption effects using the Multi-Scan method with the SADABS software. The structures were solved by intrinsic phasing methods (SHELXT) and the structure models were completed and refined using the full-matrix least-square methods on F2 (SHELXL). All non-hydrogen atoms were refined with anisotropic displacement parameters, and hydrogen atoms on carbons were placed in idealized positions (C-H = 0.95-1.00 Å) and included using a riding model with Uiso(H) = 1.2 or 1.5 Ueq(non-H). Selected crystallographic parameters are listed in **Table S16**. Crystallographic data of these structures, including cif, res, fcf, and hkl files, have been deposited with the Cambridge Crystallographic Data Centre with deposition number **2450768**. Copies of these data can be requested, free of charge, from the CCDC website at https://www.ccdc.cam.ac.uk/structures/.

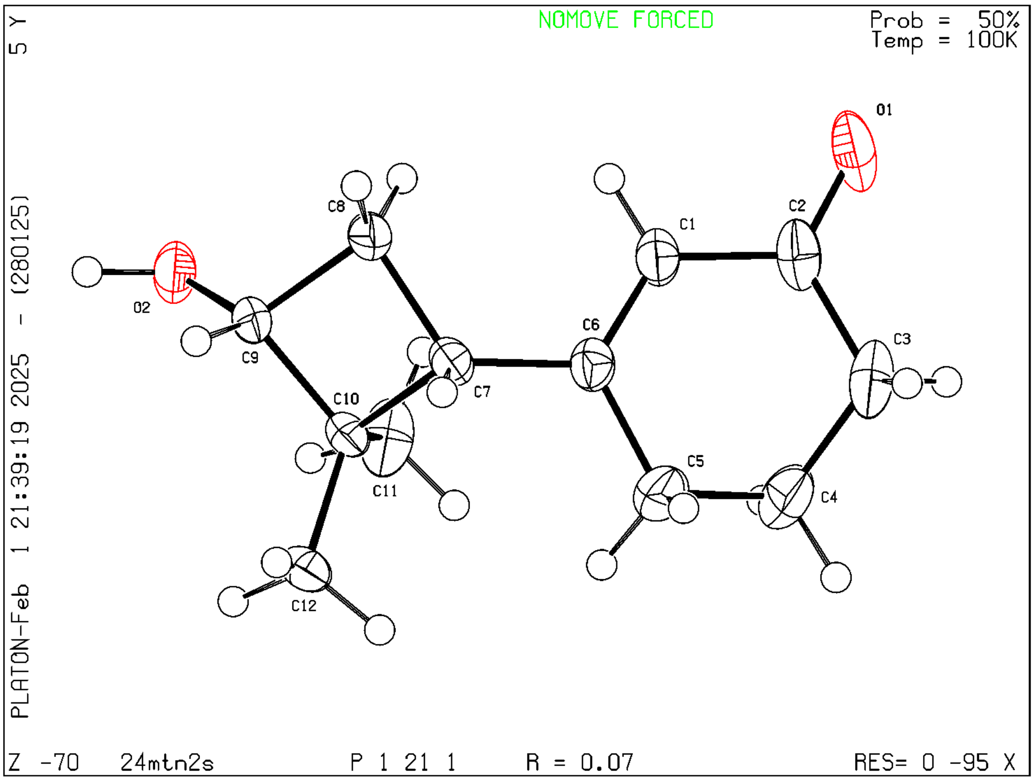

Figure S7. The crystal structure of alcohol **21**.

Table S16. Crystallographic data and structure refinement of the alcohol **21**.

Bond precision:	C−C = 0.0060 A	Wavelength=0.71073	
Cell:	a = 7.2793(11)	b = 8.9755(14)	c = 8.9544(14)
	alpha = 90	beta = 107.201(10)	gamma = 90
Temperature:	100 K		
	Calculated	Reported	
Volume	558.87(15)	558.87(15)	
Space group	P 21	P 1 21 1	
Hall group	P 2yb	P 2yb	
Moiety formula	C12 H18 O2	C12 H18 O2	
Sum formula	C12 H18 O2	C12 H18 O2	
Mr	194.26	194.26	
Dx, g cm−3	1.154	1.154	
Z	2	2	
Mu (mm−1)	0.077	0.077	
F000	212.0	212.0	
F000’	212.10		
h, k, lmax	9, 12, 12	9, 12, 11	
Nref	2811[1493]	2763	
Tmin, Tmax	0.984, 0.998	0.770, 1.000	
Tmin’	0.929		

Correction method = # Reported T Limits: Tmin = 0.770 Tmax = 1.000
AbsCorr = MULTI−SCAN

Data completeness = 1.85/0.98 Theta(max) = 28.490

R(reflections) = 0.0675(2027) wR2(reflections) = 0.1863(2763)
S = 1.087 Npar= 132

Crystallographic Analysis of Nitroarene 25.

Single crystals of nitroarene **25** suitable for X-ray analysis were obtained by the slow evaporation of a solution of compound **25** in hexanes-dichloromethane (10:1, *v*/*v*) at 23 °C. Crystals were mounted on a MiTeGen MicroMount with Type B immersion oil (Cargille Labs). Single crystal X-ray intensity data were measured on a Bruker D8 SMART APEXII "three-circle diffractometer" system equipped with a Incoatec "microfocus sealed X-ray tube" (MoK α radiation, λ = 0.71073 Å), a multilayer optics monochromator and a PHOTON-II-C14 detector. Crystal temperature was controlled by an Oxford Cryosystems 700 + Cooler. Full datasets were collected with ω scans at T = 100(2) K. The frames were integrated with the Bruker SAINT software package using a narrow-frame algorithm and the data were corrected for absorption effects using the Multi-Scan method with the SADABS software. The structures were solved by intrinsic phasing methods (SHELXT) and the structure models were completed and refined using the full-matrix least-square methods on F2 (SHELXL). All non-hydrogen atoms were refined with anisotropic displacement parameters, and hydrogen atoms on carbons were placed in idealized positions (C-H = 0.95-1.00 Å) and included using a riding model with Uiso(H) = 1.2 or 1.5 Ueq(non-H). Selected crystallographic parameters are listed in **Table S17**. Crystallographic data of these structures, including cif, res, fcf, and hkl files, have been deposited with the Cambridge Crystallographic Data Centre with deposition number **2450769**. Copies of these data can be requested, free of charge, from the CCDC website at https://www.ccdc.cam.ac.uk/structures/.

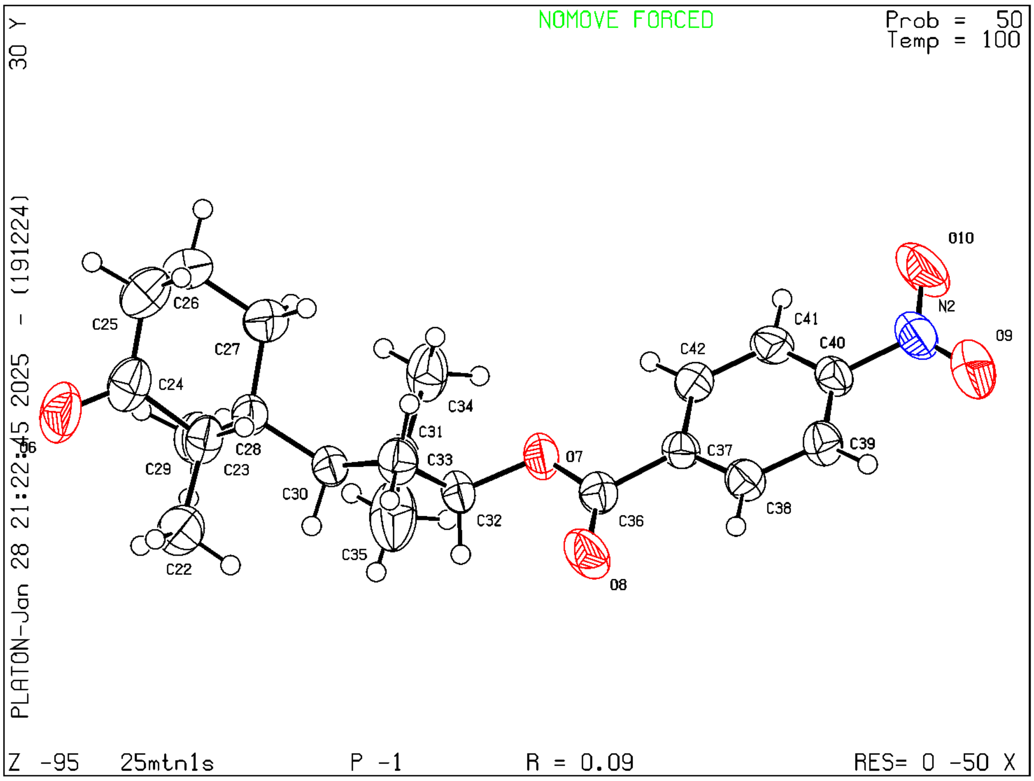

Figure S8. The crystal structure of nitroarene **25**.

Table S17. Crystallographic data and structure refinement of nitroarene **25**.

Bond precision:	C−C = 0.0051 A		Wavelength = 0.71073
Cell:	a = 7.4563(14)	b = 13.642(2)	c = 19.823(3)
	alpha = 85.045(7)	beta = 84.100(7)	gamma = 87.453(7)
Temperature:	100 K		

	Calculated	Reported
Volume	1996.9(6)	1996.9(6)
Space group	P −1	P −1
Hall group	−P 1	−P 1
Moiety formula	C21 H27 N O5	C21 H27 N O5
Sum formula	C21 H27 N O5	C21 H27 N O5
Mr	373.44	373.45
Dx, g cm−3	0.621	1.242
Z	2	4
Mu (mm−1)	0.044	0.088
F000	400.0	800.5
F000’	400.20	
h, k, lmax	9, 17, 24	9, 17, 24
Nref	8347	8244
Tmin, Tmax	0.992, 0.997	0.610, 1.000
Tmin’	0.965	

Correction method = # Reported T Limits: Tmin = 0.610 Tmax = 1.000
AbsCorr = MULTI−SCAN

Data completeness = 0.988 Theta(max) = 26.580

R(reflections) = 0.0905(4898) wR2(reflections) = 0.1712(8244)
S = 1.050 Npar= 495

Crystallographic Analysis of Talaromyolide D (4).

Single crystals of talaromyolide D (**4**) suitable for X-ray analysis were obtained by the slow evaporation of a solution of compound **4** in hexanes-dichloromethane (10:1, *v*/*v*) at 23 °C. Crystals were mounted on a MiTeGen MicroMount with Type B immersion oil (Cargille Labs). Single crystal X-ray intensity data were measured on a Bruker D8 SMART APEXII "three-circle diffractometer" system equipped with a Incoatec "microfocus sealed X-ray tube" (MoK α radiation, λ = 0.71073 Å), a multilayer optics monochromator and a PHOTON-II-C14 detector. Crystal temperature was controlled by an Oxford Cryosystems 700 + Cooler. Full datasets were collected with ω scans at T = 100(2) K. The frames were integrated with the Bruker SAINT software package using a narrow-frame algorithm and the data were corrected for absorption effects using the Multi-Scan method with the SADABS software. The structures were solved by intrinsic phasing methods (SHELXT) and the structure models were completed and refined using the full-matrix least-square methods on F2 (SHELXL). All non-hydrogen atoms were refined with anisotropic displacement parameters, and hydrogen atoms on carbons were placed in idealized positions (C-H = 0.95-1.00 Å) and included using a riding model with Uiso(H) = 1.2 or 1.5 Ueq(non-H). Selected crystallographic parameters are listed in **Table S18**. Crystallographic data of these structures, including cif, res, fcf, and hkl files, have been deposited with the Cambridge Crystallographic Data Centre with deposition number **2450770**. Copies of these data can be requested, free of charge, from the CCDC website at https://www.ccdc.cam.ac.uk/structures/.

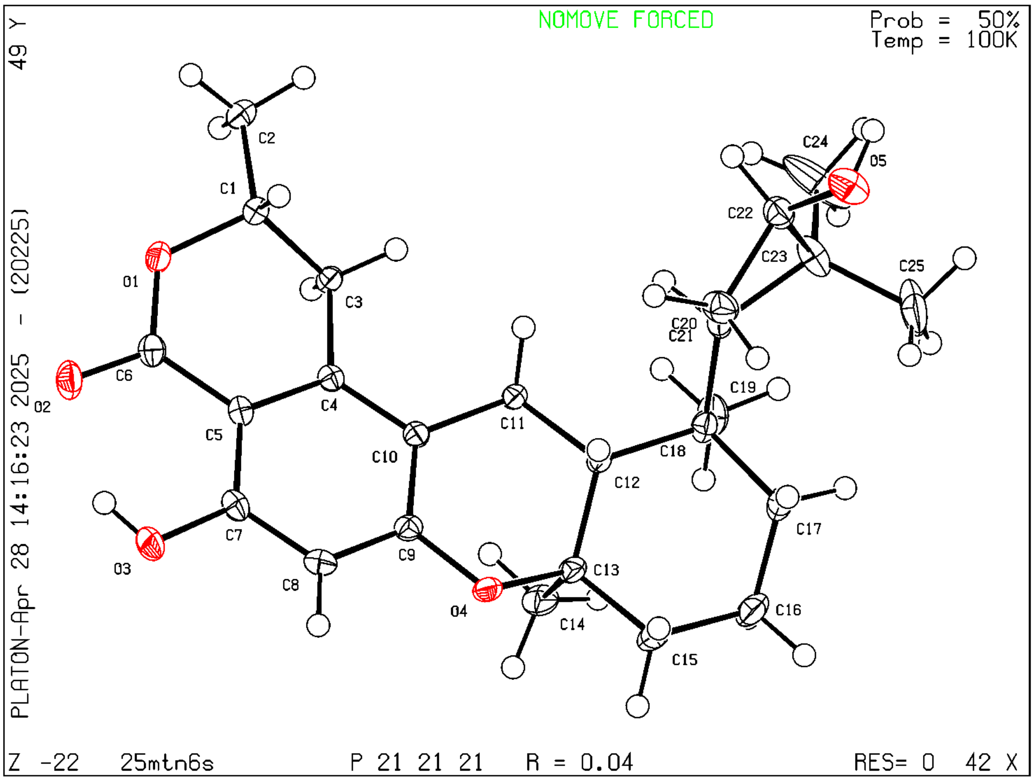

Figure S9. The crystal structure of Talaromyolide D (**4**).

Table **S18**. Crystallographic data and structure refinement of Talaromyolide D (**4**).

Bond precision:	C–C = 0.0031 A		Wavelength = 0.71073
Cell:	a = 8.094(3)	b = 15.556(9)	c = 17.297(10)
	alpha = 90	beta = 90	gamma = 90
Temperature:	100 K		

	Calculated	Reported
Volume	2178(2)	2178(2)
Space group	P 21 21 21	P 21 21 21
Hall group	P 2ac 2ab	P 2ac 2ab
Moiety formula	C25 H34 O5	?
Sum formula	C25 H34 O5	C25 H34 O5
Mr	414.52	414.52
Dx, g cm−3	1.264	1.264
Z	4	4
Mu (mm−1)	0.087	0.087
F000	896.0	896.0
F000'	896.44	
h, k, lmax	10, 20, 23	10, 20, 23
Nref	5497[3111]	5480
Tmin, Tmax	0.968, 0.978	0.910, 0.980
Tmin'	0.964	

Correction method = # Reported T Limits: Tmin = 0.910 Tmax = 0.980
AbsCorr = MULTI−SCAN

Data completeness = 1.76/1.00 Theta(max) = 28.470

R(reflections) = 0.0397(5349) wR2(reflections) = 0.0998(5480)
S = 1.134 Npar= 278

References.

[1] Vuagnoux-d'Augustin, M.; Alexakis, A. Copper-catalyzed asymmetric conjugate addition of trialkylaluminium reagents to trisubstituted enones: Construction of chiral quaternary centers. *Chem. Eur. J.* **2007**, *13*, 9647-9662.

[2] Weitz, D. J.; Bednarski, M. D. Synthesis of Acyclic Sugar Aldehydes by Ozonolysis of. Oximes. *J. Org. Chem.* **1989**, *54*, 4957-4959.

[3] Göttemann, L. T.; Wiesler, S.; Sarpong, R. Oxidative cleavage of ketoximes to ketones. using photoexcited nitroarenes. *Chem. Sci.* **2023**, *15*, 213-219.

[4] Curran, D. P. Reduction of Delta-2-Isoxazolines .3. Raney-Nickel Catalyzed Formation of. Beta-Hydroxy Ketones. *J. Am. Chem. Soc.* **1983**, *105*, 5826-5833.

[5] Jiang, D. H.; Chen, Y. W. Reduction of Δ-Isoxazolines to β-Hydroxy Ketones with Iron and. Ammonium Chloride as Reducing Agent. *J. Org. Chem.* **2008**, *73*, 9181-9183

[6] Tranmer, G. K.; Tam, W. Molybdenum-mediated cleavage reactions of isoxazoline rings. fused in bicyclic frameworks. *Org. Lett.* **2002**, *4*, 4101-4104.

[7] Hehre, W.; Klunzinger, P.; Deppmeier, B.; Driessen, A.; Uchida, N.; Hashimoto, M.; Fukushi, E.; Takata, Y. Efficient Protocol for Accurately Calculating C Chemical Shifts of Conformationally Flexible Natural Products: Scope, Assessment, and. Limitations. *J. Nat. Prod.* **2019**, *82*, 2299-2306.

[8] Lan, D.; Wu, B. Chemistry and Bioactivities of Secondary Metabolites from the Genus Talaromyces. *Chem. Biodivers.* **2020**, *17*, e2000229.

[9] Harwood, S. J.; Palkowitz, M. D.; Gannett, C. N.; Perez, P.; Yao, Z.; Sun, L. J.; Abruña, H. D.; Anderson, S. L.; Baran, P. S. Modular terpene synthesis enabled by mild electrochemical couplings. *Science* **2022**, *375*, 745-752.

[10] Arndt, F. Diazomethane. *Org. Synth.* **1935**, *15*, 3.

[11] Wu, G. J.; Zhang, Y. H.; Tan, D. X.; Han, F. S. Total synthesis of cyrneines A-B and glaucopine C. *Nat. Commun.* **2018**, *9:* 2148.

Catalogue of ^{1}H NMR and ^{13}C NMR Spectra.

^{1}H NMR (400 MHz, $CDCl_3$) of **S10**

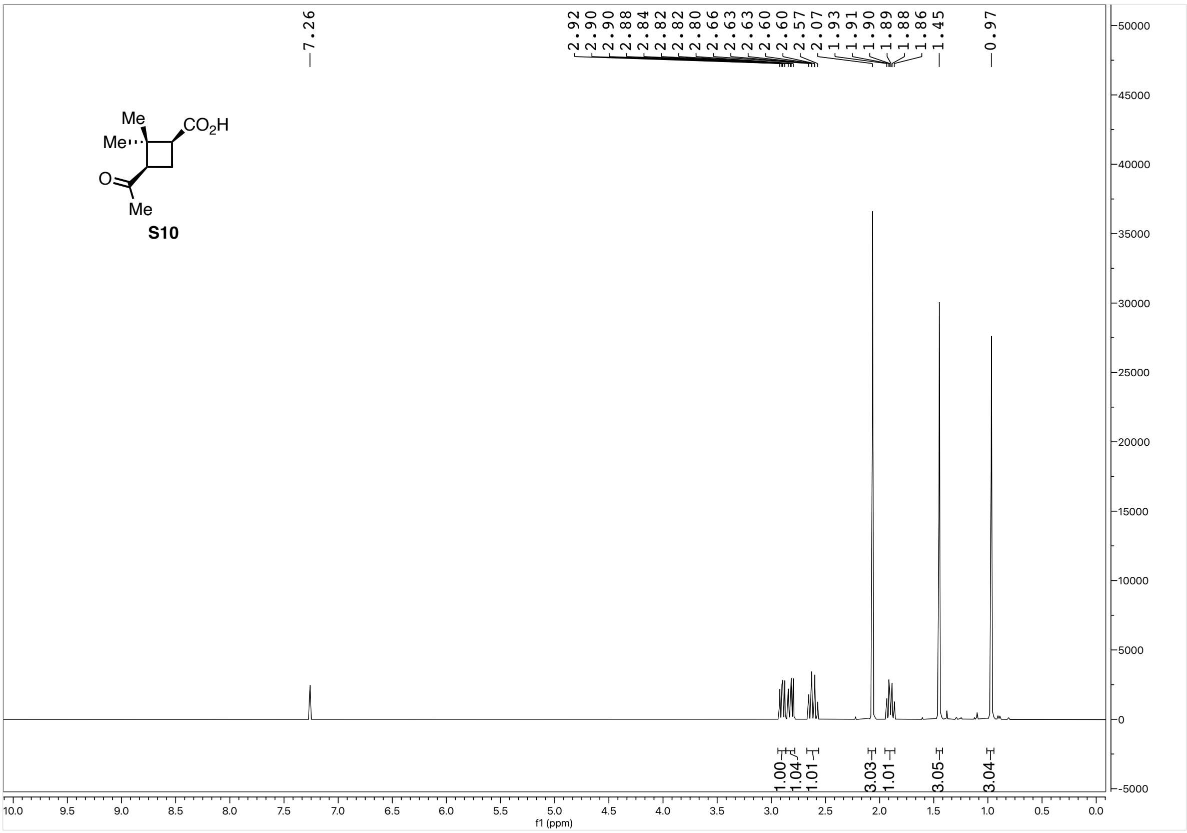

^{13}C NMR (101 MHz, $CDCl_3$) of **S10**

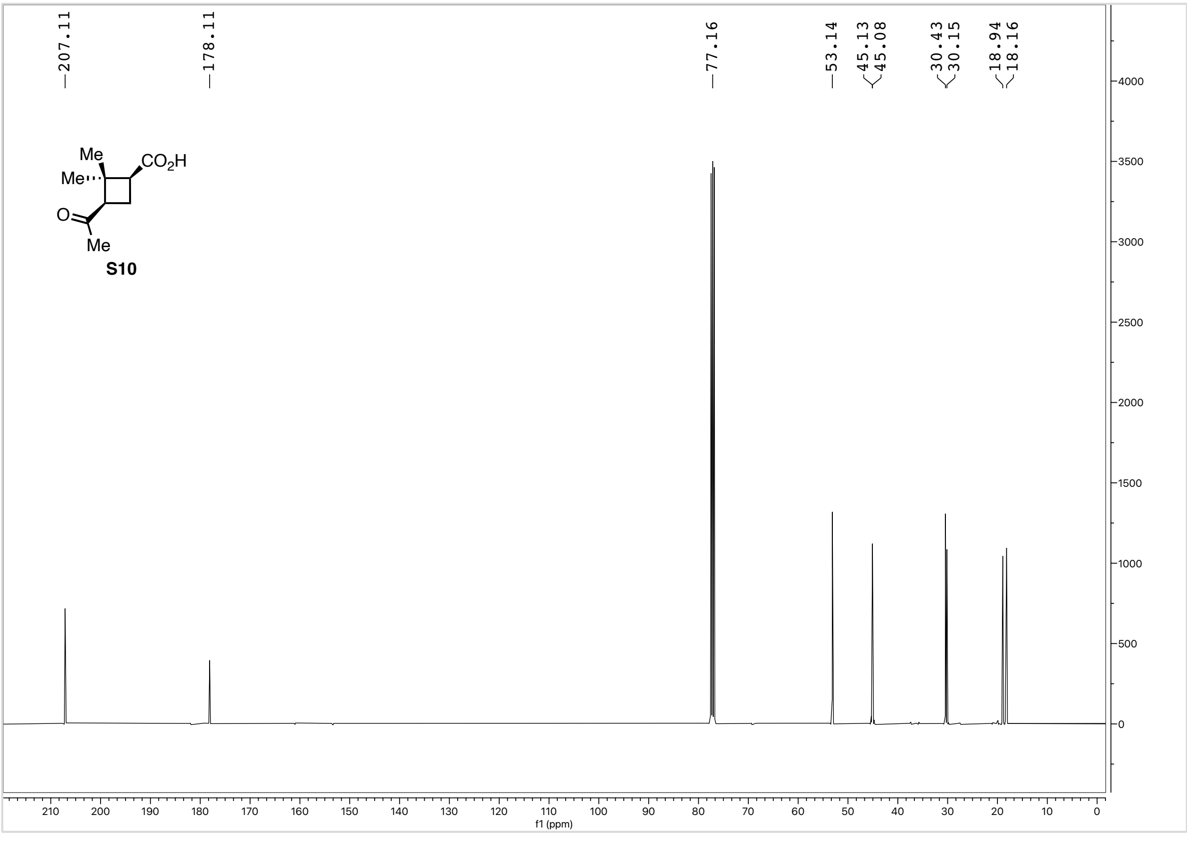

^{1}H NMR (400 MHz, $CDCl_3$) of **S11**

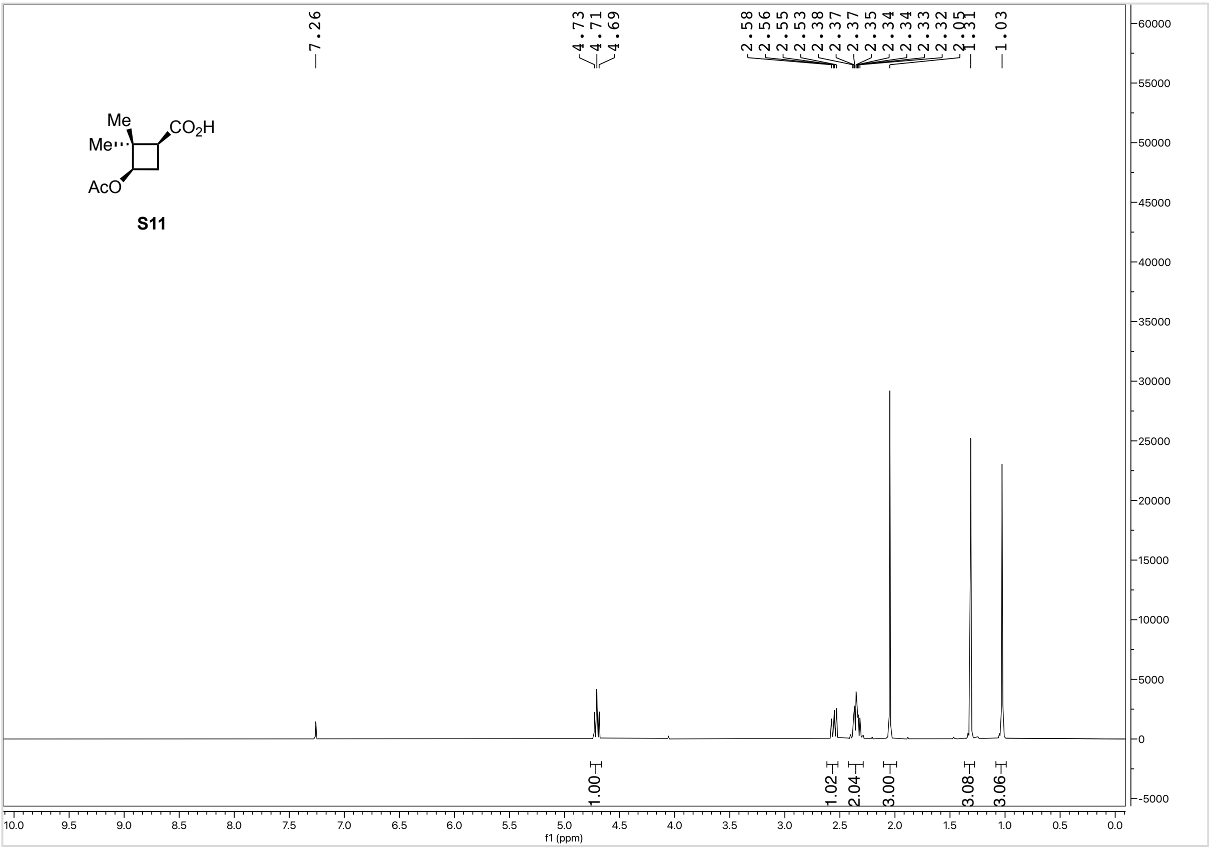

^{13}C NMR (101 MHz, $CDCl_3$) of **S11**

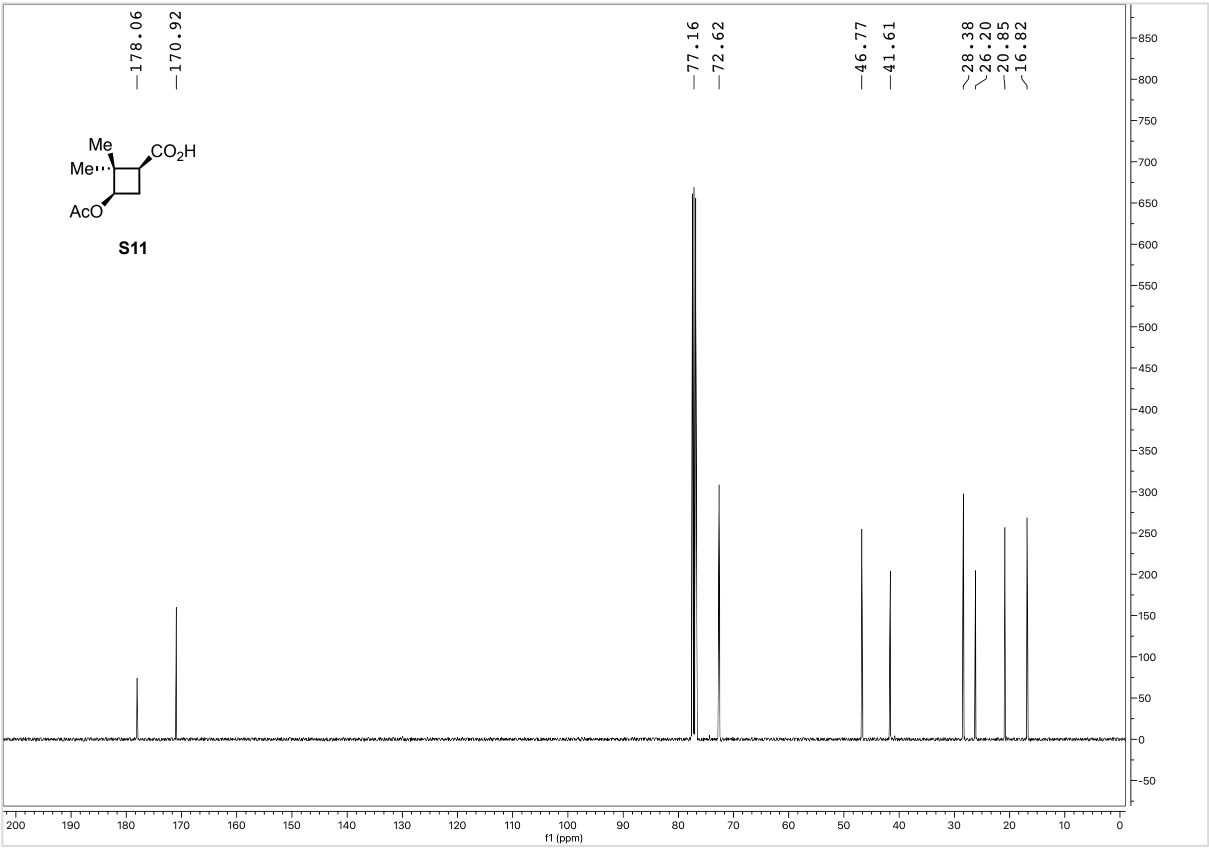

^{1}H NMR (400 MHz, $CDCl_3$) of **16**

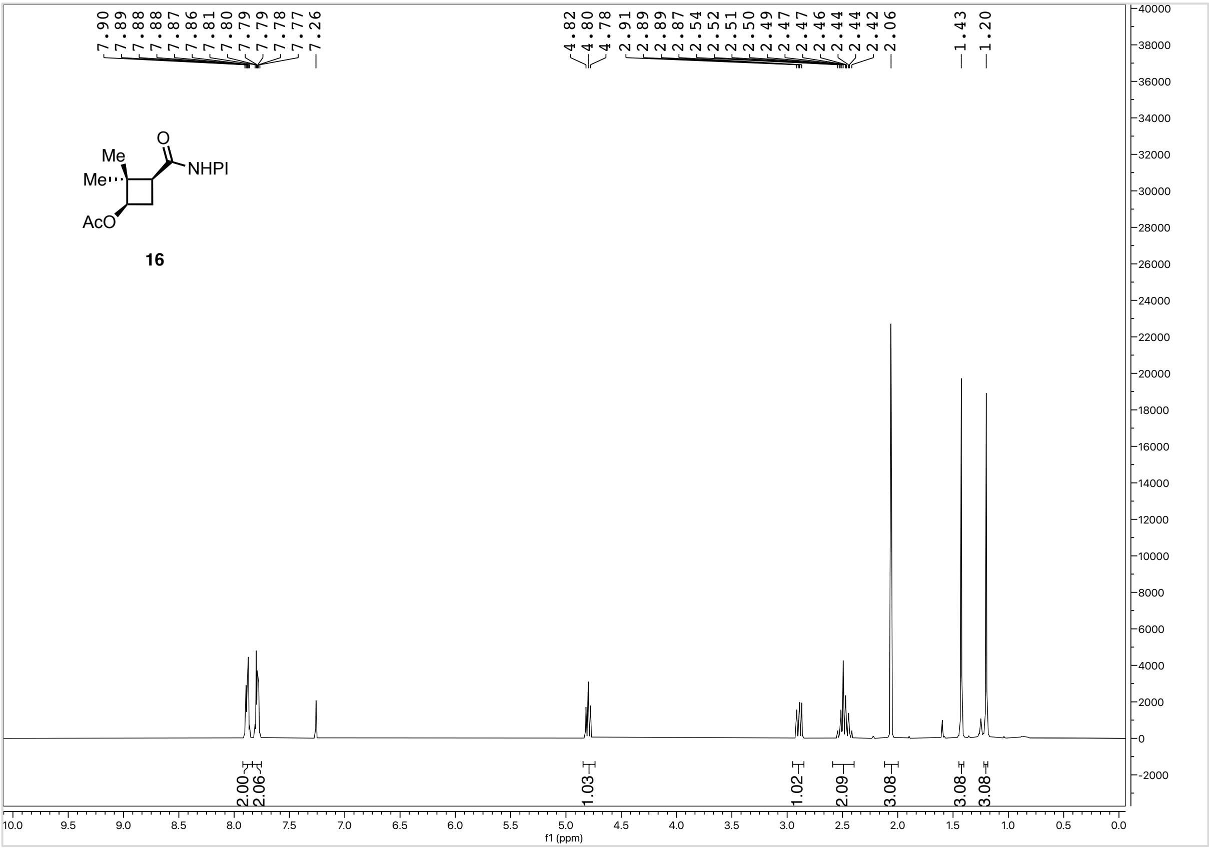

^{13}C NMR (101 MHz, $CDCl_3$) of **16**

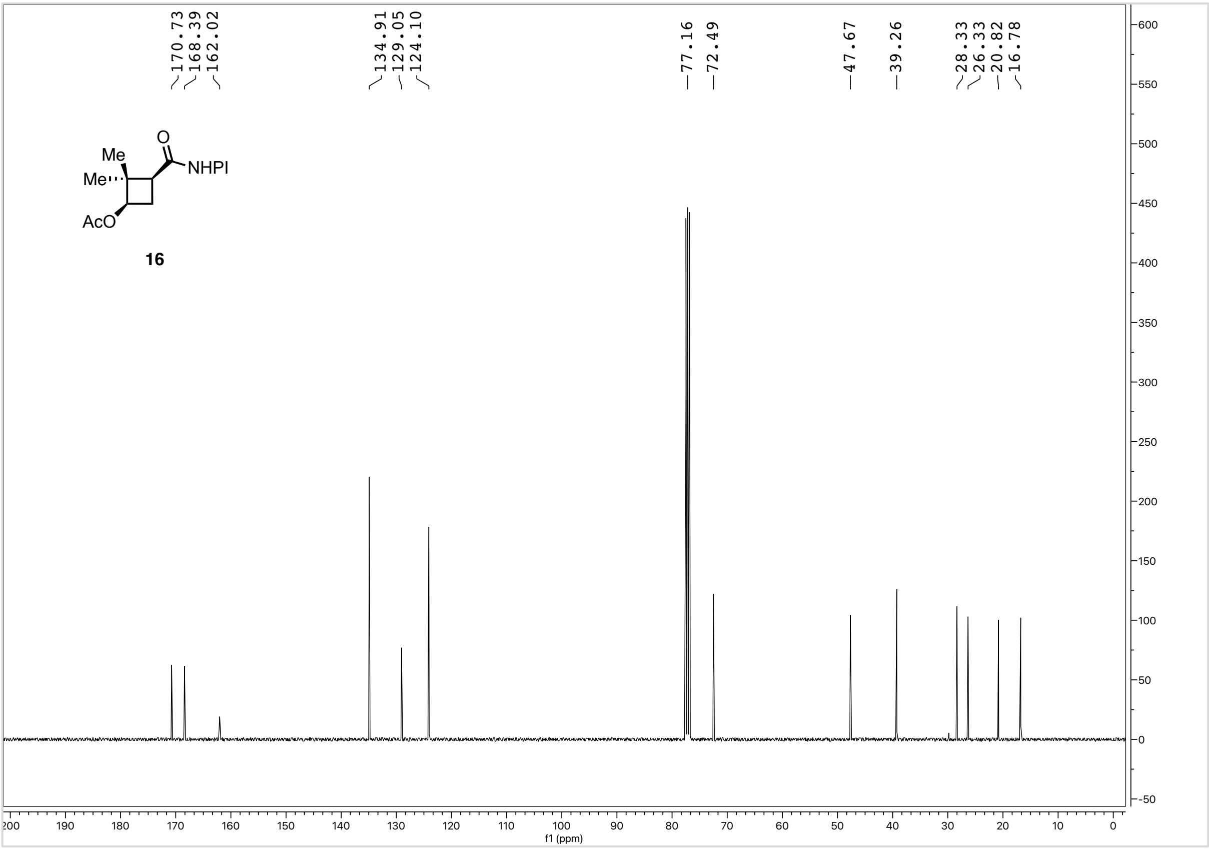

^{1}H NMR (400 MHz, $CDCl_3$) of **17**

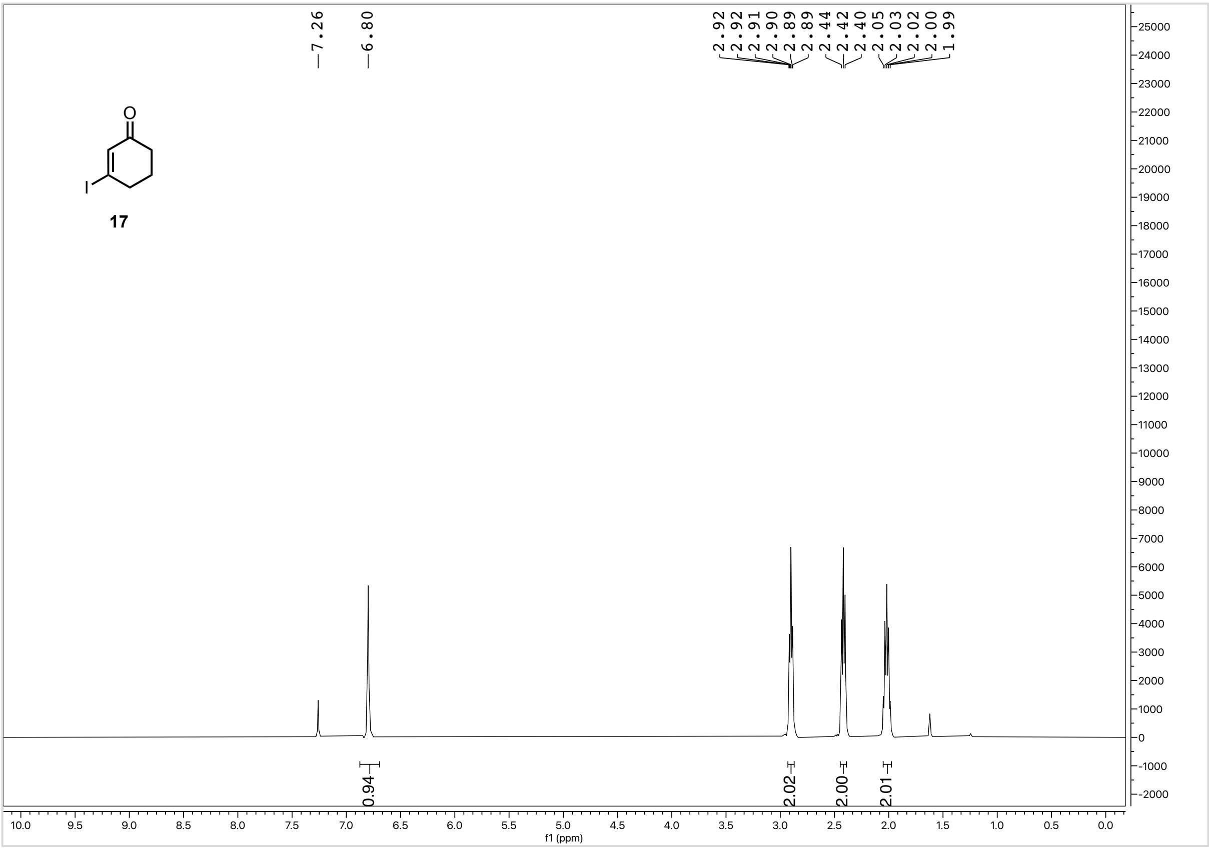

^{13}C NMR (101 MHz, $CDCl_3$) of **17**

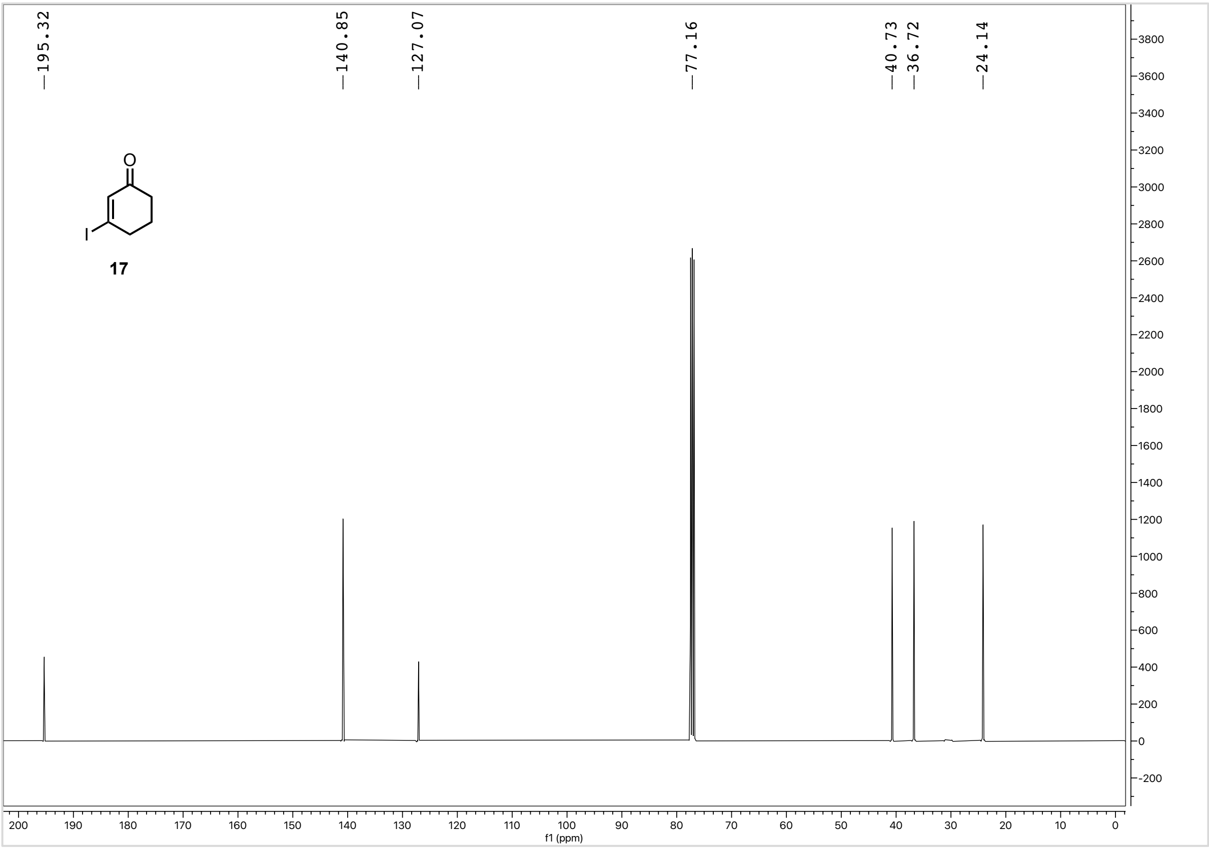

^{1}H NMR (400 MHz, $CDCl_3$) of **20** (11:1 inseparable mixture of diastereomers at C24)

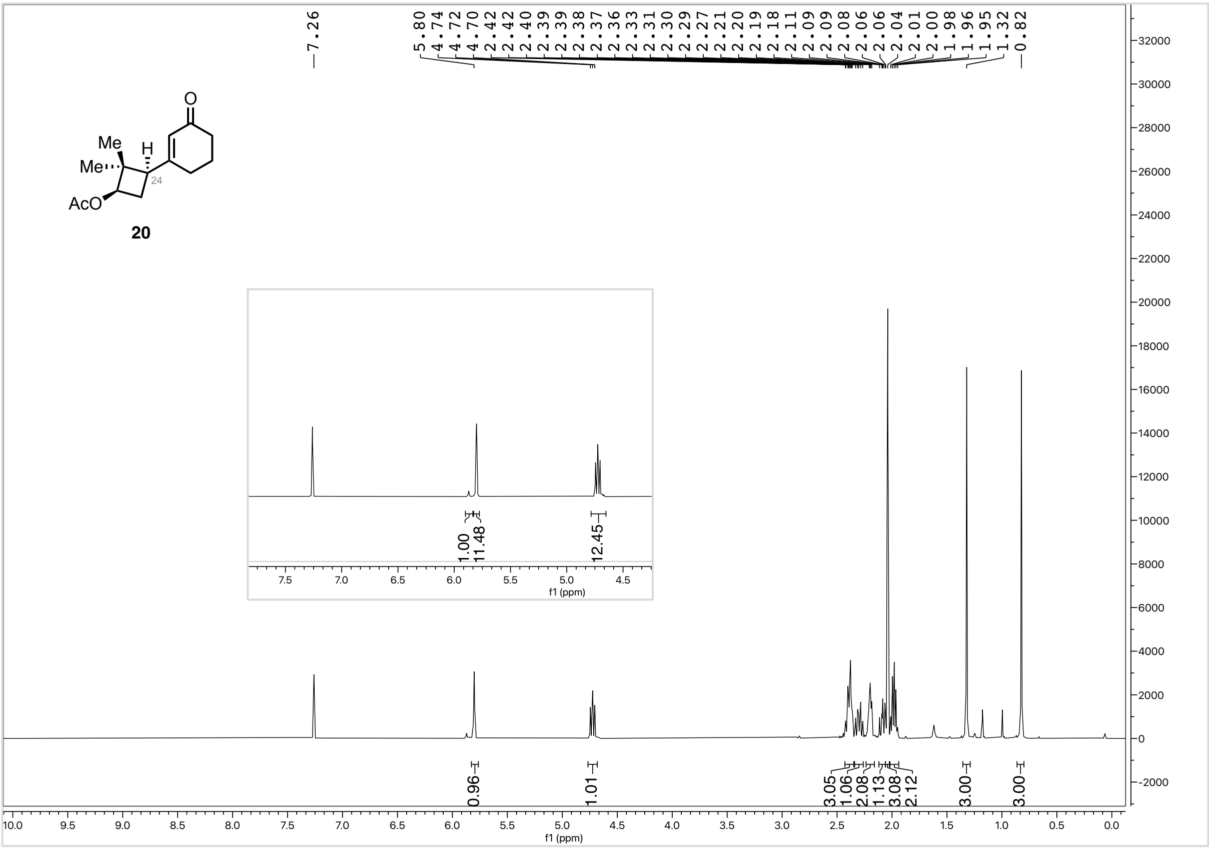

^{13}C NMR (101 MHz, $CDCl_3$) of **20** (11:1 inseparable mixture of diastereomers at C24)

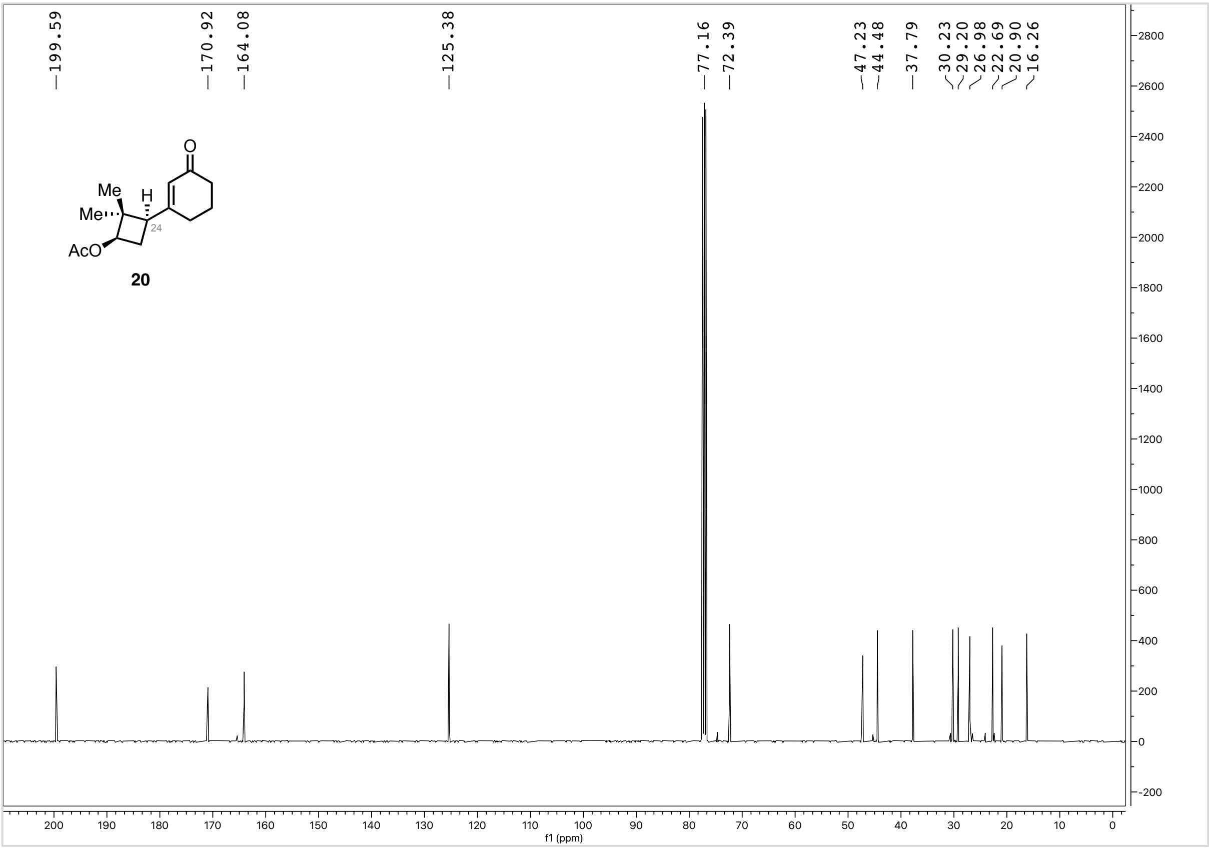

^{1}H NMR (400 MHz, $CDCl_3$) of **21** (11:1 inseparable mixture of diastereomers at C24)

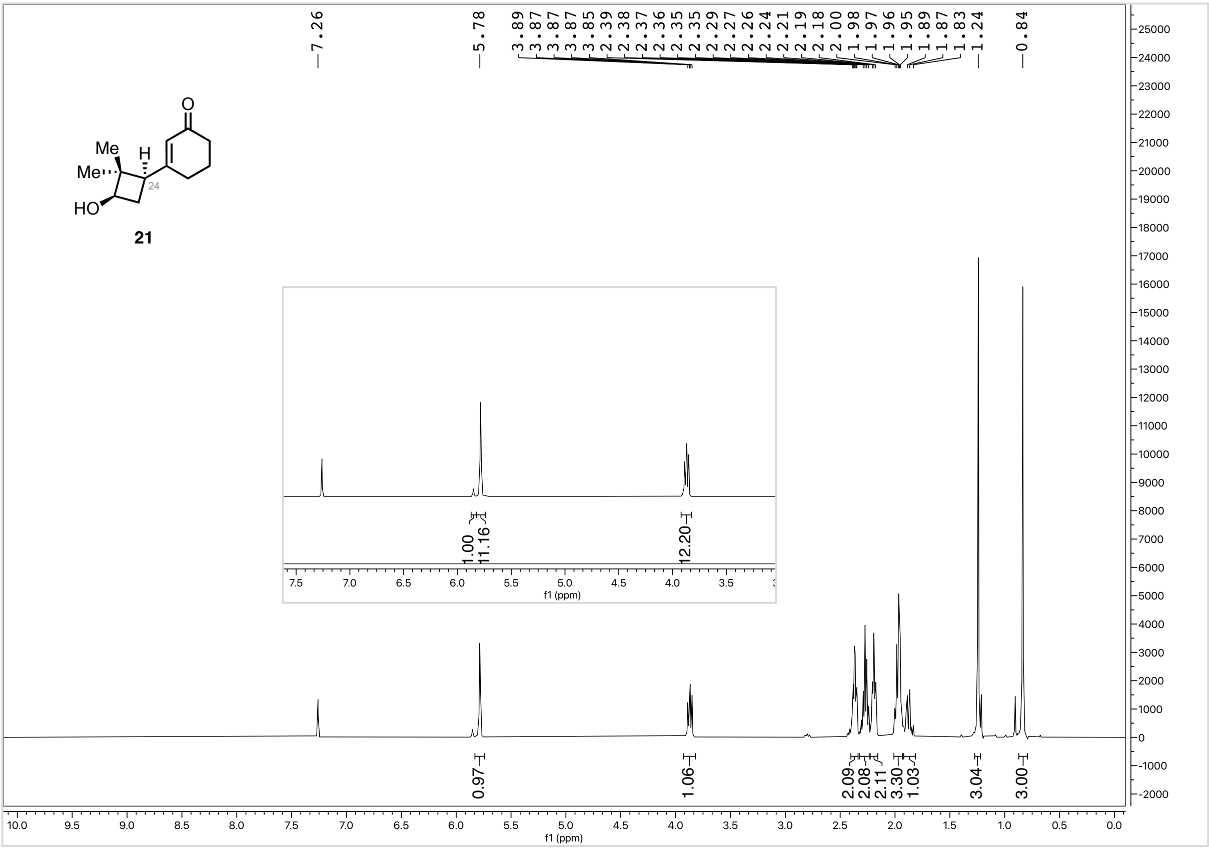

^{13}C NMR (101 MHz, $CDCl_3$) of **21** (11:1 inseparable mixture of diastereomers at C24)

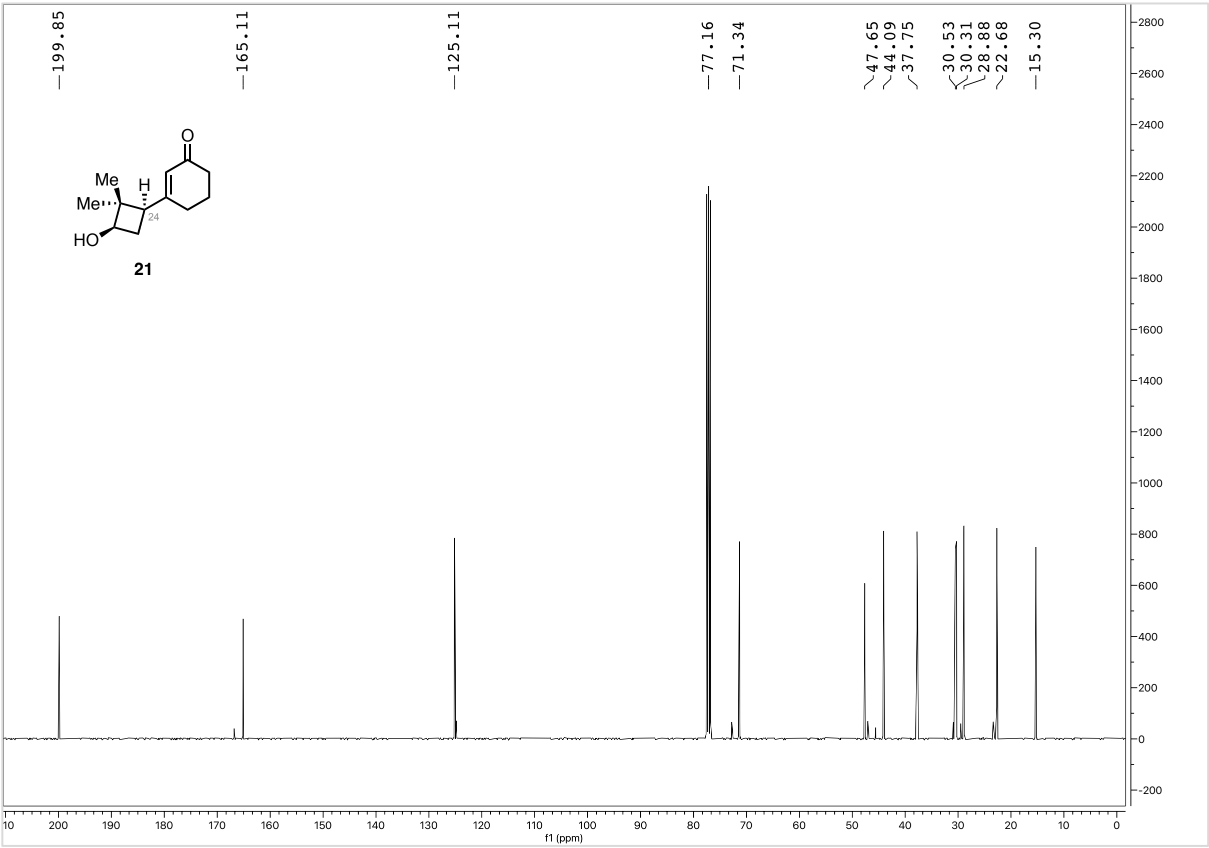

^{1}H NMR (400 MHz, $CDCl_3$) of **23**

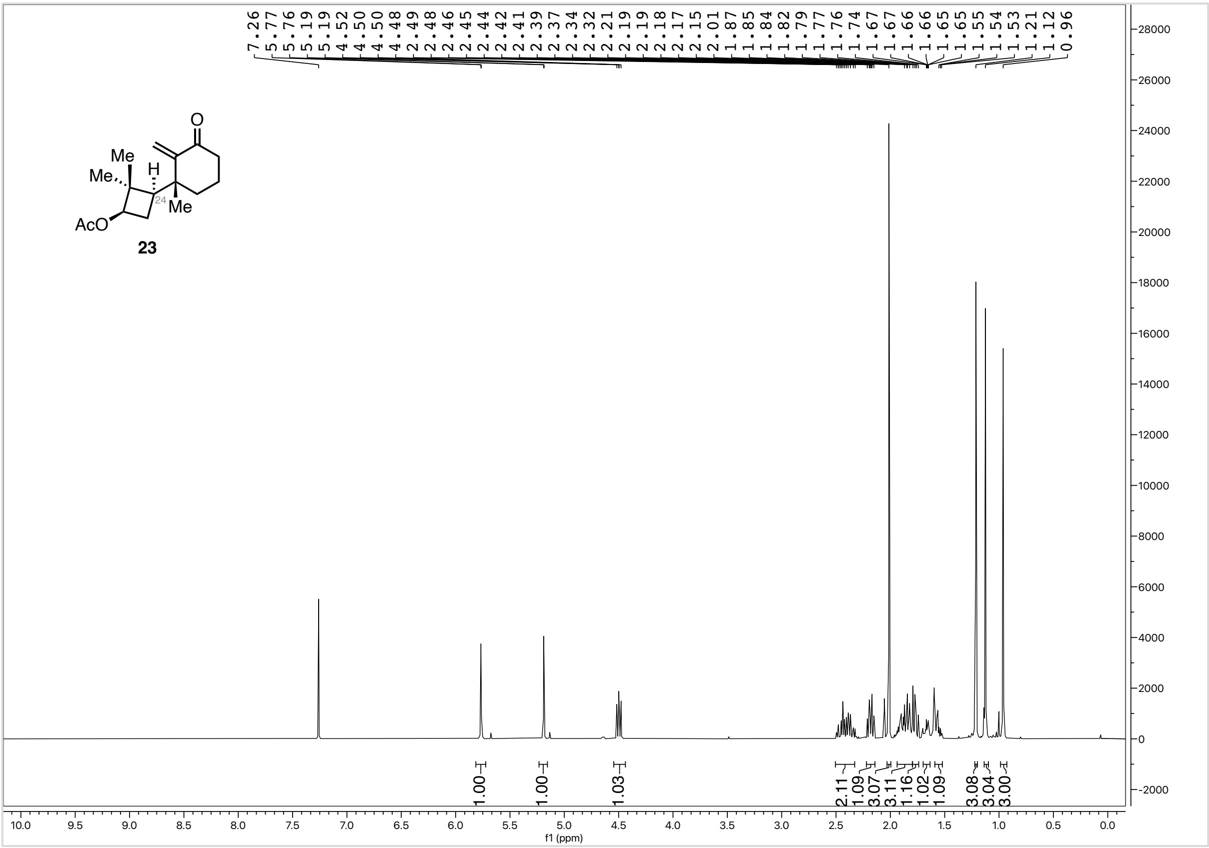

^{13}C NMR (101 MHz, $CDCl_3$) of **23** (17:1 inseparable mixture of diastereomers at C24)

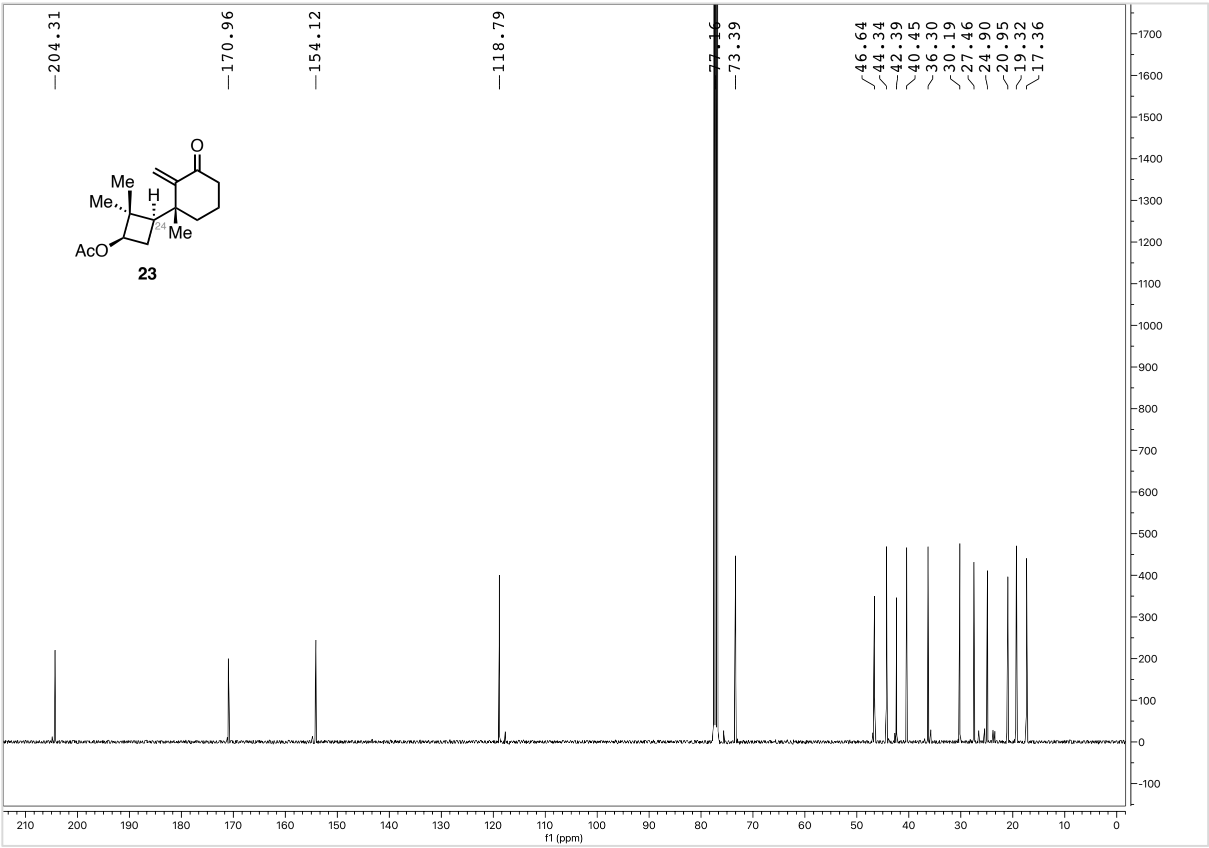

^{1}H NMR (400 MHz, $CDCl_3$) of **S12**

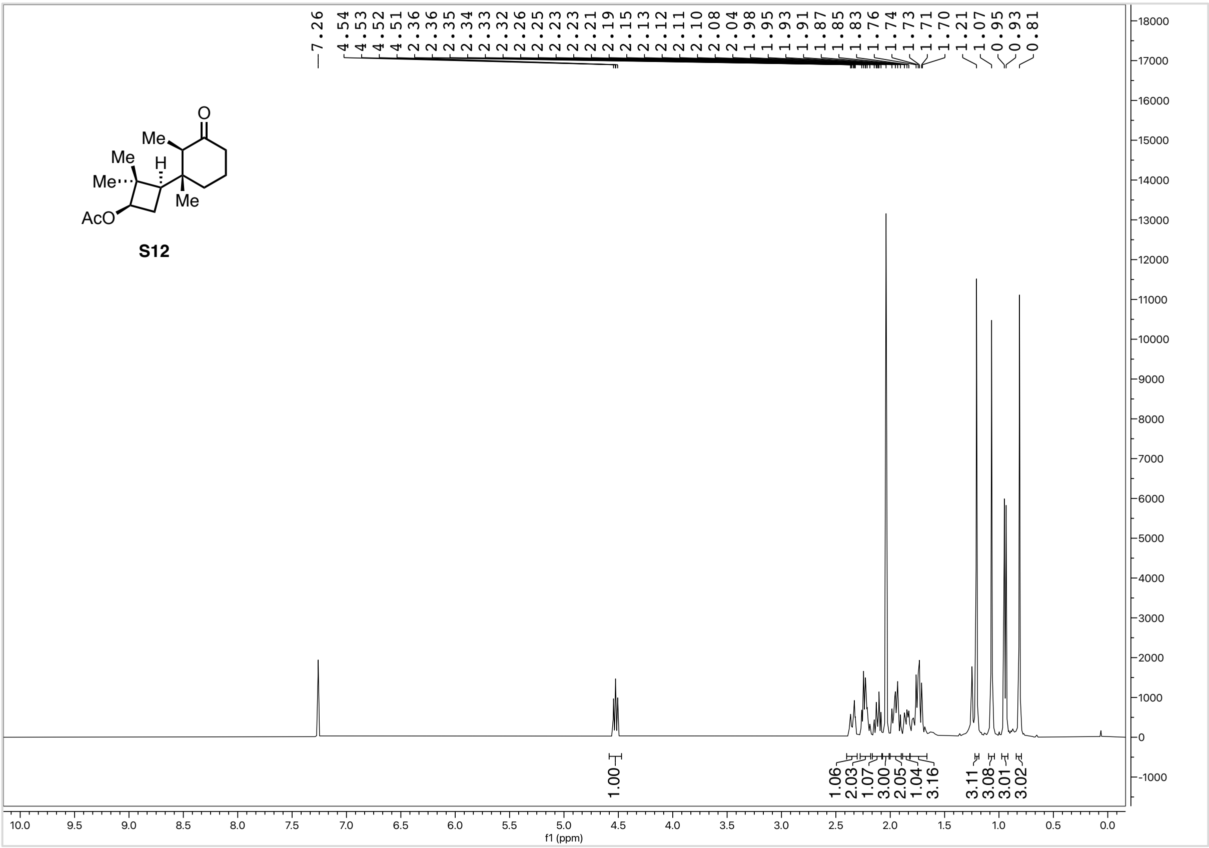

^{13}C NMR (101 MHz, $CDCl_3$) of **S12**

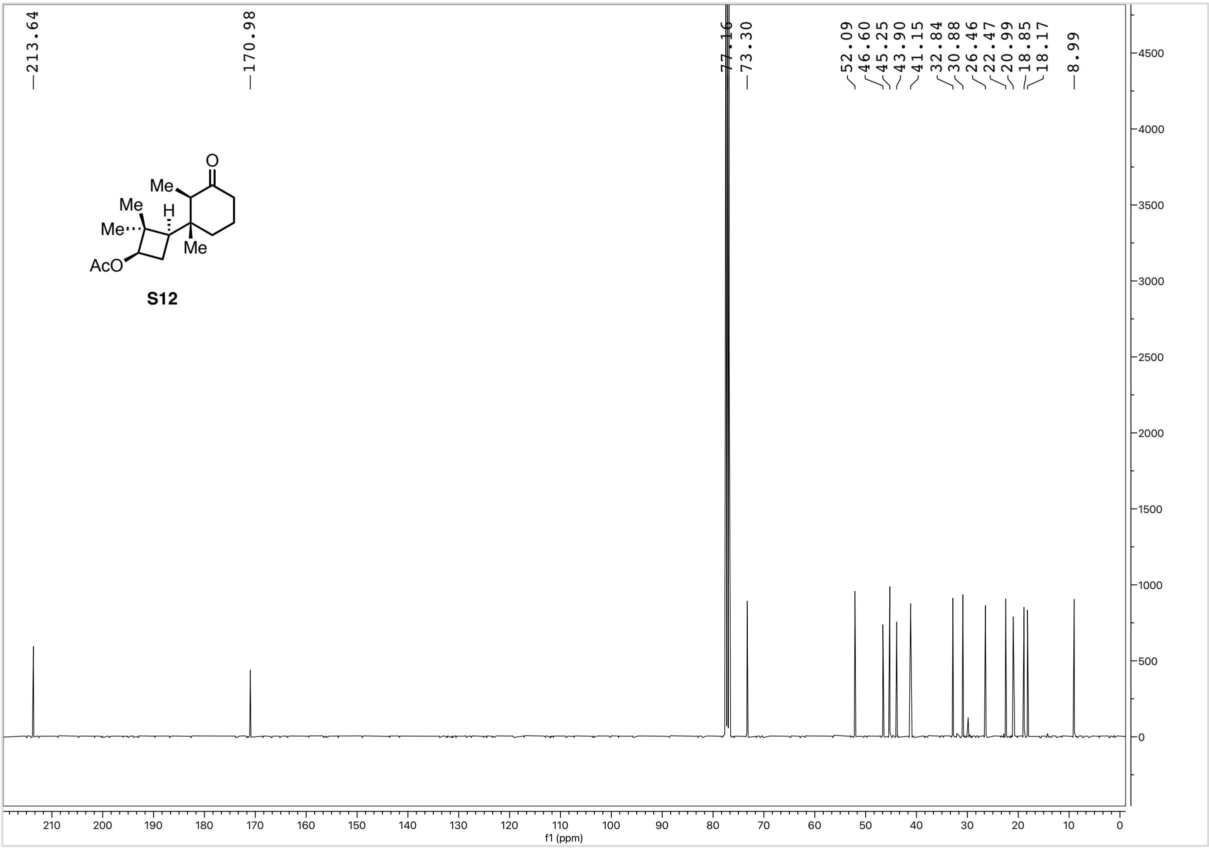

^{1}H NMR (400 MHz, $CDCl_3$) of **24**

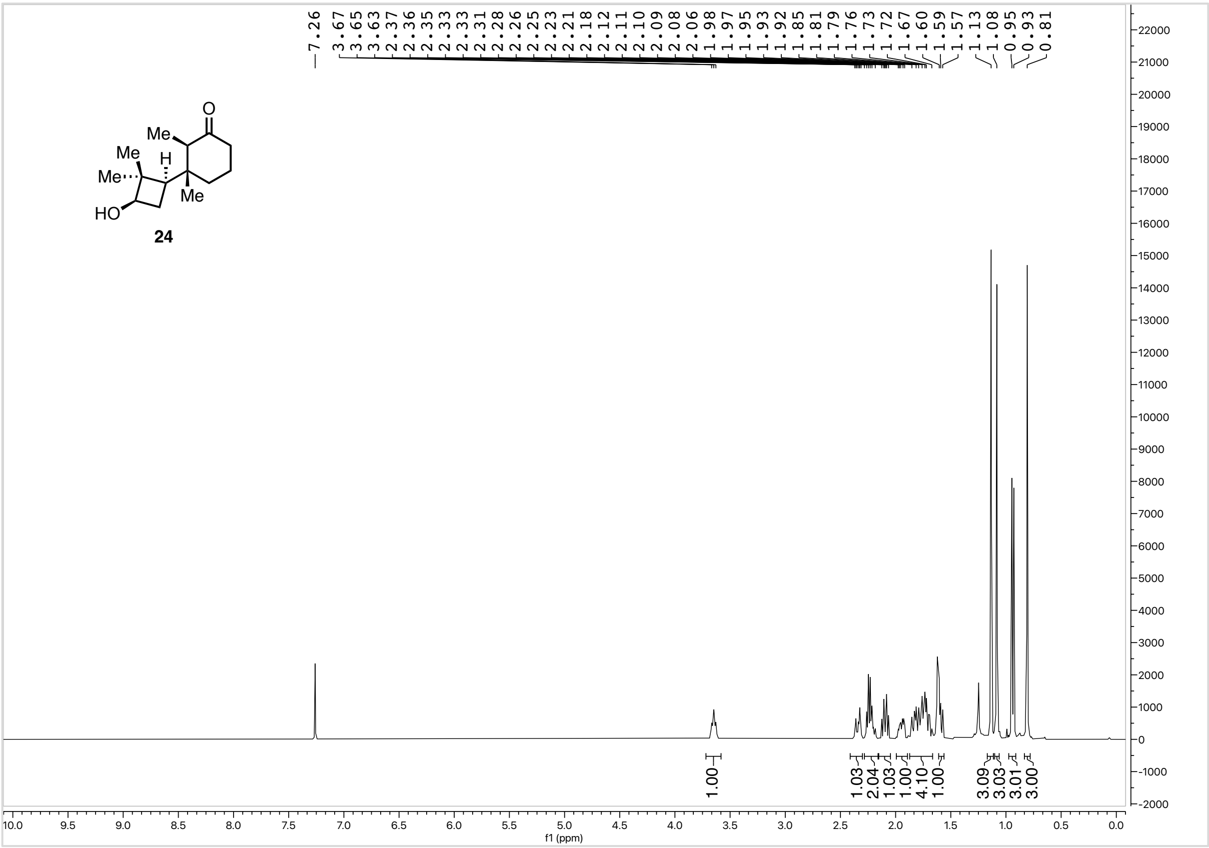

^{13}C NMR (101 MHz, $CDCl_3$) of **24**

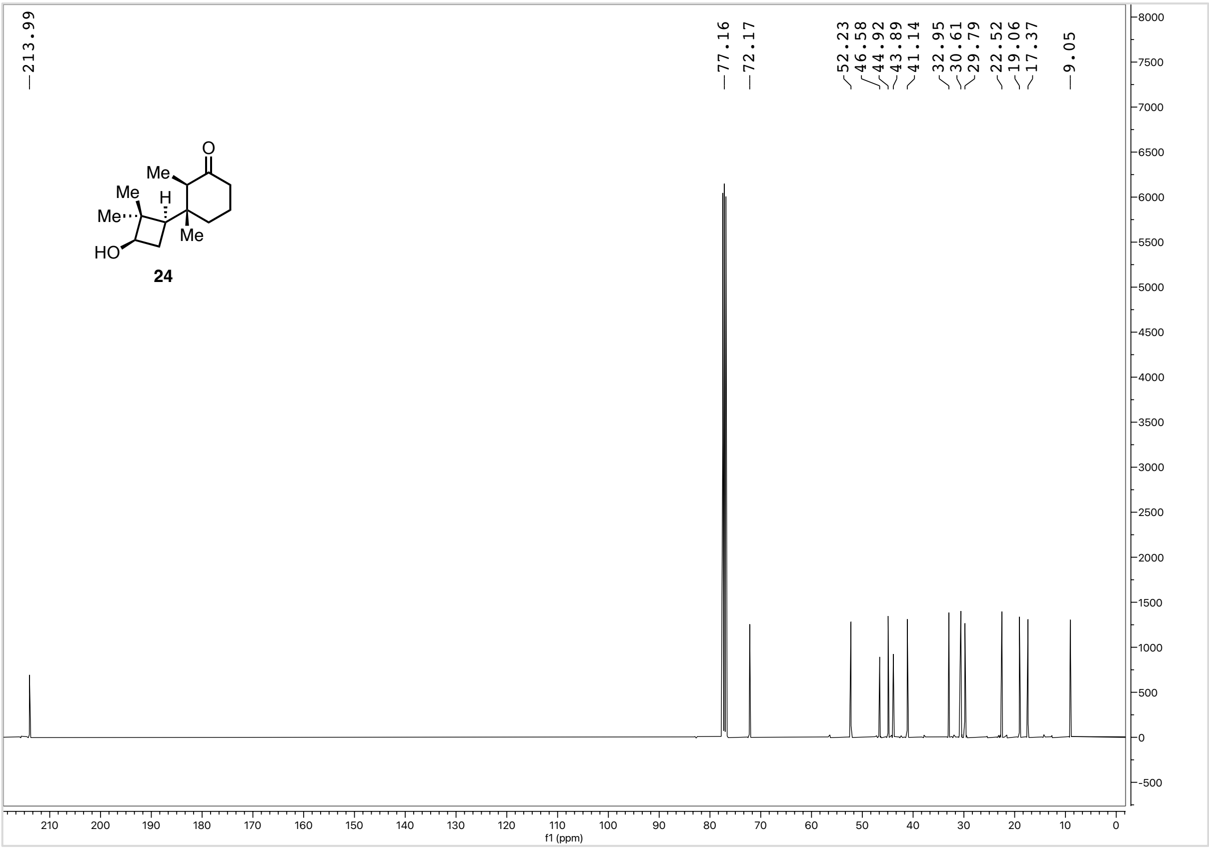

^{1}H NMR (400 MHz, $CDCl_3$) of **25**

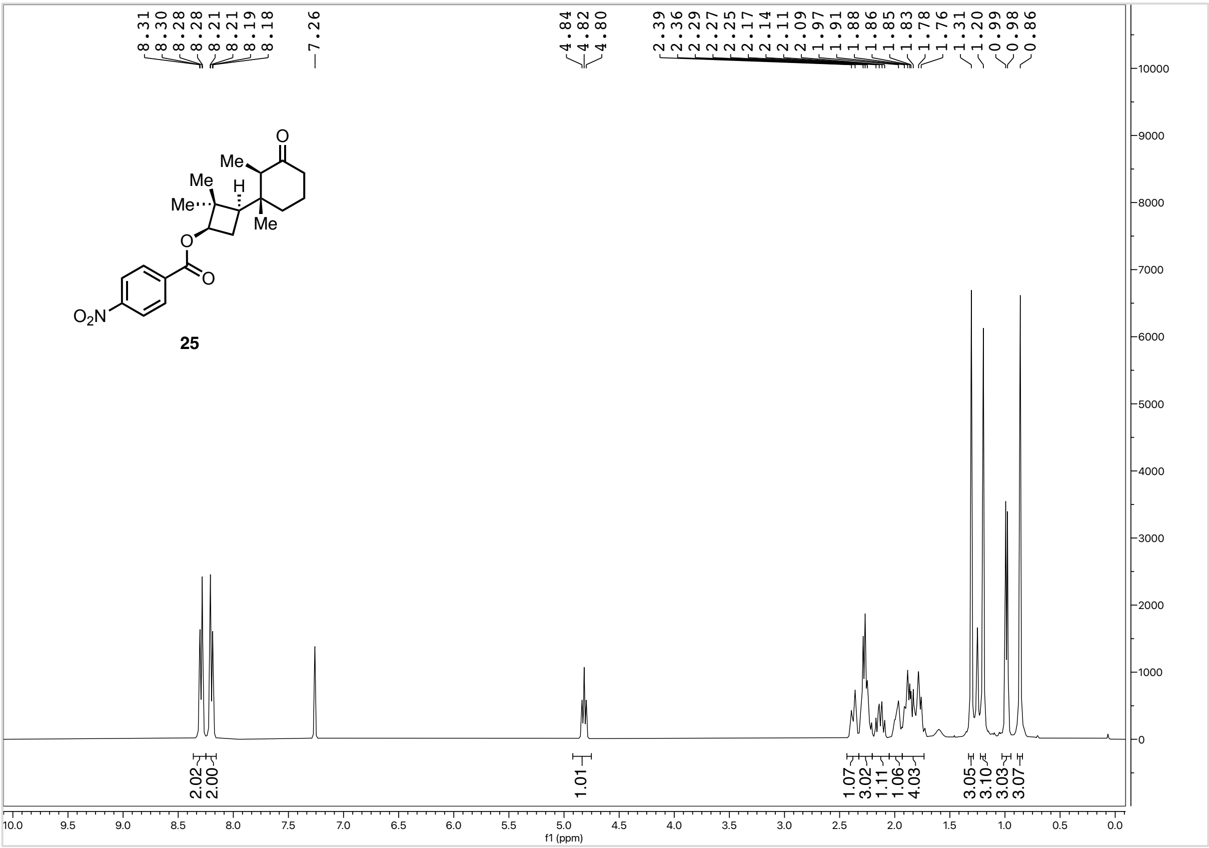

^{13}C NMR (101 MHz, $CDCl_3$) of **25**

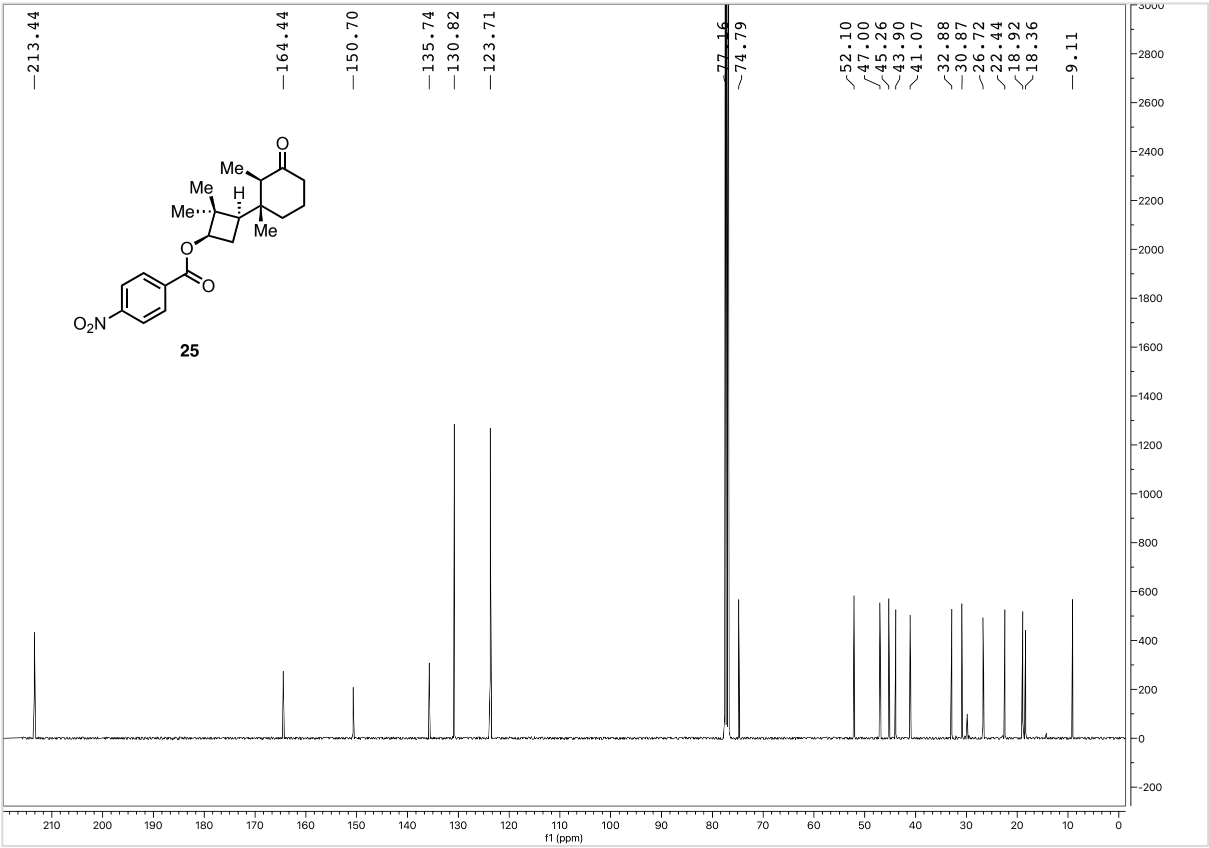

^{1}H NMR (400 MHz, $CDCl_3$) of **S13**

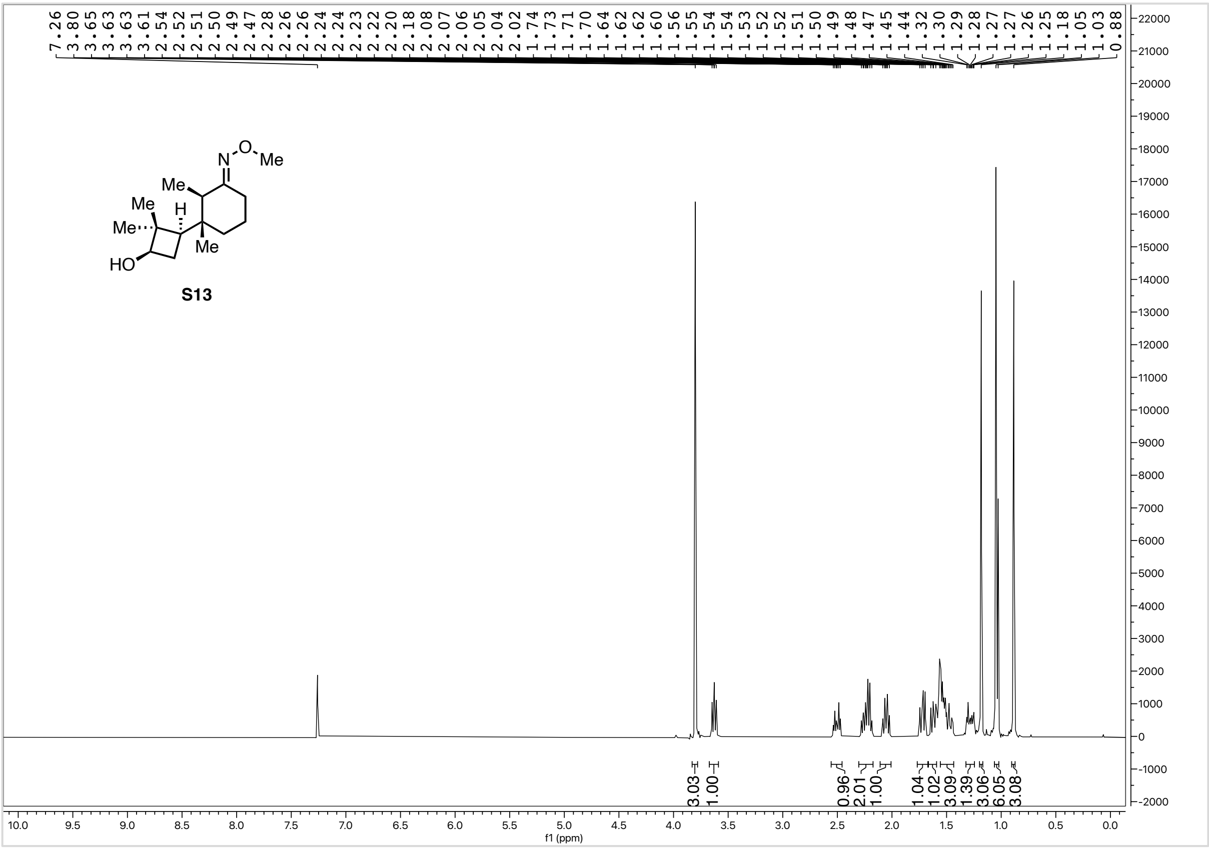

^{13}C NMR (101 MHz, $CDCl_3$) of **S13**

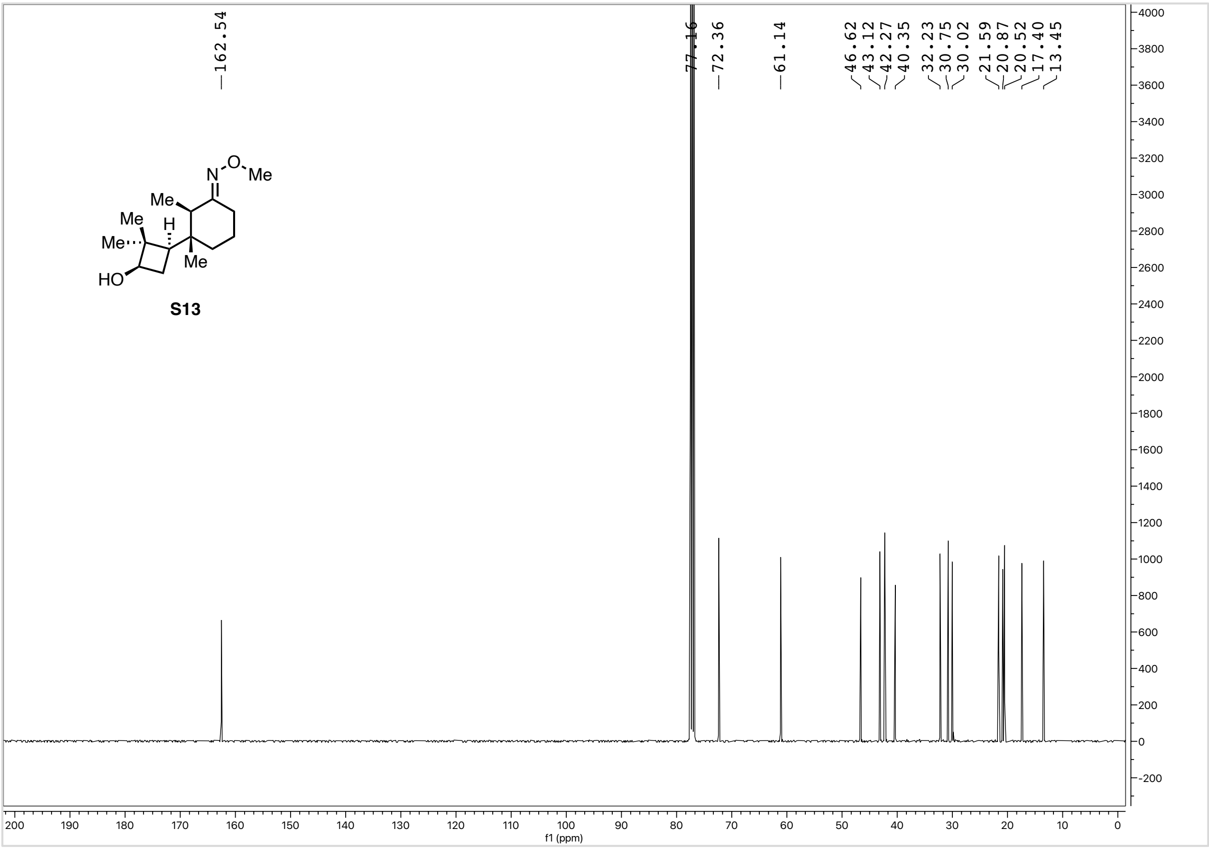

^{1}H NMR (400 MHz, $CDCl_3$) of **26**

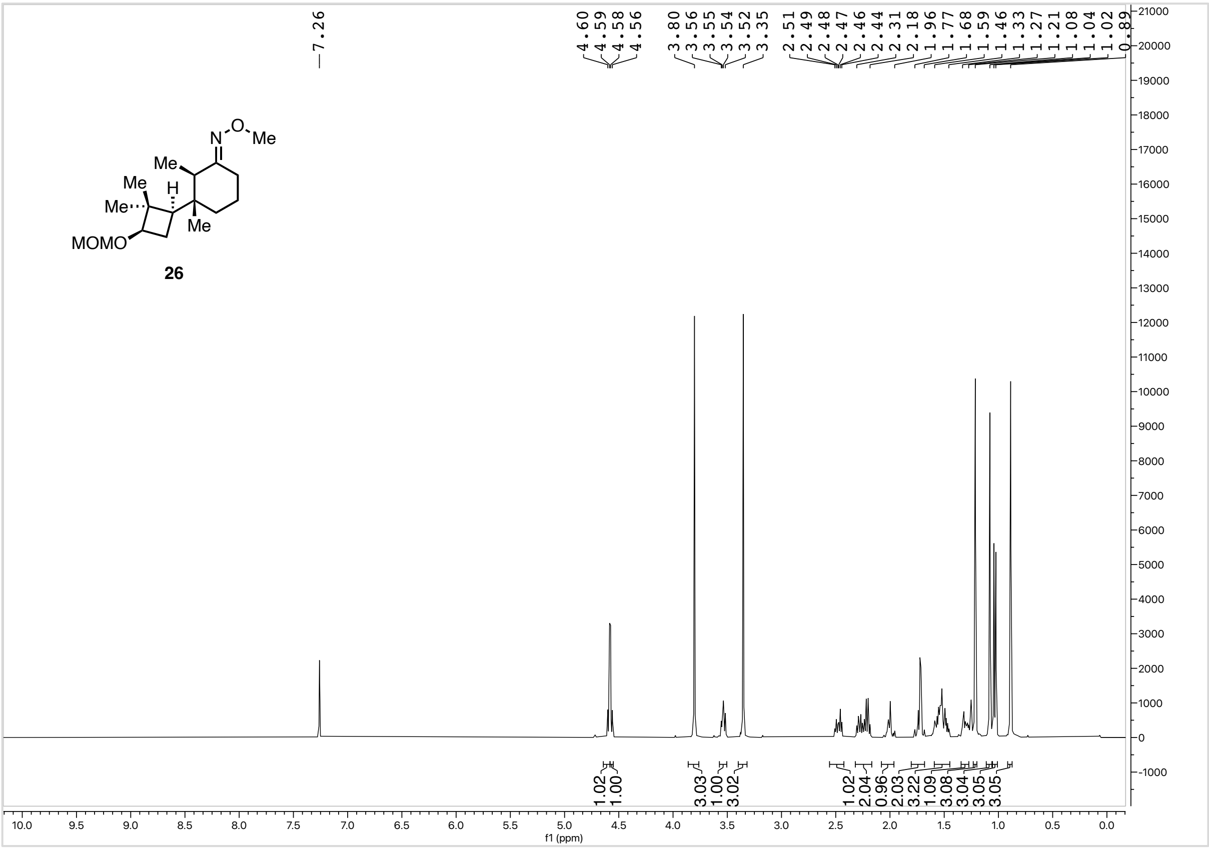

^{13}C NMR (101 MHz, $CDCl_3$) of **26**

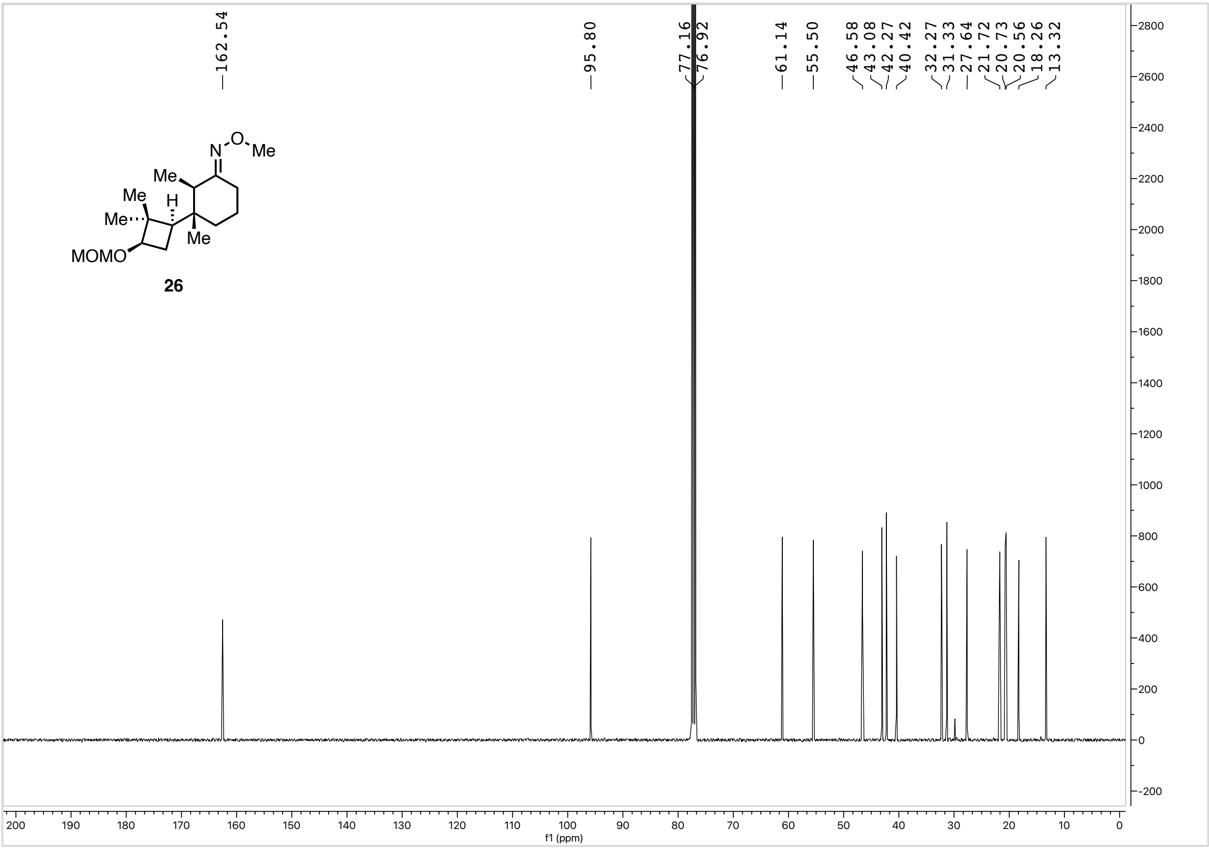

^{1}H NMR (400 MHz, $CDCl_3$) of **S14**

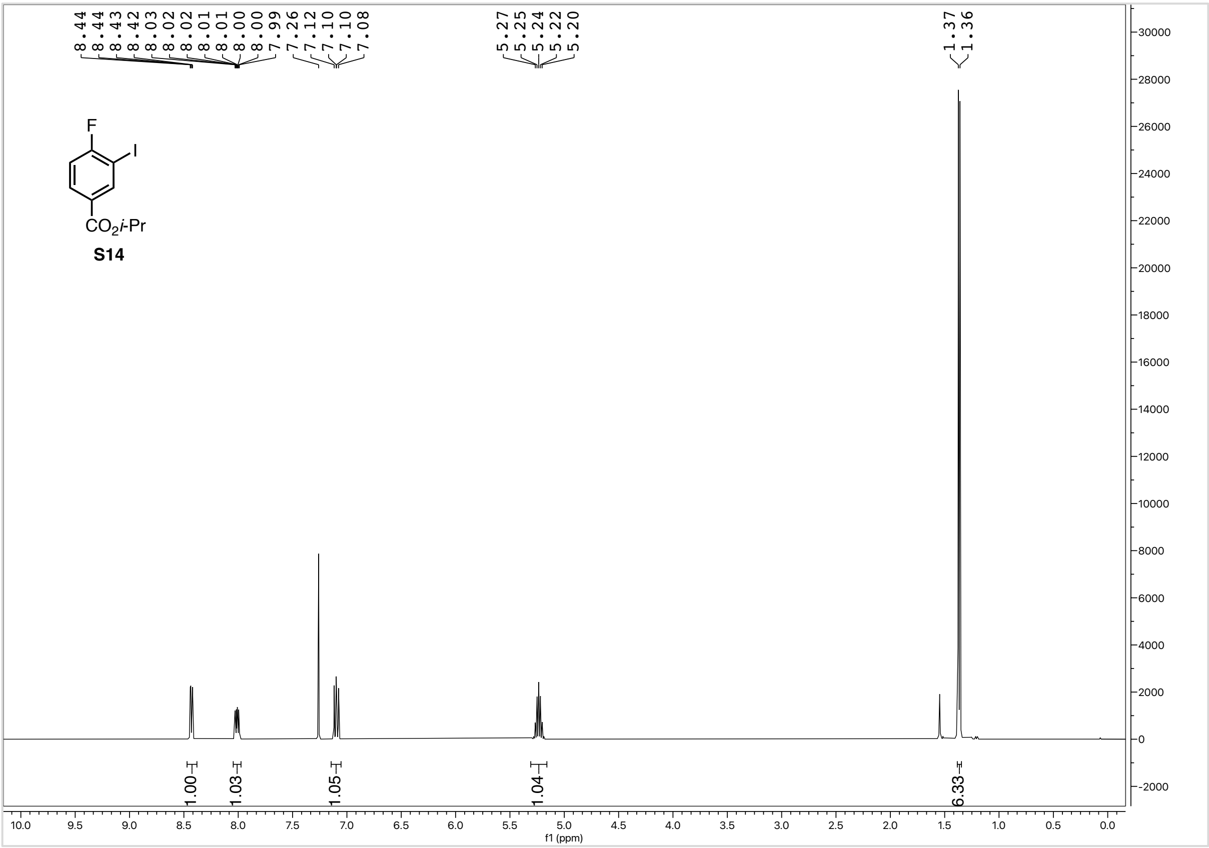

^{13}C NMR (101 MHz, $CDCl_3$) of **S14**

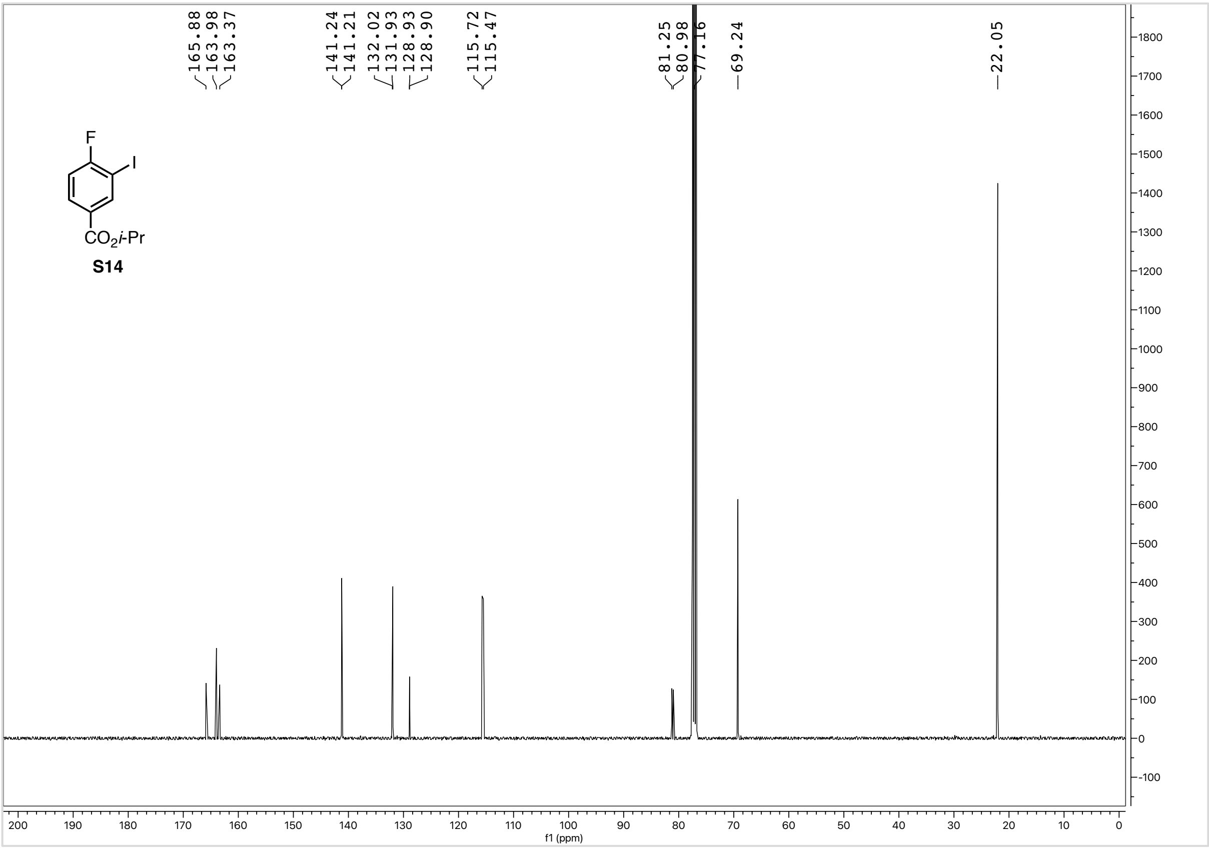

^{1}H NMR (400 MHz, DMSO-d6) of **S15**

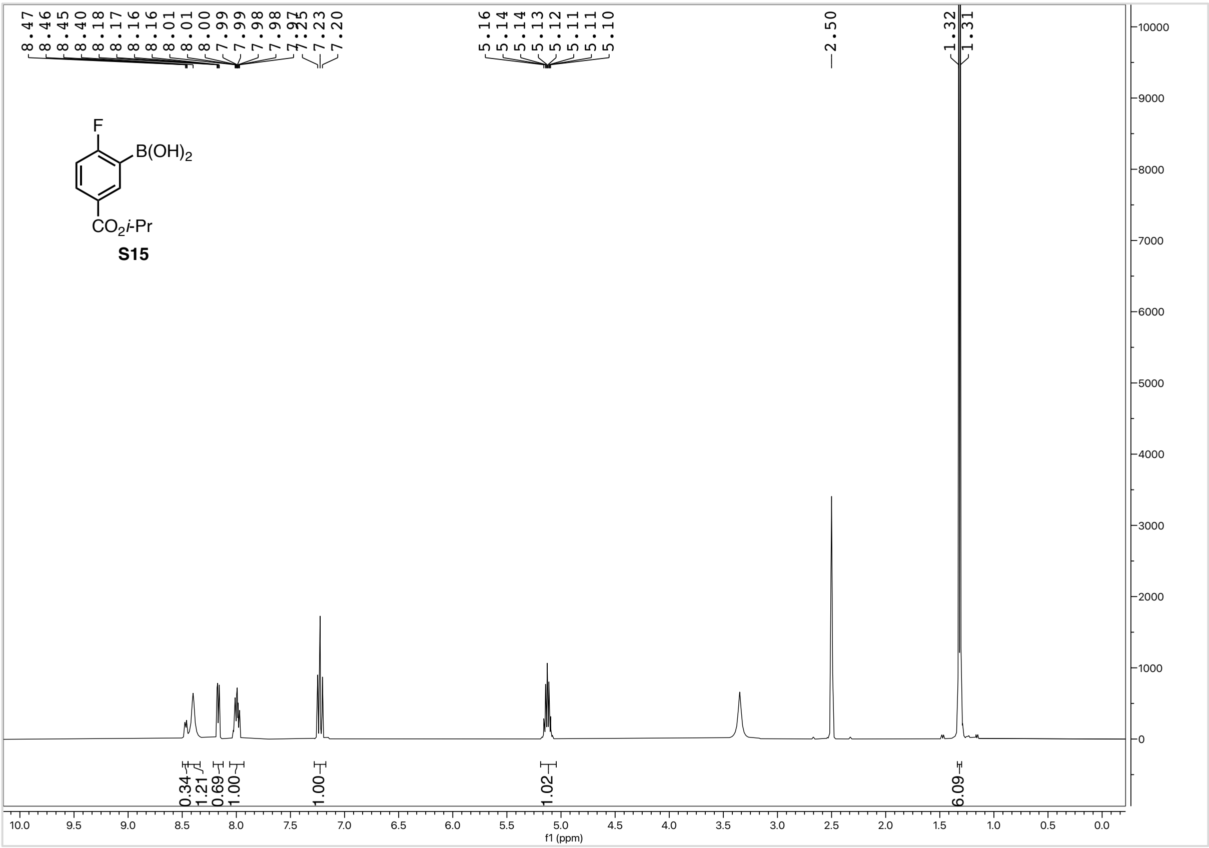

^{13}C NMR (101 MHz, DMSO-d6) of **S15**

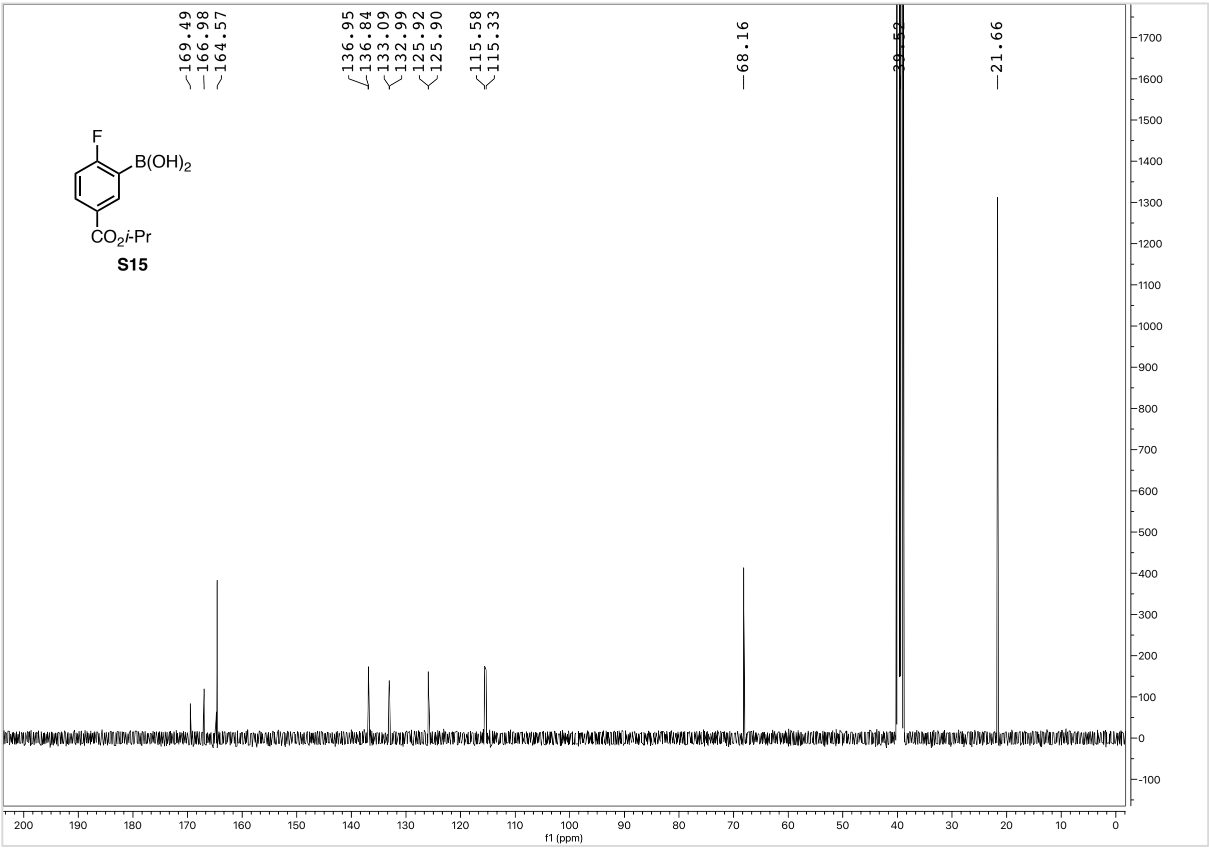

^{1}H NMR (400 MHz, DMSO-d6) of **28**

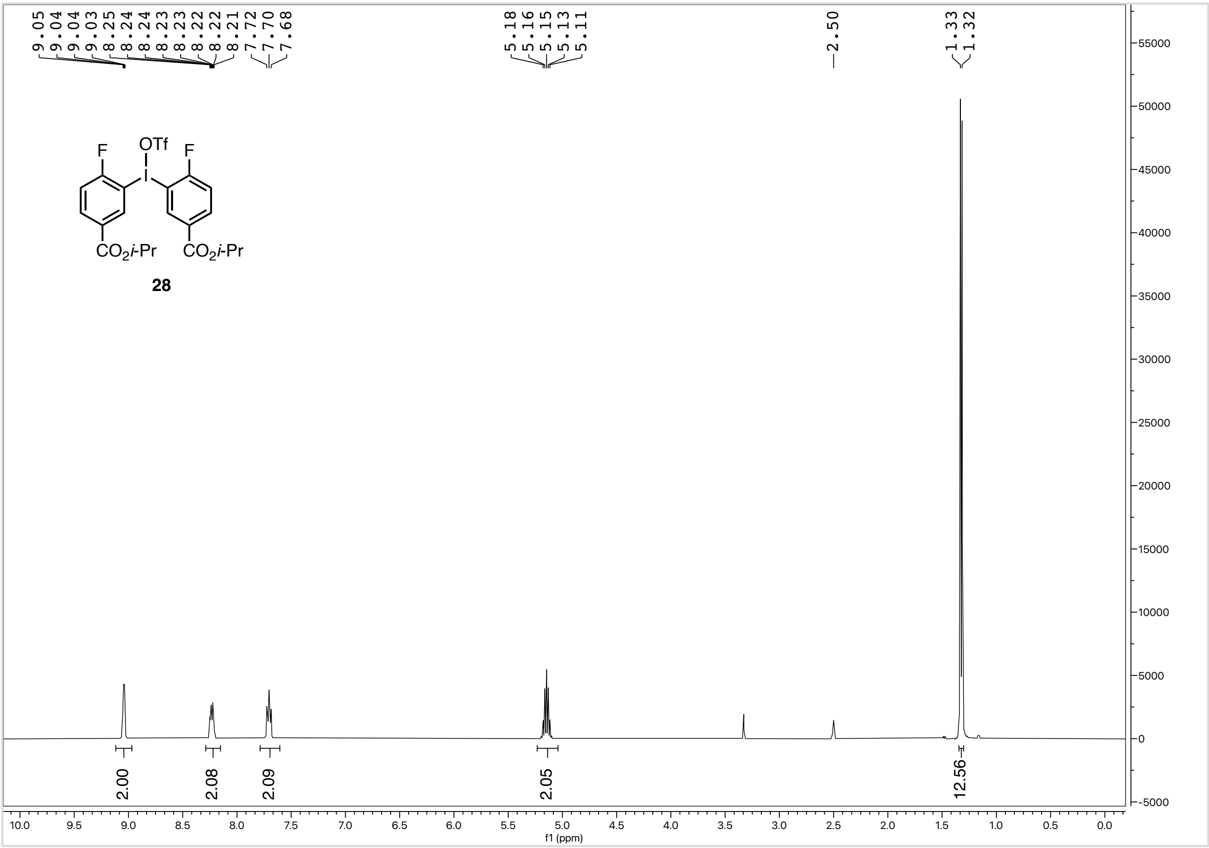

^{13}C NMR (101 MHz, DMSO-d6) of **28**

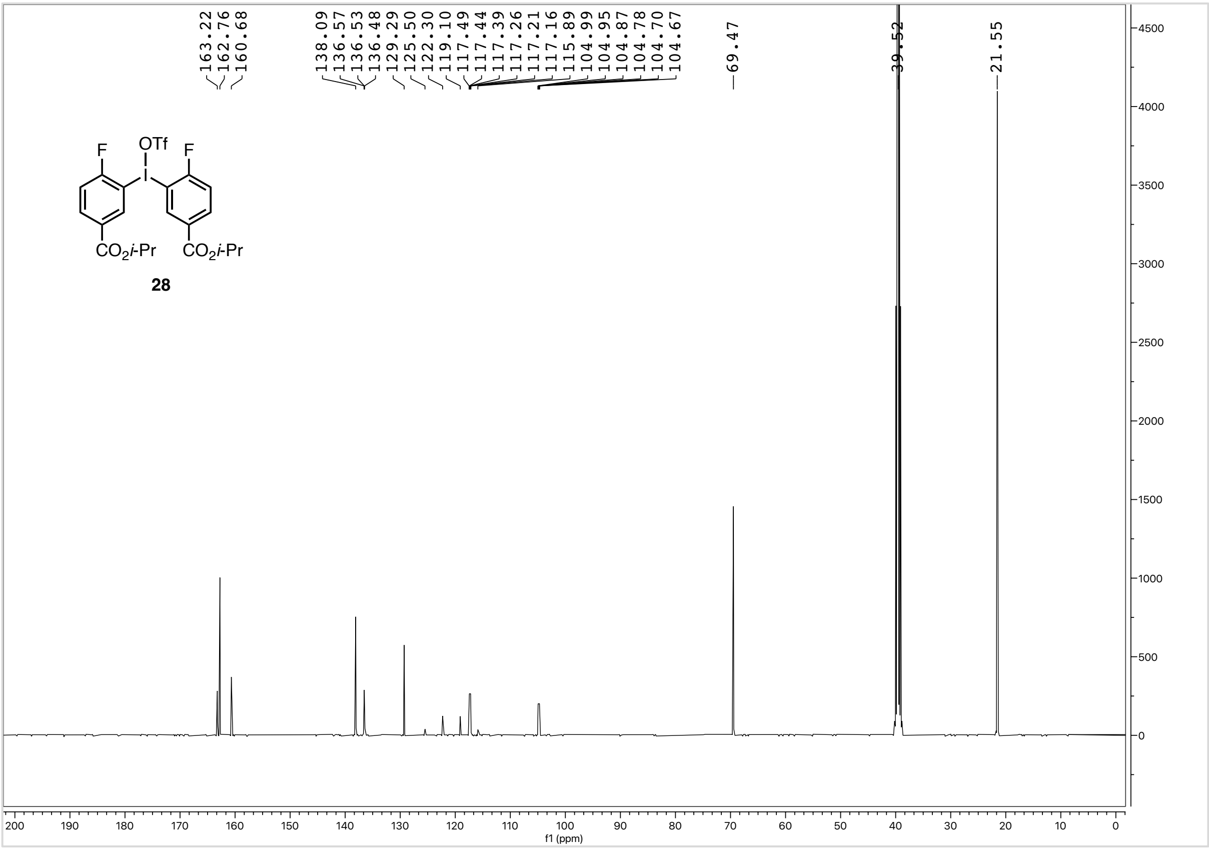

^{1}H NMR (400 MHz, $CDCl_3$) of **27**

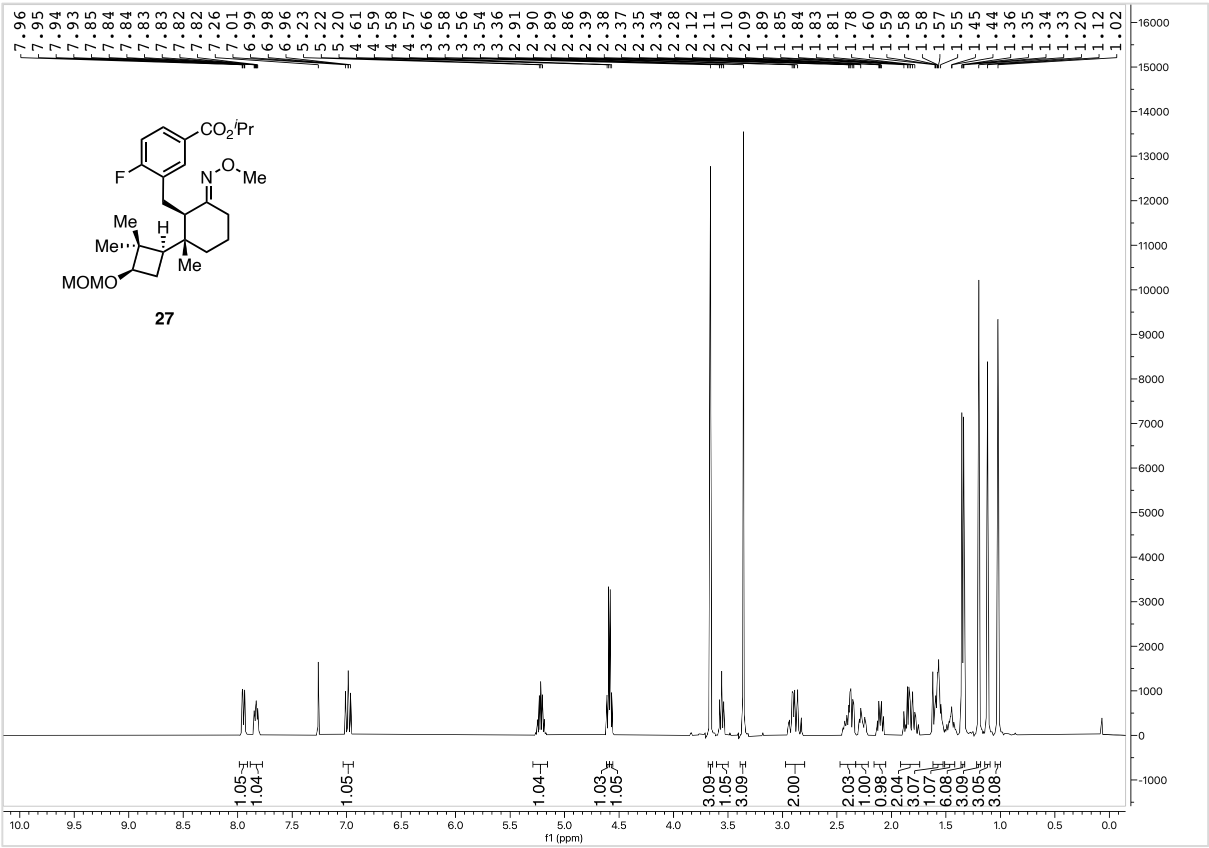

^{13}C NMR (101 MHz, $CDCl_3$) of **27**

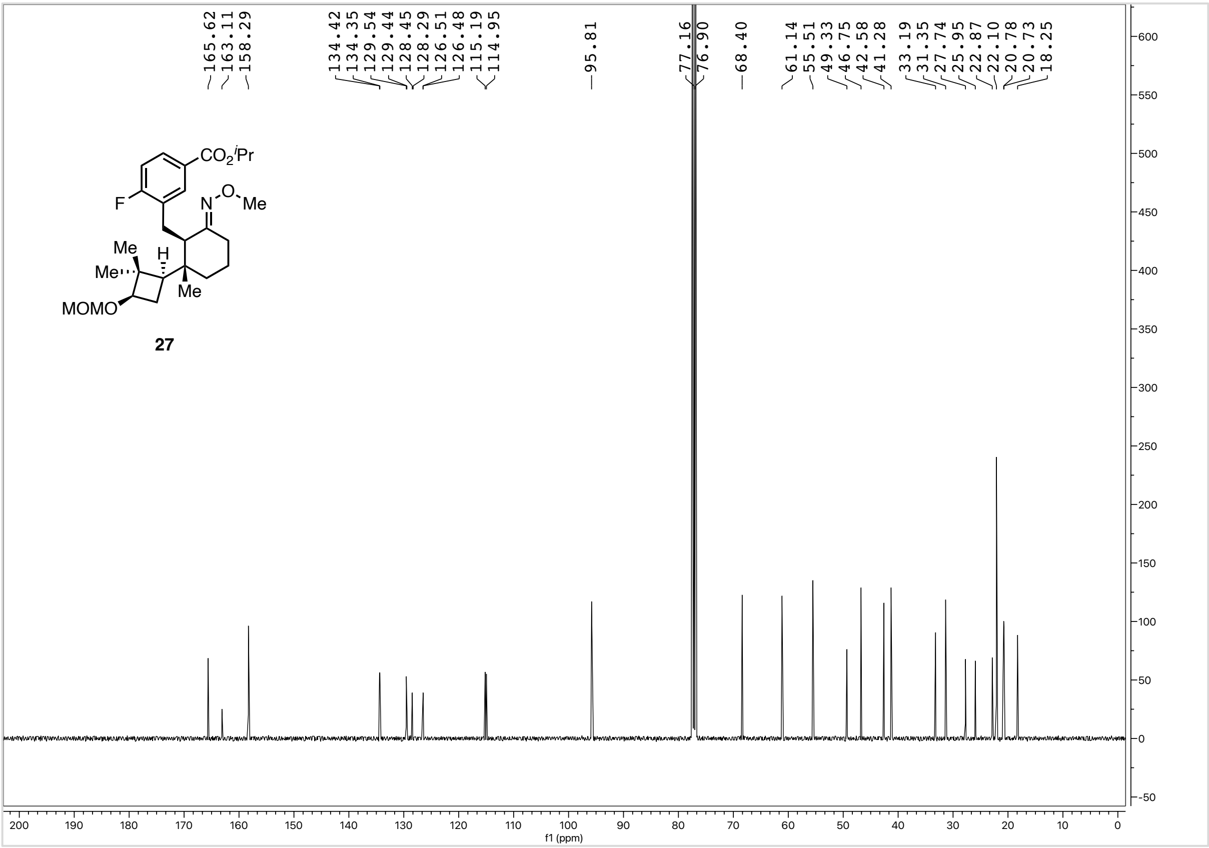

^{1}H NMR (400 MHz, $CDCl_3$) of **S16**

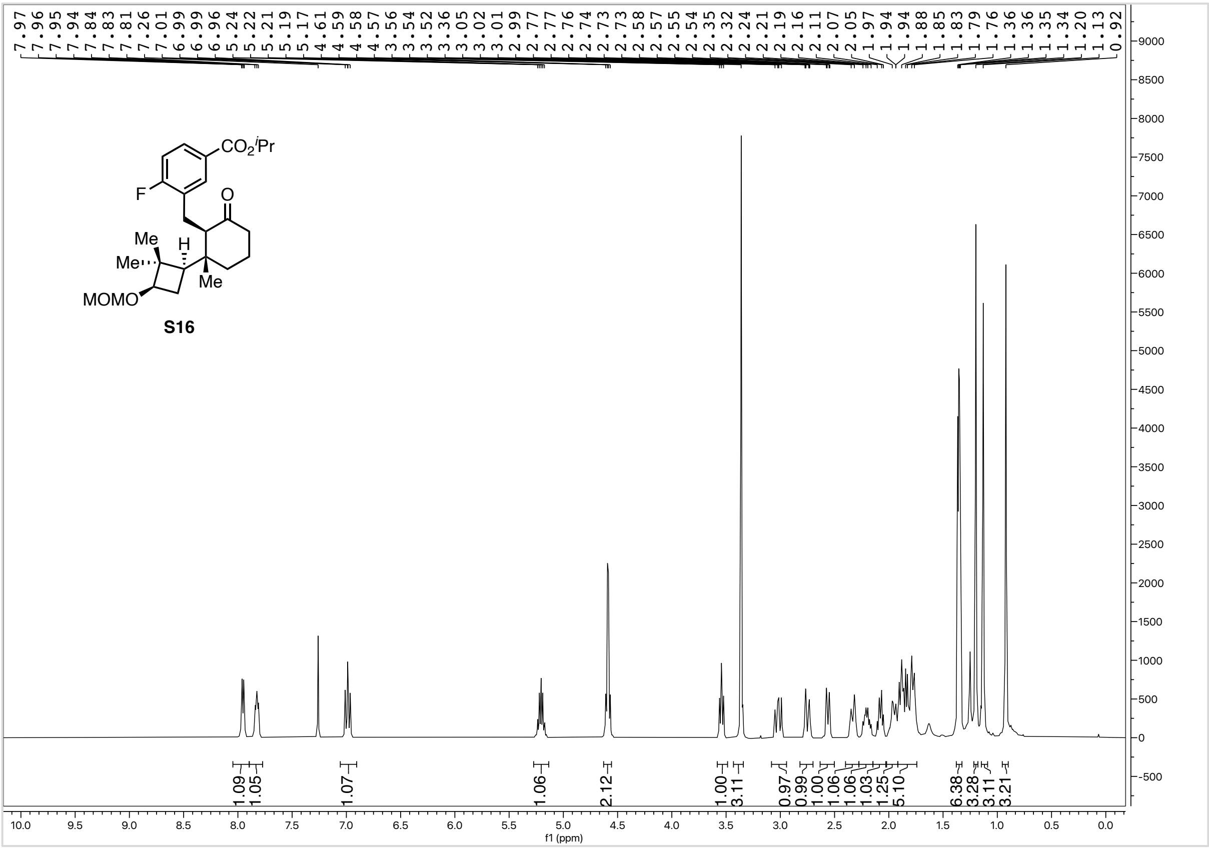

^{13}C NMR (101 MHz, $CDCl_3$) of **S16**

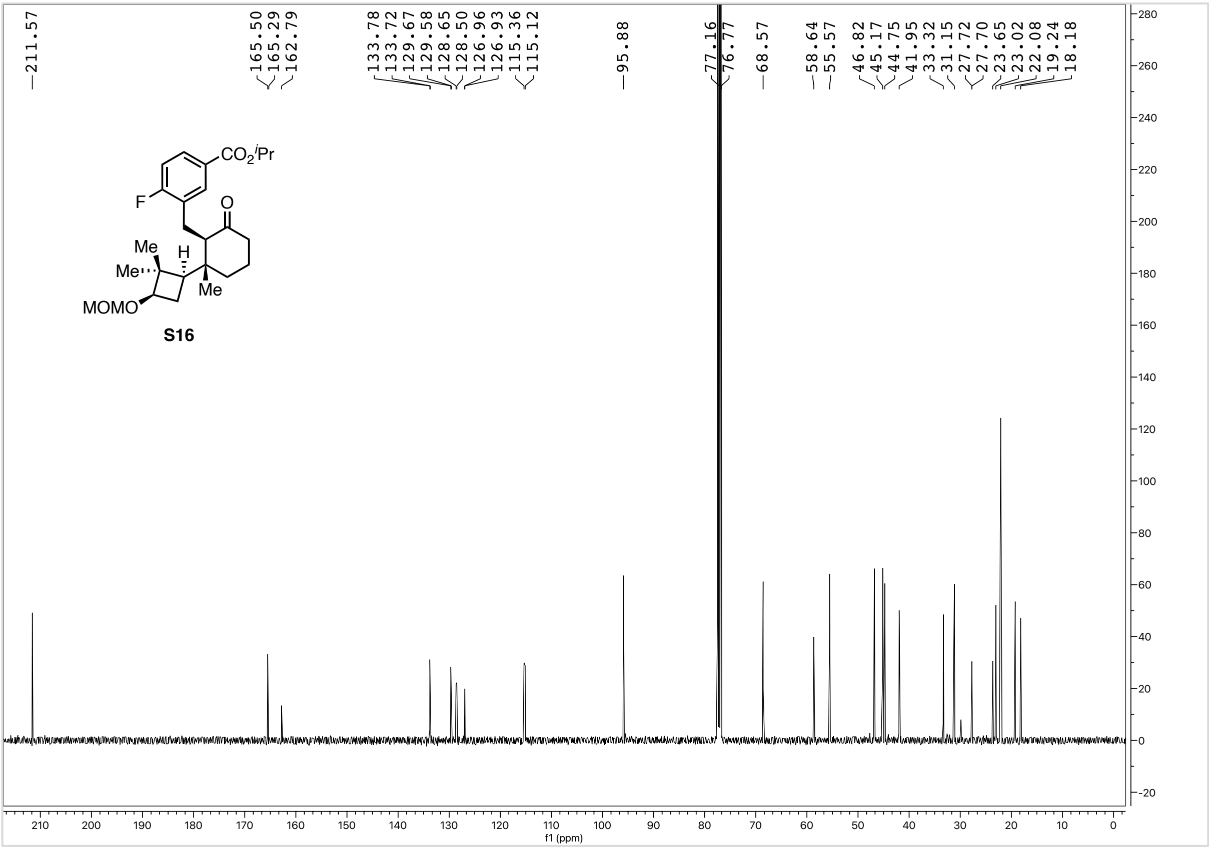

^{1}H NMR (400 MHz, $CDCl_3$) of **29**

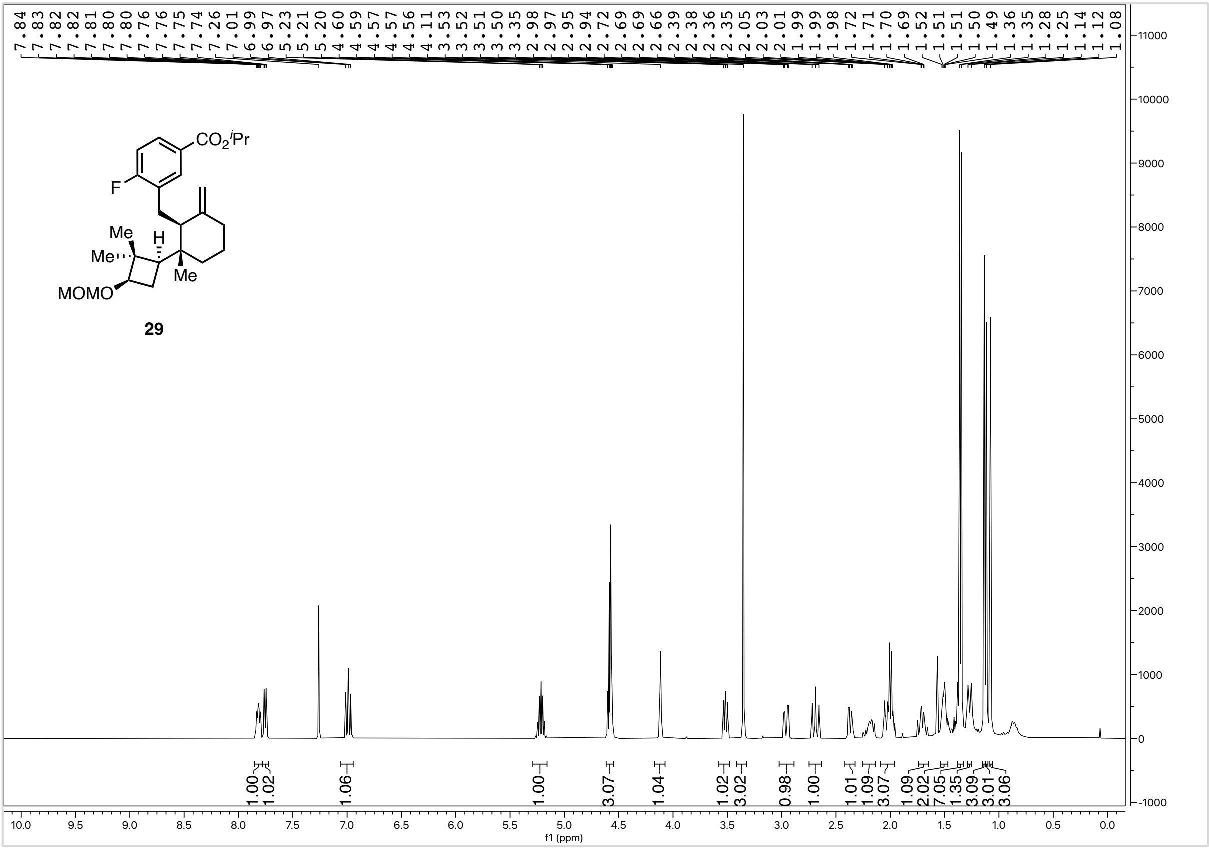

^{13}C NMR (101 MHz, $CDCl_3$) of **29**

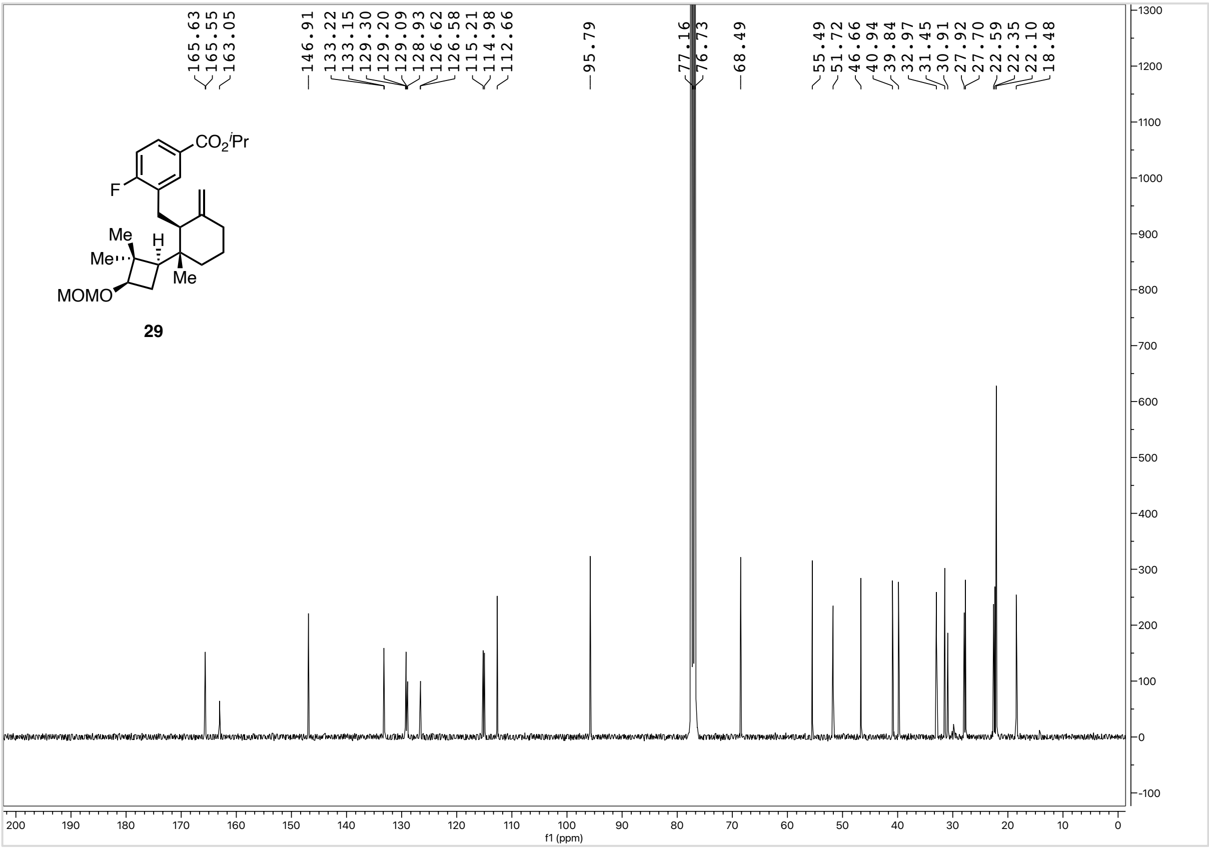

^{1}H NMR (400 MHz, $CDCl_3$) of **S17**

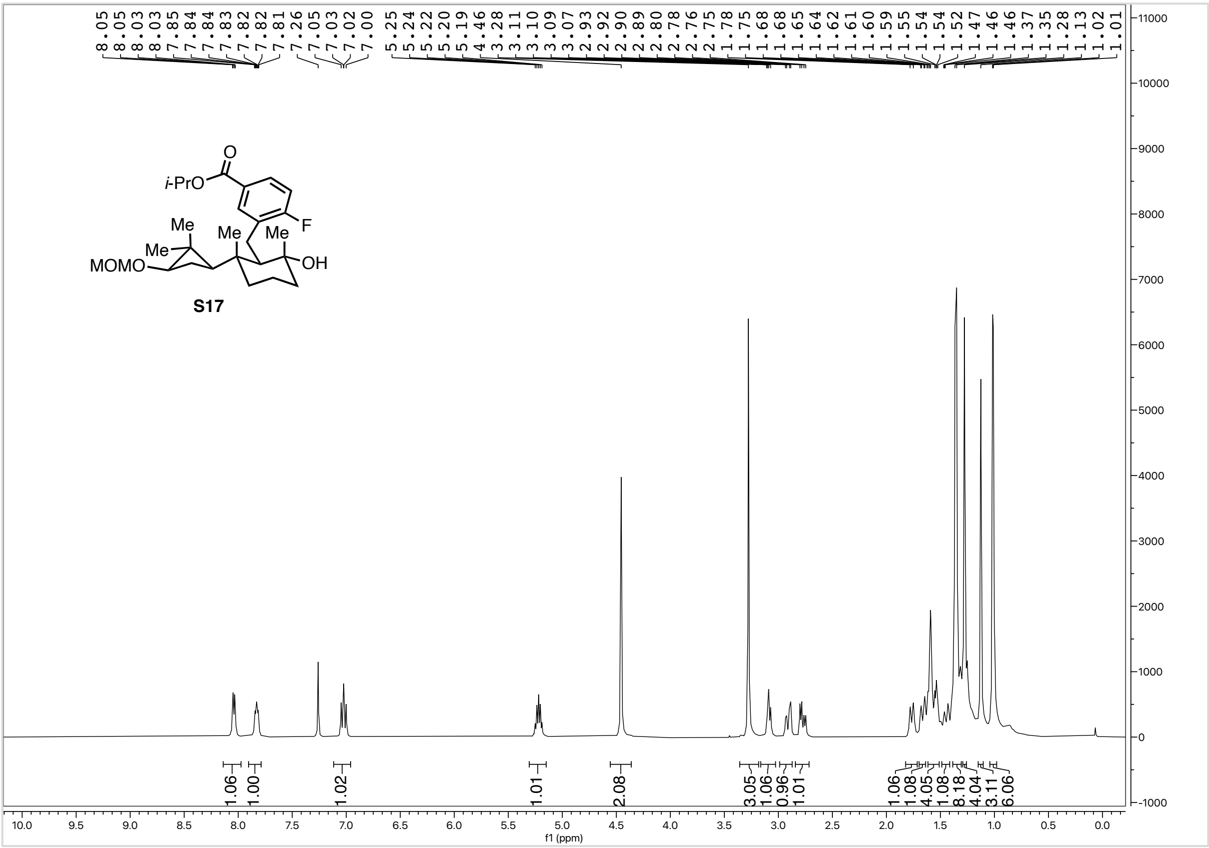

^{13}C NMR (101 MHz, $CDCl_3$) of **S17**

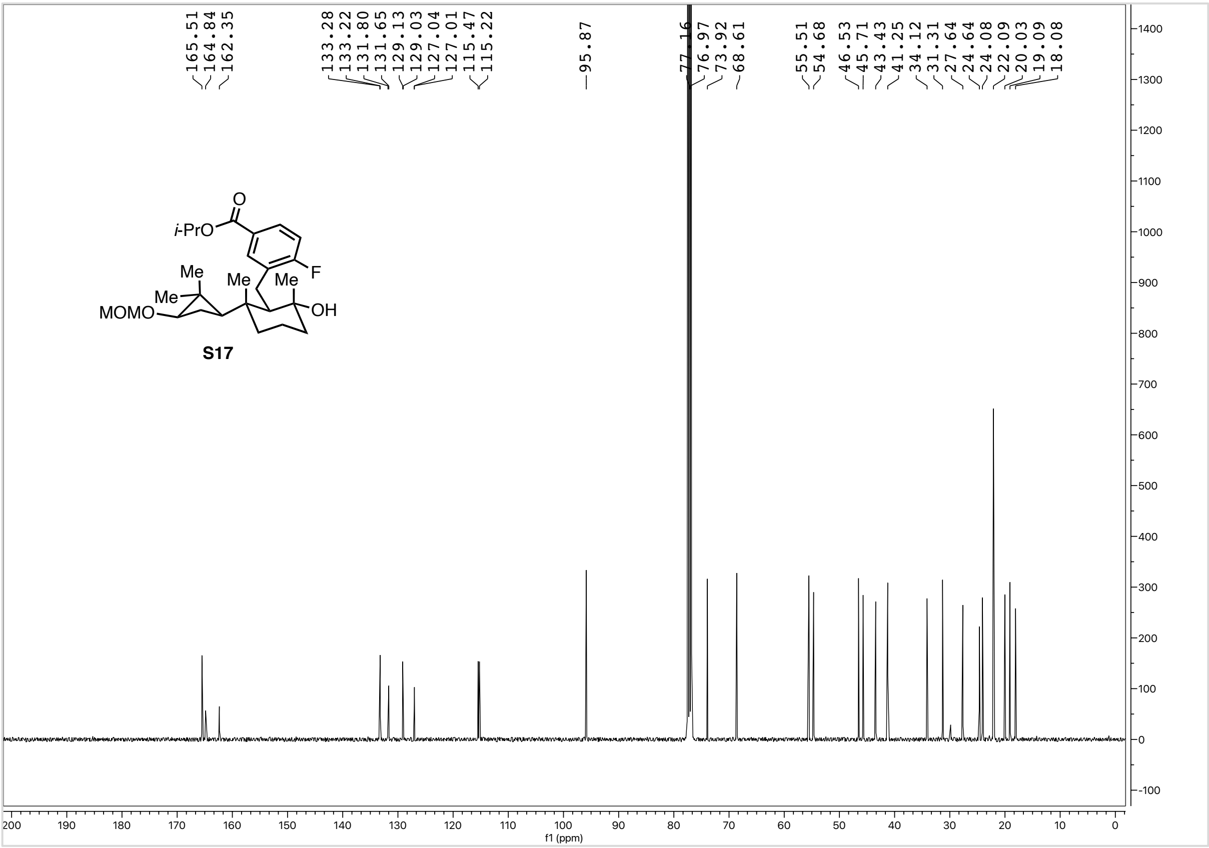

^{1}H NMR (400 MHz, $CDCl_3$) of **30**

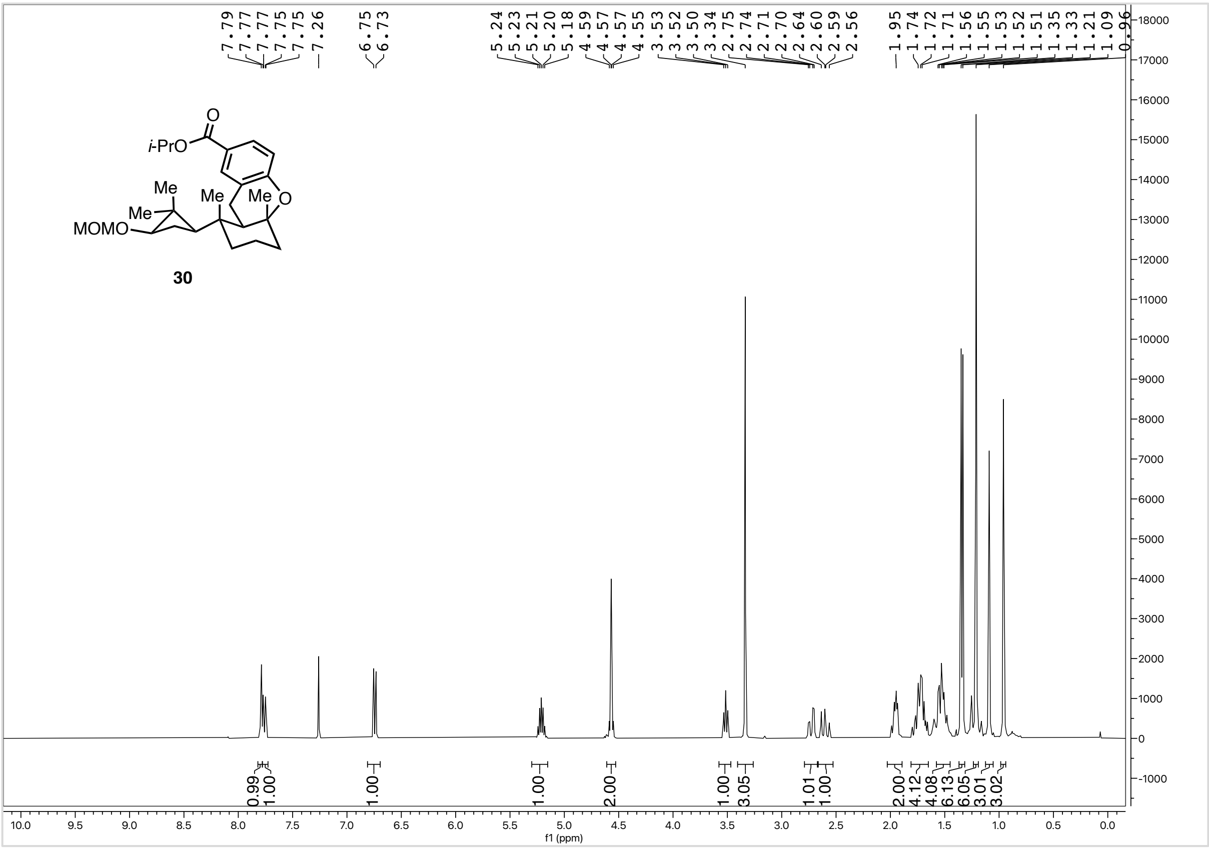

^{13}C NMR (101 MHz, $CDCl_3$) of **30**

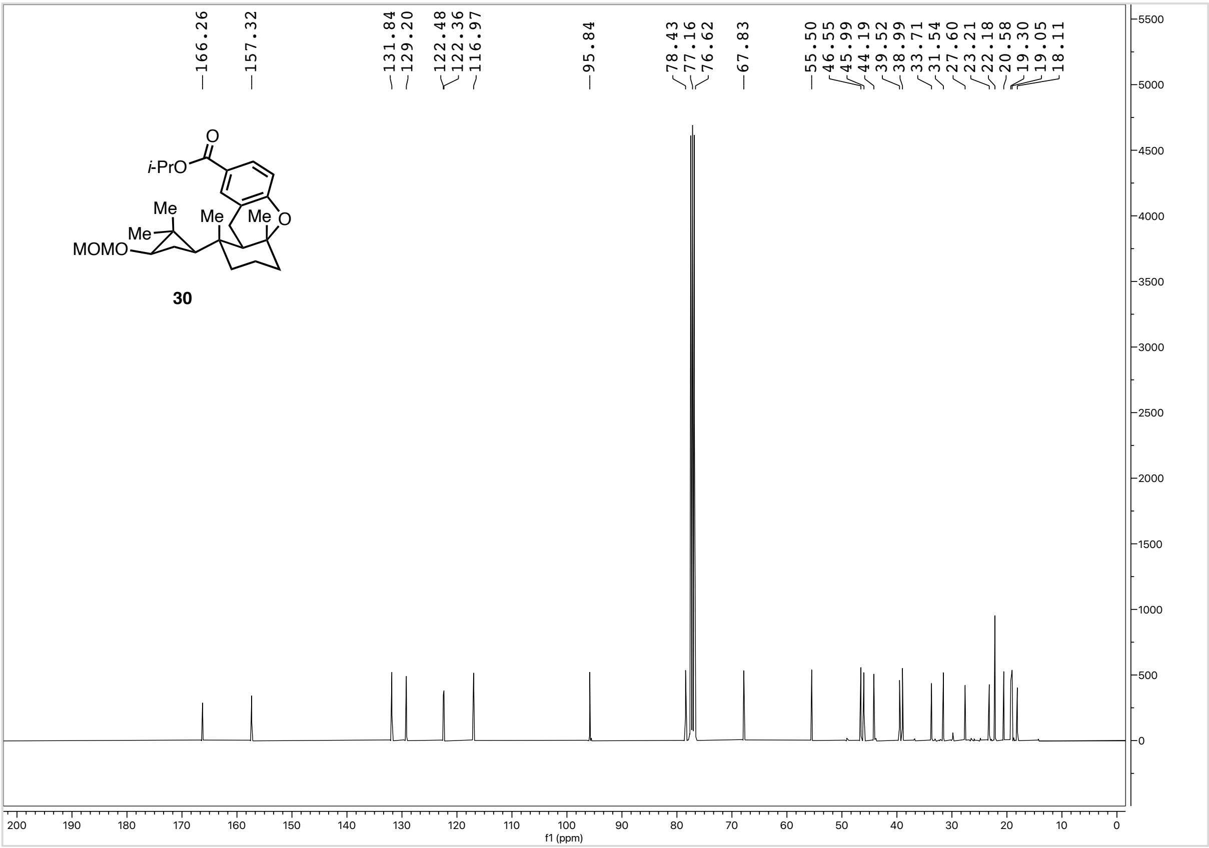

^{1}H NMR (400 MHz, $CDCl_3$) of **31**

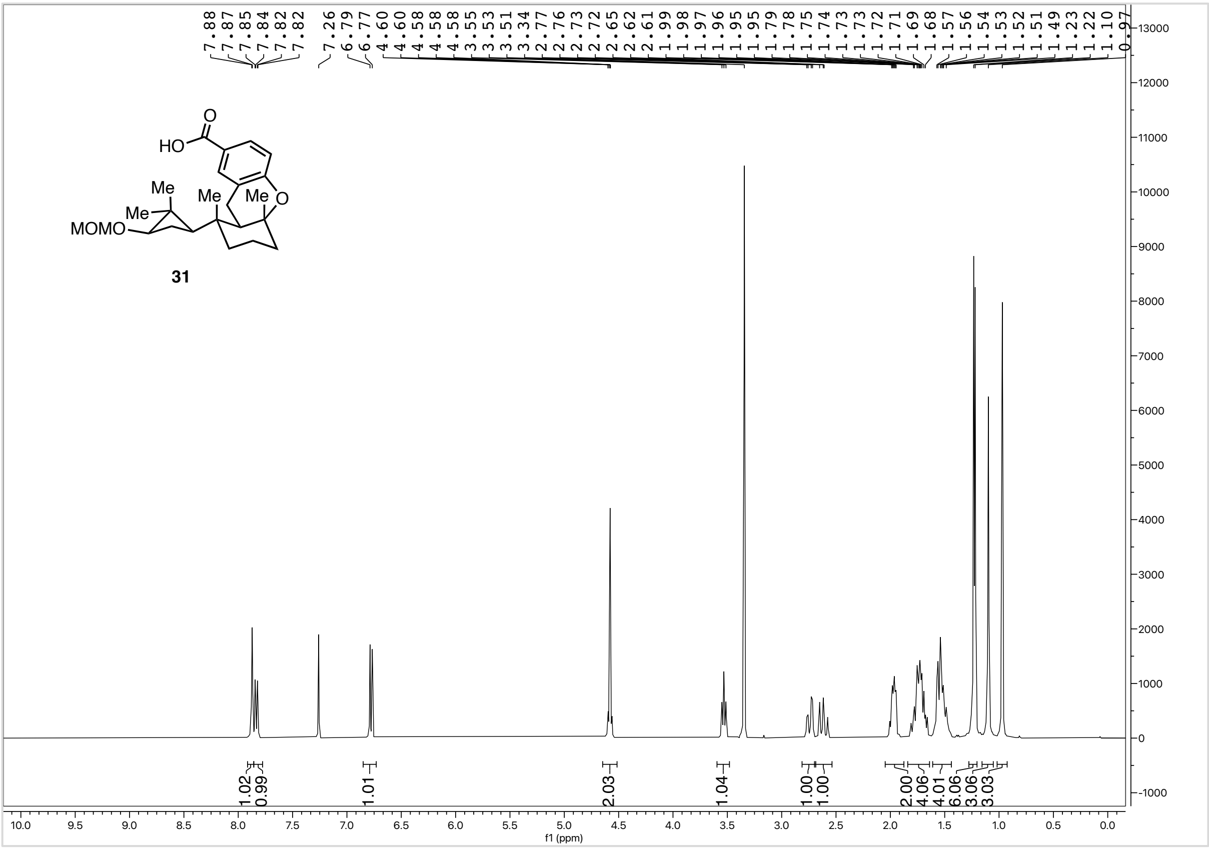

^{13}C NMR (101 MHz, $CDCl_3$) of **31**

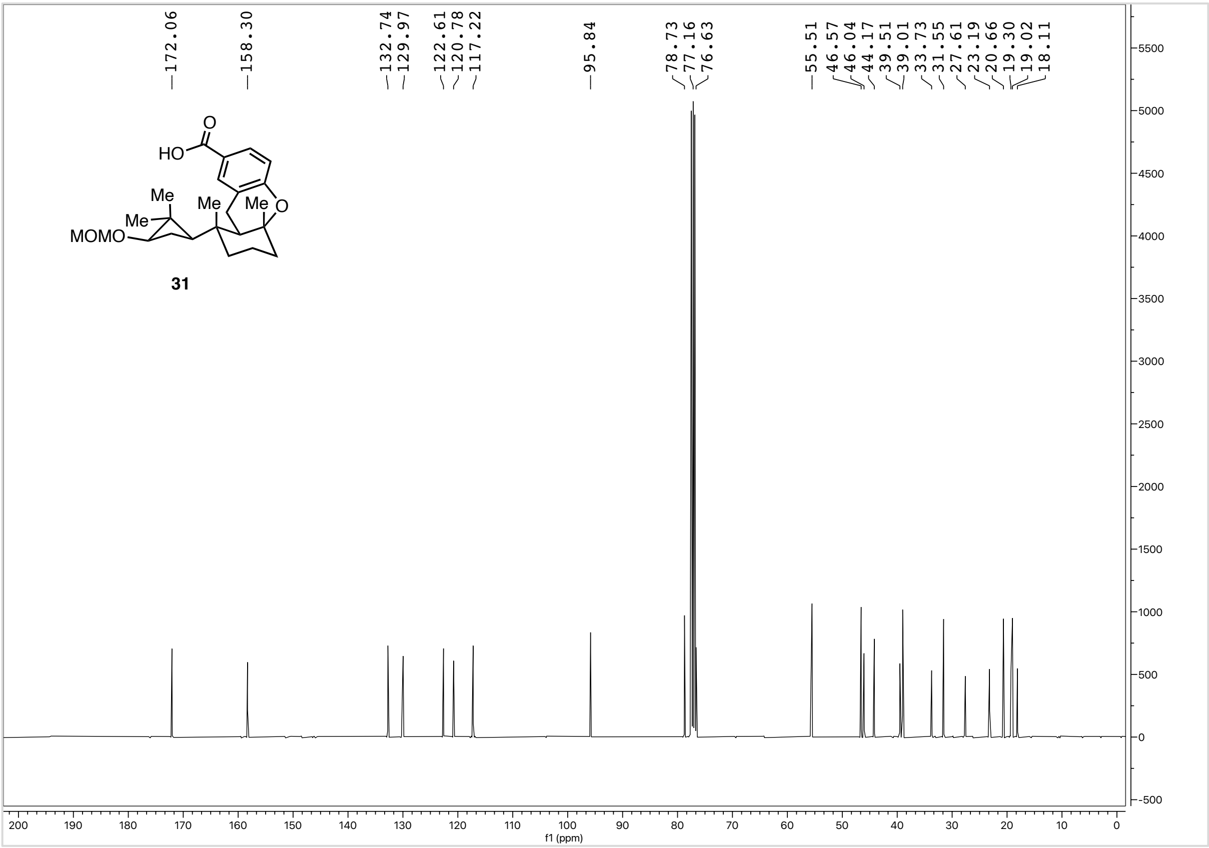

NOESY (400 MHz, CDCl3) of **31**

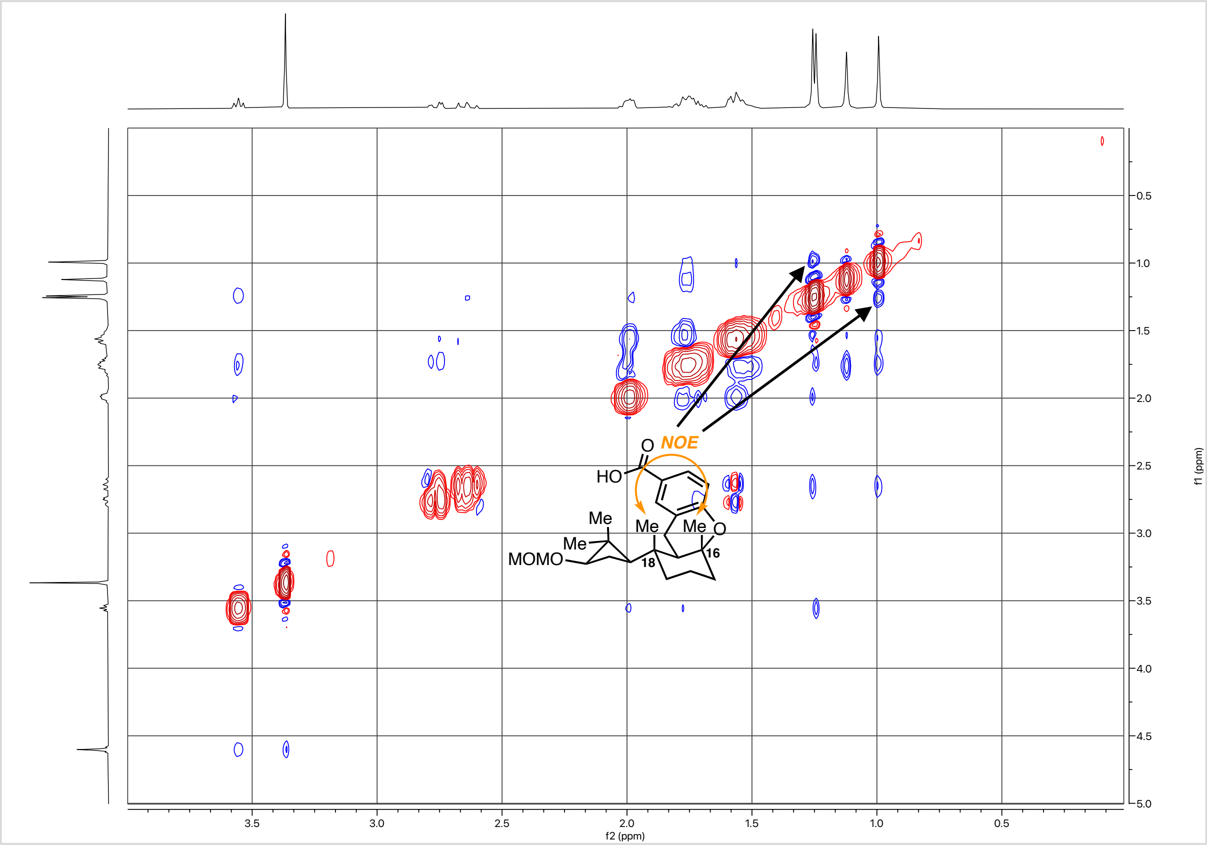

^{1}H NMR (400 MHz, $CDCl_3$) of **33**

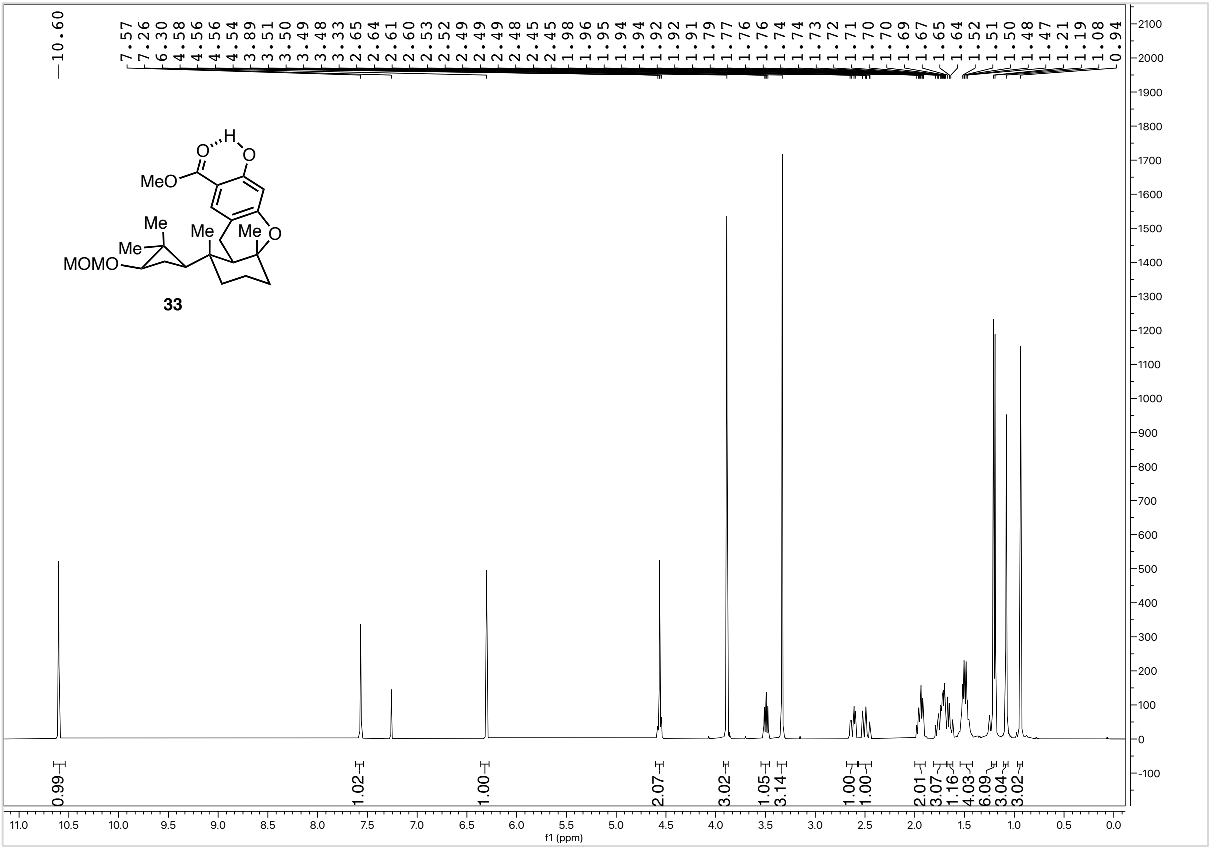

^{13}C NMR (101 MHz, $CDCl_3$) of **33**

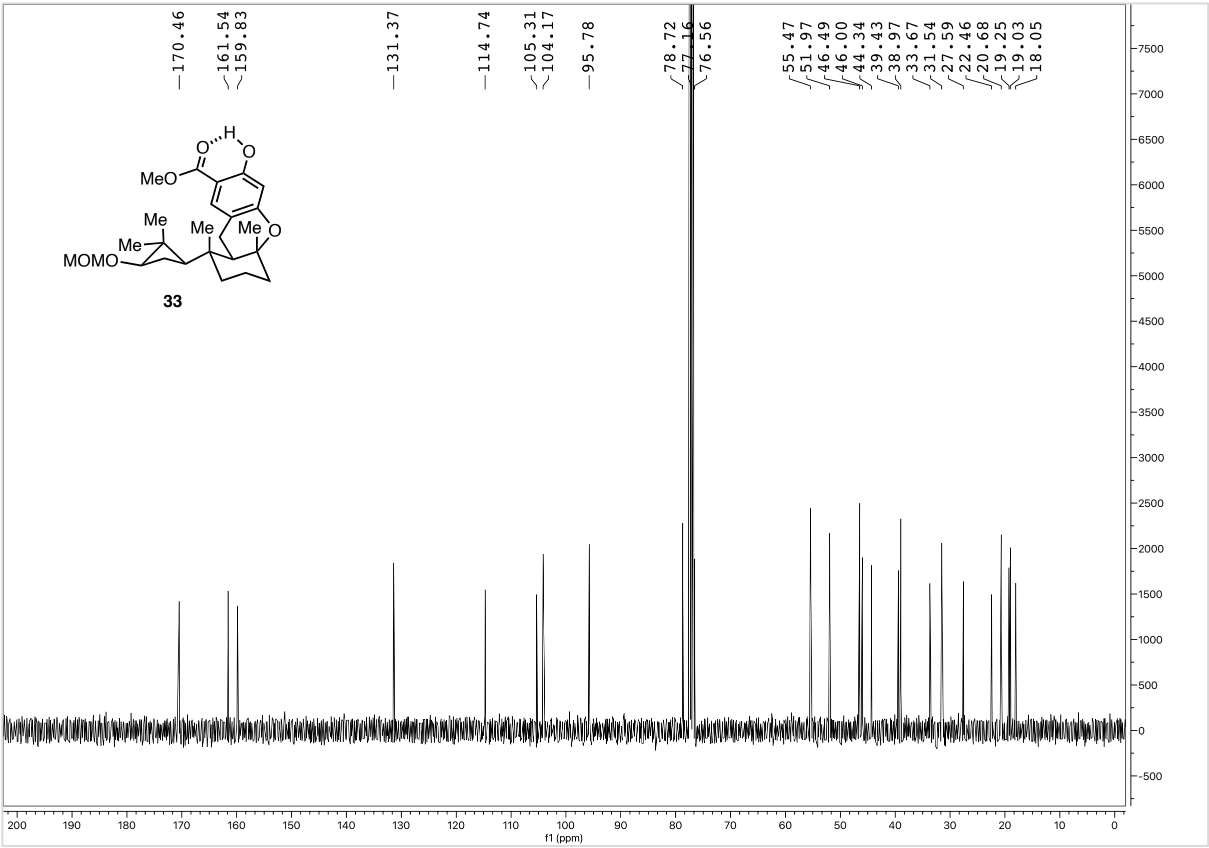

^{1}H NMR (400 MHz, $CDCl_3$) of **S18**

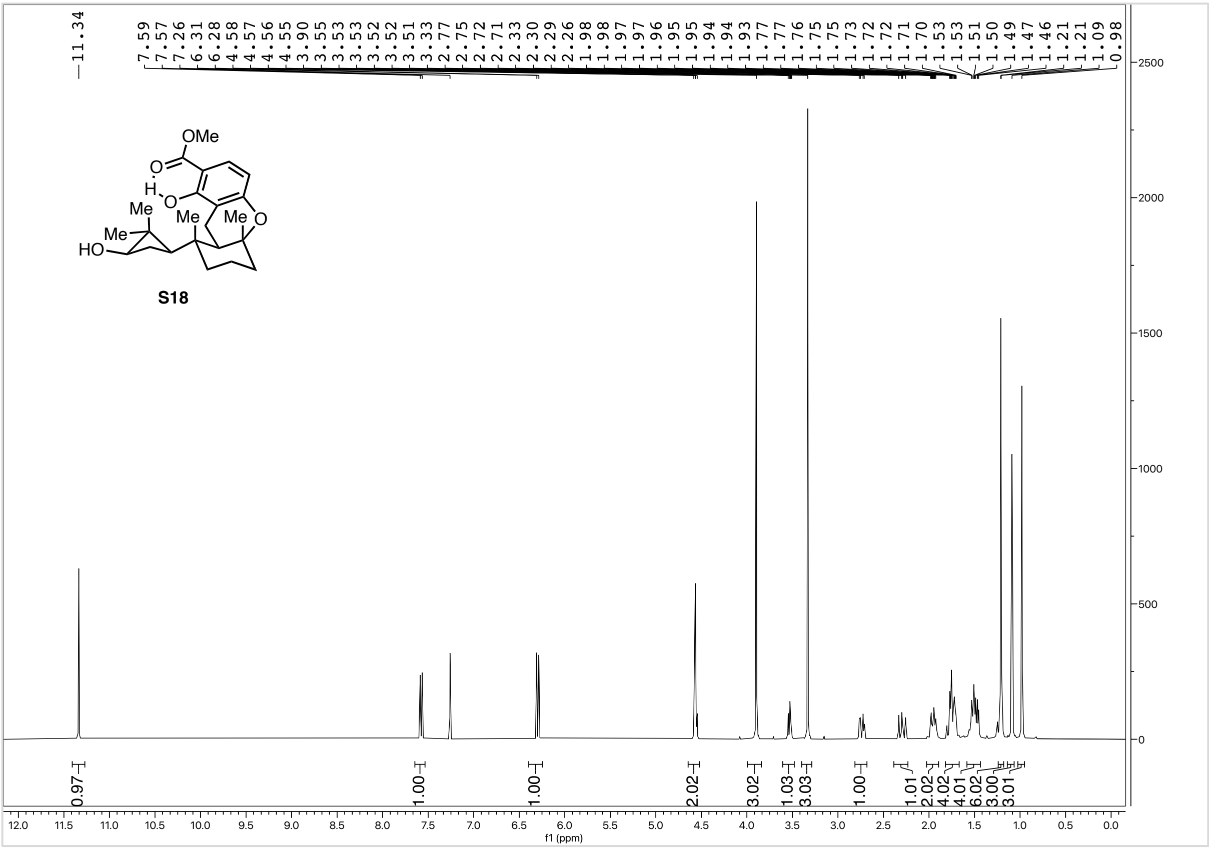

^{13}C NMR (101 MHz, $CDCl_3$) of **S18**

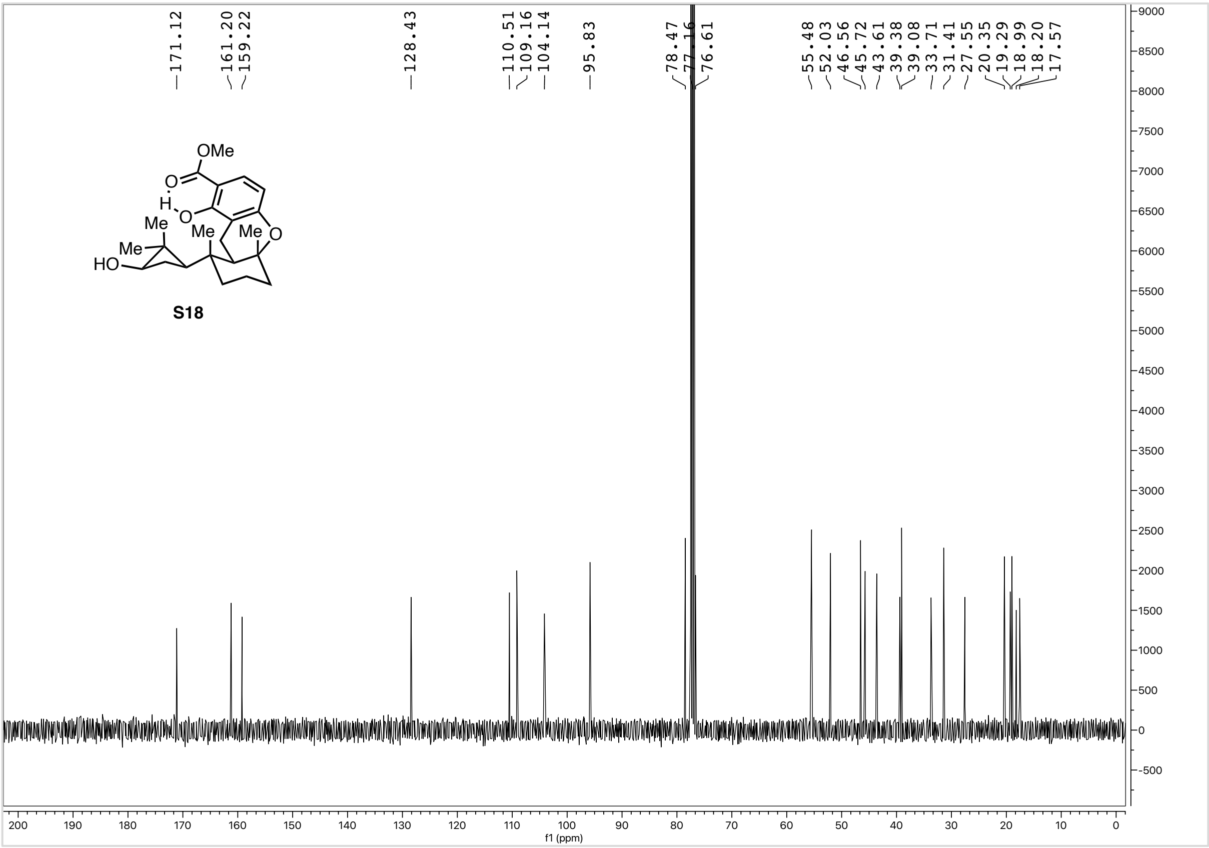

^{1}H NMR (400 MHz, $CDCl_3$) of **S19**

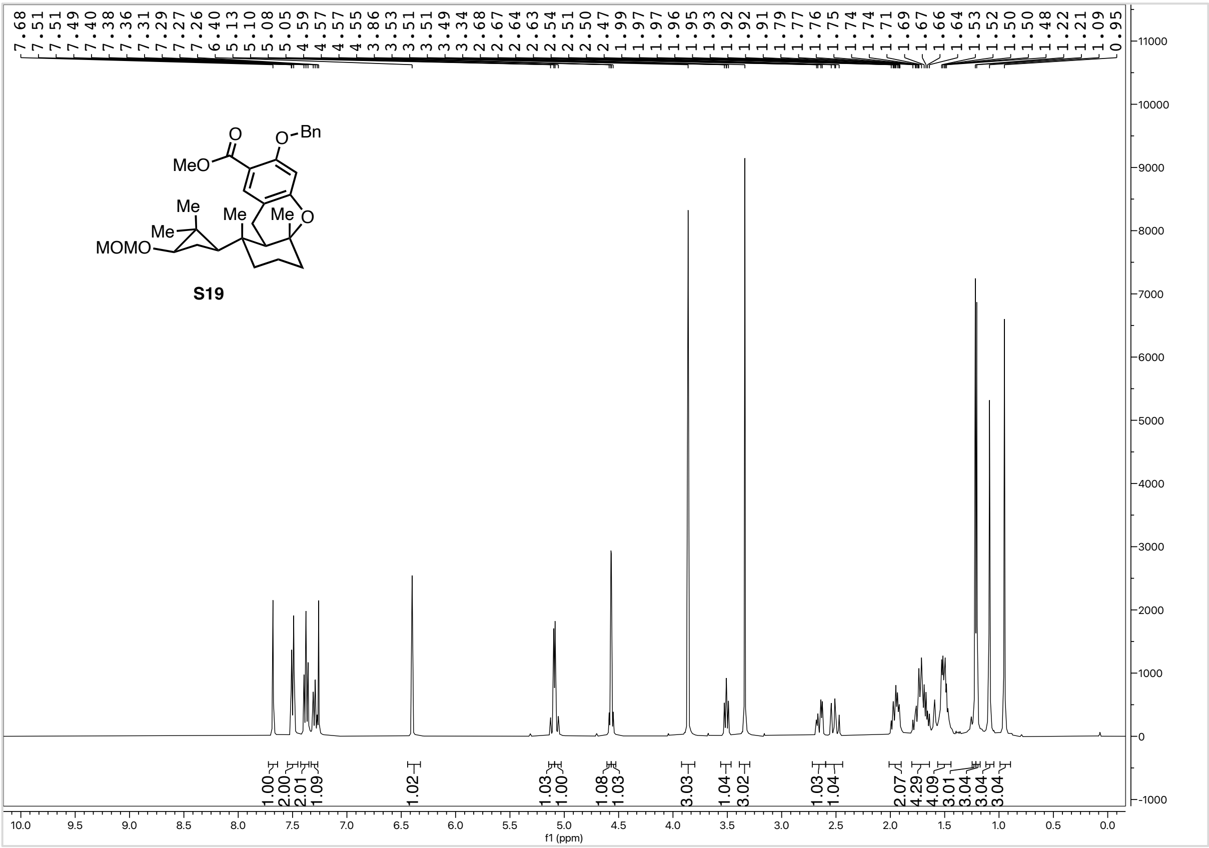

^{13}C NMR (101 MHz, $CDCl_3$) of **S19**

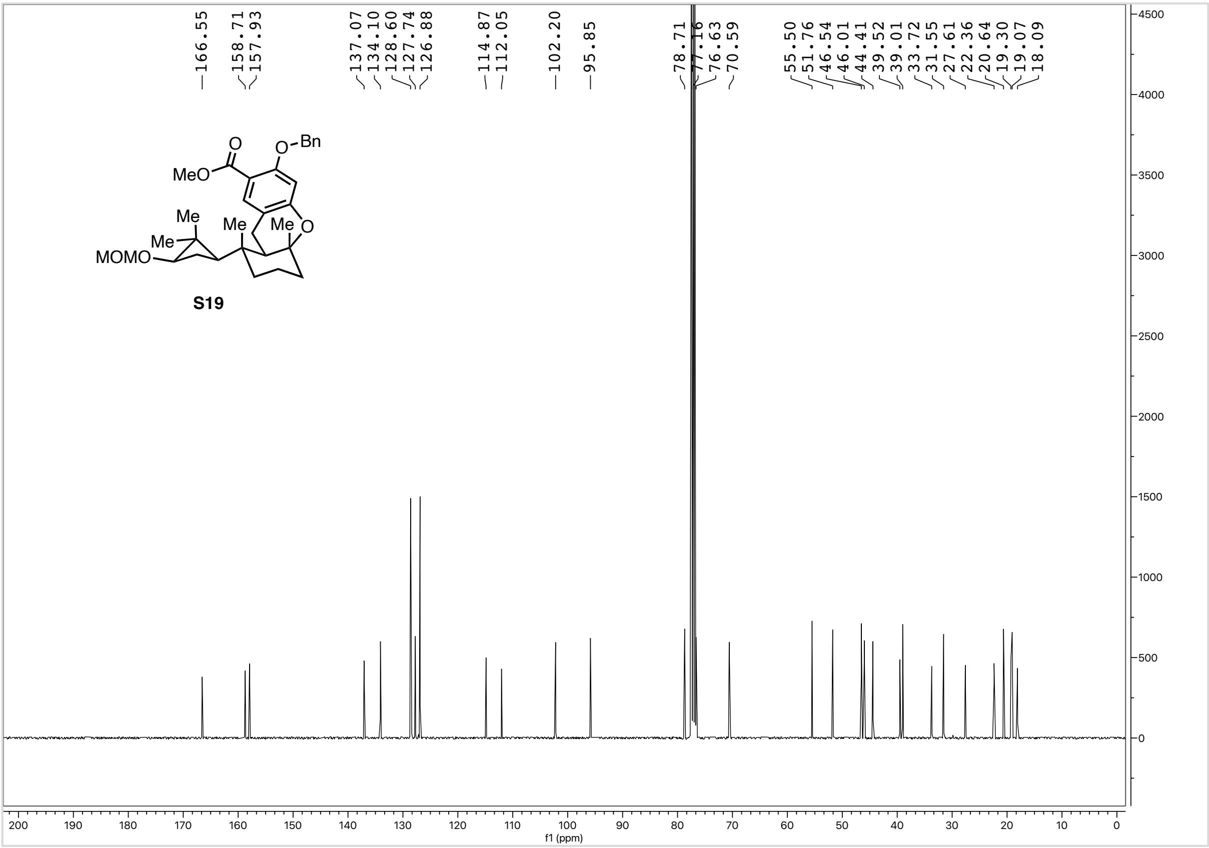

^{1}H NMR (400 MHz, $CDCl_3$) of **34**

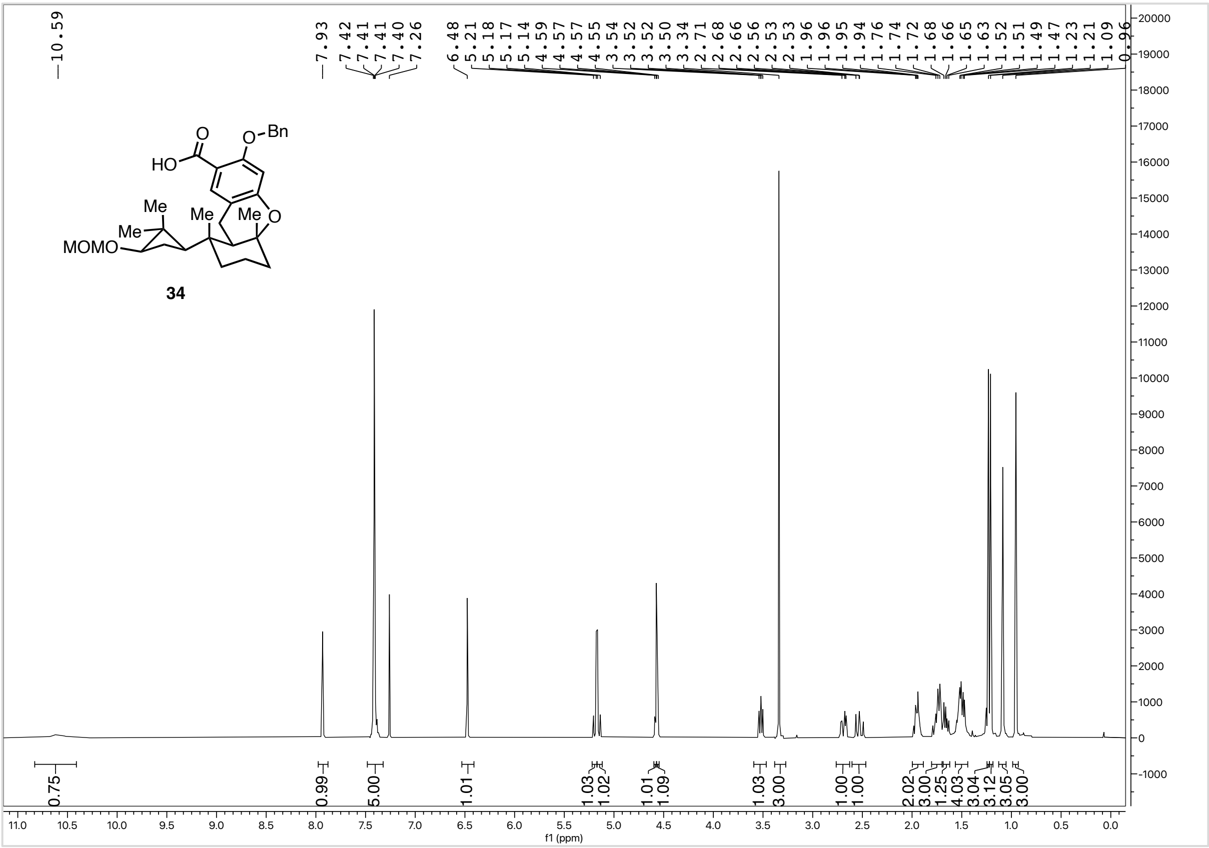

^{13}C NMR (101 MHz, $CDCl_3$) of **34**

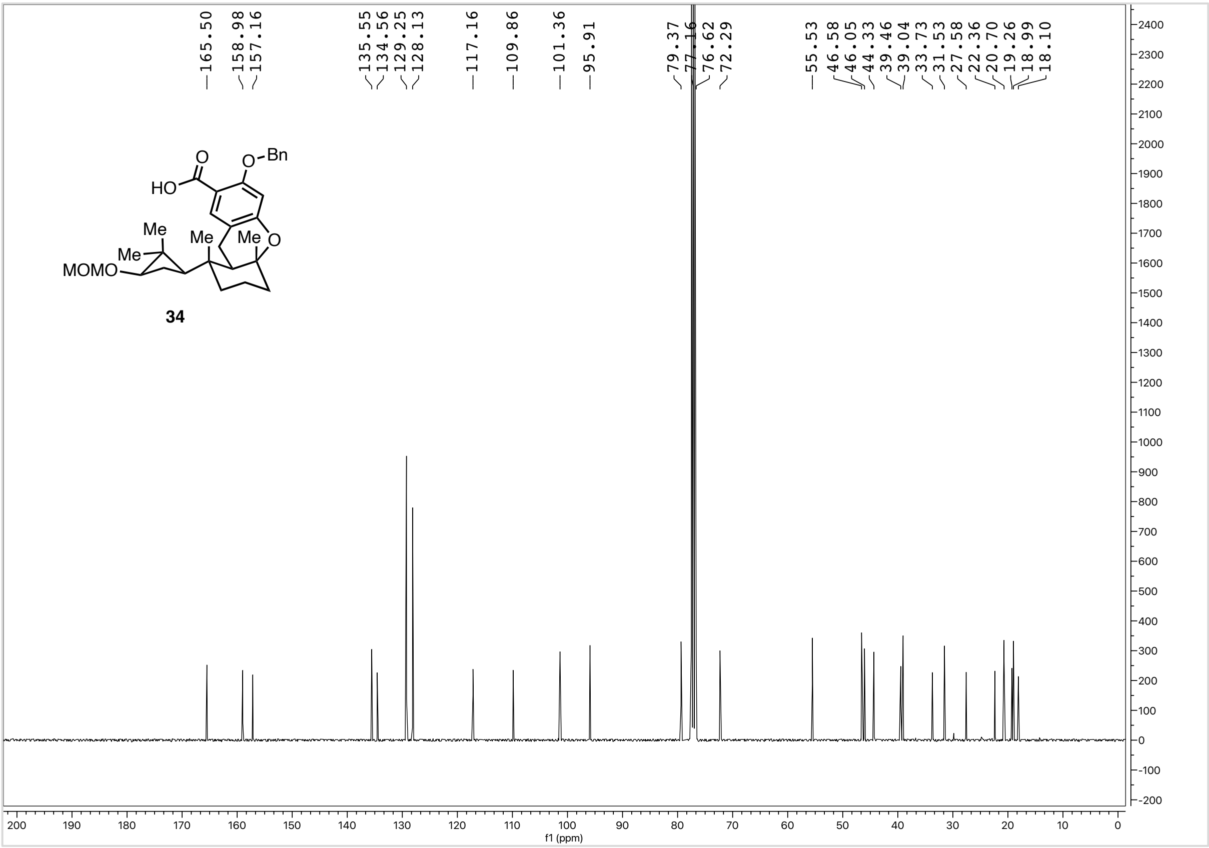

^{1}H NMR (400 MHz, $CDCl_3$) of **37**

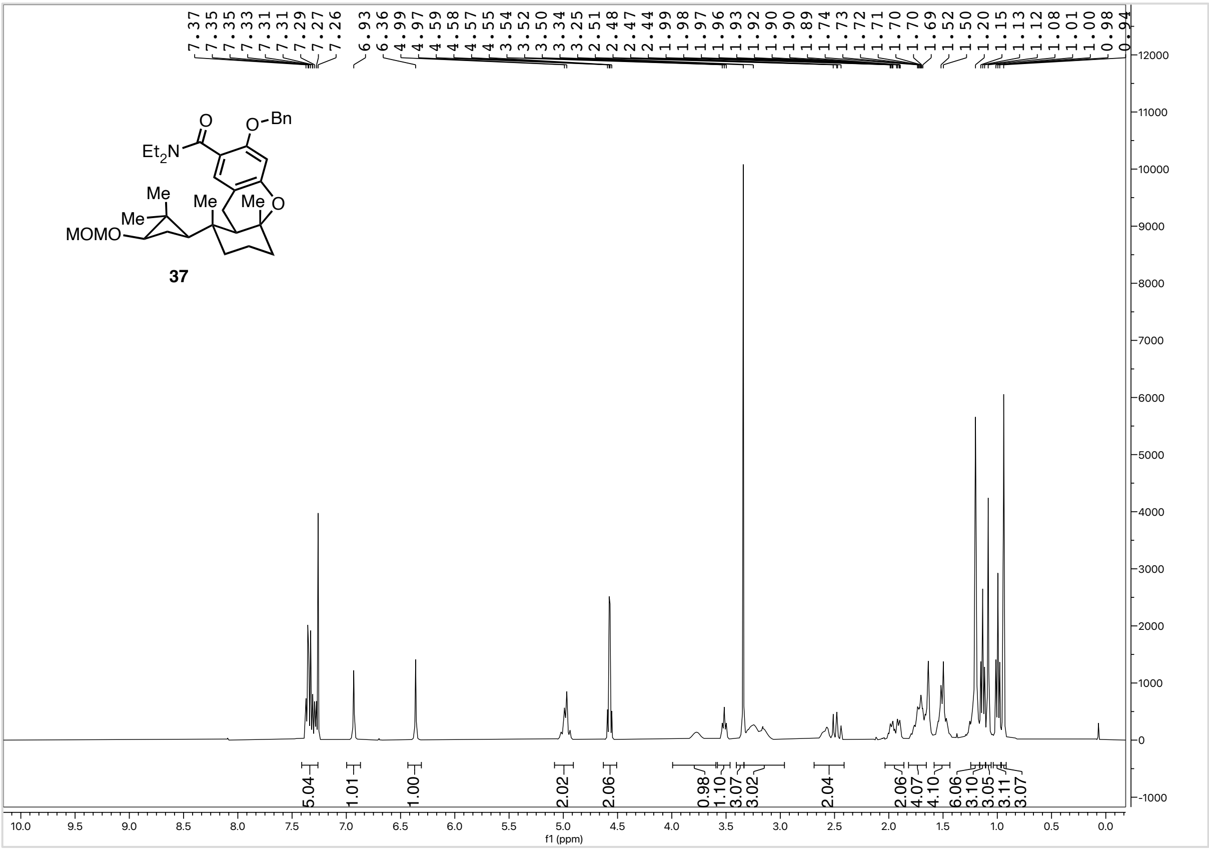

^{13}C NMR (101 MHz, $CDCl_3$) of **37**

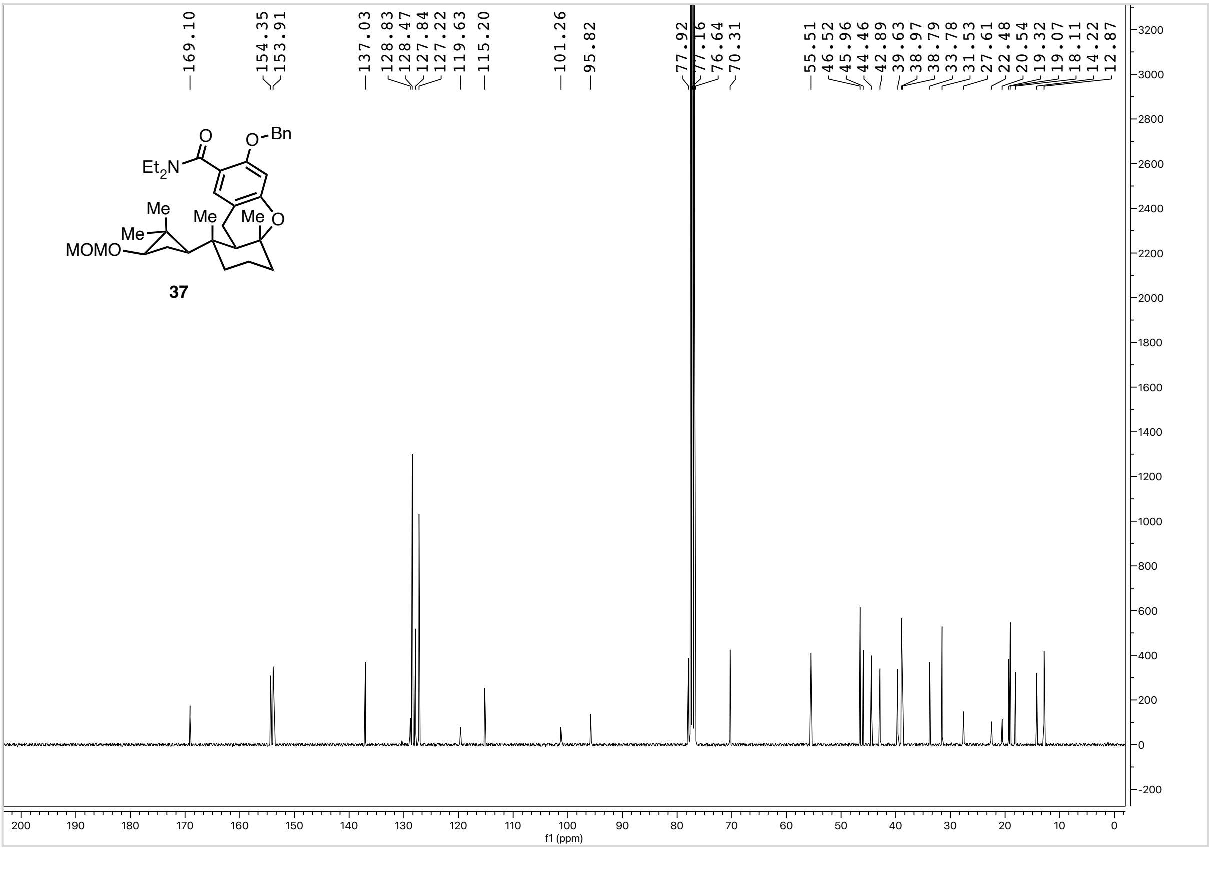

^{1}H NMR (400 MHz, $CDCl_3$) of **39**

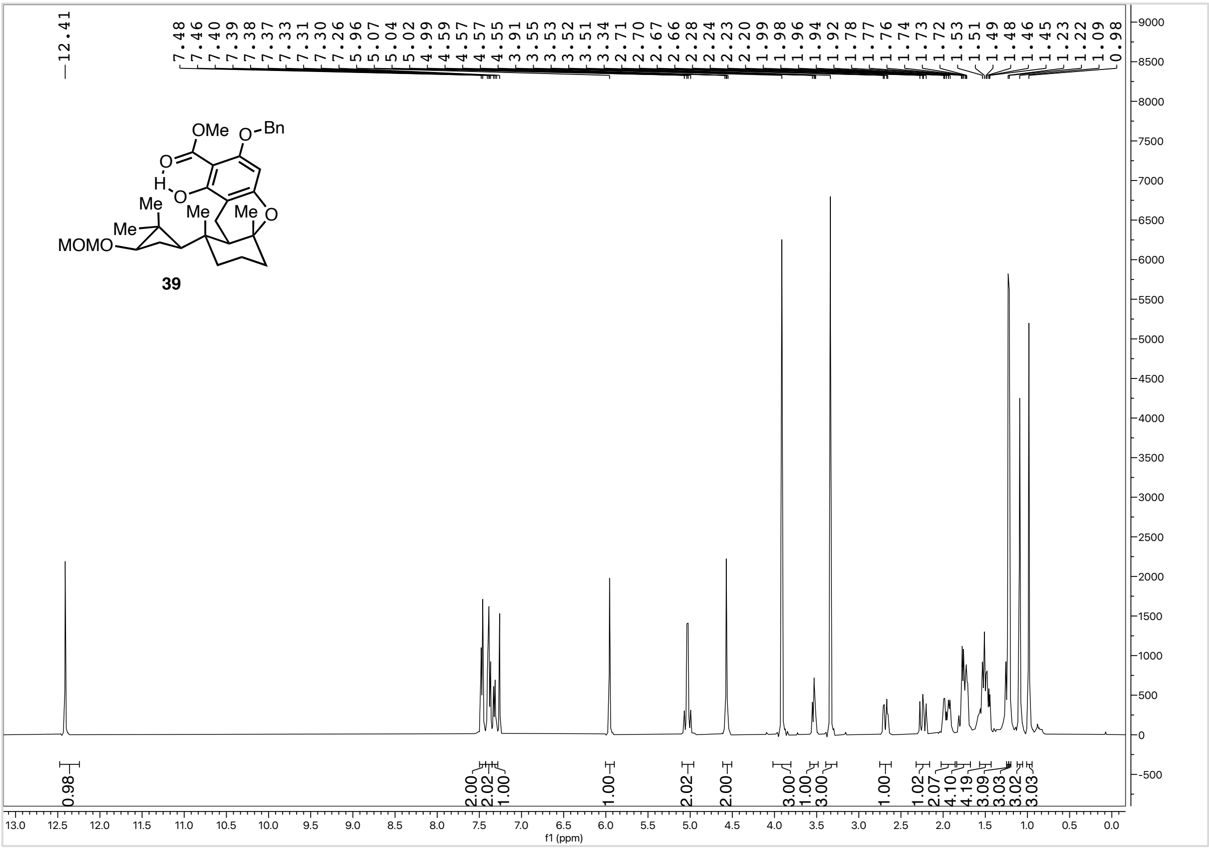

^{13}C NMR (101 MHz, $CDCl_3$) of **39**

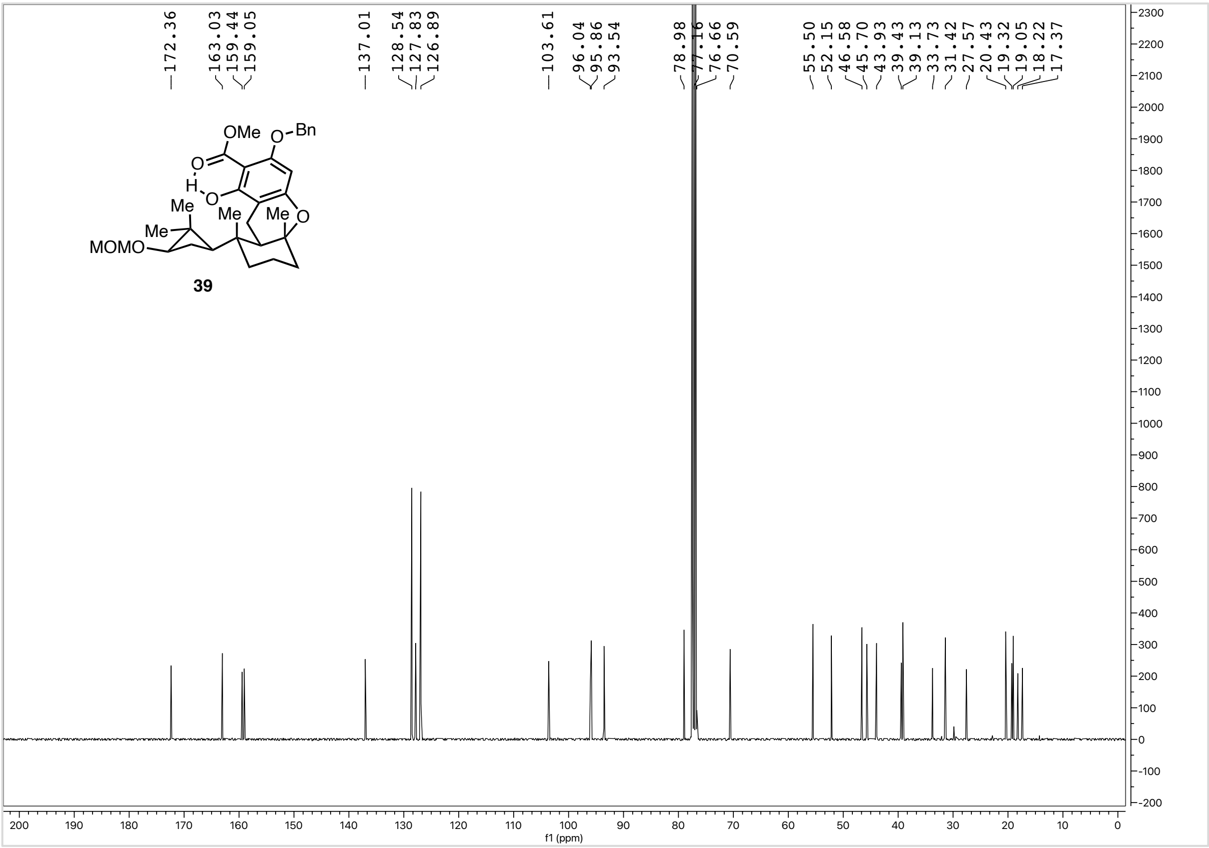

^{1}H NMR (400 MHz, $CDCl_3$) of **S20**

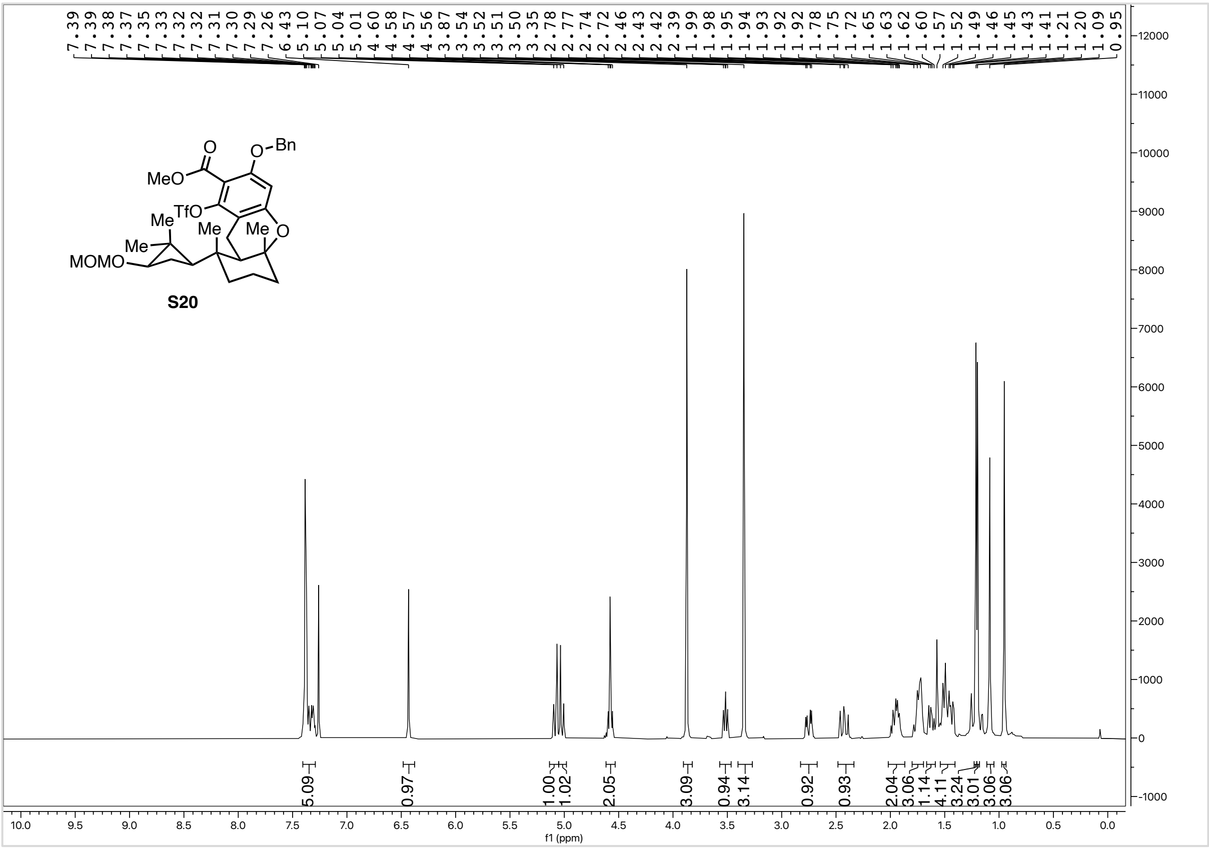

^{13}C NMR (101 MHz, $CDCl_3$) of **S20**

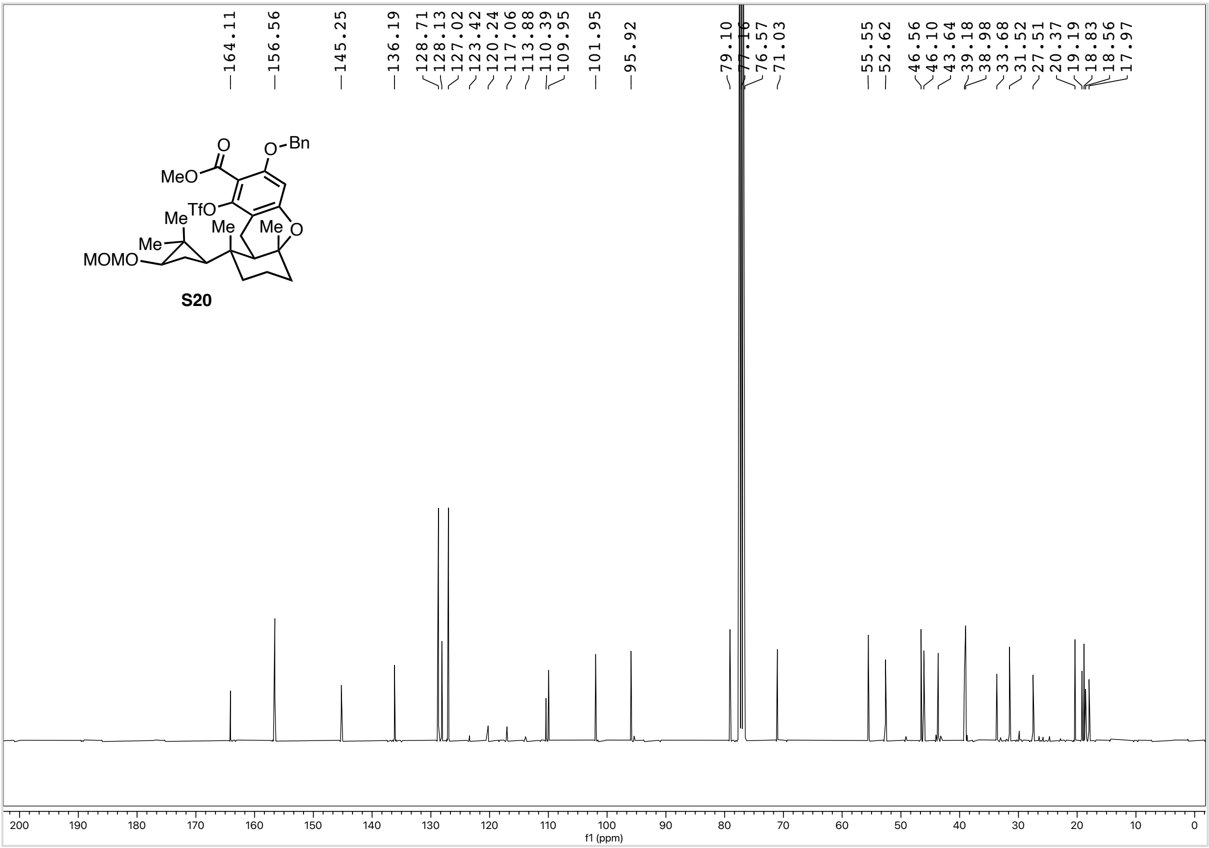

^{1}H NMR (400 MHz, $CDCl_3$) of **40**

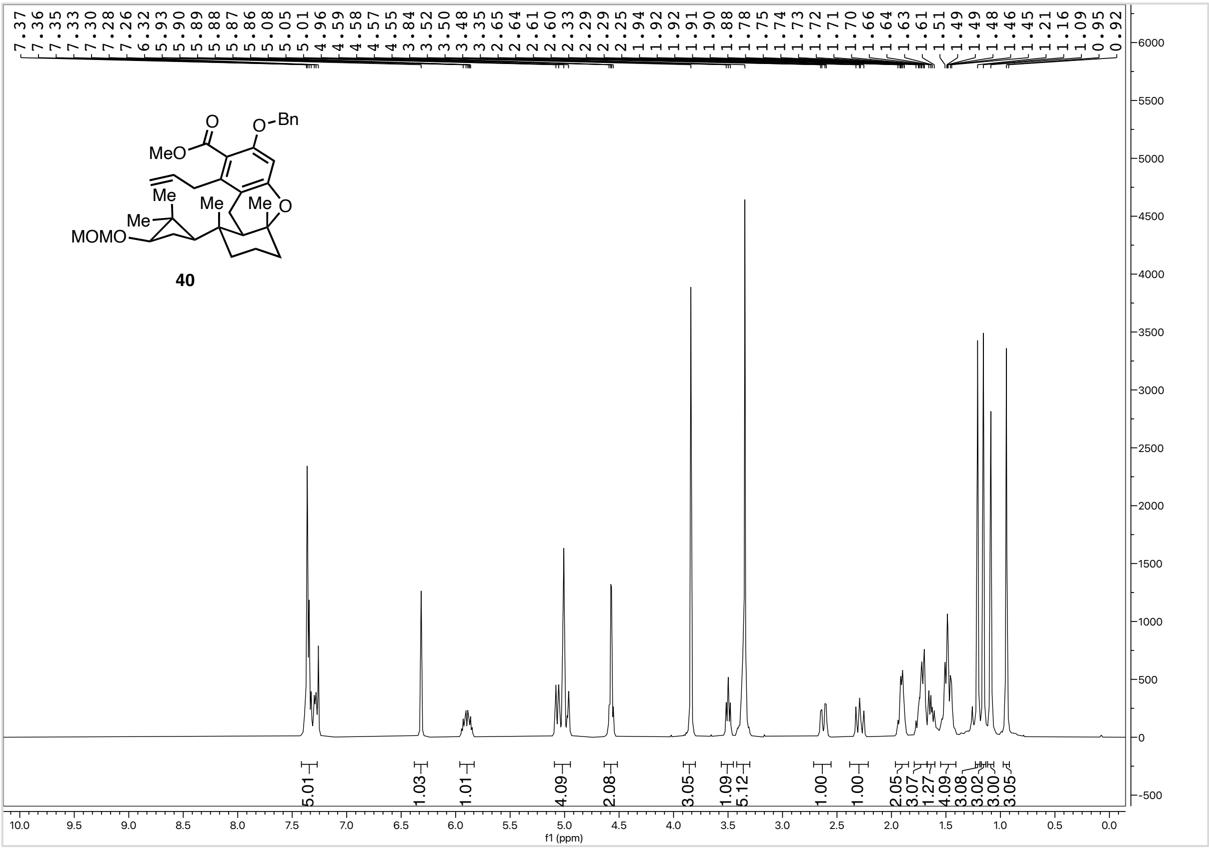

^{13}C NMR (101 MHz, $CDCl_3$) of **40**

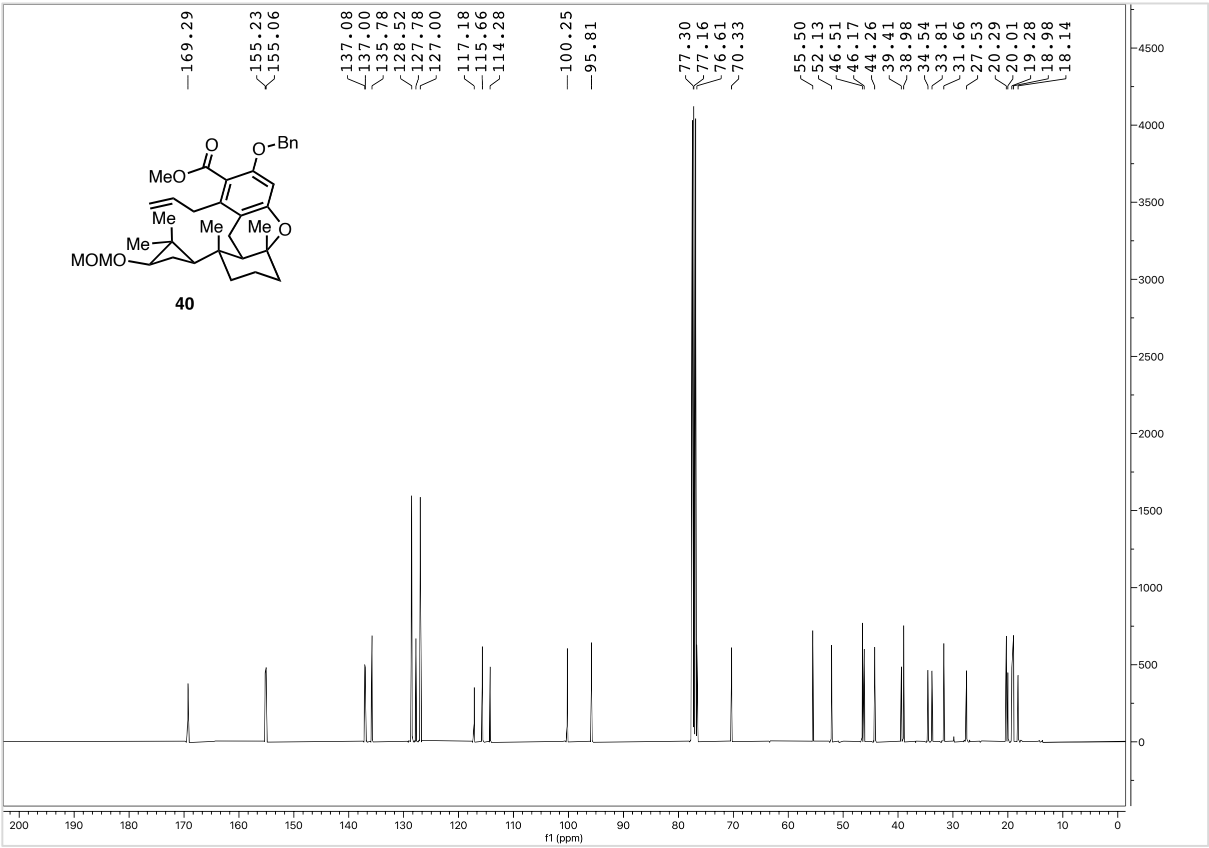

^{1}H NMR (400 MHz, $CDCl_3$) of **S21**

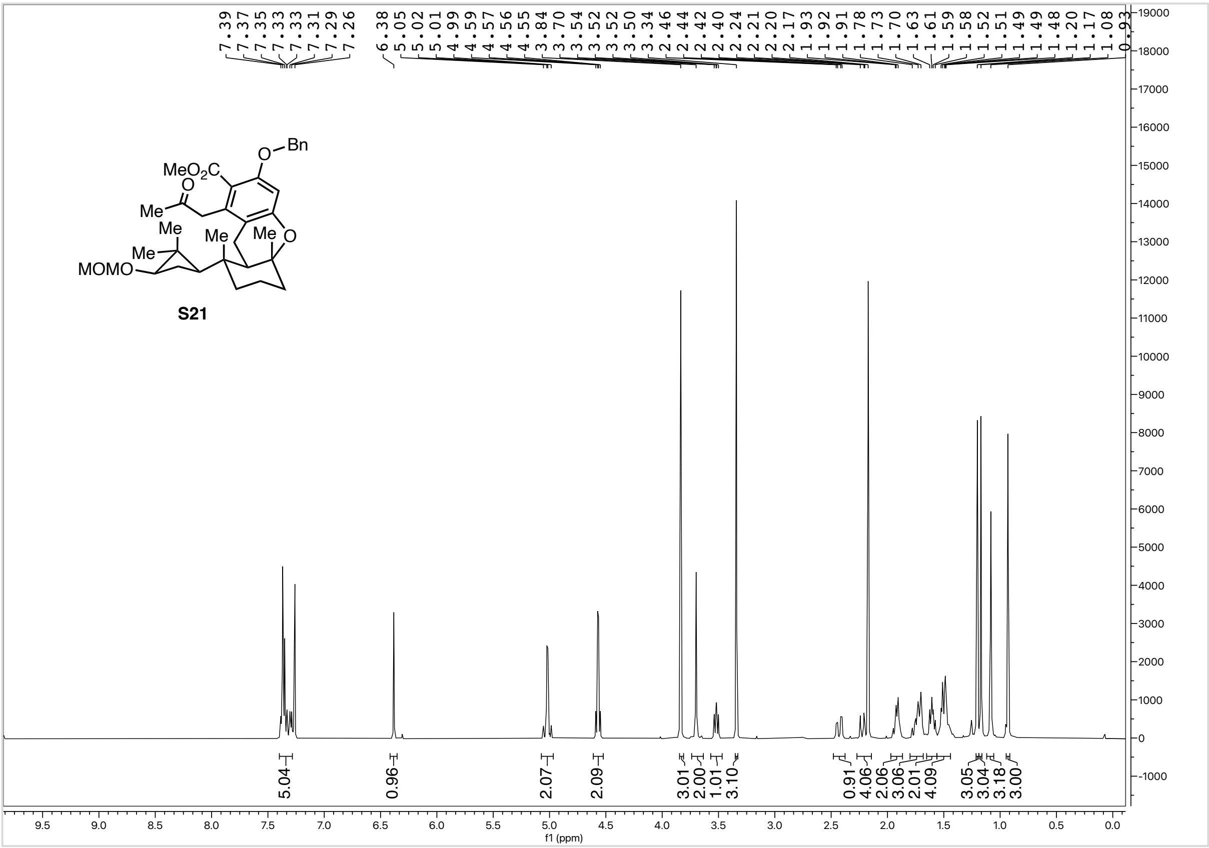

^{13}C NMR (101 MHz, $CDCl_3$) of **S21**

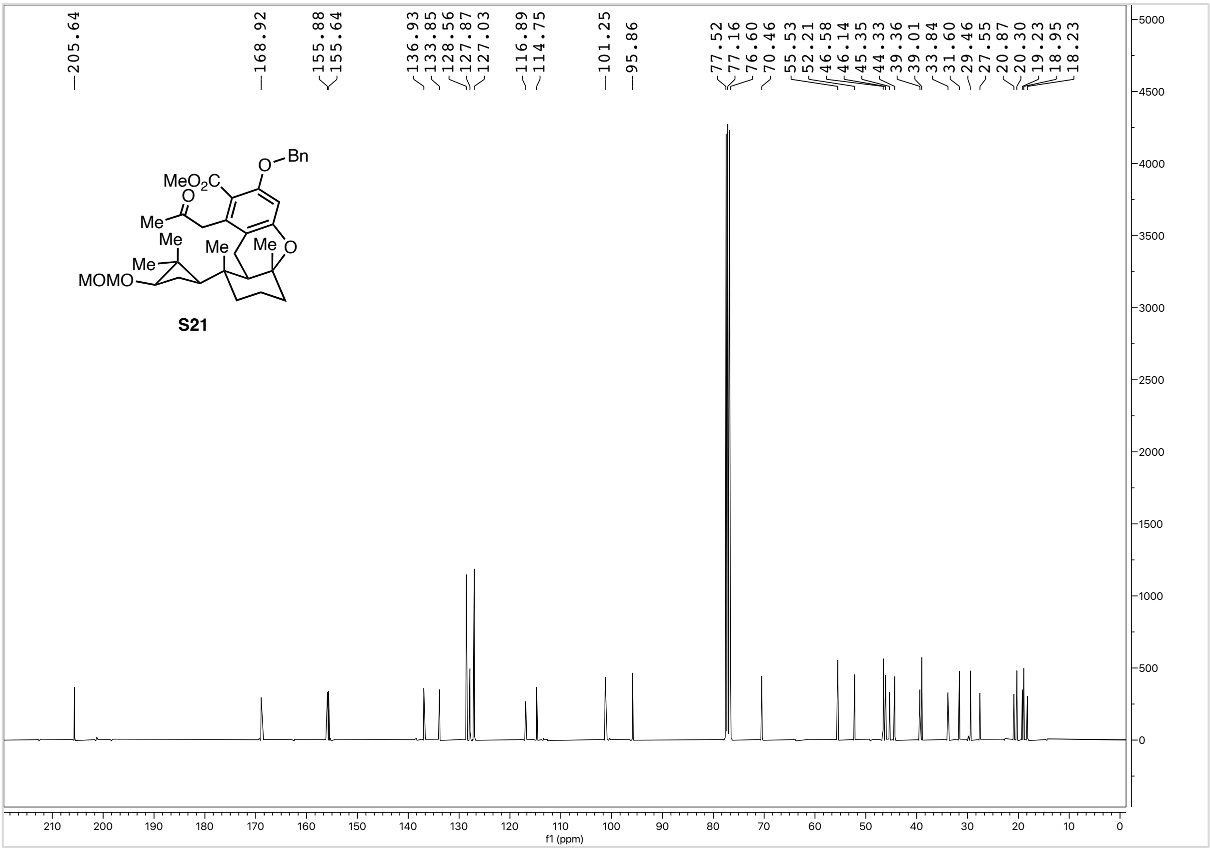

^{1}H NMR (400 MHz, $CDCl_3$) of **38**

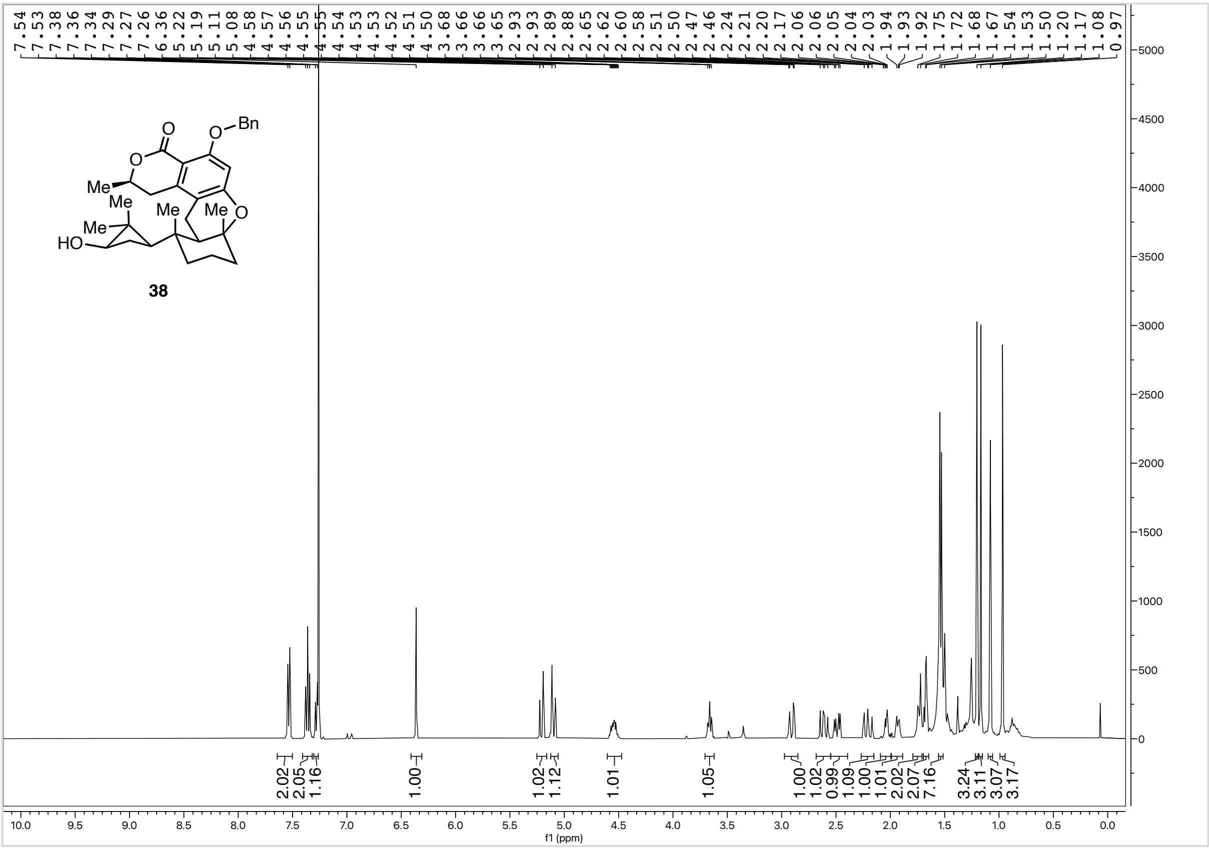

^{13}C NMR (151 MHz, $CDCl_3$) of **38**

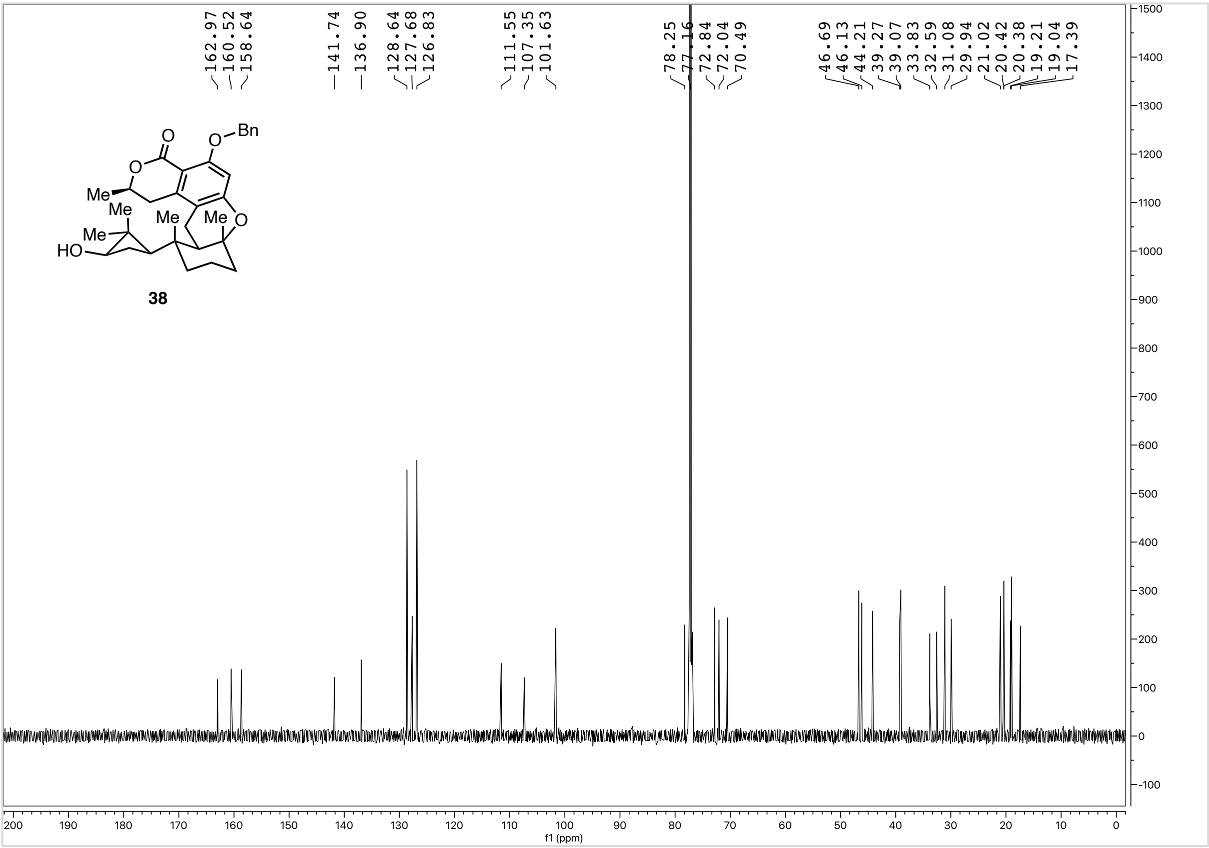

^{1}H NMR (400 MHz, $CDCl_3$) of **S22**

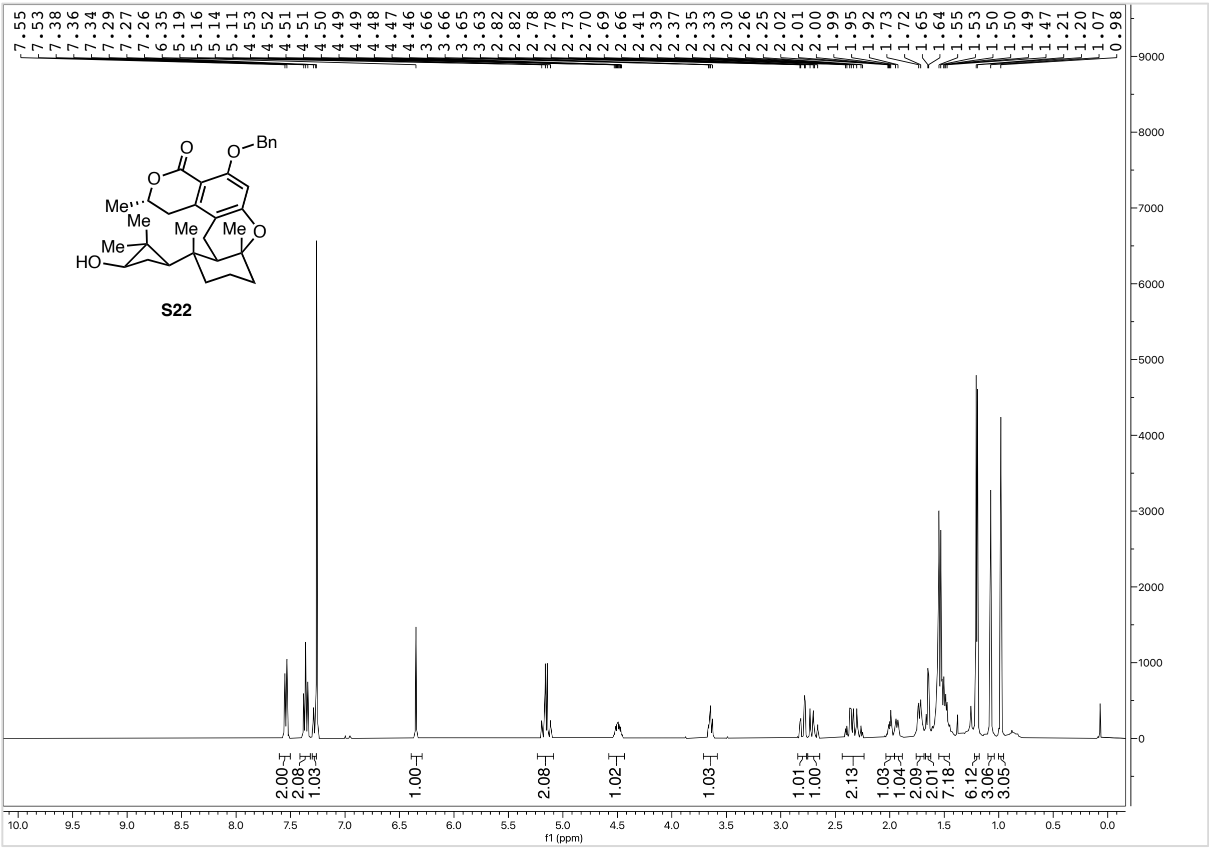

^{13}C NMR (151 MHz, $CDCl_3$) of **S22**

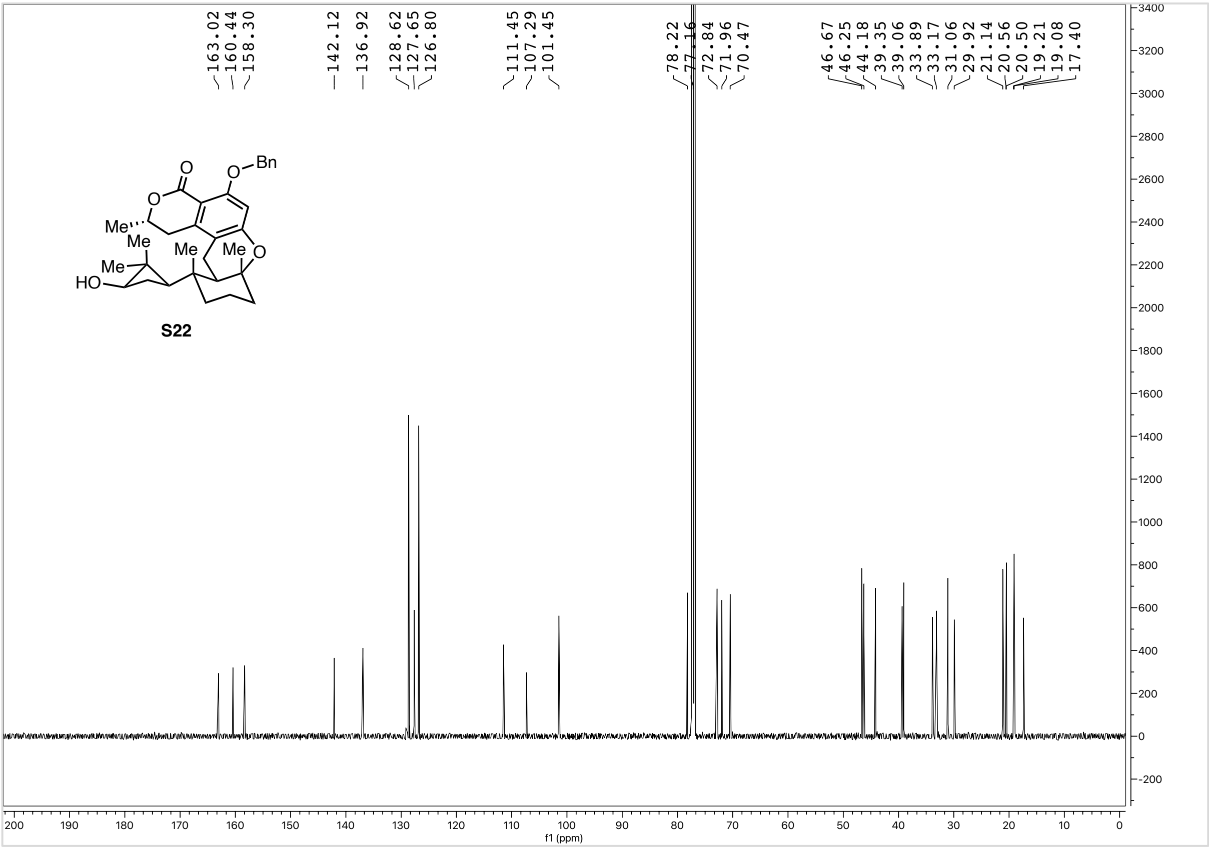

^{1}H NMR (400 MHz, $CDCl_3$) of talaromyolide D (**4**)

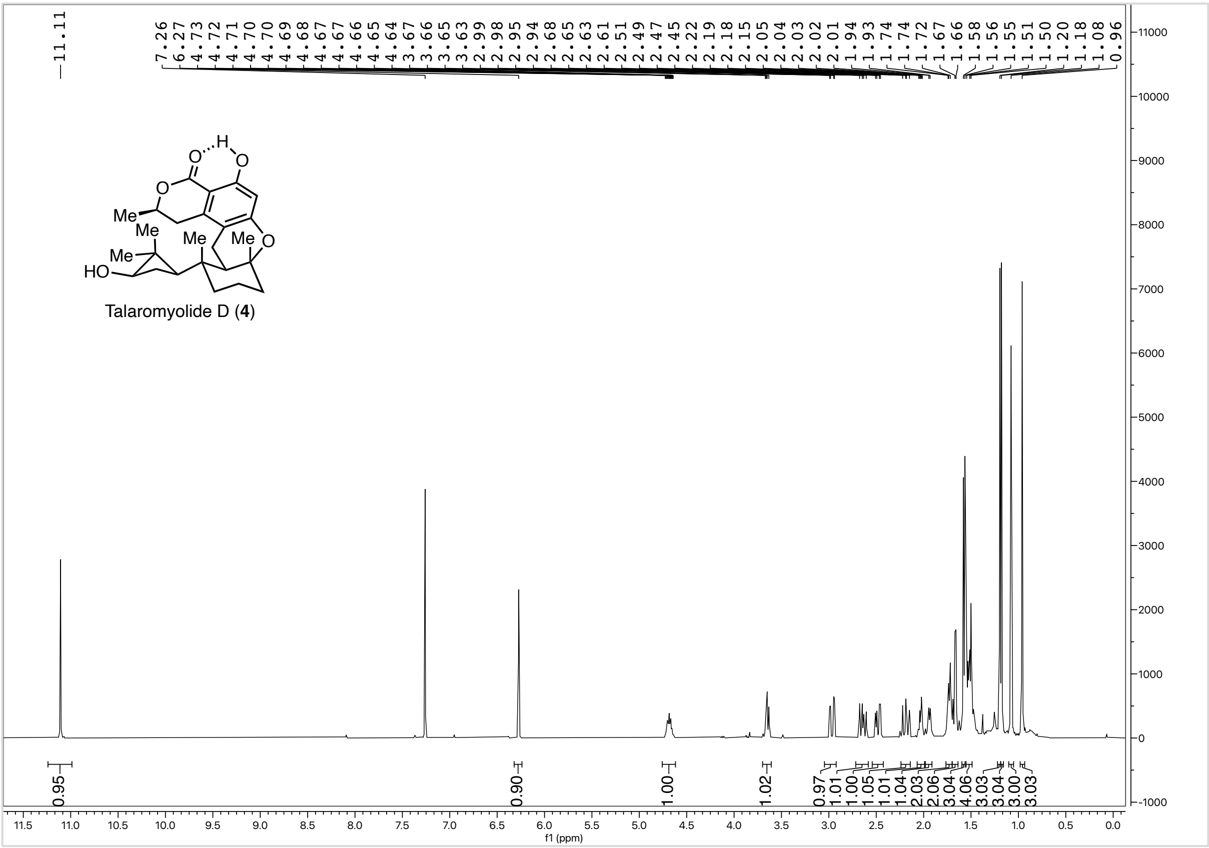

^{13}C NMR (151 MHz, $CDCl_3$) of talaromyolide D (**4**)

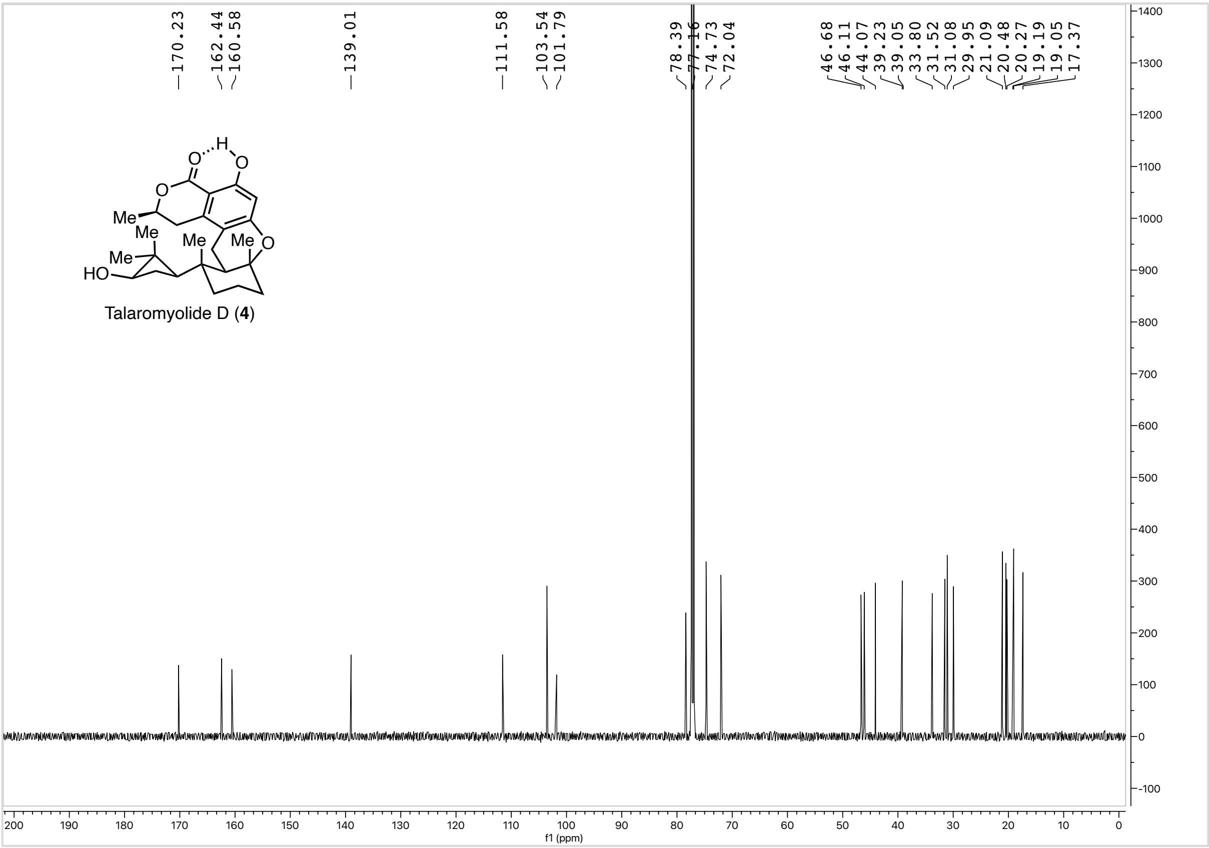

^{1}H NMR (400 MHz, $CDCl_3$) of **S24**

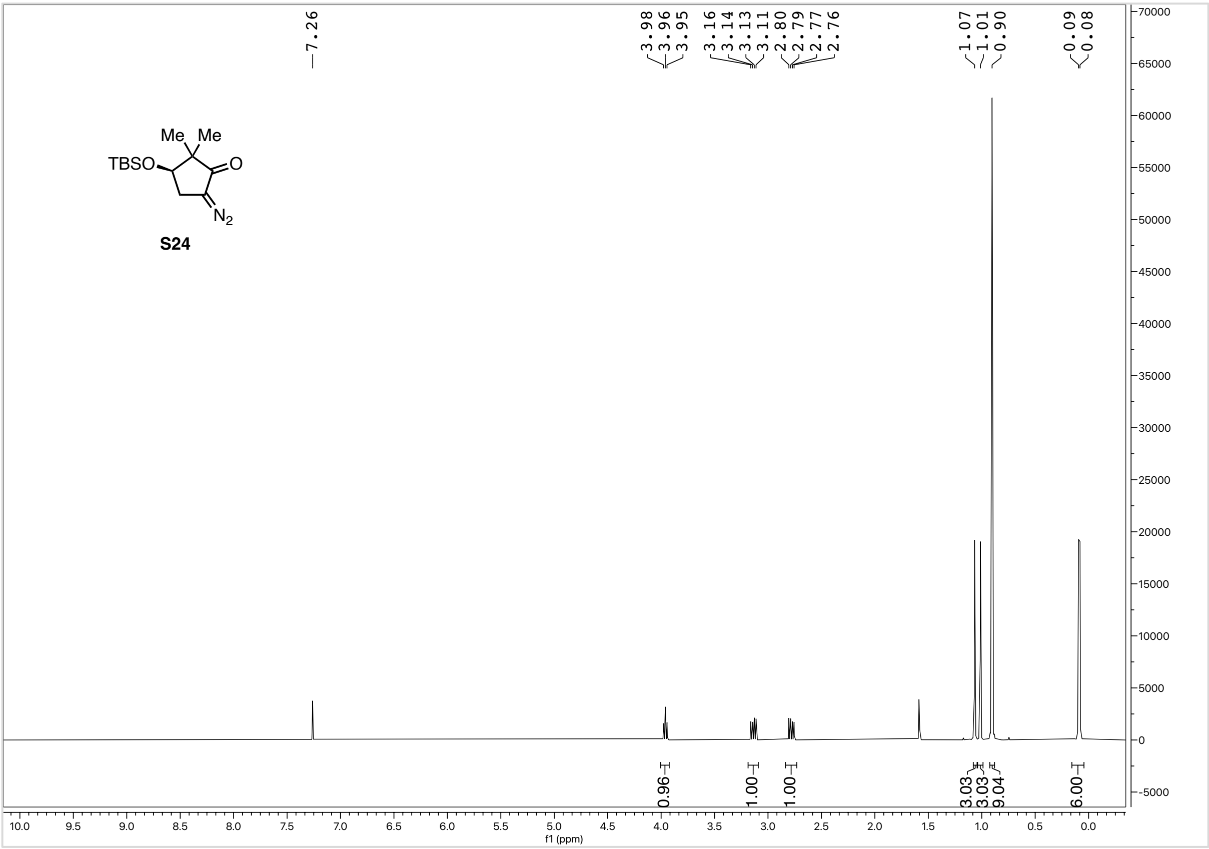

^{13}C NMR (101 MHz, $CDCl_3$) of **S24**

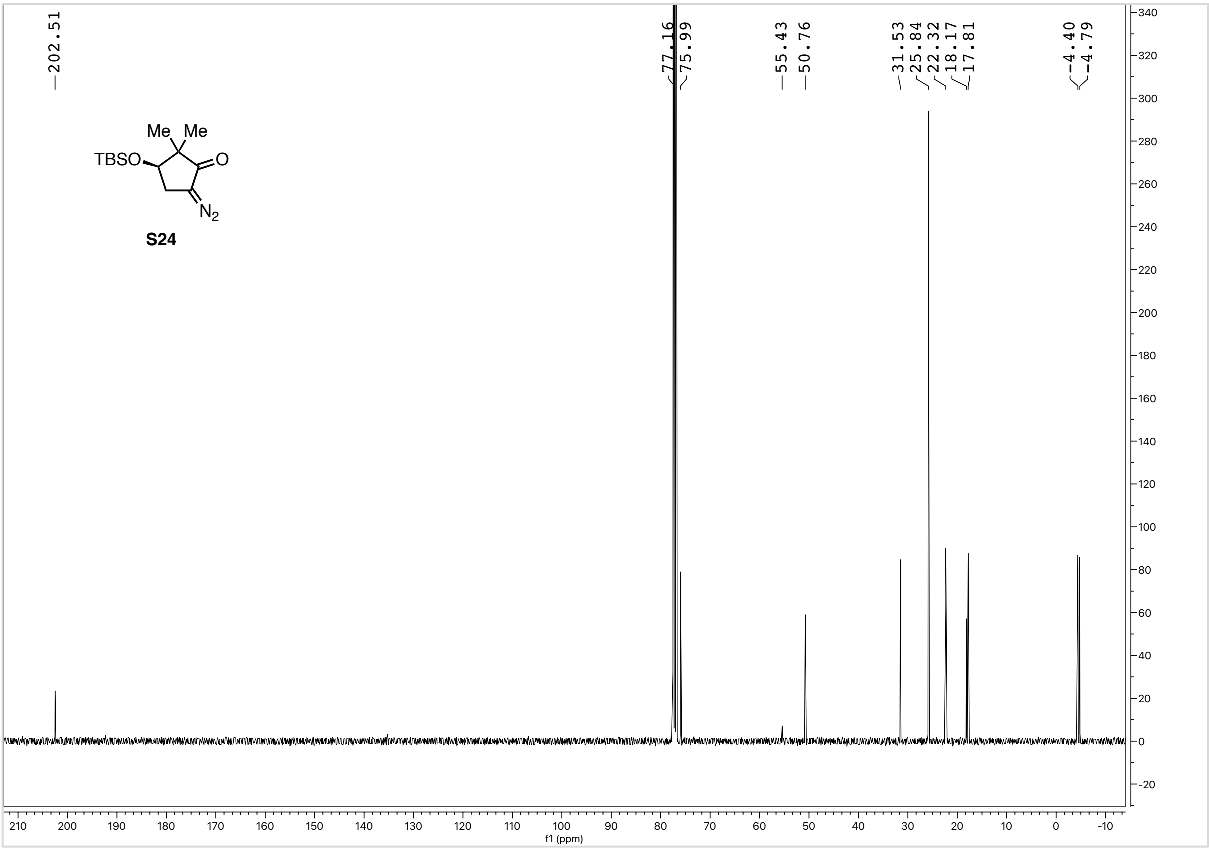

^{1}H NMR (400 MHz, $CDCl_3$) of **S25**/**C24-*epi*-S25** (1:1.3 inseparable diastereomers)

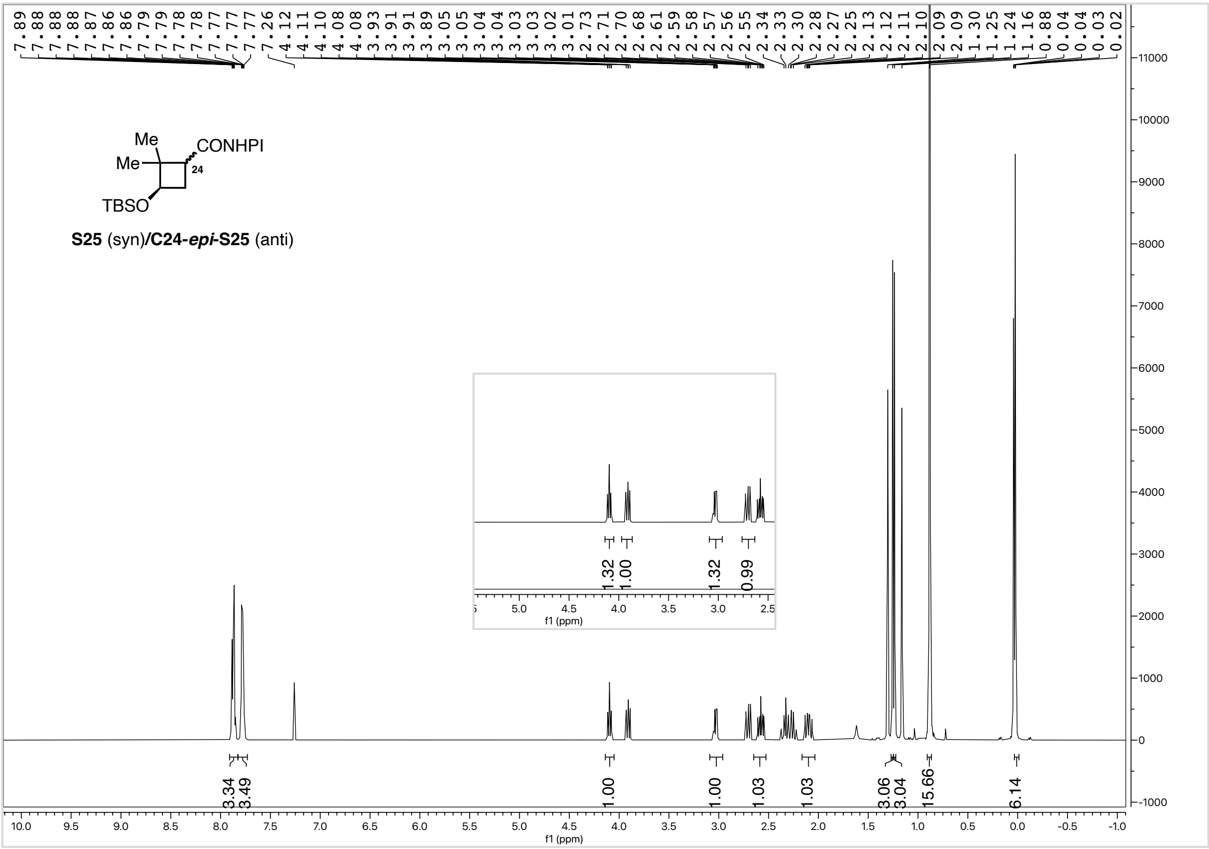

^{1}H NMR (400 MHz, $CDCl_3$) of **S25**/**C24-*epi*-S25** (1:1.3 inseparable diastereomers)

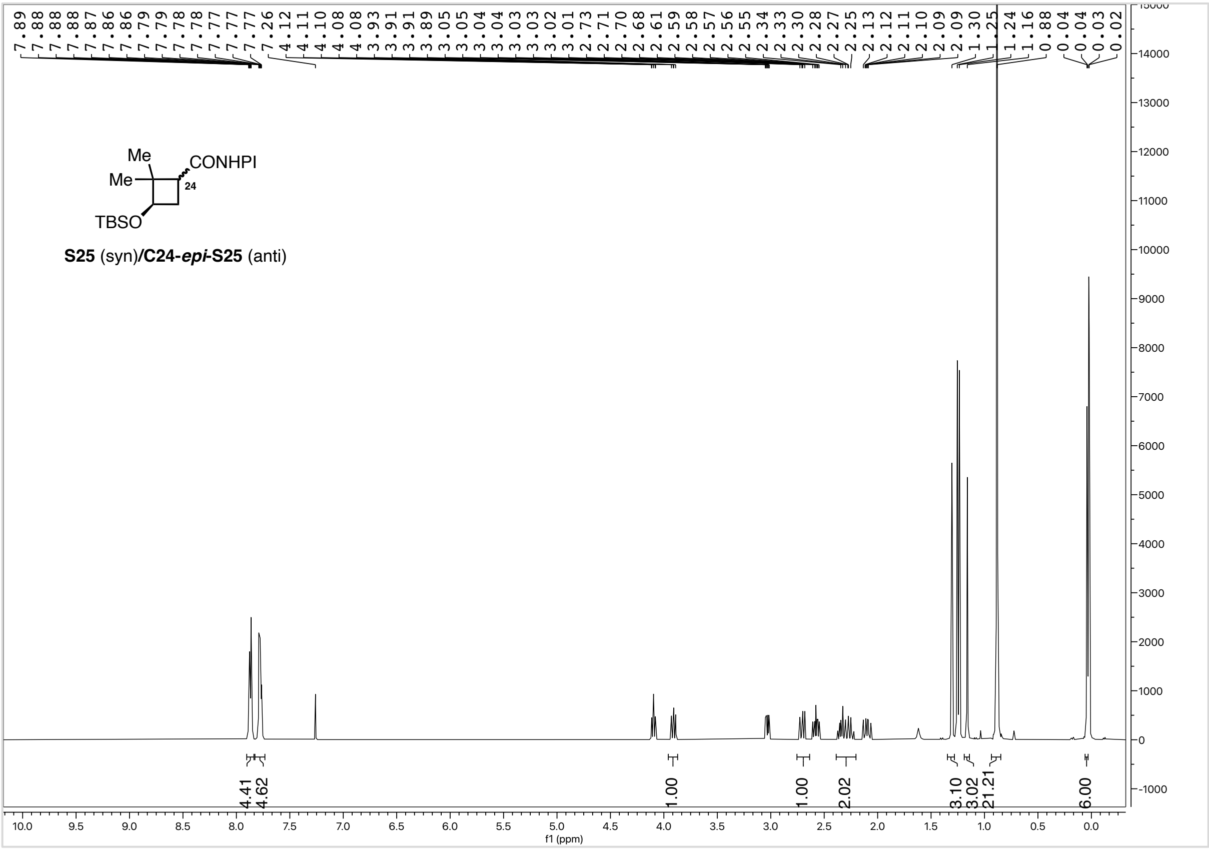

^{13}C NMR (101 MHz, $CDCl_3$) of **S25**/**C24-*epi*-S25** (1:1.3 inseparable diastereomer)

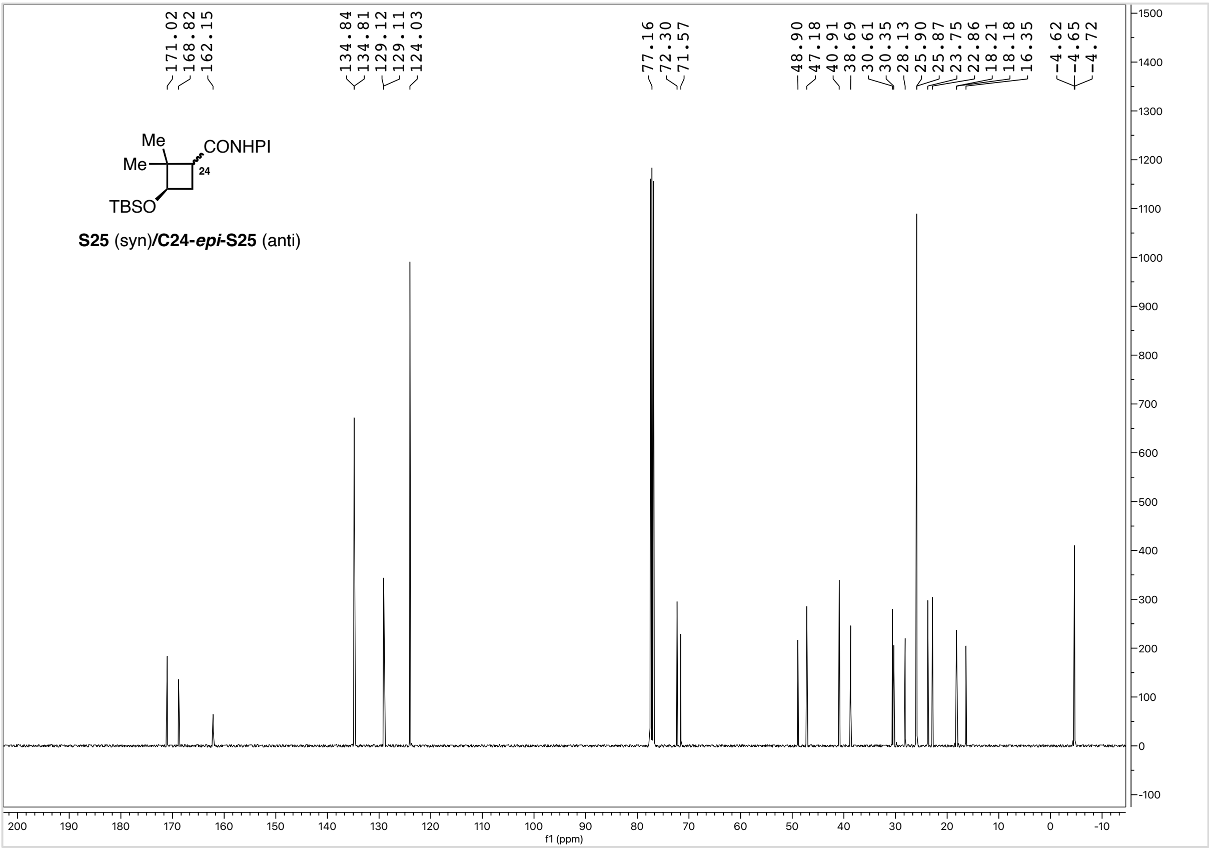

^{1}H NMR (400 MHz, $CDCl_3$) of **16/C24-*epi*-16** (1:1.4 inseparable mixture of diastereomers)

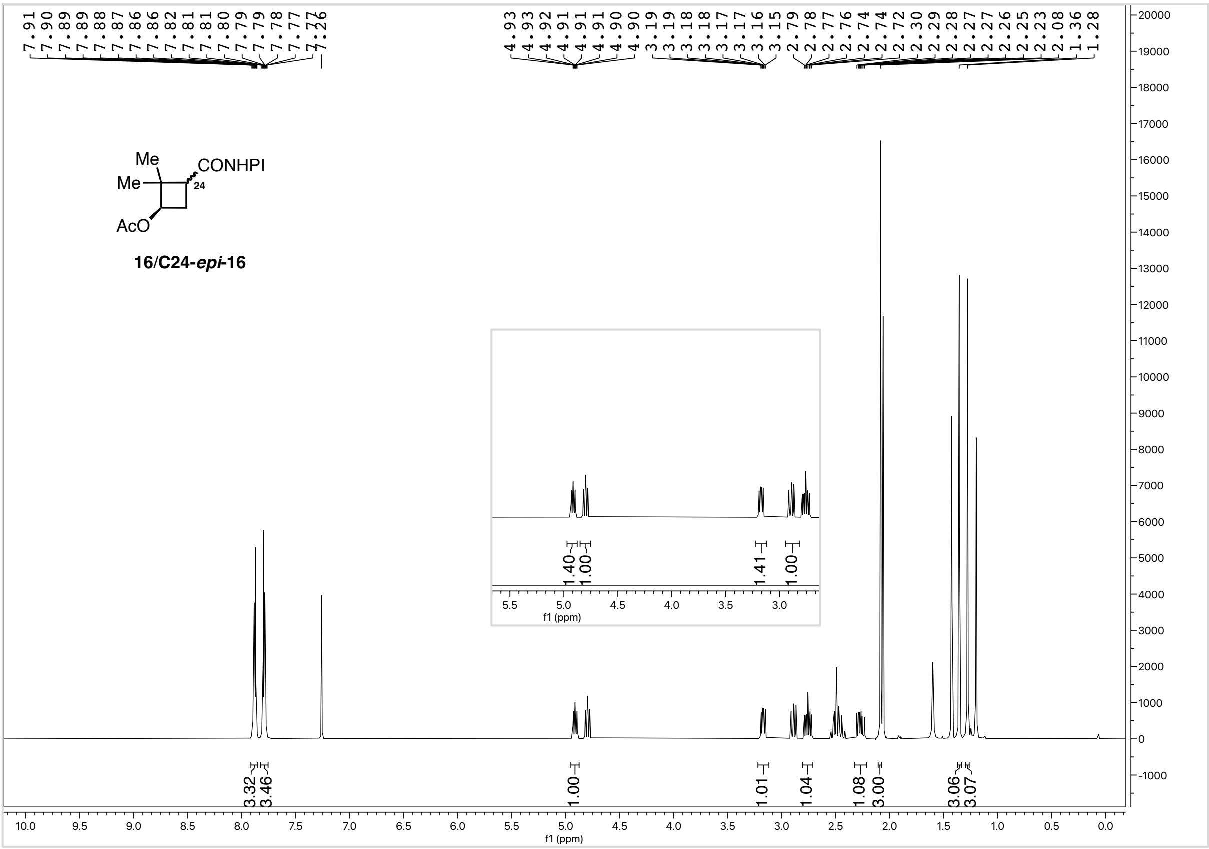

^{13}C NMR (101 MHz, $CDCl_3$) of **16/C24-*epi*-16** (1:1.4 inseparable mixture of diastereomer)

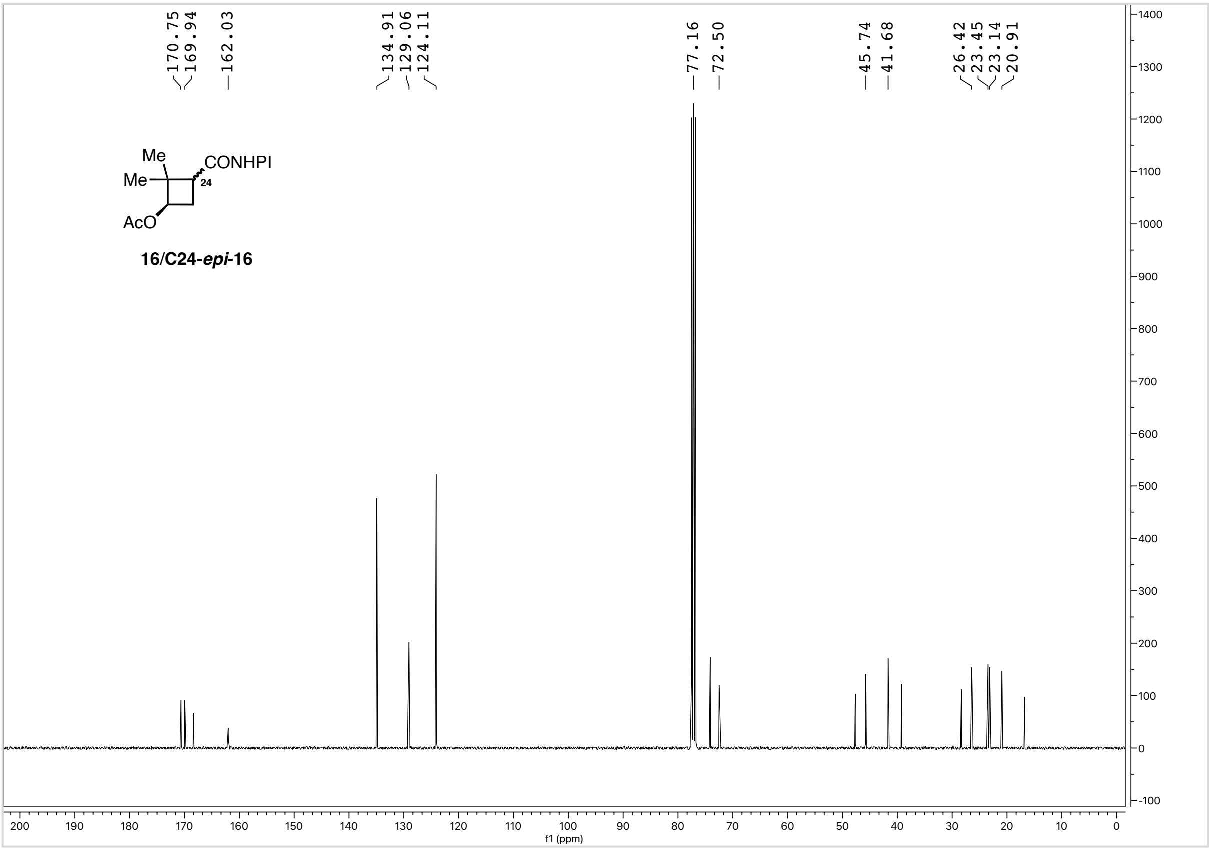

Supplement: Supplementary file 1 [file ja5c10325_si_001.pdf]
